# Synthesis of new pyrazolo[1,2,3]triazines by cyclative cleavage of pyrazolyltriazenes

**DOI:** 10.3762/bjoc.17.187

**Published:** 2021-11-22

**Authors:** Nicolai Wippert, Martin Nieger, Claudine Herlan, Nicole Jung, Stefan Bräse

**Affiliations:** 1 Institute of Biological and Chemical Systems – Functional Molecular Systems (IBCS-FMS), Karlsruhe Institute of Technology, Campus North, Hermann-von-Helmholtz-Platz 1, 76344 Eggenstein-Leopoldshafen, Germany; 2 Department of Chemistry, University of Helsinki, P.O. Box 55 (A. I. Virtasen aukio 1), 00014 University of Helsinki, Finland; 3 Institute of Organic Chemistry, Karlsruhe Institute of Technology, Fritz-Haber-Weg 6, 76131 Karlsruhe, Germany

**Keywords:** cyclization, diazonium chemistry, pyrazoles, triazenes, triazines

## Abstract

We describe the synthesis of so far synthetically not accessible 3,6-substituted-4,6-dihydro-3*H*-pyrazolo[3,4-*d*][1,2,3]triazines as nitrogen-rich heterocycles. The target compounds were obtained in five steps, including an amidation and a cyclative cleavage reaction as key reaction steps. The introduction of two side chains allowed a variation of the pyrazolo[3,4-*d*][1,2,3]triazine core with commercially available building blocks, enabling the extension of the protocol to gain other derivatives straightforwardly. Attempts to synthesize 3,7-substituted-4,7-dihydro-3*H*-pyrazolo[3,4-*d*][1,2,3]triazines, the regioisomers of the successfully gained 3,6-substituted 4,6-dihydro-3*H*-pyrazolo[3,4-*d*][1,2,3]triazines, were not successful under similar conditions due to the higher stability of the triazene functionality in the regioisomeric precursors and thus, the failure of the removal of the protective group.

## Introduction

The structural motif of pyrazolotriazines, particularly the pyrazolotriazinones, has drawn attention regarding a possible application as therapeutic agents due to manifold biological activities. Amongst other known constitutional isomers such as pyrazolo[4,3-*e*][1,2,4]triazines [1–3] and pyrazolo[1,5-*a*][1,3,5]triazines [4–6], pyrazolo[3,4-*d*][1,2,3]triazines [7–34] and their derivatives are literature known and subject of different biological studies. Pyrazolo[3,4-*d*][1,2,3]triazines and their derivatives, for example, were reported to function as anticancer compounds [28–29,32], herbicides [19–21], antimicrobials [18], and pest control agents [35].

**Scheme 1:**
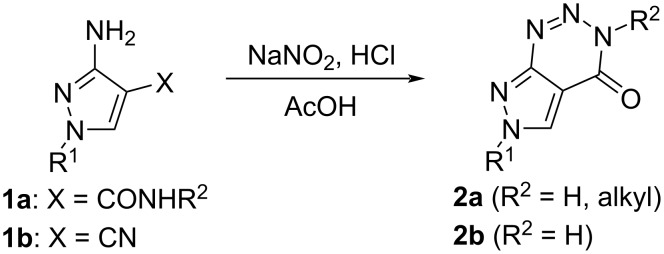
Synthesis of 3,6-dihydro-4*H*-pyrazolo[3,4-*d*][1,2,3]triazin-4-ones **2a**,**b** by diazotization of 3-amino-1*H*-pyrazole-4-carboxamides **1a** or 3-amino-1*H*-pyrazole-4-carbonitriles **1b** [22].

Several possibilities have been reported to gain the scaffold of pyrazolo[3,4-*d*][1,2,3]triazines synthetically and successful syntheses of different manifold isomers. 3,6-Dihydro-4*H*-pyrazolo[3,4-*d*][1,2,3]triazin-4-ones **2**, as one example of the diverse compound class, can be gained via diazotization of 3-amino-1*H*-pyrazole-4-carboxamides **1a** or 3-amino-1*H*-pyrazole-4-carbonitriles **1b** and subsequent cyclization of the intermediate diazo compounds under acidic conditions [22] (Scheme 1).

**Figure 1:**
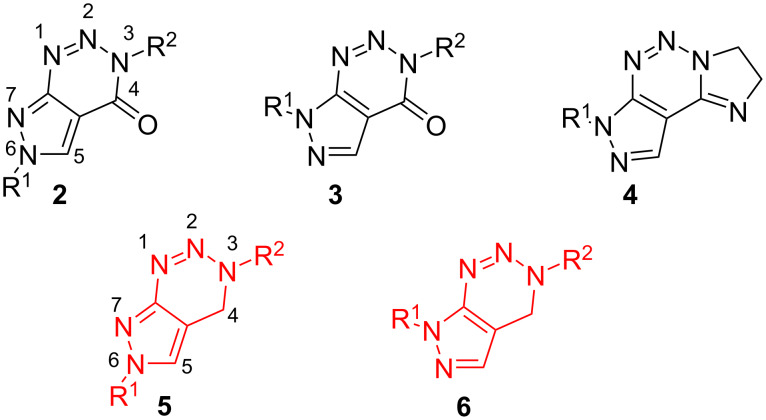
Structural differences of several known (**2**–**4**) and so far unknown (**5** and **6**) pyrazolo[3,4-*d*][1,2,3]-3*H*-triazine derivatives.

The structurally related 3,7-dihydro-4*H*-pyrazolo[3,4-*d*][1,2,3]triazin-4-ones **3** (Figure 1) which are substituted in position N-7 can be obtained in the same manner as described in Scheme 1, if the substitution position R^1^ in the starting 3-amino-1*H*-pyrazole-4-carboxamides **1a** or 3-amino-1*H*-pyrazole-4-carbonitriles **1b** is altered [26]. Furthermore, several 2,7-dihydro-3*H*-imidazo[1,2-*c*]pyrazolo[4,3-*e*][1,2,3]triazines **4** were described. However, while 3,6-substituted-3,6-dihydro-4*H*-pyrazolo[3,4-*d*][1,2,3]triazin-4-ones **2** and 3,7-substituted-3,7-dihydro-4*H*-pyrazolo[3,4-*d*][1,2,3]triazin-4-ones **3** are reported in several references dealing with their synthesis, modification, and application [36], their non-oxidized derivatives **5** and **6** are yet, to the best of our knowledge, unknown in the literature.

**Scheme 2:**
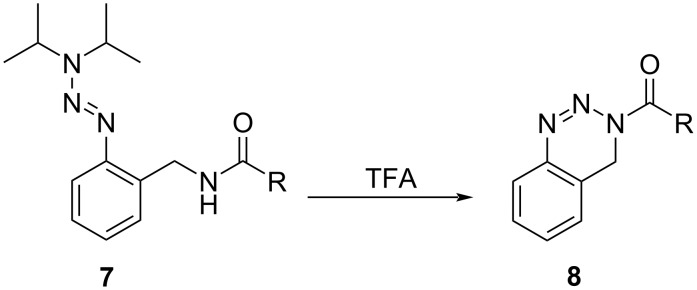
Synthesis of 3,4-dihydrobenzo[*d*][1,2,3]triazine derivatives **8** from triazene-containing precursors **7** [37].

In the past, it was shown that *ortho*-methylamide-substituted aryltriazenes **7** could be efficiently converted into 3,4-dihydrobenzo[*d*][1,2,3]triazine derivatives **8** [37] (Scheme 2).

In this context, triazenes have shown beneficial properties as they can be used as protected diazonium species which can be handled and converted in various transformations without decomposition [37–39]. In the herein presented study, we apply the cyclative cleavage reaction to pyrazolyltriazenes instead of aryltriazenes, which results in the synthesis of diverse pyrazolo[3,4-*d*][1,2,3]-3*H*-triazine derivatives **5**.

## Results and Discussion

**Scheme 3:**
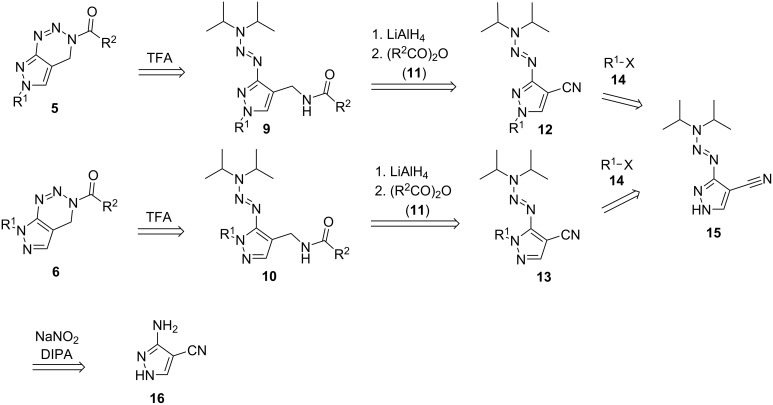
Planned retrosynthesis to obtain 4,6-dihydropyrazolo[3,4-*d*][1,2,3]-3*H*-triazines **5** and 4,7-dihydropyrazolo[3,4-*d*][1,2,3]-3*H*-triazines **6** from pyrazolylamines **16**. DIPA = diisopropylamine.

According to the literature-known synthetic access to benzotriazines **8**, we designed a retrosynthetic route consisting of five steps to gain 4,6-dihydropyrazolo[3,4-*d*][1,2,3]-3*H*-triazines **5** and 4,7-dihydropyrazolo[3,4-*d*][1,2,3]-3*H*-triazines **6** starting from pyrazolyltriazenes **15** (Scheme 3).

The sequence contains two key steps that have a major influence on the outcome of the reaction: (1) the addition of side chains R^1^ to the core pyrazole ring system, which can occur in position N-6 or N-7, and (2) the cyclative cleavage of the triazene group of compounds **9** and **10** which should lead to the target compounds **5** and **6**. To carry out the designed synthetic route, 3-(3,3-diisopropyltriaz-1-en-1-yl)-1*H*-pyrazole-4-carbonitrile (**15**) was synthesized in a first step using the commercially available 3-amino-1*H*-pyrazole-4-carbonitrile (**16**). Thus, the aminopyrazole was diazotized in aqueous media using hydrochloric acid and sodium nitrite. Diisopropylamine and an aqueous solution of potassium carbonate were added to the in-situ generated diazonium salt according to literature-known protocols [40]. The resulting 3-(3,3-diisopropyltriaz-1-en-1-yl)-1*H*-pyrazole-4-carbonitrile (**15**) was used as starting material for the attempts to add different side chains to the pyrazole moiety.

The addition of several aliphatic bromides or iodides **14** in combination with potassium or cesium carbonate in DMSO gave a mixture of the regioisomeric compounds **12** and **13** due to the addition of the alkyl substituents to one of both pyrazole-nitrogen atoms. As shown in Table 1, the alkylation protocol did not give a selective conversion in favor of one of the generated isomers. The protocol was not changed or adapted to gain a higher selectivity of one of the isomers, as both regioisomers were used in the subsequent syntheses.

**Table 1:** Synthesis of N-substituted pyrazoles **12** and **13**.

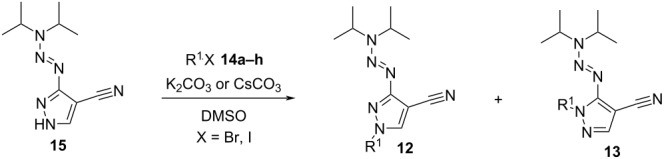					

entry^a^	**14** (equiv)	R^1^	X	**12** (yield)	**13** (yield)

1	**14a** (2.0)	Bn	Br	**12a** (54%)	**13a** (36%)
2	**14b** (1.5)	tolyl-CH_2_	Br	**12b** (59%)	**13b** (40%)
3	**14c** (2.0)	3,5-difluorobenzyl	Br	**12c** (48%)	**13c** (42%)
4^b^	**14d** (1.2)	ethyl	I	**12d** (34%)	**13d** (57%)
5^b^	**14e** (1.2)	cyclopentyl	Br	**12e** (39%)	**13e** (52%)
6	**14f** (1.2)	isobutyl	Br	**12f** (28%)	**13f** (47%)
7^c^	**14g** (1.1)	EtO_2_COCH_2_	Br	**12g** (54%)	**13g** (15%)
8^b^	**14h** (2.0)	4-bromobenzyl	Br	**12h** (51%)	**13h** (42%)

^a^Typical conditions for the conversion are: **15**, Cs_2_CO_3_ or K_2_CO_3_ (1.2 equiv), **14** (1.1–2.0 equiv), DMSO, room temperature; the products **12** and **13** were separated by column chromatography. ^b^The conditions were varied in temperature (reaction temperature was 80 °C for entries 4 and 5 and 40 °C for entry 8. ^c^0.95 equiv of K_2_CO_3_ were used.

**Figure 2:**
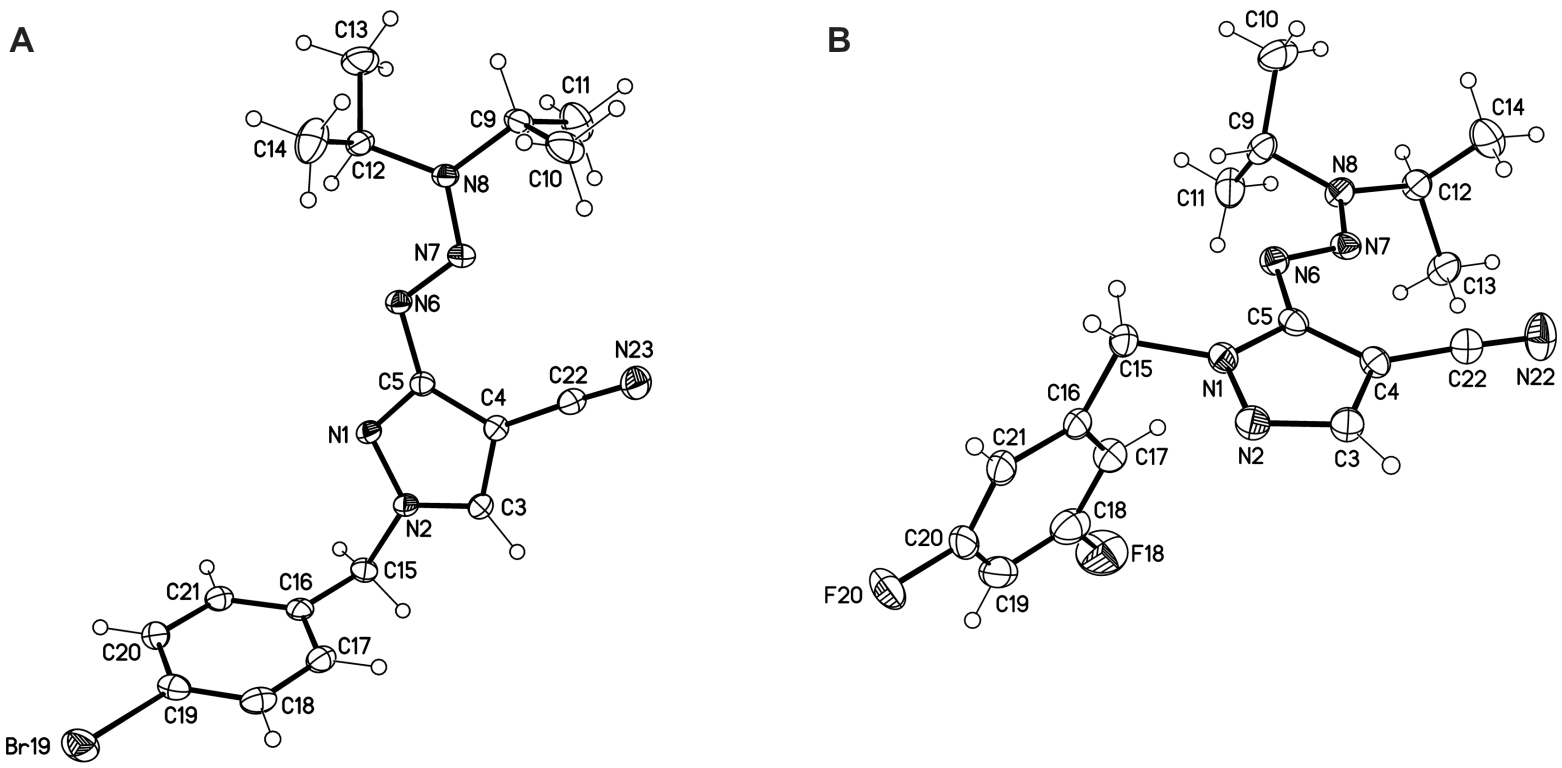
Molecular structures of compounds **12h** (A) and **13c** (B) representing both possible regioisomers of the alkylation reaction (displacement parameters are drawn at the 50% probability level). The X-ray data corroborate the obtained NMR data.

For the derivatives **12h** and **13c**, we were able to exemplarily determine the molecular structure by X-ray crystallography, proving the regioisomer obtained in the alkylation reaction (Figure 2).

While isomer **12** was used for the synthesis of pyrazolo[3,4-*d*][1,2,3]-3*H*-triazine derivatives of general structure **5**, the isomer **13** was intended to deliver pyrazolo[3,4-*d*][1,2,3]-3*H*-triazine derivative of general structure **6**. The synthetic sequence to compounds **5** is described in detail in the following sections. The synthesis of the precursors to compound **6** derived from **13** is shifted to Supporting Information File 1 as the cyclative cleavage to the final product **6** failed in the last step of the synthesis.

The conversion of **12** to the reduced aminomethyl compounds **17** was challenging. While the reduction with LiAlH_4_ in THF gave good results (shown via TLC and LC–MS analysis), the isolated products were not stable and degraded quickly, making full characterization impossible. Therefore, the crude aminomethyl compounds **17a**–**g** were directly converted to the corresponding amides **9a**–**i** using different anhydrides or acid chlorides **11a**–**c**. The resulting amides were stable and gained mediocre to good yields except for amides **9a** and **9d** (Table 2). The yield of **9d** was found to be very low due to a reductive replacement of the fluoro atoms of the benzyl ring during the reduction of the nitrile with LiAlH_4_. Only a small amount (5% yield) of the desired compound was isolated. Also, a reductive replacement was observed during the conversion of **12h**, yielding the intermediate **17a** with R^1^ = Bn instead of R^1^ = bromobenzyl. Depending on the nature of the side chain R^1^ in compounds **12a**–**g**, the reduction to compounds **17a**–**g** leads to a change of R^1^ to a different side chain R^1'^ which can be used for further transformation to R^1''^ by conversion with electrophiles. This was shown with compound **12g** (R^1^ = -CH_2_CO_2_Et), being reduced to compound **17g** (R^1'^ = -(CH_2_)_2_OH) and acylated to **9i** with R^1''^ = -(CH_2_)_2_OCOMe.

**Table 2:** Synthesis of amides **9a**–**i** via reduction of nitriles **12a**–**g** to pyrazolo-*ortho*-methylamines and subsequent conversion with aliphatic anhydrides or chlorides **11a**–**c**.

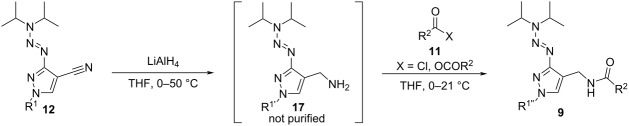								

entry^a^	**12**	R^1^ (**12**)	**17**	**11**	**9**	R^1''^ (**9**)	R^2^	yield **9** (%)

1	**12a**	Bn	**17a**	**11a**	**9a**	Bn	Me	23
2^b^	**12a**	Bn	**17a**	**11b**	**9b**	Bn	Ph	41
3	**12b**	4-methylbenzyl	**17b**	**11c**	**9c**	*p*-tolyl-CH_2_	iBu	61
4^b^	**12c**	3,5-difluorobenzyl	**17c**	**11a**	**9d**	3,5-difluorobenzyl	Me	5
5	**12d**	ethyl	**17d**	**11a**	**9e**	ethyl	Me	59
6	**12e**	cyclopentyl	**17e**	**11a**	**9f**	cyclopentyl	Me	74
7	**12e**	cyclopentyl	**17e**	**11b**	**9g**	cyclopentyl	Ph	63
8	**12f**	isobutyl	**17f**	**11a**	**9h**	isobutyl	Me	52
9	**12g**	EtCO_2_CH_2_	**17g**	**11a**	**9i**	MeCO_2_(CH_2_)_2_	Me	72

^a^The reaction consists of two steps. The intermediate compound **17** was isolated but not purified and used as obtained. Conditions: first step: **12**, LiAlH_4_ (3.0 equiv), THF, 0 °C to 21 °C, then 50 °C. Second step: **11** (X = OCOR^2^) (1.5 equiv), THF, 0 °C to 21 °C. ^b^In a modified protocol, acid chlorides were used instead of anhydrides to introduce R^2^. The second step was altered as follows: **11** (X = Cl) (1.5 equiv), THF, NEt_3_ (3.0 equiv), 0 °C to 21 °C.

**Scheme 4:**
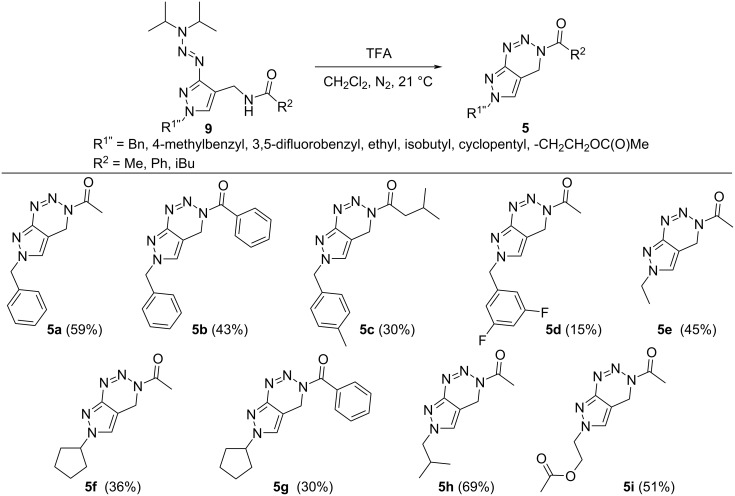
Cleavage of the triazene protective group and cyclization of the resulting diazonium intermediate yielding pyrazolo[3,4-*d*][1,2,3]-3*H*-triazine derivatives **5a**–**i**.

The last step in the synthesis of the target compounds **5a**–**i** included the cleavage of the triazene unit of the amides **9** with subsequent cyclization to the final pyrazolo[3,4-*d*][1,2,3]triazine compounds **5** (Scheme 4). The successful cyclizations gave the desired pyrazolo[3,4-*d*][1,2,3]triazines **5** in moderate to good yields. Not all cyclization products were air-stable. While compounds **5a**–**d** with a benzylic side chain in R^1''^ were stable, a full characterization was possible, especially pyrazolotriazines with an aliphatic substituent on the pyrazole-nitrogen (**5e**–**i**) degraded rapidly in contact with air/moisture.

The conversion of the regioisomeric compounds **10** to **6** failed under the conditions described in Scheme 4. The triazene protective group could not be cleaved even under harsh conditions (temperatures up to 100 °C in dichloroethane and use of H_2_SO_4_ as acid), and the starting material was recovered in all of the reactions.

Selected final compounds **5** and intermediates **9**, **12**, **13**, and **17** obtained in this work were tested for their cytotoxicity. We conducted standardized MTT assays [41] to evaluate if the newly accessible compounds of type **5** and their precursors could become interesting target molecules for biological investigations or if the compounds show high toxicity, which might prevent their use. We monitored cytotoxicity at six different concentrations ranging from 0.5 µM to 50 µM (for detailed results, see Supporting Information File 1). It was found that the exemplarily chosen compounds of type **5**, namely **5a**, **5d**–**f**, and **5h**, did not reduce the viability of the human epithelial cervix carcinoma (HeLa) cells at every concentration tested. The derivatives of the target compounds of class **5** were chosen as they were available in sufficient amounts and showed no decomposition during storage and dilution in DMSO. Also, most of the intermediates showed no reduction of cell viability; however, compounds **9b**, **12b**,**c**, **13a**,**b**, and **13f**–**h** showed some cytotoxic effects at high concentrations.

**Figure 3:**
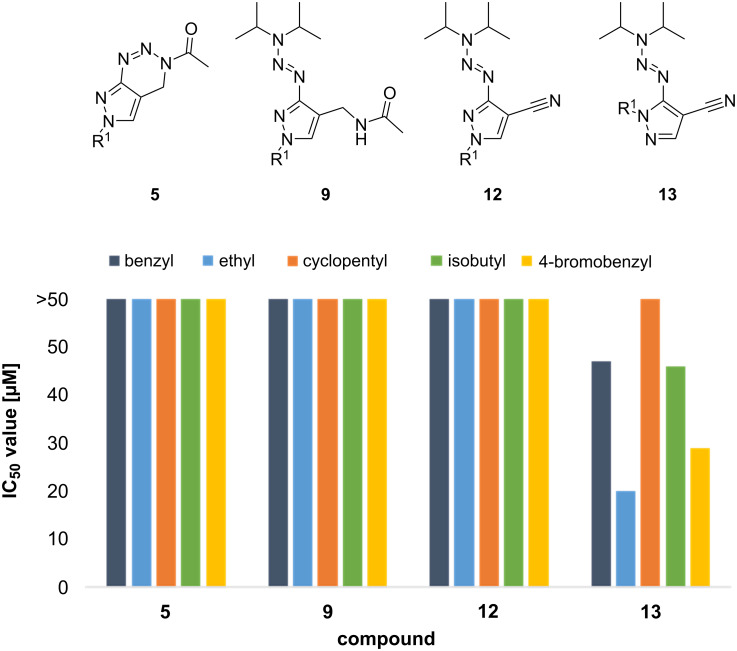
Graphical overview about selected pyrazolo[1,2,3]triazines **5** and intermediates **9**, **12**, and **13** and their calculated IC_50_ values after treatment of HeLa cells with different concentrations of the respective compounds.

Interestingly, no common structural motif promoted an increase in the in vitro cytotoxicity. However, by comparing the IC_50_ values of the compound classes **12** and **13**, regioisomerism seems to play a decisive role: while compounds **12a** and **12d**–**h** had no influence on the viability of HeLa cells, their regioisomers **13a** and **13d**–**h** decreased the viability at high micromolar concentrations. A slightly increased cytotoxicity of some derivatives of compound class **12** compared to **13** was observed for **12b** and **12c**. The amides **9** had no influence on the viability except for derivate **9b**, characterized by two aromatic moieties at both variable positions R^1^ and R^2^. In Table S3 (Supporting Information File 1), the results for compounds **5**, **9**, **12**, and **13** are summarized allowing the direct comparison of the toxicity of the four compound classes with respect to 5 different residues R^1^. The full data of the toxicity studies for all obtained compounds are given in Supporting Information File 1, Tables S1–S3. Comparing the 20 derivatives depicted in Figure 3 reveals that compounds of the classes **5**, **9**, and **12** are in general less toxic than the respective compounds of class **13**, at least with respect to the derivatives that were obtained in this study.

As the IC_50_ value of every compound tested lies above the concentration range that is interesting for biological applications, we consider molecules of type **5** as feasible for further biological screenings. We will continue our studies to search for potential targets for the versatile pyrazolo[1,2,3]triazine library presented herein.

## Conclusion

In analogy to literature-known acid-induced conversions of triazene-benzyl acetamides to 3,4-dihydrobenzo[*d*][1,2,3]triazines, so far not described pyrazolo[3,4-*d*][1,2,3]-3*H*-triazines **5** were successfully synthesized. Altogether nine derivatives **5a**–**i** were synthesized in five steps starting from the commercially available 3-(3,3-diisopropyltriaz-1-en-1-yl)-1*H*-pyrazole-4-carbonitrile. The herein given examples were generated by introducing two side-chains, one on the pyrazole core and the other as a side chain added to a methylamine intermediate in step 2 and step 4 of the reaction sequence. Depending on the introduced side chain, further modifications were obtained (shown for compound **9g**). The triazene protective group tolerates the reaction conditions used for the described processes and is probably compatible with many others described in the literature. However, limitations are given to acidic reaction media, which tend to cleave the protective group. So far, the attempts to synthesize compounds of general structure **6**, a regioisomer of the successfully gained pyrazolo[3,4-*d*][1,2,3]-3*H*-triazines **5**, failed under similar procedures.

## Abbreviations

TFA, trifluoroacetic acid; DMSO, dimethyl sulfoxide; THF, tetrahydrofuran, TLC, thin-layer chromatography, LC–MS, liquid chromatography/mass spectrometry.

## Supporting Information

The Supporting Information contains detailed descriptions of the reactions and protocols as well as the characterization of all target compounds.

File 1 Experimental section and characterization data, biological assay details, and data availability in chemotion repository.

File 2 Copies of spectra.

File 3 Direkt links to datasets and reference numbers of target compounds in Molecule Archive.
