# Supplementary material for: Synthesis of new pyrazolo[1,2,3]triazines by cyclative cleavage of pyrazolyltriazenes
Source: Beilstein J Org Chem. 2021 Nov 22;17:2773–80. doi: 10.3762/bjoc.17.187 (PMC8630434; doi:10.3762/bjoc.17.187)
Supplement: File 1 — Experimental section and characterization data, biological assay details, and data availability in chemotion repository. [file Beilstein_J_Org_Chem-17-2773-s001.pdf]

BEILSTEIN JOURNAL OF ORGANIC CHEMISTRY

Supporting Information

for

Synthesis of new pyrazolo[1,2,3]triazines by cyclative cleavage of pyrazolyltriazenes

Nicolai Wippert, Martin Nieger, Claudine Herlan, Nicole Jung and Stefan Bräse

Beilstein J. Org. Chem. **2021,** *17,* 2773–2780. doi:10.3762/bjoc.17.187

Experimental section and characterization data, biological assay details, and data availability in chemotion repository

License and Terms: This is a supporting information file under the terms of the Creative Commons Attribution License (https://creativecommons.org/licenses/by/4.0). Please note that the reuse, redistribution and reproduction in particular requires that the author(s) and source are credited and that individual graphics may be subject to special legal provisions.
The license is subject to the *Beilstein Journal of Organic Chemistry* terms and conditions: (https://www.beilstein-journals.org/bjoc/terms)

Table of contents

1 General remarks S2

2 Synthesis and characterization S3

(*E*)-1-Benzyl-3-(3,3-diisopropyltriaz-1-en-1-yl)-1*H*-pyrazole-4-carbonitrile (**12a**), (*E*)-1-benzyl-5-(3,3-diisopropyltriaz-1-en-1-yl)-1*H*-pyrazole-4-carbonitrile (**13a**) S3

(*E*)-3-(3,3-Diisopropyltriaz-1-en-1-yl)-1-(4-methylbenzyl)-1*H*-pyrazole-4-carbonitrile (**12b**), (*E*)-5-(3,3-diisopropyltriaz-1-en-1-yl)-1-(4-methylbenzyl)-1*H*-pyrazole-4-carbonitrile (**13b**) S4

(*E*)-1-(3,5-Difluorobenzyl)-3-(3,3-diisopropyltriaz-1-en-1-yl)-1*H*-pyrazole-4-carbonitrile (**12c**), (*E*)-1-(3,5-difluorobenzyl)-5-(3,3-diisopropyltriaz-1-en-1-yl)-1*H*-pyrazole-4-carbonitrile (**13c**) S5

(*E*)-3-(3,3-Diisopropyltriaz-1-en-1-yl)-1-ethyl-1*H*-pyrazole-4-carbonitrile (**12d**), (*E*)-5-(3,3-diisopropyl-triaz-1-en-1-yl)-1-ethyl-1*H*-pyrazole-4-carbonitrile (**13d**) S7

(*E*)-1-Cyclopentyl-3-(3,3-diisopropyltriaz-1-en-1-yl)-1*H*-pyrazole-4-carbonitrile (**12e**), (*E*)-1-cyclo-pentyl-5-(3,3-diisopropyltriaz-1-en-1-yl)-1*H*-pyrazole-4-carbonitrile (**13e**) S9

(*E*)-3-(3,3-Diisopropyltriaz-1-en-1-yl)-1-isobutyl-1*H*-pyrazole-4-carbonitrile (**12f**), (*E*)-5-(3,3-diisopropyltriaz-1-en-1-yl)-1-isobutyl-1*H*-pyrazole-4-carbonitrile (**13f**) S10

Ethyl (*E*)-2-(4-cyano-3-(3,3-diisopropyltriaz-1-en-1-yl)-1*H*-pyrazol-1-yl)acetate (**12g**), ethyl (*E*)-2-(4-cyano-5-(3,3-diisopropyltriaz-1-en-1-yl)-1*H*-pyrazol-1-yl)acetate (**13g**) S10

(*E*)-1-(4-Bromobenzyl)-3-(3,3-diisopropyltriaz-1-en-1-yl)-1*H*-pyrazole-4-carbonitrile (**12h**), (*E*)-1-(4-bromobenzyl)-5-(3,3-diisopropyltriaz-1-en-1-yl)-1*H*-pyrazole-4-carbonitrile (**13h**) S11

(*E*)-*N*-((1-Benzyl-3-(3,3-diisopropyltriaz-1-en-1-yl)-1*H*-pyrazol-4-yl)methyl)acetamide (**9a**) S12

(*E*)-*N*-((1-Benzyl-3-(3,3-diisopropyltriaz-1-en-1-yl)-1*H*-pyrazol-4-yl)methyl)benzamide (**9b**) S13

(*E*)-*N*-((3-(3,3-Diisopropyltriaz-1-en-1-yl)-1-(4-methylbenzyl)-1*H*-pyrazol-4-yl)methyl)-3-methylbutanamide (**9c**) S14

(*E*)-*N*-((1-(3,5-Difluorobenzyl)-3-(3,3-diisopropyltriaz-1-en-1-yl)-1*H*-pyrazol-4-yl)methyl)acet-amide (**9d**) S15

(*E*)-*N*-((3-(3,3-Diisopropyltriaz-1-en-1-yl)-1-ethyl-1*H*-pyrazol-4-yl)methyl)acetamide (**9e**) S16

(*E*)-*N*-((1-Cyclopentyl-3-(3,3-diisopropyltriaz-1-en-1-yl)-1*H*-pyrazol-4-yl)methyl)acetamide (**9f**) S17

(*E*)-*N*-((1-Cyclopentyl-3-(3,3-diisopropyltriaz-1-en-1-yl)-1*H*-pyrazol-4-yl)methyl)benzamide (**9g**) S18

(*E*)-*N*-((3-(3,3-Diisopropyltriaz-1-en-1-yl)-1-isobutyl-1*H*-pyrazol-4-yl)methyl)acetamide (**9h**) S19

(*E*)-2-(4-(Acetamidomethyl)-3-(3,3-diisopropyltriaz-1-en-1-yl)-1*H*-pyrazol-1-yl)ethyl acetate (**9i**) S20

1-(6-Benzyl-4,6-dihydro-3*H*-pyrazolo[3,4-*d*][1,2,3]triazin-3-yl)ethan-1-one (**5a**) S21

(6-Benzyl-4,6-dihydro-3*H*-pyrazolo[3,4-*d*][1,2,3]triazin-3-yl)(phenyl)methanone (**5b**) S21

3-Methyl-1-(6-(4-methylbenzyl)-4,6-dihydro-3*H*-pyrazolo[3,4-*d*][1,2,3]triazin-3-yl)butan-1-one (**5c**) S22

1-(6-(3,5-Difluorobenzyl)-4,6-dihydro-3*H*-pyrazolo[3,4-*d*][1,2,3]triazin-3-yl)ethan-1-one (**5d**) S23

1-(6-Ethyl-4,6-dihydro-3*H*-pyrazolo[3,4-*d*][1,2,3]triazin-3-yl)ethan-1-one (**5e**) S24

1-(6-Cyclopentyl-4,6-dihydro-3*H*-pyrazolo[3,4-*d*][1,2,3]triazin-3-yl)ethan-1-one (**5f**) S24

(6-Cyclopentyl-4,6-dihydro-3*H*-pyrazolo[3,4-*d*][1,2,3]triazin-3-yl)(phenyl)methanone (**5g**) S25

1-(6-Isobutyl-4,6-dihydro-3*H*-pyrazolo[3,4-*d*][1,2,3]triazin-3-yl)ethan-1-one (**5h**) S26

2-(3-Acetyl-3,4-dihydro-6*H*-pyrazolo[3,4-*d*][1,2,3]triazin-6-yl)ethyl acetate (**5i**) S26

3. Amidation of triazenes **13** S28

4. Crystal structures of compounds **12h** and **13c** S33

5. Biological assays S35

6. References S46

1 General remarks

NMR spectra of dissolved samples were recorded on a Bruker Ascend 400 at 21 °C. Chemical shifts (δ) are expressed in parts per million (ppm) downfield from tetramethylsilane (TMS). References for ^{1}H NMR and ^{13}C NMR were the residual solvent peaks of chloroform-d_1 (^{1}H: δ = 7.26 ppm; ^{13}C: δ = 77.0 ppm) if not stated otherwise. All coupling constants (*J*) are absolute values and are expressed in hertz (Hz). The description of signals includes: s = singlet, d = doublet, t = triplet, m = multiplet, dd = doublet of doublets, and ddd = double doublet of doublets and so forth. The spectra were analyzed according to the first order. The assignments of the signal structure in ^{1}H NMR were made by the detected multiplicity and for ^{13}C NMR by DEPT 90- and DEPT 135-spectra (DEPT = distortionless enhancement by polarization transfer) and are described as follows: + = primary or tertiary C-atom (positive DEPT-signal), − = secondary C-atom (negative signal) and C_q = quaternary C-atom (no signal). Furthermore, 2D experiments such as COSY (correlated spectroscopy) and HSQC (heteronuclear single quantum coherence) were performed.

IR spectra were recorded on a Bruker Alpha ATR spectrometer. The compounds were measured as pure substances by the ATR (ATR = attenuated total reflection) technique. The position of the absorption band is given in wave numbers ṽ in cm^{-1}. The intensities of the bands were characterized as follows: vs = very strong (0–20% T), s = strong (21–40 % T), m = medium (41–60% T), w = weak (61–80% T), vw = very weak (81–100% T).

Mass spectra were measured by EIMS (electron impact mass spectrometry) and were recorded on a Finnigan MAT 95. The peaks are given as mass-to-charge-ratio (*m/z*). The molecule peak is given as $[M]^+$ and characteristic fragment peaks are given as $[M-fragment]^+$ or $[fragment]^+$. The signal intensities are given in percent, relatively to the intensity of the base signal (100%). For the high resolution mass, the following abbreviations were used: calcd = calculated data, found = measured data. The software of FAB and EI adds the mass of one electron.

Analytical thin layer chromatography (TLC) was carried out on Merck silica gel coated aluminum plates (silica gel 60, F_{254}), detected under UV light at 254 nm or stained with “Seebach staining solution” (mixture of molybdato phosphoric acid, cerium(IV) sulfate tetrahydrate, sulfuric acid and water) or basic potassium permanganate solution. Solvent mixtures are understood as volume/volume.

All solvents, reagents and chemicals were used as purchased unless stated otherwise.

Air or moisture-sensitive reactions were carried out under argon atmosphere in oven-dried and previously evacuated glass ware. Liquids were transferred with plastic syringes and steel cannula. Silica gel 60 (0.040 × 0.063 mm, Geduran®, Merck) was used as stationary phase and solvents of *p.a.* quality were used as mobile phase.

InChI Strings were generated with InChI Version (1.04), Smiles with the SMILES Version of Daylight.

2 Synthesis and characterization

5-[(*E*)-[Di(propan-2-yl)amino]diazenyl]-1*H*-pyrazole-4-carbonitrile (15)

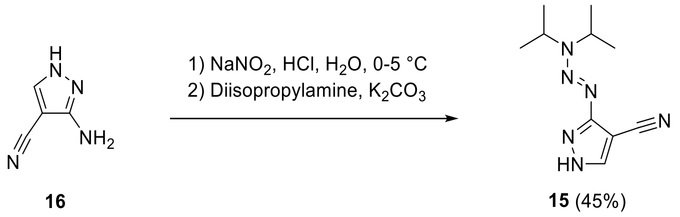

15: 5-[(~{E})-[di(propan-2-yl)amino]diazenyl]-1~{H}-pyrazole-4-carbonitrile; Formula: $C_{10}H_{16}N_6$; Exact Mass: 220.1436; Smiles: CC(N(C(C)C)/N=N/c1n[nH]cc1C#N)C; InChIKey: UCPMQTTXXMXIFF-CCEZHUSRSA-N.

To a mixture of 5-amino-1*H*-pyrazole-4-carbonitrile (2.00 g, 19 mmol, 1.00 equiv) in 6 mL of water, conc. hydrochloric acid (6.17 mL, 74 mmol, 4.00 equiv) was added. The mixture was cooled to 0 °C and a solution of sodium nitrite (1.91 g, 28 mmol, 1.50 equiv) in 20 mL of water was added. Additional 10 mL of water were added to get a stirrable slurry. After stirring for 30 min at 0 °C, a mixture of diisopropylamine (2.43 g, 3.37 mL, 24 mmol, 1.30 equiv) and dipotassium carbonate (5.11 g, 37 mmol, 2.00 equiv) in 50 mL of water were added. The reaction mixture was stirred at 21 °C until TLC showed that all diazonium salt had disappeared. The reaction mixture was extracted with 3 × 150 mL of DCM. Some precipitate was formed between the layers which had to be filtered off (clogged the frit). The combined organic phases were washed with 3 × 60 mL of water, dried over sodium sulfate, and the solvent was evaporated under reduced pressure to give the desired product. The obtained crude product was purified *via* flash chromatography on silica gel using cyclohexane/ethyl acetate 4:1 to 2:1 to give the target compound in 45% yield (1.85 g, 8.4 mmol).

R_f = 0.08 (cyclohexane/ethyl acetate 4:1). ^{1}H NMR (400 MHz, $CDCl_3$, ppm) δ = 9.04 (bs, 1H), 7.76 (s, 1H), 5.27 (hept, J = 6.80 Hz, 1H), 4.08 (hept, J = 6.7 Hz, 1H), 1.44 (d, J = 6.6 Hz, 6H), 1.28 (d, J = 6.8 Hz, 6H); ^{13}C NMR (100 MHz, $CDCl_3$, ppm) δ = 158.4, 141.3, 115.4 (+, CH), 79.7, 50.8 (+, CH), 47.6 (+, CH), 23.4 (+, 2C, CH_3), 19.2 (+, 2C, CH_3); EI (*m/z*, 70 eV, 80 °C): 220 (100) $[M]^+$, 135 (23), 134 (11), 120 (35), 109 (16), 108 (37), 100 (32), 86 (87), 84 (38), 70 (15), 69 (13), 65 (23), 58 (74), 52 (21); HRMS ($C_{10}H_{16}N_6$): Calcd 220.1436, Found 220.1438; IR (ATR, ṽ) = 611, 629, 643, 713, 722, 751, 771, 816, 839, 861, 880, 899, 928, 948, 1031, 1067, 1077, 1096, 1130, 1163, 1194, 1217, 1231, 1262, 1295, 1306, 1319, 1339, 1364, 1378, 1392, 1412, 1451, 1466, 1492, 1543, 1572, 1720, 1792, 2183, 2227, 2873, 2934, 2973, 3053, 3095, 3143, 3231 cm^{-1}.

Additional information on the chemical synthesis is available *via* Chemotion repository:
https://doi.org/10.14272/reaction/SA-FUHFF-UHFFFADPSC-UCPMQTTXXM-UHFFFADPSC-NUHFF-NRHPV-NUHFF-ZZZ
Additional information on the analysis of the target compound is available *via* Chemotion repository:
https://doi.org/10.14272/UCPMQTTXXMXIFF-CCEZHUSRSA-N.1

(*E*)-1-Benzyl-3-(3,3-diisopropyltriaz-1-en-1-yl)-1*H*-pyrazole-4-carbonitrile (12a), (*E*)-1-benzyl-5-(3,3-diisopropyltriaz-1-en-1-yl)-1*H*-pyrazole-4-carbonitrile (13a)

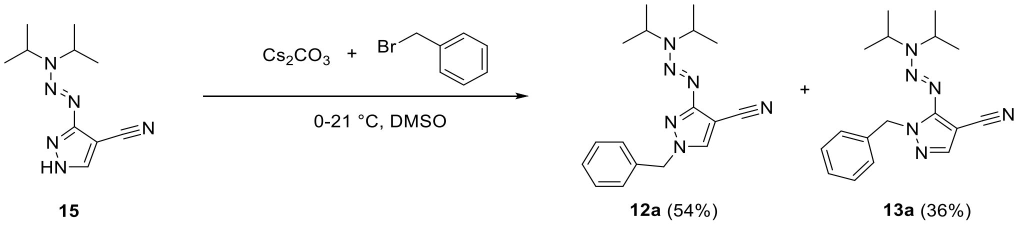

(*E*)-3-(3,3-Diisopropyltriaz-1-en-1-yl)-4-carbonitrile-1*H*-pyrazole (**15**, 76.5 mg, 347 µmol, 1.00 equiv) was dissolved in 10 mL of DMSO. Cesium carbonate (133 mg, 409 µmol, 1.18 equiv) was added and the solution was

cooled to 0 °C. Bromomethylbenzene (117 mg, 80.9 µL, 681 µmol, 1.96 equiv) was added and the vial was closed and slowly warmed to 21 °C. The reaction mixture was stirred at 21 °C for 48 hours. The reaction was quenched by addition of ice and was extracted with EtOAc (3 × 15 mL). The obtained organic layers were co-evaporated with Celite(R) to give the Celite-immobilized crude product. The obtained crude product was purified *via* flash chromatography on silica gel using cyclohexane/ethyl acetate 10:1 to 4:1, giving (*E*)-1-benzyl-3-(3,3-diisopropyltriaz-1-en-1-yl)-1*H*-pyrazole-4-carbonitrile (**12a**, 58.2 mg, 188 µmol, 54% yield) as a light-orange solid and (*E*)-1-benzyl-5-(3,3-diisopropyltriaz-1-en-1-yl)-1*H*-pyrazole-4-carbonitrile (**13a**, 38.7 mg, 125 µmol, 36% yield) as a light-orange solid.

12a: (E)-1-benzyl-3-(3,3-diisopropyltriaz-1-en-1-yl)-1H-pyrazole-4-carbonitrile; Formula: $C_{17}H_{22}N_6$; Exact Mass: 310.1906; Smiles: N#Cc1cn(nc1/N=N/N(C(C)C)C(C)C)Cc1ccccc1; InChIKey: AONLLYWWGOIMLR-XUTLUUPISA-N

R_f = 0.23 (cyclohexane/ethyl acetate 4:1). ^{1}H NMR (400 MHz, $CDCl_3$, ppm) δ = 7.53 (s, 1H), 7.30–7.15 (m, 5H), 5.32 (hept, *J* = 6.8 Hz, 1H), 5.13 (s, 2H), 3.93 (hept, *J* = 6.6 Hz, 1H), 1.35 (d, *J* = 6.6 Hz, 6H), 1.15 (d, *J* = 6.8 Hz, 6H); ^{13}C NMR (100 MHz, $CDCl_3$, ppm) δ = 162.9, 135.7 (+, CH), 134.8, 129.1 (+, 2C, CH), 128.8 (+, CH), 128.3 (+, 2C, CH), 115.3, 81.5, 56.9 (–, CH_2), 49.9 (+, CH), 46.5 (+, CH), 23.3 (+, 2C, CH_3), 19.3 (+, 2C, CH_3); EI (m/z, 70 eV, 80 °C): 310 (39) $[M]^+$, 210 (60), 181 (13), 131 (17), 100 (48), 92 (11), 91 (100), 86 (10), 84 (14), 77 (22), 71 (12), 70 (10), 69 (35), 58 (54), 57 (23), 55 (15). HRMS ($C_{17}H_{22}N_6$): Calcd 310.1906, Found 310.1905; IR (ATR, ṽ) = 3122 (w), 3058 (vw), 3031 (vw), 2979 (w), 2934 (w), 2868 (vw), 2223 (m), 1816 (vw), 1700 (vw), 1604 (vw), 1537 (s), 1497 (w), 1456 (m), 1412 (vs), 1368 (vs), 1353 (s), 1326 (w), 1313 (w), 1261 (vs), 1239 (m), 1227 (vs), 1184 (w), 1149 (vs), 1132 (m), 1095 (m), 1081 (w), 1028 (s), 1001 (m), 970 (w), 909 (w), 881 (w), 851 (m), 843 (m), 819 (w), 799 (w), 752 (w), 741 (s), 721 (m), 711 (vs), 694 (s), 649 (m), 632 (w) cm^{-1}.

13a: (E)-1-benzyl-5-(3,3-diisopropyltriaz-1-en-1-yl)-1H-pyrazole-4-carbonitrile; Formula: $C_{17}H_{22}N_6$; Exact Mass: 310.1906; Smiles: CC(N(C(C)C)/N=N/c1n(ncc1C#N)Cc1ccccc1)C; InChIKey: AQYSAXXLCHFEGV-QZQOTICOSA-N

R_f = 0.30 (cyclohexane/ethyl acetate 4:1). ^{1}H NMR (400 MHz, $CDCl_3$, ppm) δ = 7.65 (s, 1H), 7.35–7.21 (m, 5H), 5.36 (s, 2H), 5.16 (hept, *J* = 6.9 Hz, 1H), 4.08 (hept, *J* = 6.6 Hz, 1H), 1.44 (d, *J* = 6.6 Hz, 6H), 1.26 (d, *J* = 6.8 Hz, 6H). ^{13}C NMR (100 MHz, $CDCl_3$, ppm) δ = 154.8, 142.5 (+, CH), 136.5, 128.8 (+, 2C, CH), 128.0 (+, CH), 127.9 (+, 2C, CH), 116.0, 77.9, 52.3 (–, CH_2), 51.4 (+, CH), 48.1 (+, CH), 23.3 (+, 2C, CH_3), 19.0 (+, 2C, CH_3); EI (m/z, 70 eV, 70 °C): 311 (10) $[M+H]^+$, 310 (55) $[M]^+$, 210 (26), 181 (20), 131 (30), 119 (12), 100 (42), 91 (78), 69 (100), 58 (21). HRMS ($C_{17}H_{22}N_6$): Calcd 310.1906, Found 310.1905; IR (ATR, ṽ) = 3111 (w), 3089 (vw), 3067 (vw), 3031 (w), 2987 (w), 2975 (w), 2935 (w), 2871 (w), 2220 (s), 1761 (vw), 1606 (vw), 1533 (m), 1494 (w), 1466 (w), 1455 (m), 1441 (w), 1419 (vs), 1391 (s), 1375 (vs), 1363 (vs), 1313 (m), 1272 (s), 1238 (vs), 1211 (vs), 1164 (s), 1132 (s), 1103 (vs), 1078 (m), 1026 (vs), 946 (w), 936 (w), 909 (m), 884 (s), 850 (w), 815 (w), 785 (m), 722 (vs), 710 (s), 691 (vs), 666 (m), 630 (m), 615 (w) cm^{-1}.

Additional information on the chemical synthesis is available *via* Chemotion repository:
https://doi.org/10.14272/reaction/SA-FUHFF-UHFFFADPSC-KRKANRSFKB-UHFFFADPSC-NUHFF-NVGOA-NUHFF-ZZZ
Additional information on the analysis of the target compound is available *via* Chemotion repository:
https://doi.org/10.14272/AONLLYWWGOIMLR-XUTLUUPISA-N.1
https://doi.org/10.14272/AQYSAXXLCHFEGV-QZQOTICOSA-N.1

(*E*)-3-(3,3-Diisopropyltriaz-1-en-1-yl)-1-(4-methylbenzyl)-1*H*-pyrazole-4-carbonitrile (12b), (*E*)-5-(3,3-diisopropyltriaz-1-en-1-yl)-1-(4-methylbenzyl)-1*H*-pyrazole-4-carbonitrile (13b)

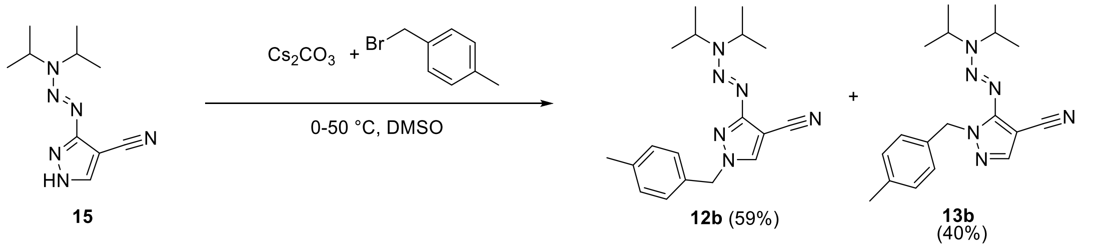

In a vial, 5-(*E*)-[di(propan-2-yl)amino]diazenyl]-1*H*-pyrazole-4-carbonitrile (**15**, 563 mg, 2.55 mmol, 1.00 equiv) was dissolved in 20 mL of DMSO. The solution was cooled to 0 °C. Cesium carbonate (1.00 g, 3.07 mmol, 1.20 equiv) and 1-(bromomethyl)-4-methylbenzene (700 mg, 3.78 mmol, 1.48 equiv) were added. The mixture was stirred first at 21 °C for 2 hours, then at 50 °C for 12 hours. The reaction was quenched with ice and extracted with EtOAc (3 × 20 mL). The solvent was removed under reduced pressure to give the crude product. The obtained crude product was purified *via* flash chromatography on silica gel using cyclohexane/ethyl acetate 10:1 to 4:1, giving 3-(3,3-diisopropyltriaz-1-en-1-yl)-1-(4-methylbenzyl)-1*H*-pyrazole-4-carbonitrile (**12b**, 485 mg, 1.50 mmol, 59% yield) as a colorless solid and 5-(3,3-diisopropyltriaz-1-en-1-yl)-1-(4-methylbenzyl)-1*H*-pyrazole-4-carbonitrile (**13b**, 329 mg, 1.01 mmol, 40% yield) as a colorless solid.

12b: (E)-3-(3,3-diisopropyltriaz-1-en-1-yl)-1-(4-methylbenzyl)-1H-pyrazole-4-carbonitrile; Formula: $C_{18}H_{24}N_6$; Exact Mass: 324.2062; Smiles: N#Cc1cn(nc1/N=N/N(C(C)C)C(C)C)Cc1ccc(cc1)C; InChIKey: GJXVYPGROXVEAZ-LSDHQDQOSA-N

R_f = 0.34 (cyclohexane/ethyl acetate 4:1). ^{1}H NMR (400 MHz, $CDCl_3$, ppm) δ = 7.58 (s, 1H), 7.21–7.10 (m, 4H), 5.39 (hept, *J* = 7.1 Hz, 1H), 5.16 (s, 2H), 4.01 (hept, *J* = 6.6 Hz, 1H), 2.33 (s, 3H), 1.43 (d, *J* = 6.7 Hz, 6H), 1.23 (d, *J* = 6.8 Hz, 6H); ^{13}C NMR (100 MHz, $CDCl_3$, ppm) δ = 162.8, 138.6, 135.5 (+, CH), 131.7, 129.8 (+, CH, 2C), 128.4 (+, CH, 2C), 115.3, 81.3, 56.6 (–, CH_2), 49.8 (+, CH), 46.4 (+, CH), 23.3 (+, CH_3, 2C), 21.2 (+, CH_3), 19.3 (+, CH_3, 2C); MS (FAB, Matrix: 3-NBA): *m/z* (%) = 326 (22) $[M+2H]^+$, 325 (100) $[M+H]^+$, 324 (20) $[M]^+$, 224 (20), 154 (10), 105 (45), 100 (11), 95 (10). HRMS–FAB *(m/z)*: $[M+H]^+$ Calcd for $C_{18}H_{25}N_6$ 325.2141; Found 325.2139; IR (ATR, ṽ) = 3132 (vw), 2978 (w), 2931 (w), 2873 (vw), 2218 (vs), 1615 (vw), 1540 (s), 1513 (w), 1468 (w), 1456 (m), 1439 (w), 1414 (vs), 1405 (vs), 1384 (s), 1370 (vs), 1353 (s), 1310 (w), 1264 (vs), 1232 (s), 1203 (w), 1157 (vs), 1132 (m), 1115 (w), 1099 (m), 1031 (m), 1021 (w), 1001 (w), 932 (vw), 912 (w), 843 (w), 820 (s), 782 (s), 752 (s), 717 (vs), 693 (m), 640 (w), 630 (m) cm^{-1}.

13b: (E)-5-(3,3-diisopropyltriaz-1-en-1-yl)-1-(4-methylbenzyl)-1H-pyrazole-4-carbonitrile; Formula: $C_{18}H_{24}N_6$; Exact Mass: 324.2062; Smiles: N#Cc1cnn(c1/N=N/N(C(C)C)C(C)C)Cc1ccc(cc1)C; InChIKey: RAZAPXAZBSDVNH-QURGRASLSA-N

R_f = 0.43 (cyclohexane/ethyl acetate 4:1). ^{1}H NMR (400 MHz, $CDCl_3$, ppm) δ = 7.64 (s, 1H), 7.22–7.07 (m, 4H), 5.32 (s, 2H), 5.19 (hept, *J* = 6.8 Hz, 1H), 4.10 (hept, *J* = 6.6 Hz, 1H), 2.31 (s, 3H), 1.45 (d, *J* = 6.6 Hz, 6H), 1.28 (d, *J* = 6.8 Hz, 6H). ^{13}C NMR (100 MHz, $CDCl_3$, ppm) δ = 154.7, 142.4 (+, CH), 137.8, 133.5, 129.4 (+, CH, 2C), 128.0 (+, CH, 2C), 116.0, 77.8, 52.0 (–, CH_2), 51.4 (+, CH), 48.1 (+, CH), 23.2 (+, CH_3, 2C), 21.2 (+, CH_3), 19.0 (+, CH_3, 2C); MS (FAB, Matrix: 3-NBA): *m/z* (%) = 326 (22) $[M+2H]^+$, 325 (100) $[M+H]^+$, 324 (29) $[M]^+$, 105 (49), 100 (11). HRMS–FAB *(m/z)*: $[M+H]^+$ Calcd for $C_{18}H_{25}N_6$ 325.2141; Found 325.2140; IR (ATR, ṽ) = 3121 (vw), 2972 (w), 2945 (w), 2873 (w), 2221 (vs), 1741 (vw), 1615 (vw), 1537 (m), 1514 (w), 1489 (w), 1466 (w), 1422 (vs), 1394 (vs), 1378 (vs), 1366 (vs), 1322 (w), 1279 (m), 1266 (s), 1238 (vs), 1208 (s), 1173 (w), 1162 (m), 1133 (m), 1102 (vs), 1026 (vs), 990 (w), 945 (w), 925 (w), 909 (w), 881 (w), 873 (m), 851 (w), 839 (w), 817 (m), 810 (w), 762 (s), 754 (vs), 728 (w), 707 (m), 659 (w), 642 (vw), 618 (m), cm^{-1}.

Additional information on the chemical synthesis is available *via* Chemotion repository:
https://doi.org/10.14272/reaction/SA-FUHFF-UHFFFADPSC-UZTCZZRTCD-UHFFFADPSC-NUHFF-NPQQK-NUHFF-ZZZ
Additional information on the analysis of the target compound is available *via* Chemotion repository:
https://doi.org/10.14272/GJXVYPGROXVEAZ-LSDHQDQOSA-N.1
https://doi.org/10.14272/RAZAPXAZBSDVNH-QURGRASLSA-N.1

(*E*)-1-(3,5-Difluorobenzyl)-3-(3,3-diisopropyltriaz-1-en-1-yl)-1*H*-pyrazole-4-carbonitrile (12c), (*E*)-1-(3,5-Difluorobenzyl)-5-(3,3-diisopropyltriaz-1-en-1-yl)-1*H*-pyrazole-4-carbonitrile (13c)

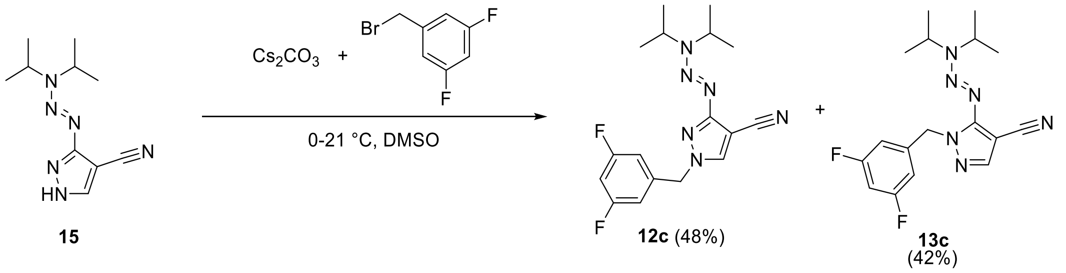

In a vial, 5-[(*E*)-(diisopropylamino)azo]-1*H*-pyrazole-4-carbonitrile (**15**, 75.0 mg, 340 µmol, 1.00 equiv) was dissolved in 5 mL of DMSO. Cesium carbonate (133 mg, 409 µmol, 1.20 equiv) was added and the solution was cooled to 0 °C. 1-(Bromomethyl)-3,5-difluorobenzene (141 mg, 88.1 µL, 681 µmol, 2.00 equiv) was added and the vial was closed and slowly warmed to 21 °C. The reaction mixture was stirred at 21 °C for 48 hours. The reaction was quenched by addition of ice and was extracted with EtOAc (3 × 15 mL). The obtained organic layers were co-evaporated with Celite(R) to give the Celite-immobilized crude product. The obtained crude product was purified *via* flash-chromatography on silica gel using cyclohexane/ethyl acetate 10:1 to 4:1, giving (*E*)-1-(3,5-difluorobenzyl)-3-(3,3-diisopropyltriaz-1-en-1-yl)-1*H*-pyrazole-4-carbonitrile (**12c**, 57.0 mg, 165 µmol, 48% yield) as a colorless solid and (*E*)-1-(3,5-difluorobenzyl)-5-(3,3-diisopropyltriaz-1-en-1-yl)-1*H*-pyrazole-4-carbonitrile (**13c**, 49.2 mg, 142 µmol, 42% yield) as a colorless solid.

12c: (E)-1-(3,5-difluorobenzyl)-3-(3,3-diisopropyltriaz-1-en-1-yl)-1H-pyrazole-4-carbonitrile; Formula: $C_{17}H_{20}F_2N_6$; Exact Mass: 346.1718; Smiles: N#Cc1cn(nc1/N=N/N(C(C)C)C(C)C)Cc1cc(F)cc(c1)F; InChIKey: WSQPCVJPBSUDNZ-XTQSDGFTSA-N

R_f = 0.21 (cyclohexane/ethyl acetate 4:1). ^{1}H NMR (400 MHz, $CDCl_3$, ppm) δ = 7.74 (s, 1H), 6.83–6.68 (m, 3H), 5.39 (hept, *J* = 6.8 Hz, 1H), 5.19 (s, 2H), 4.03 (hept, *J* = 6.5 Hz, 1H), 1.43 (d, *J* = 6.6 Hz, 6H), 1.23 (d, *J* = 6.8 Hz, 6H); ^{13}C NMR (100 MHz, $CDCl_3$, ppm) δ = 163.3 (dd, *J* = 250.3 Hz, *J* = 12.6 Hz; 2C), 163.2, 138.9 (t, *J* = 9.0 Hz), 136.2 (+, CH), 115.0, 111.7 (dd, *J* = 18.8 Hz, *J* = 7.3 Hz; +, CH, 2C), 104.1 (t, *J* = 25.2 Hz; +, CH), 82.1, 55.7 (t, *J* = 2.3 Hz; –, CH_2), 50.0 (+, CH), 46.7 (+, CH), 23.3 (+, CH_3, 2C), 19.2 (+, CH_3, 2C); ^{19}F NMR (376 MHz, $CDCl_3$, ppm) δ = -108.33 (2F); EI (m/z, 70 eV, 80 °C): 346 (9) $[M]^+$, 253 (24), 252 (16), 246 (11), 127 (100), 101 (11), 100 (12), 83 (11), 77 (10), 71 (13), 69 (24), 58 (27), 57 (29), 55 (15). HRMS ($C_{17}H_{20}N_6F_2$): Calcd 346.1718, Found 346.1716; IR (ATR, ṽ) = 3125 (vw), 3098 (vw), 3055 (vw), 2989 (w), 2976 (w), 2962 (w), 2932 (w), 2873 (w), 2221 (s), 1625 (s), 1598 (m), 1540 (s), 1460 (s), 1439 (m), 1411 (vs), 1368 (vs), 1356 (vs), 1329 (w), 1317 (vs), 1262 (vs), 1230 (s), 1188 (w), 1156 (vs), 1143 (s), 1119 (vs), 1099 (s), 1031 (s), 1014 (s), 997 (s), 976 (w), 948 (w), 909 (w), 892 (w), 873 (m), 849 (vs), 839 (vs), 796 (s), 734 (s), 718 (vs), 690 (m), 654 (s), 633 (m), 612 (w), 601 (w) cm^{-1}.

13c: (E)-1-(3,5-difluorobenzyl)-5-(3,3-diisopropyltriaz-1-en-1-yl)-1H-pyrazole-4-carbonitrile; Formula: $C_{17}H_{20}F_2N_6$; Exact Mass: 346.1718; Smiles: CC(N(C(C)C)/N=N/c1n(ncc1C#N)Cc1cc(F)cc(c1)F)C; InChIKey: ZFEWCQZPKGQXCJ-GHVJWSGMSA-N

R_f = 0.30 (cyclohexane/ethyl acetate 4:1). ^{1}H NMR (400 MHz, $CDCl_3$, ppm) δ = 7.65 (s, 1H), 6.89–6.73 (m, 2H), 6.72–6.64 (m, 1H), 5.31 (s, 2H), 5.14 (hept, *J* = 6.8 Hz, 1H), 4.10 (hept, *J* = 6.6 Hz, 1H), 1.44 (d, *J* = 6.7 Hz, 6H), 1.25 (d, *J* = 6.8 Hz, 6H); ^{13}C NMR (100 MHz, $CDCl_3$, ppm) δ = 163.1 (dd, *J* = 249.4 Hz, *J* = 12.7 Hz, 2C), 154.9, 142.6 (+, CH), 140.2 (t, *J* = 9.1 Hz), 115.6, 110.8 (dd, *J* = 18.6 Hz, *J* = 7.0 Hz, CH, 2C), 103.4 (t, *J* = 25.2 Hz; +, CH), 79.4, 51.5 (+, CH), 51.3 (t, *J* = 2.3 Hz, –, CH_2), 48.2 (+, CH), 23.1 (+, CH_3, 2C), 18.8 (+, CH_3, 2C); ^{19}F NMR (376 MHz, $CDCl_3$, ppm) δ = -109.1; EI (m/z, 70 eV, 80 °C): 347 (10) $[M+H]^+$, 346 (52) $[M]^+$, 246 (29), 181 (19), 131 (29), 127 (51), 119 (10), 100 (33), 69 (100), 58 (22). HRMS ($C_{17}H_{20}N_6F_2$): Calcd 346.1718, Found 346.1716; IR (ATR, ṽ) = 3129 (vw), 3074 (vw), 3055 (vw), 2985 (w), 2948 (w), 2878 (vw), 2218 (s), 1822 (vw), 1737 (vw), 1711 (vw), 1660 (vw), 1625 (m), 1599 (s), 1536 (m), 1493 (w), 1466 (m), 1446 (m), 1424 (vs), 1392 (w), 1366 (vs), 1349 (vs), 1315 (s), 1293 (w), 1271 (s), 1242 (s), 1221 (vs), 1205 (s), 1174 (m), 1164 (m), 1136 (m), 1122 (vs), 1101 (vs), 1043 (w), 1028 (vs), 990 (s), 935 (s), 911 (m), 884 (w), 873 (m), 858 (vs), 847 (vs), 773 (s), 734 (w), 713 (s), 697 (m), 663 (m), 632 (s), 606 (m) cm^{-1}.

Additional information on the chemical synthesis is available *via* Chemotion repository:
https://doi.org/10.14272/reaction/SA-FUHFF-UHFFFADPSC-RPEOLKMCSJ-UHFFFADPSC-NUHFF-NJTWA-NUHFF-ZZZ
Additional information on the analysis of the target compound is available *via* Chemotion repository:
https://doi.org/10.14272/WSQPCVJPBSUDNZ-XTQSDGFTSA-N.1
https://doi.org/10.14272/ZFEWCQZPKGQXCJ-GHVJWSGMSA-N.1

(*E*)-3-(3,3-Diisopropyltriaz-1-en-1-yl)-1-ethyl-1*H*-pyrazole-4-carbonitrile (12d), (*E*)-5-(3,3-diisopropyltriaz-1-en-1-yl)-1-ethyl-1*H*-pyrazole-4-carbonitrile (13d)

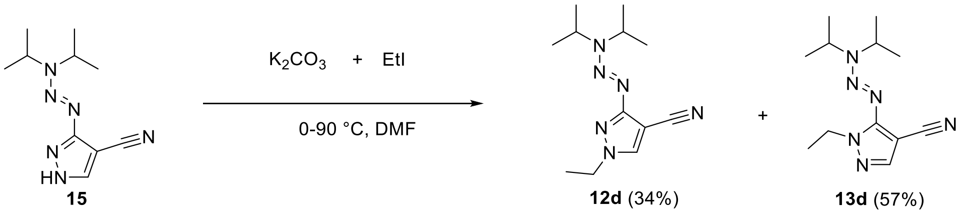

To a solution of 5-[(*E*)-[di(propan-2-yl)amino]diazenyl]-1*H*-pyrazole-4-carbonitrile (**15**, 301 mg, 1.36 mmol, 1.00 equiv) in 8 mL of anhydrous *N,N*-dimethylformamide, potassium carbonate (226 mg, 1.63 mmol, 1.20 equiv) was added at 0 °C and the resulting mixture was stirred at 0 °C for 45 minutes. Iodoethane (255 mg, 131 µL, 1.63 mmol, 1.20 equiv) was slowly added over 15 minutes and the reaction mixture was heated to 90 °C for 14 hours. The resulting mixture was cooled, poured over ice and the aqueous phase was extracted with EtOAc (3 × 15 mL). The combined organic layers were dried over Na_2SO_4, filtered and the solvent was removed under reduced pressure to give the crude product. The obtained crude product was purified *via* flash-chromatography on silica gel using cyclohexane/ethyl acetate 10:1 to 2:1, giving (*E*)-5-(3,3-diisopropyltriaz-1-en-1-yl)-1-ethyl-1*H*-pyrazole-4-carbonitrile (**12d**, 194 mg, 780 µmol, 57% yield) as a slightly grey solid and (*E*)-3-(3,3-diisopropyltriaz-1-en-1-yl)-1-ethyl-1*H*-pyrazole-4-carbonitrile (**13d**, 117 mg, 470 µmol, 34% yield) as a slightly grey solid.

12d: (E)-3-(3,3-diisopropyltriaz-1-en-1-yl)-1-ethyl-1H-pyrazole-4-carbonitrile; Formula: $C_{12}H_{20}N_6$; Exact Mass: 248.1749; Smiles: N#Cc1cn(nc1/N=N/N(C(C)C)C(C)C)CC; InChIKey: IOAQHBXBXAUXJG-JQIJEIRASA-N

R_f = 0.49 (cyclohexane/ethyl acetate 2:1). ^{1}H NMR (400 MHz, $CDCl_3$, ppm) δ = 7.66 (s, 1H), 5.38 (hept, *J* = 6.8 Hz, 1H), 4.09 (q, *J* = 7.3 Hz, 2H), 4.00 (hept, *J* = 6.7 Hz, 1H), 1.48 (t, *J* = 7.3 Hz, 3H), 1.42 (d, *J* = 6.6 Hz, 6H), 1.21 (d, *J* = 6.8 Hz, 6H); ^{13}C NMR (100 MHz, $CDCl_3$, ppm) δ = 162.8, 134.9 (+, CH), 115.4, 80.6, 49.8 (+, CH), 47.9 (–, CH_2), 46.4 (+, CH), 23.3 (+, CH_3, 2C), 19.2 (+, CH_3, 2C), 15.1 (+, CH_3); MS (FAB, Matrix: 3-NBA): *m/z* (%) = 250 (17) $[M+2H]^+$, 249 (100) $[M+H]^+$, 248 (15) $[M]^+$, 148 (53), 147 (23), 137 (12), 136 (10), 123 (11), 111 (20), 109 (22), 100 (28), 99 (10), 97 (39), 95 (32), 93 (10), 85 (25). HRMS–FAB *(m/z)*: $[M+H]^+$ Calcd for $C_{12}H_{21}N_6$, 249.1822; Found 249.1823; IR (ATR, ṽ) = 3125 (vw), 3061 (vw), 2975 (m), 2935 (w), 2873 (w), 2220 (m), 2169 (vw), 1650 (vw), 1537 (s), 1455 (m), 1412 (vs), 1377 (s), 1366 (vs), 1349 (s), 1322 (w), 1261 (vs), 1225 (vs), 1186 (w), 1153 (vs), 1132 (m), 1102 (s), 1082 (w), 1033 (s), 1013 (w), 958 (w), 908 (w), 884 (w), 839 (m), 819 (m), 802 (w), 771 (vw), 718 (m), 690 (w), 629 (m) cm^{-1}.

13d: (E)-5-(3,3-diisopropyltriaz-1-en-1-yl)-1-ethyl-1H-pyrazole-4-carbonitrile; Formula: $C_{12}H_{20}N_6$; Exact Mass: 248.1749; Smiles: N#Cc1cnn(c1/N=N/N(C(C)C)C(C)C)CC; InChIKey: ZTYVCHQAFNRURB-FOCLMDBBSA-N

R_f = 0.71 (cyclohexane/ethyl acetate 2:1). ^{1}H NMR (400 MHz, $CDCl_3$, ppm) δ = 7.61 (s, 1H), 5.18 (hept, *J* = 6.9 Hz, 1H), 4.23 (q, *J* = 7.3 Hz, 2H), 4.09 (hept, *J* = 6.6 Hz, 1H), 1.45 (d, *J* = 6.6 Hz, 6H), 1.41 (t, *J* = 7.3 Hz, 3H), 1.30 (d, *J* = 6.9 Hz, 6H); ^{13}C NMR (100 MHz, $CDCl_3$, ppm) δ = 154.2, 142.0 (+, CH), 116.1, 77.7, 51.3 (+, CH), 47.9 (+, CH), 43.7 (–, CH_2), 23.2 (+, CH_3, 2C), 19.0 (+, CH_3, 2C), 15.0 (+, CH_3); MS (FAB, Matrix: 3-NBA): *m/z* (%) = 250 (15) $[M+2H]^+$, 249 (100) $[M+H]^+$, 248 (45) $[M]^+$, 148 (13). HRMS–FAB *(m/z)*: $[M+H]^+$ Calcd for $C_{12}H_{21}N_6$ 249.1822; Found 249.1825; IR (ATR, ṽ) = 3111 (vw), 2978 (w), 2938 (w), 2874 (w), 2221 (s), 2172 (vw), 1758 (vw), 1536 (w), 1497 (w), 1470 (w), 1462 (w), 1422 (vs), 1392 (vs), 1378 (vs), 1367 (vs), 1319 (m), 1272 (s), 1251 (m), 1220 (s), 1196 (m), 1179 (m), 1162 (m), 1135 (m), 1109 (s), 1098 (s), 1086 (m), 1028 (w), 1010 (s), 958 (m), 909 (w), 882 (s), 846 (w), 793 (vw), 735 (vw), 722 (w), 711 (m), 674 (vw), 652 (w), 623 (vw), 606 (w), 577 (w), 550 (w), 531 (m), 518 (w), 431 (vw), 408 (vw), 384 (vw) cm^{-1}.

Additional information on the chemical synthesis is available *via* Chemotion repository:
https://doi.org/10.14272/reaction/SA-FUHFF-UHFFFADPSC-PTHGCHFVSV-UHFFFADPSC-NUHFF-NXOJS-NUHFF-ZZZ
Additional information on the analysis of the target compound is available *via* Chemotion repository:
https://doi.org/10.14272/IOAQHBXBXAUXJG-JQIJEIRASA-N.1
https://doi.org/10.14272/ZTYVCHQAFNRURB-FOCLMDBBSA-N.1

(*E*)-1-Cyclopentyl-3-(3,3-diisopropyltriaz-1-en-1-yl)-1*H*-pyrazole-4-carbonitrile (12e), (*E*)-1-cyclopentyl-5-(3,3-diisopropyltriaz-1-en-1-yl)-1*H*-pyrazole-4-carbonitrile (13e)

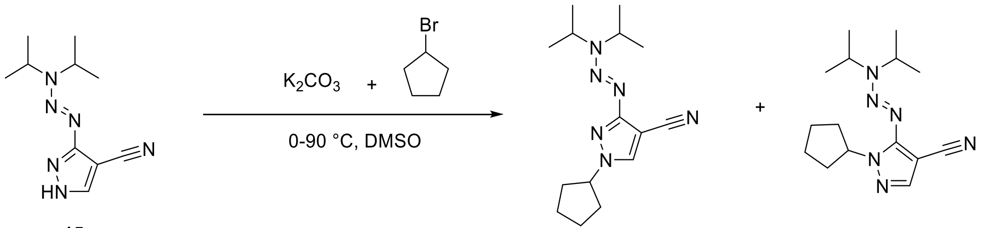

15 **12e** (39%) **13e** (52%)

To a solution of 5-[[di(propan-2-yl)amino]diazenyl]-1*H*-pyrazole-4-carbonitrile (**15**, 306 mg, 1.39 mmol, 1.00 equiv) in 15 mL of anhydrous *N*,*N*-dimethylformamide, potassium carbonate (231 mg, 1.67 mmol, 1.20 equiv) was added at 0 °C and the resulting mixture was stirred at 0 °C for 45 minutes. Bromocyclopentane (249 mg, 179 µL, 1.67 mmol, 1.20 equiv) was slowly added over 15 minutes and the reaction mixture was heated to 90 °C for 14 hours. The resulting mixture was cooled, poured over ice and the aqueous phase was extracted with EtOAc (3 × 25 mL). The combined organic layers were dried over Na_2SO_4, filtered and the solvent was removed under reduced pressure to give the crude product. The obtained crude product was purified *via* flash-chromatography on silica gel using cyclohexane/ethyl acetate 10:1 to 4:1, giving 1-cyclopentyl-3-(3,3-diisopropyltriaz-1-en-1-yl)-1*H*-pyrazole-4-carbonitrile (**12e**, 157 mg, 544 µmol, 39% yield) as a colorless solid and 1-cyclopentyl-5-(3,3-diisopropyltriaz-1-en-1-yl)-1*H*-pyrazole-4-carbonitrile (**13e**, 207 mg, 718 µmol, 52% yield) as a colorless solid.

12e: (E)-1-cyclopentyl-3-(3,3-diisopropyltriaz-1-en-1-yl)-1H-pyrazole-4-carbonitrile; Formula: $C_{15}H_{24}N_6$; Exact Mass: 288.2062; Smiles: CC(N(C(C)C)/N=N/c1nn(cc1C#N)C1CCCC1)C; InChIKey: VHDGMVPZVVGIBB-HTXNQAPBSA-N

R_f = 0.18 (cyclohexane/ethyl acetate 4:1). ^{1}H NMR (400 MHz, $CDCl_3$, ppm) δ = 7.69 (s, 1H), 5.46 (hept, *J* = 6.5 Hz, 1H), 4.54 (p, *J* = 7.1 Hz, 1H), 4.00 (hept, *J* = 6.7 Hz, 1H), 2.20–1.98 (m, 4H), 1.94–1.77 (m, 2H), 1.77–1.60 (m, 2H), 1.44 (d, *J* = 6.6 Hz, 6H), 1.22 (d, *J* = 6.9 Hz, 6H); ^{13}C NMR (100 MHz, $CDCl_3$, ppm) δ = 162.8, 134.3 (+, CH), 115.7, 80.3, 64.0 (+, CH), 49.7 (+, CH), 46.2 (+, CH), 32.7 (–, CH_2, 2C), 24.2 (–, CH_2, 2C), 23.4 (+, CH_3, 2C), 19.4 (+, CH_3, 2C); MS (FAB, Matrix: 3-NBA): *m/z* (%) = 290 (20) $[M+2H]^+$, 289 (100) $[M+H]^+$, 288 (14) $[M]^+$, 287 (12) $[M-H]^+$, 221 (12), 188 (65), 147 (32), 136 (11), 123 (10), 120 (19), 111 (14), 109 (21), 107 (10), 100 (52), 97 (28), 95 (25), 86 (10), 85 (35). HRMS–FAB *(m/z)*: $[M+H]^+$ Calcd for $C_{15}H_{25}N_6$, 289.2135; Found 289.2133; IR (ATR, ṽ) = 3128 (vw), 2975 (m), 2938 (w), 2873 (w), 2224 (s), 2169 (vw), 1541 (m), 1459 (w), 1451 (w), 1402 (vs), 1381 (vs), 1366 (vs), 1315 (w), 1258 (vs), 1227 (vs), 1187 (w), 1157 (vs), 1129 (s), 1101 (s), 1028 (m), 1000 (w), 942 (vw), 909 (w), 882 (w), 823 (m), 720 (m), 690 (vw), 637 (w) cm^{-1}.

13e: (E)-1-cyclopentyl-5-(3,3-diisopropyltriaz-1-en-1-yl)-1H-pyrazole-4-carbonitrile; Formula: $C_{15}H_{24}N_6$; Exact Mass: 288.2062; Smiles: CC(N(C(C)C)/N=N/c1n(ncc1C#N)C1CCCC1)C; InChIKey: OPJIUORDWAXUPI-VHEBQXMUSA-N

R_f = 0.33 (cyclohexane/ethyl acetate 4:1). ^{1}H NMR (400 MHz, $CDCl_3$, ppm) δ = 7.61 (s, 1H), 5.19 (hept, *J* = 6.8 Hz, 1H), 5.01 (p, *J* = 7.6 Hz, 1H), 4.08 (hept, *J* = 6.8 Hz, 1H), 2.11–1.98 (m, 4H), 1.98–1.83 (m, 2H), 1.73–1.58 (m, 2H), 1.45 (d, *J* = 6.7 Hz, 6H), 1.29 (d, *J* = 6.8 Hz, 6H); ^{13}C NMR (100 MHz, $CDCl_3$, ppm) δ = 154.4, 141.8 (+, CH), 116.3, 77.6, 58.6 (+, CH), 51.2 (+, CH), 47.8 (+, CH), 32.4 (–, CH_2, 2C), 24.9 (–, CH_2, 2C), 23.2 (+, CH_3, 2C), 19.1 (+, CH_3, 2C); MS (FAB, Matrix: 3-NBA): *m/z* (%) = 290 (20) $[M+2H]^+$, 289 (100) $[M+H]^+$, 288 (43) $[M]^+$, 188 (10), 154 (13), 136 (10), 120 (10), 100 (11). HRMS–FAB *(m/z)*: $[M+H]^+$ Calcd for $C_{15}H_{25}N_6$, 289.2135; Found 289.2133; IR (ATR, ṽ) = 3101 (vw), 2970 (w), 2935 (w), 2870 (w), 2218 (s), 2166 (vw), 1747 (vw), 1531 (m), 1480 (w), 1470 (m), 1445 (w), 1418 (s), 1394 (vs), 1378 (s), 1358 (vs), 1322 (w), 1269 (vs), 1222 (s), 1197 (m), 1171 (m), 1160 (m), 1130 (m), 1098 (s), 1031 (w), 1013 (s), 931 (w), 907 (w), 875 (m), 846 (w), 734 (vw), 711 (w), 652 (w), 608 (vw) cm^{-1}.

Additional information on the chemical synthesis is available *via* Chemotion repository:
https://doi.org/10.14272/reaction/SA-FUHFF-UHFFFADPSC-CCXXYIBWZN-UHFFFADPSC-NUHFF-NCWUQ-NUHFF-ZZZ
Additional information on the analysis of the target compound is available *via* Chemotion repository:
https://doi.org/10.14272/VHDGMVPZVVGIBB-HTXNQAPBSA-N.1
https://doi.org/10.14272/OPJIUORDWAXUPI-VHEBQXMUSA-N.1

(*E*)-3-(3,3-Diisopropyltriaz-1-en-1-yl)-1-isobutyl-1*H*-pyrazole-4-carbonitrile (12f), (*E*)-5-(3,3-diisopropyltriaz-1-en-1-yl)-1-isobutyl-1*H*-pyrazole-4-carbonitrile (13f)

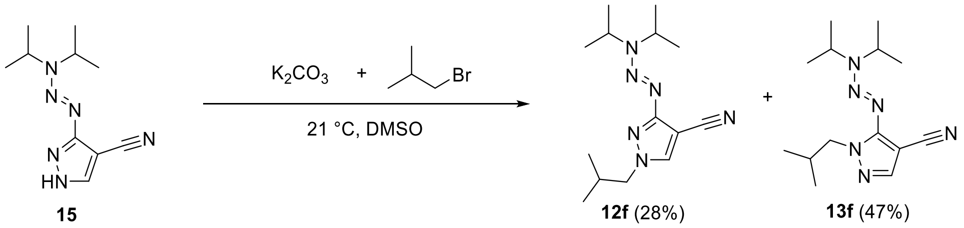

To a stirred suspension of 5-[(*E*)-[di(propan-2-yl)amino]diazenyl]-1*H*-pyrazole-4-carbonitrile (**15**, 507 mg, 2.30 mmol, 1.00 equiv) and potassium carbonate (314 mg, 2.27 mmol, 0.986 equiv) in 5 mL of *N*,*N*-dimethylformamide, 1-bromo-2-methylpropane (366 mg, 291 µL, 2.67 mmol, 1.16 equiv) was added dropwise within 10 min at 21 °C. The reaction mixture was stirred at 21 °C for 14 hours. The reaction was quenched in ice-cold water and extracted with ethyl acetate (3 × 5 mL). The combined organic layers were dried over sodium sulfate and concentrated in vacuo to give the crude product.
The obtained crude product was purified *via* flash-chromatography on silica gel using cyclohexane/ethyl acetate 20:1 to 4:1, giving 3-(3,3-diisopropyltriaz-1-en-1-yl)-1-isobutyl-1*H*-pyrazole-4-carbonitrile (**12f**, 177 mg, 640 µmol, 28% yield) as a light-yellow solid and 5-(3,3-diisopropyltriaz-1-en-1-yl)-1-isobutyl-1*H*-pyrazole-4-carbonitrile (**13f**, 299 mg, 1.08 mmol, 47% yield) as a transparent yellow solid.

12f: (E)-3-(3,3-diisopropyltriaz-1-en-1-yl)-1-isobutyl-1H-pyrazole-4-carbonitrile; Formula: $C_{14}H_{24}N_6$; Exact Mass: 276.2062; Smiles: N#Cc1cn(nc1/N=N/N(C(C)C)C(C)C)CC(C)C; InChIKey: GSWCLGSPFBAJHR-FBMGVBCBSA-N

R_f = 0.34 (cyclohexane/ethyl acetate 4:1). ^{1}H NMR (400 MHz, $CDCl_3$, ppm) δ = 7.62 (s, 1H), 5.40 (hept, *J* = 6.6 Hz, 1H), 4.01 (hept, *J* = 6.8 Hz, 1H), 3.81 (d, *J* = 7.3 Hz, 2H), 2.27 (hept, *J* = 6.9 Hz, 1H), 1.43 (d, *J* = 6.6 Hz, 6H), 1.22 (d, *J* = 6.8 Hz, 6H), 0.91 (d, *J* = 6.7 Hz, 6H); ^{13}C NMR (100 MHz, $CDCl_3$, ppm) δ = 162.9, 136.0 (+, CH), 115.5, 80.4, 60.6 (–, CH_2), 49.8 (+, CH), 46.4 (+, CH), 29.1 (+, CH), 23.3 (+, CH_3, 2C), 19.9 (+, CH_3, 2C), 19.3 (+, CH_3, 2C); MS (FAB, Matrix: 3-NBA): *m/z* (%) = 278 (18) $[M+2H]^+$, 277 (100) $[M+H]^+$, 276 (17) $[M]^+$, 176 (50), 109 (15), 107 (11), 100 (31), 97 (15), 95 (25), 93 (16), 91 (14). HRMS–FAB *(m/z)*: $[M+H]^+$ Calcd for $C_{14}H_{25}N_6$ 277.2141; Found 277.2139; IR (ATR, ṽ) = 3123 (vw), 3064 (vw), 2972 (w), 2956 (w), 2934 (w), 2871 (w), 2225 (s), 1544 (s), 1460 (m), 1445 (w), 1405 (vs), 1387 (vs), 1370 (vs), 1357 (vs), 1296 (w), 1258 (vs), 1227 (s), 1215 (m), 1157 (vs), 1130 (m), 1115 (w), 1101 (s), 1031 (m), 1009 (m), 948 (w), 926 (w), 909 (w), 894 (w), 881 (w), 858 (m), 844 (w), 819 (w), 796 (w), 724 (m), 711 (w), 630 (m) cm^{-1}.

13f: (E)-5-(3,3-diisopropyltriaz-1-en-1-yl)-1-isobutyl-1H-pyrazole-4-carbonitrile; Formula: $C_{14}H_{24}N_6$; Exact Mass: 276.2062; Smiles: N#Cc1cnn(c1/N=N/N(C(C)C)C(C)C)CC(C)C; InChIKey: MWOHIDYQOPZPNH-ISLYRVAYSA-N

R_f = 0.62 (cyclohexane/ethyl acetate 4:1). ^{1}H NMR (400 MHz, $CDCl_3$, ppm) δ = 7.62 (s, 1H), 5.12 (hept, *J* = 6.8 Hz, 1H), 4.09 (hept, *J* = 6.6 Hz, 1H), 3.98 (d, *J* = 7.2 Hz, 2H), 2.22 (hept, *J* = 6.8 Hz, 1H), 1.45 (d, *J* = 6.6 Hz, 6H), 1.29 (d, *J* = 6.8 Hz, 6H), 0.90 (d, *J* = 6.7 Hz, 6H); ^{13}C NMR (100 MHz, $CDCl_3$, ppm) δ = 154.9, 141.9 (+, CH), 116.1, 77.4, 55.6 (–, CH_2), 51.4 (+, CH), 48.1 (+, CH), 29.3 (+, CH), 23.2 (+, CH_3, 2C), 20.1 (+, CH_3, 2C), 19.0 (+, CH_3, 2C); MS (FAB, Matrix: 3-NBA): *m/z* (%) = 278 (17) $[M+2H]^+$, 277 (100) $[M+H]^+$, 276 (29) $[M]^+$, 176 (16), 100 (12), 95 (12). HRMS–FAB *(m/z)*: $[M+H]^+$ Calcd for $C_{14}H_{25}$N6, 277.2141; Found 277.2142; IR (ATR, ṽ) = 3112 (vw), 2968 (m), 2955 (m), 2946 (m), 2925 (m), 2868 (w), 2853 (w), 2217 (s), 2163 (vw), 1738 (vw), 1690 (vw), 1533 (s), 1490 (w), 1468 (m), 1446 (w), 1435 (m), 1417 (vs), 1397 (vs), 1383 (vs), 1357 (vs), 1322 (m), 1271 (vs), 1241 (vs), 1220 (vs), 1173 (s), 1162 (s), 1130 (s), 1111 (s), 1098 (s), 1034 (m), 1014 (vs), 946 (m), 926 (w), 908 (m), 892 (w), 882 (w), 873 (m), 847 (m), 823 (w), 775 (m), 727 (w), 708 (m), 654 (w), 628 (w), 620 (w) cm^{-1}.

Additional information on the chemical synthesis is available *via* Chemotion repository:
https://doi.org/10.14272/reaction/SA-FUHFF-UHFFFADPSC-NZDLOEMQHU-UHFFFADPSC-NUHFF-NXEFH-NUHFF-ZZZ
Additional information on the analysis of the target compound is available *via* Chemotion repository:
https://doi.org/10.14272/GSWCLGSPFBAJHR-FBMGVBCBSA-N.1
https://doi.org/10.14272/MWOHIDYQOPZPNH-ISLYRVAYSA-N.1

Ethyl (*E*)-2-(4-cyano-3-(3,3-diisopropyltriaz-1-en-1-yl)-1*H*-pyrazol-1-yl)acetate (12g), ethyl (*E*)-2-(4-cyano-5-(3,3-diisopropyltriaz-1-en-1-yl)-1*H*-pyrazol-1-yl)acetate (13g)

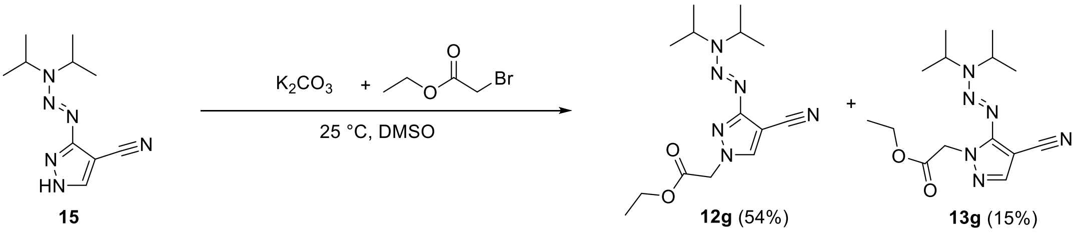

To a stirred suspension of 5-[(*E*)-[di(propan-2-yl)amino]diazenyl]-1*H*-pyrazole-4-carbonitrile (**15**, 529 mg, 2.40 mmol, 1.00 equiv) and potassium carbonate (314 mg, 2.27 mmol, 0.946 equiv) in 10 mL of *N*,*N*-dimethylformamide, ethyl 2-bromoacetate (447 mg, 297 µL, 2.67 mmol, 1.11 equiv) was added dropwise within 10 min at 21 °C. The reaction mixture was stirred at 21 °C for 14 hours. The reaction was quenched in ice-cold water and extracted with ethyl acetate (3 × 10 mL). The combined organic layers were dried over sodium sulfate and concentrated under vacuo to give the crude product. The obtained crude product was purified *via* flash-chromatography on silica gel using cyclohexane/ethyl acetate 10:0 to 4:1, giving ethyl 2-(4-cyano-3-(3,3-diisopropyltriaz-1-en-1-yl)-1*H*-pyrazol-1-yl)acetate (**12g**, 397 mg, 1.30 mmol, 54% yield) as a colorless solid and ethyl 2-(4-cyano-5-(3,3-diisopropyltriaz-1-en-1-yl)-1*H*-pyrazol-1-yl)acetate (**13g**, 109 mg, 356 µmol, 15% yield) as a colorless solid.

12g: ethyl (E)-2-(4-cyano-3-(3,3-diisopropyltriaz-1-en-1-yl)-1H-pyrazol-1-yl)acetate; Formula: $C_{14}H_{22}N_6O_2$; Exact Mass: 306.1804; Smiles: CCOC(=O)Cn1nc(c(c1)C#N)/N=N/N(C(C)C)C(C)C; InChIKey: GMSYXQOQYOUAHU-FBMGVBCBSA-N

R_f = 0.16 (cyclohexane/ethyl acetate 4:1). ^{1}H NMR (400 MHz, $CDCl_3$, ppm) δ = 7.80 (s, 1H), 5.37 (hept, *J* = 6.8 Hz, 1H), 4.82 (s, 2H), 4.24 (q, *J* = 7.2 Hz, 2H), 4.03 (hept, *J* = 6.6 Hz, 1H), 1.44 (d, *J* = 6.6 Hz, 6H), 1.28 (t, *J* = 7.1 Hz, 3H), 1.23 (d, *J* = 6.9 Hz, 6H). ^{13}C NMR (100 MHz, $CDCl_3$, ppm) δ = 166.8, 163.0, 137.5 (+, CH), 115.0, 82.5, 62.4 (–, CH_2), 53.6 (–, CH_2), 50.0 (+, CH), 46.7 (+, CH), 23.3 (+, CH_3, 2C), 19.3 (+, CH_3, 2C), 14.2 (+, CH_3); MS (FAB, Matrix: 3-NBA): *m/z* (%) = 308 (17) $[M+2H]^+$, 307 (100) $[M+H]^+$, 306 (10) $[M]^+$, 305 (11), 207 (13), 206 (76), 154 (14), 147 (12), 136 (20), 135 (11), 133 (10), 131 (11), 123 (19), 121 (33), 119 (16), 117 (11), 111 (23), 109 (38), 107 (26), 106 (17), 105 (24), 100 (42), 97 (43), 95 (70), 94 (10), 93 (34), 91 (40), 86 (10). HRMS–FAB *(m/z)*: $[M+H]^+$ Calcd for $C_{14}H_{23}O_2N_6$, 307.1882; Found 307.1882; IR (ATR, ṽ) = 3132 (vw), 3071 (vw), 2976 (w), 2936 (w), 2874 (vw), 2227 (m), 1748 (vs), 1544 (s), 1465 (m), 1409 (vs), 1368 (vs), 1356 (vs), 1299 (w), 1261 (vs), 1208 (vs), 1157 (vs), 1130 (s), 1099 (s), 1031 (s), 1006 (s), 970 (w), 909 (m), 874 (m), 843 (m), 805 (w), 718 (s), 697 (m), 639 (m), 620 (w) cm^{-1}.

13g: ethyl (E)-2-(4-cyano-5-(3,3-diisopropyltriaz-1-en-1-yl)-1H-pyrazol-1-yl)acetate; Formula: $C_{14}H_{22}N_6O_2$; Exact Mass: 306.1804; Smiles: CCOC(=O)Cn1ncc(c1/N=N/N(C(C)C)C(C)C)C#N; InChIKey: UVXRQPKXJULFTR-ISLYRVAYSA-N

R_f = 0.26 (cyclohexane/ethyl acetate 4:1). ^{1}H NMR (400 MHz, $CDCl_3$, ppm) δ = 7.67 (s, 1H), 5.10 (hept, *J* = 6.8 Hz, 1H), 4.94 (s, 2H), 4.21 (q, *J* = 7.1 Hz, 2H), 4.10 (hept, *J* = 6.6 Hz, 1H), 1.45 (d, *J* = 6.6 Hz, 6H), 1.30–1.21 (m, 9H); ^{13}C NMR (100 MHz, $CDCl_3$, ppm) δ = 167.5, 155.4, 142.6 (+, CH), 115.6, 78.1, 61.9 (–, CH_2), 51.7 (+, CH), 50.0 (–, CH_2), 48.3 (+, CH), 23.2 (+, CH_3, 2C), 18.9 (+, CH_3, 2C), 14.3 (+, CH_3); MS (FAB, Matrix: 3-NBA): *m/z* (%) = 308 (18) $[M+2H]^+$, 307 (100) $[M+H]^+$, 306 (23) $[M]^+$, 206 (10). HRMS–FAB *(m/z)*: $[M+H]^+$ Calcd for $C_{14}H_{23}O_2N_6$, 307.1882; Found 307.1881; IR (ATR, ṽ) = 2982 (w), 2941 (w), 2878 (vw), 2220 (s), 1735 (vs), 1538 (m), 1502 (w), 1473 (w), 1465 (w), 1426 (vs), 1418 (vs), 1391 (vs), 1375 (s), 1363 (vs), 1341 (s), 1313 (w), 1293 (m), 1278 (vs), 1255 (vs), 1242 (vs), 1211 (vs), 1191 (m), 1177 (w), 1163 (s), 1142 (m), 1135 (m), 1102 (vs), 1051 (m), 1030 (vs), 1021 (vs), 945 (w), 911 (m), 884 (w), 867 (w), 858 (m), 850 (m), 805 (w), 779 (m), 730 (w), 711 (m), 646 (m), 626 (w) cm^{-1}.

Additional information on the chemical synthesis is available *via* Chemotion repository:
https://doi.org/10.14272/reaction/SA-FUHFF-UHFFFADPSC-HLSFJQKZZG-UHFFFADPSC-NUHFF-NQFOL-NUHFF-ZZZ
Additional information on the analysis of the target compound is available *via* Chemotion repository:
https://doi.org/10.14272/GMSYXQOQYOUAHU-FBMGVBCBSA-N.1
https://doi.org/10.14272/UVXRQPKXJULFTR-ISLYRVAYSA-N.1

(*E*)-1-(4-Bromobenzyl)-3-(3,3-diisopropyltriaz-1-en-1-yl)-1*H*-pyrazole-4-carbonitrile (12h), (*E*)-1-(4-bromobenzyl)-5-(3,3-diisopropyltriaz-1-en-1-yl)-1*H*-pyrazole-4-carbonitrile (13h)

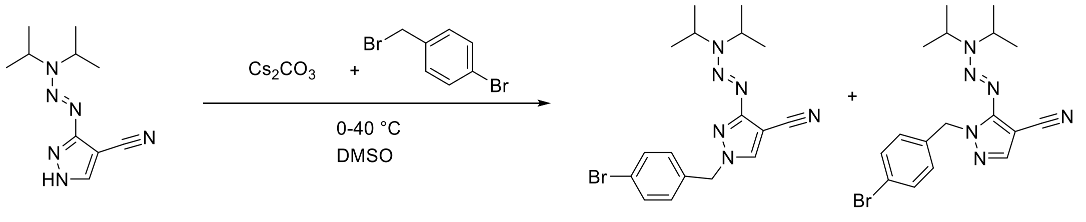

15 **12h** (51%) **13h** (42%)

In a vial, 5-(*E*)-[di(propan-2-yl)amino]diazenyl]-1*H*-pyrazole-4-carbonitrile (**15**, 307 mg, 1.39 mmol, 1.00 equiv) was dissolved in 12 mL of DMSO. Cesium carbonate (532 mg, 1.63 mmol, 1.17 equiv) was added and the solution was cooled to 0 °C. 1-Bromo-4-(bromomethyl)benzene (681 mg, 2.72 mmol, 1.96 equiv) was added and the vial was closed and slowly warmed to 21 °C. The reaction mixture was stirred at 40 °C for 2 days. The reaction was quenched by addition of ice and was extracted with EtOAc (3 × 15 mL). The obtained organic layers were co-evaporated with Celite(R) to give the Celite-immobilized crude product. The obtained crude product was purified *via* flash-chromatography on silica gel using cyclohexane/ethyl acetate 10:1 to 41:, giving (*E*)-1-(4-bromobenzyl)-3-(3,3-diisopropyltriaz-1-en-1-yl)-1*H*-pyrazole-4-carbonitrile (**12h**, 278 mg, 713 µmol) 51% yield and (*E*)-1-(4-bromobenzyl)-5-(3,3-diisopropyltriaz-1-en-1-yl)-1*H*-pyrazole-4-carbonitrile (**13h**, 227 mg, 582 µmol) in 42% yield.

12h: (E)-1-(4-bromobenzyl)-3-(3,3-diisopropyltriaz-1-en-1-yl)-1H-pyrazole-4-carbonitrile; Formula: $C_{17}H_{21}BrN_6$; Exact Mass: 388.1011; Smiles: N#Cc1cn(nc1/N=N/N(C(C)C)C(C)C)Cc1ccc(cc1)Br; InChIKey: JVCZUZVUVDDSML-LSDHQDQOSA-N

R_f = 0.2 (cyclohexane/ethyl acetate 4:1). ^{1}H NMR (400 MHz, $CDCl_3$, ppm) δ = 7.67 (s, 1H), 7.48–7.38 (m, 2H), 7.17–7.09 (m, 2H), 5.42–5.28 (m, 1H), 5.14 (d, *J* = 3.1 Hz, 2H), 4.06–3.93 (m, 1H), 1.41 (dd, *J* = 6.7 Hz, *J* = 3.0 Hz, 6H), 1.20 (dd, *J* = 7.0 Hz, J = 3.5 Hz, 6H); ^{13}C NMR (100 MHz, $CDCl_3$, ppm) δ = 163.0, 135.8 (+, CH), 134.0, 132.1 (+, CH, 2C), 129.8 (+, CH, 2C), 122.7, 115.1, 81.6, 56.0 (–, CH2), 49.8 (+, CH), 46.5 (+, CH), 23.2 (+, CH3, 2C), 19.2 (+, CH3, 2C); MS (EI, 70 eV, 90 °C): m/z (%) = 490/388 (20/22) [M]+, 297/295 (21/18), 290/288 (11/11), 208 (11), 181 (24), 171/169 (92/100), 149 (11), 131 (24), 116 (19), 100 (58), 97 (15), 91/89 (15/27), 90 (29), 85 (14), 84 (15), 83 (15), 71 (21), 70 (11), 69 (53), 58 (51), 57 (33), 55 (16). HRMS (EI, $C_{17}H_{21}N_6{}^{79}Br$): Calcd 388.1011; Found 388.1010.

13h: (E)-1-(4-bromobenzyl)-5-(3,3-diisopropyltriaz-1-en-1-yl)-1H-pyrazole-4-carbonitrile; Formula: $C_{17}H_{21}BrN_6$; Exact Mass: 388.1011; Smiles: N#Cc1cnn(c1/N=N/N(C(C)C)C(C)C)Cc1ccc(cc1)Br; InChIKey: HUNPOMLAEFCWSA-QURGRASLSA-N

R_f = 0.31 (cyclohexane/ethyl acetate 4:1). ^{1}H NMR (400 MHz, $CDCl_3$, ppm) δ = 7.64 (s, 1H), 7.46–7.37 (m, 2H), 7.16–7.08 (m, 2H), 5.30 (s, 2H), 5.14 (sept, *J* = 6.8 Hz, 1H), 4.09 (sept, *J* = 6.6 Hz, 1H), 1.44 (d, *J* = 6.6 Hz, 6H), 1.26 (d, *J* = 6.8 Hz, 6H); ^{13}C NMR (100 MHz, $CDCl_3$, ppm) δ = 154.7, 142.4 (+, CH), 135.4, 131.8 (+, CH, 2C), 129.6 (+, CH, 2C), 121.9, 115.7, 77.9, 51.5 (–, CH2), 51.4 (+, CH), 48.2 (+, CH), 23.1 (+, CH3, 2C), 18.9 (+, CH3, 2C); MS (EI, 70 eV, 80 °C): m/z (%) = 390/388 (32/31) [M]+, 338 (12), 290/288 (11/11), 231/229 (23/22), 187/185 (53/58), 181 (20), 172/170 (11/12), 171/169 (95/100), 131 (26), 100 (74), 91/89 (24/31), 90 (44), 84 (14), 78 (17), 77 (32), 71 (11), 69 (52), 58 (67), 57 (18), 55 (12). HRMS (EI, $C_{17}H_{21}N_6{}^{79}Br$): Calcd 388.1011; Found 388.1010.

Additional information on the chemical synthesis is available *via* Chemotion repository:
https://doi.org/10.14272/reaction/SA-FUHFF-UHFFFADPSC-CYZFVCDWYP-UHFFFADPSC-NUHFF-NPQQK-NUHFF-ZZZ
Additional information on the analysis of the target compound is available *via* Chemotion repository:
https://doi.org/10.14272/JVCZUZVUVDDSML-LSDHQDQOSA-N.1
https://doi.org/10.14272/HUNPOMLAEFCWSA-QURGRASLSA-N.1

(*E*)-*N*-((1-Benzyl-3-(3,3-diisopropyltriaz-1-en-1-yl)-1*H*-pyrazol-4-yl)methyl)acetamide (9a)

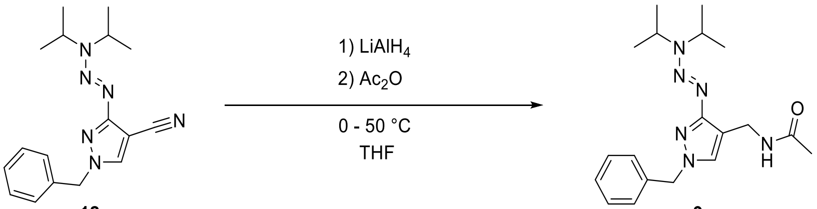

12a **9a** (23%)

Step 1: (*E*)-1-Benzyl-3-(3,3-diisopropyltriaz-1-en-1-yl)-1*H*-pyrazole-4-carbonitrile (**12a**, 120 mg, 388 µmol, 1.00 equiv) was dissolved in 65 mL of dry THF under nitrogen atmosphere. The solution was cooled to 0 °C before lithium aluminum hydride (44.1 mg, 1.16 mL, 1.16 mmol, 3.00 equiv) was slowly added. The cooling was removed and the solution was stirred at 21 °C for 14 hours, then additional 5 hours at 50 °C. The solution was cooled to 21 °C and the reaction was quenched with a saturated K/Na-tartrate solution (70 mL). The organic solvent was removed under reduced pressure and the remaining aqueous phase was extracted with methylene chloride (3 × 70 mL). The combined organic layers were washed with brine (200 mL) and dried over Na_2SO_4. The solvent was removed under reduced pressure to give the crude (*E*)-(1-benzyl-3-(3,3-diisopropyltriaz-1-en-1-yl)-1*H*-pyrazol-4-yl)methanamine, which was used for the next step without further purification.
Step 2: The crude (*E*)-(1-benzyl-3-(3,3-diisopropyltriaz-1-en-1-yl)-1*H*-pyrazol-4-yl)methanamine was dissolved in 65 mL of dry THF under nitrogen atmosphere. The solution was cooled to 0 °C before acetic anhydride (59.3 mg, 55.0 µL, 581 µmol, 1.50 equiv) was added dropwise. The cooling was removed and the solution was stirred for 14 hours at 21 °C. The reaction was quenched with a saturated K_2CO_3 solution (70 mL) and the organic solvent was removed under reduced pressure. The remaining aqueous phase was extracted with methylene chloride (3 × 70 mL). The combined organic layers were washed with brine (200 mL) and dried over Na_2SO_4. The solvent was removed under reduced pressure to give the crude product. The obtained crude product was purified *via* flash-chromatography on silica gel using methylene chloride/methanol 50:1 to 30:1. It was further purified *via* HPLC using MeCN/H_2O 10:1, to give (*E*)-*N*-((1-benzyl-3-(3,3-diisopropyltriaz-1-en-1-yl)-1*H*-pyrazol-4-yl)methyl)acetamide (**9a**, 32.0 mg, 89.8 µmol, 23% yield) as a brown oil.

9a: (E)-N-((1-benzyl-3-(3,3-diisopropyltriaz-1-en-1-yl)-1H-pyrazol-4-yl)methyl)acetamide; Formula: $C_{19}H_{28}N_6O$; Exact Mass: 356.2325; Smiles: CC(=O)NCc1cn(nc1/N=N/N(C(C)C)C(C)C)Cc1ccccc1; InChIKey: VXSAZKPHEZACHZ-XTQSDGFTSA-N

R_f = 0.06 (cyclohexane/ethyl acetate 2:1). ^{1}H NMR (400 MHz, $CDCl_3$, ppm) δ = 7.40–7.31 (m, 5H), 7.20 (s, 1H), 5.91 (br.t, *J* = 5.5 Hz, 1H), 5.41 (br.s, 1H), 5.16 (s, 2H), 4.29 (d, *J* = 5.3 Hz, 2H), 3.98 (br.s, 1H), 1.88 (s, 3H), 1.32 (br.s, 6H), 1.20 (br.s, 6H); ^{13}C NMR (100 MHz, $CDCl_3$, ppm) δ = 169.3, 158.0, 136.4, 130.2 (+, CH), 128.8 (+, CH, 2C), 128.1 (+, CH, 3C), 107.5, 56.2 (–, CH_2), 48.3 (+, CH), 45.3 (+, CH), 34.4 (–, CH_2), 23.7 (+, CH_3, 2C), 23.5 (+, CH_3), 19.4 (+, CH_3, 2C); MS (FAB, Matrix: 3-NBA): *m/z* (%) = 358 (22) $[M+2H]^+$, 357 (100) $[M+H]^+$, 356 (21) $[M]^+$, 355 (13) $[M-H]^+$, 298 (31), 257 (12), 256 (79), 100 (13), 91 (60). HRMS–FAB *(m/z)*: $[M+H]^+$ Calcd for $C_{19}H_{29}ON_6$, 357.2403; Found 357.2402; IR (ATR, ṽ) = 3285 (w), 3080 (vw), 3065 (vw), 3033 (vw), 2973 (w), 2931 (w), 2871 (vw), 2230 (vw), 1650 (s), 1543 (m), 1497 (w), 1465 (m), 1455 (s), 1419 (vs), 1402 (vs), 1364 (vs), 1341 (s), 1244 (vs), 1224 (vs), 1150 (vs), 1128 (s), 1096 (m), 1033 (s), 1011 (m), 969 (w), 909 (m), 851 (w), 841 (w), 813 (w), 727 (vs), 704 (vs), 643 (m), 630 (w) cm^{-1}.

Additional information on the chemical synthesis is available *via* Chemotion repository:
https://doi.org/10.14272/reaction/SA-FUHFF-UHFFFADPSC-VXSAZKPHEZ-UHFFFADPSC-NUHFF-NQYKM-NUHFF-ZZZ
Additional information on the analysis of the target compound is available *via* Chemotion repository:
https://doi.org/10.14272/VXSAZKPHEZACHZ-XTQSDGFTSA-N.1

(*E*)-*N*-((1-Benzyl-3-(3,3-diisopropyltriaz-1-en-1-yl)-1*H*-pyrazol-4-yl)methyl)benzamide (9b)

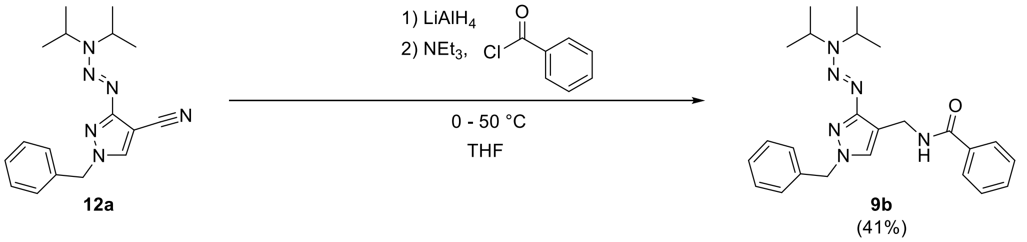

Step 1: 1-Benzyl-3-(3,3-diisopropyltriaz-1-en-1-yl)-1*H*-pyrazole-4-carbonitrile (**12a**, 202 mg, 650 µmol, 1.00 equiv) was dissolved in 100 mL of dry THF under nitrogen atmosphere. The solution was cooled to 0 °C before lithium aluminum hydride (74.0 mg, 1.95 mL, 1.95 mmol, 3.00 equiv) was slowly added. The cooling was removed and the solution was stirred at 21 °C for 14 hours, then additional 5 hours at 50 °C. The solution was cooled to 21 °C and the reaction was quenched with a saturated K/Na-tartrate solution (100 mL). The organic solvent was removed under reduced pressure and the remaining aqueous phase was extracted with methylene chloride (3 x 100 mL). The combined organic layers were washed with brine (300 mL) and dried over Na_2SO_4. The solvent was removed under reduced pressure to give the crude (1-benzyl-3-(3,3-diisopropyltriaz-1-en-1-yl)-1*H*-pyrazol-4-yl)methanamine, which was used for the next step without further purification.

Step 2: The crude (1-benzyl-3-(3,3-diisopropyltriaz-1-en-1-yl)-1*H*-pyrazol-4-yl)methanamine was dissolved in 100 mL of dry THF under nitrogen atmosphere. Triethylamine (197 mg, 272 µL, 1.95 mmol, 3.00 equiv) was added and the solution was cooled to 0 °C before benzoyl chloride (137 mg, 113 µL, 975 µmol, 1.50 equiv) was slowly added. The cooling was removed and the solution was stirred for 14 hours at 21 °C. The reaction was quenched with a saturated K_2CO_3 solution (100 mL) and the organic solvent was removed under reduced pressure. The remaining aqueous phase was extracted with methylene chloride (3 × 100 mL). The combined organic layers were washed with brine (300 mL) and dried over Na_2SO_4. The solvent was removed under reduced pressure to give the crude product. The obtained crude product was purified *via* flash-chromatography on silica gel using cyclohexane/ethyl acetate 10:1 to 4:1, giving *N*-((1-benzyl-3-(3,3-diisopropyltriaz-1-en-1-yl)-1*H*-pyrazol-4-yl)methyl)benzamide (**9b**, 111 mg, 266 µmol, 41% yield) as a brown oil.

9b: (E)-N-((1-benzyl-3-(3,3-diisopropyltriaz-1-en-1-yl)-1H-pyrazol-4-yl)methyl)benzamide; Formula: $C_{24}H_{30}N_6O$; Exact Mass: 418.2481; Smiles: CC(N(C(C)C)/N=N/c1nn(cc1CNC(=O)c1ccccc1)Cc1ccccc1)C; InChIKey: CYCAXLGFKDSWNS-BYCLXTJYSA-N

R_f = 0.38 (cyclohexane/ethyl acetate 1:1). ^{1}H NMR (400 MHz, $CDCl_3$, ppm) δ = 7.73–7.66 (m, 2H), 7.49–7.27 (m, 9H), 6.50 (br.t, *J* = 5.5 Hz, 1H), 5.42 (br.s, 1H), 5.21 (s, 2H), 4.53 (d, *J* = 5.3 Hz, 2H), 3.97 (br.s, 1H), 1.33–1.17 (m, 12H); ^{13}C NMR (100 MHz, $CDCl_3$, ppm) δ = 167.2, 158.1, 136.4, 135.0, 131.4 (+, CH, 2C), 130.5 (+, CH), 128.9 (+, CH, 2C), 128.6 (+, CH, 2C), 128.2 (+, CH, 2C), 127.0 (+, CH, 2C), 107.5, 56.3 (–, CH_2), 48.6 (+, CH), 45.5 (+, CH), 35.0 (–, CH_2), 23.7 (+, CH_3, 2C), 19.5 (+, CH_3, 2C); MS (FAB, Matrix: 3-NBA): *m/z* (%) = 419 (27) $[M+H]^+$, 318 (36) $[M]^+$, 298 (22), 154 (12), 136 (12), 109 (11), 107 (14), 105 (75), 102 (16), 100 (10), 97 (12), 95 (23), 93 (16), 91 (100). HRMS–FAB *(m/z)*: $[M+H]^+$ Calcd for $C_{24}H_{31}ON_6$ 419.2559; Found 419.2559; IR (ATR, ṽ) = 3312 (w), 3064 (w), 3031 (vw), 2972 (w), 2928 (w), 2868 (w), 1730 (vw), 1642 (s), 1602 (w), 1578 (w), 1530 (s), 1487 (m), 1465 (m), 1455 (s), 1417 (vs), 1404 (vs), 1380 (m), 1363 (s), 1346 (s), 1293 (m), 1244 (vs), 1224 (vs), 1150 (vs), 1128 (s), 1098 (m), 1075 (w), 1031 (s), 1010 (w), 1001 (w), 986 (w), 925 (w), 911 (w), 890 (vw), 850 (vw), 802 (w), 755 (w), 694 (vs), 647 (m), 605 (w) cm^{-1}.

Additional information on the chemical synthesis is available *via* Chemotion repository:
https://doi.org/10.14272/reaction/SA-FUHFF-UHFFFADPSC-CYCAXLGFKD-UHFFFADPSC-NUHFF-NULGB-NUHFF-ZZZ
Additional information on the analysis of the target compound is available *via* Chemotion repository:
https://doi.org/10.14272/CYCAXLGFKDSWNS-BYCLXTJYSA-N.1

(*E*)-*N*-((3-(3,3-Diisopropyltriaz-1-en-1-yl)-1-(4-methylbenzyl)-1*H*-pyrazol-4-yl)methyl)-3-methylbutanamide (9c)

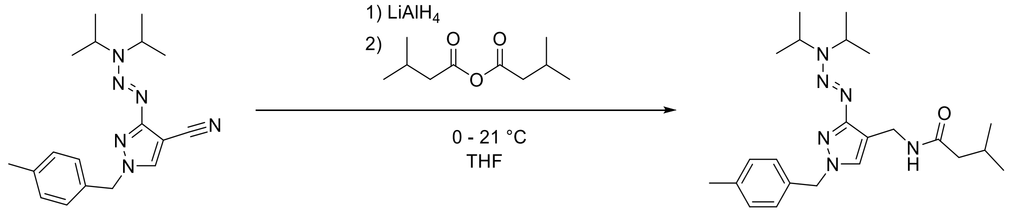

12b **9c** (61%)

Step 1: (*E*)-3-(3,3-diisopropyltriaz-1-en-1-yl)-1-(4-methylbenzyl)-1*H*-pyrazole-4-carbonitrile (**12b**, 293 mg, 903 µmol, 1.00 equiv) was dissolved in 150 mL of dry THF under nitrogen atmosphere. The solution was cooled to 0 °C before lithium aluminum hydride (103 mg, 2.71 mL, 2.71 mmol, 3.00 equiv) was slowly added. The cooling was removed and the solution was stirred at 21 °C for 14 hours, then additional 5 hours at 50 °C. The solution was cooled to 21 °C and the reaction was quenched with a saturated K/Na-tartrate solution (150 mL). The organic solvent was removed under reduced pressure and the remaining aqueous phase was extracted with methylene chloride (3 x 150 mL). The combined organic layers were washed with brine (400 mL) and dried over Na_2SO_4. The solvent was removed under reduced pressure to give the crude (*E*)-(3-(3,3-diisopropyltriaz-1-en-1-yl)-1-(4-methylbenzyl)-1*H*-pyrazol-4-yl)methanamine, which was used for the next step without further purification.
Step 2: The crude (*E*)-(3-(3,3-diisopropyltriaz-1-en-1-yl)-1-(4-methylbenzyl)-1*H*-pyrazol-4-yl)methanamine was dissolved in 150 mL of dry THF under nitrogen atmosphere. The solution was cooled to 0 °C before 3-methylbutanoyl 3-methylbutanoate (252 mg, 271 µL, 1.35 mmol, 1.50 equiv) was slowly added. The cooling was removed and the solution was stirred for 14 hours at 21 °C. The reaction was quenched with a saturated K_2CO_3 solution (150 mL) and the organic solvent was removed under reduced pressure. The remaining aqueous phase was extracted with methylene chloride (3 × 150 mL). The combined organic layers were washed with brine (400 mL) and dried over Na_2SO_4. The solvent was removed under reduced pressure to give the crude product. The obtained crude product was purified *via* flash-chromatography on silica gel using cyclohexane/ethyl acetate 4:1 to 1:1, giving (*E*)-*N*-((3-(3,3-diisopropyltriaz-1-en-1-yl)-1-(4-methylbenzyl)-1*H*-pyrazol-4-yl)methyl)-3-methylbutanamide (**9c**, 227 mg, 551 µmol, 61% yield) as a light-brown oil.

9c: (E)-N-((3-(3,3-diisopropyltriaz-1-en-1-yl)-1-(4-methylbenzyl)-1H-pyrazol-4-yl)methyl)-3-methylbutanamide; Formula: $C_{23}H_{36}N_6O$; Exact Mass: 412.2951; Smiles: CC(CC(=O)NCc1cn(nc1/N=N/N(C(C)C)C(C)C)Cc1ccc(cc1)C)C; InChIKey: MORMFGNFEXIKDM-IMVLJIQESA-N

R_f = 0.05 (cyclohexane/ethyl acetate 4:1). ^{1}H NMR (400 MHz, $CDCl_3$, ppm) δ = 7.21–7.08 (m, 5H), 5.82 (br.t, *J* = 5.5 Hz, 1H), 5.42 (br.s, 1H), 5.14 (s, 2H), 4.31 (d, *J* = 5.5 Hz, 2H), 4.00 (br.s, 1H), 2.32 (s, 3H), 2.13–1.99 (m, 1H), 1.94 (d, *J* = 7.1 Hz, 2H), 1.34 (br.s, 6H), 1.22 (br.s, 6H), 0.88 (d, *J* = 6.6 Hz, 6H); ^{13}C NMR (100 MHz, $CDCl_3$, ppm) δ = 171.8, 157.9, 137.9, 133.3, 130.1 (+, CH), 129.5 (+, CH, 2C), 128.2 (+, CH, 2C), 107.8, 56.0, 48.4, 46.5 (–, CH_2), 45.4 (–, CH_2), 34.1 (–, CH_2), 26.2 (+, CH), 23.8 (+, CH_3, 2C), 22.5 (+, CH_3, 2C), 21.2 (+, CH_3), 19.4 (+, CH_3, 2C). MS (FAB, Matrix: 3-NBA): *m/z* (%) = 414 (13) $[M+2H]^+$, 413 (54) $[M+H]^+$, 412 (14) $[M]^+$, 411 (15), 313 (24), 312 (100), 105 (78). HRMS–FAB *(m/z)*: $[M+H]^+$ Calcd for $C_{23}H_{37}ON_6$, 413.3029; Found 413.3030; IR (ATR, ṽ) = 3432 (vw), 3292 (w), 3078 (vw), 3055 (vw), 2956 (m), 2928 (w), 2868 (w), 1643 (vs), 1536 (m), 1516 (m), 1465 (s), 1421 (vs), 1402 (vs), 1380 (m), 1364 (vs), 1307 (w), 1242 (vs), 1224 (vs), 1181 (m), 1149 (vs), 1126 (vs), 1099 (m), 1033 (s), 1010 (m), 962 (w), 922 (w), 911 (w), 885 (w), 843 (w), 823 (w), 796 (s), 752 (m), 727 (m), 698 (w), 677 (w), 640 (w), 629 (w) cm^{-1}.

Additional information on the chemical synthesis is available *via* Chemotion repository:
https://doi.org/10.14272/reaction/SA-FUHFF-UHFFFADPSC-MORMFGNFEX-UHFFFADPSC-NUHFF-NIWVK-NUHFF-ZZZ
Additional information on the analysis of the target compound is available *via* Chemotion repository:
https://doi.org/10.14272/MORMFGNFEXIKDM-IMVLJIQESA-N.1

(*E*)-*N*-((1-(3,5-difluorobenzyl)-3-(3,3-diisopropyltriaz-1-en-1-yl)-1*H*-pyrazol-4-yl)methyl)acetamide (9d)

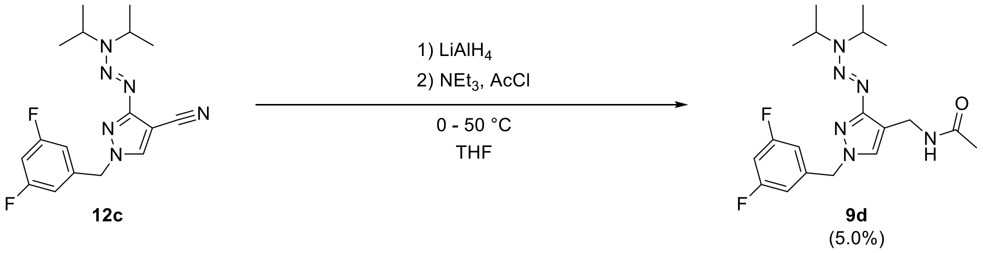

Step 1: 1-(3,5-difluorobenzyl)-3-(3,3-diisopropyltriaz-1-en-1-yl)-1*H*-pyrazole-4-carbonitrile (**12c**, 261 mg, 754 µmol, 1.00 equiv) was dissolved in 75 mL of dry THF under nitrogen atmosphere. The solution was cooled to 0 °C before lithium aluminum hydride (85.9 mg, 2.26 mL, 2.26 mmol, 3.00 equiv) was slowly added. The cooling was removed and the solution was stirred at 21 °C for 14 hours, then additional 5 hours at 50 °C. The solution was cooled to 21 °C and the reaction was quenched with a saturated K/Na-tartrate solution (75 mL). The organic solvent was removed under reduced pressure and the remaining aqueous phase was extracted with methylene chloride (3 x 75 mL). The combined organic layers were washed with brine (150 mL) and dried over Na_2SO_4. The solvent was removed under reduced pressure to give the crude (1-(3,5-difluorobenzyl)-3-(3,3-diisopropyltriaz-1-en-1-yl)-1*H*-pyrazol-4-yl)methanamine, which was used for the next step without further purification.

Step 2: The crude (1-(3,5-difluorobenzyl)-3-(3,3-diisopropyltriaz-1-en-1-yl)-1*H*-pyrazol-4-yl)methanamine was dissolved in 75 mL of dry THF under nitrogen atmosphere. Triethylamine (229 mg, 315 µL, 2.26 mmol, 3.00 equiv) was added and the solution was cooled to 0 °C before acetyl chloride (88.8 mg, 80.4 µL, 1.13 mmol, 1.50 equiv) was added dropwise. The cooling was removed and the solution was stirred for 14 hours at 21 °C. The reaction was quenched with a saturated K_2CO_3 solution (75 mL) and the organic solvent was removed under reduced pressure. The remaining aqueous phase was extracted with methylene chloride (3 × 75 mL). The combined organic layers were washed with brine (150 mL) and dried over Na_2SO_4. The solvent was removed under reduced pressure to give the crude product. The obtained crude product was purified *via* flash-chromatography on silica gel using methylene chloride/methanol 50:1 to 30:1. It was further purified *via* HPLC using MeCN/H_2O 10:1, to give (*E*)-*N*-((1-(3,5-difluorobenzyl)-3-(3,3-diisopropyltriaz-1-en-1-yl)-1*H*-pyrazol-4-yl)methyl)acetamide (**9d**, 15.0 mg, 38.2 µmol, 5% yield) as a colorless oil.

9d: (E)-N-((1-(3,5-difluorobenzyl)-3-(3,3-diisopropyltriaz-1-en-1-yl)-1H-pyrazol-4-yl)methyl)acetamide; Formula: $C_{19}H_{26}F_2N_6O$; Exact Mass: 392.2136; Smiles: CC(=O)NCc1cn(nc1/N=N/N(C(C)C)C(C)C)Cc1cc(F)cc(c1)F; InChIKey: NQVQLXTURKHERM-WJTDDFOZSA-N

R_f = 0.47 (cyclohexane/ethyl acetate 1:1). ^{1}H NMR (400 MHz, $CDCl_3$, ppm) δ = 7.30 (s, 1H), 6.80–6.65 (m, 3H), 5.91 (br.t, *J* = 5.6 Hz, 1H), 5.43 (br.s, 1H), 5.15 (s,2H), 4.34 (d, *J* = 5.5 Hz, 2H), 4.02 (br.s, 1H), 1.93 (s, 3H), 1.36 (d, *J* = 6.6 Hz, 6H), 1.22 (d, *J* = 6.8 Hz, 6H); ^{19}F NMR (375 MHz, ppm) δ = -109.05; ^{13}C NMR (100 MHz, $CDCl_3$, ppm) δ = 169.5, 163.3 (2C; dd, *J* = 249.5 Hz, *J* = 12.6 Hz), 158.5, 140.6 (t, *J* = 8.9 Hz), 130.7 (+, CH), 110.6 (+, CH, 2C; dd, *J* = 18.6 Hz, *J* = 7.2 Hz), 108.2, 103.5 (+, CH; t, *J* = 25.3 Hz), 55.2 (–, CH_2; t, *J* = 2.3 Hz), 48.4 (+, CH), 45.5 (+, CH), 34.3 (–, CH_2), 23.8 (+, CH_3, 2C), 23.6 (+, CH_3), 19.5 (+, CH_3, 2C); MS (FAB, Matrix: 3-NBA): *m/z* (%) = 394 (20) $[M+2H]^+$, 393 (100) $[M+H]^+$, 392 (14) $[M]^+$, 375 (11), 334 (17), 292 (52), 154 (30), 138 (12), 137 (19), 136 (24), 127 (32), 100 (21). HRMS–FAB *(m/z)*: $[M+H]^+$ Calcd for $C_{19}H_{27}ON_6F_2$, 393.2214; Found 393.2212; IR (ATR, ṽ) = 3288 (w), 3081 (vw), 2975 (w), 2931 (w), 2870 (vw), 1650 (s), 1626 (vs), 1596 (s), 1543 (m), 1460 (s), 1418 (vs), 1364 (vs), 1340 (s), 1317 (s), 1245 (vs), 1224 (vs), 1153 (vs), 1118 (vs), 1098 (m), 1034 (m), 1001 (m), 846 (s), 728 (vs), 670 (m), 645 (w), 526 (m), 510 (m) cm^{-1}.

Additional information on the chemical synthesis is available *via* Chemotion repository:
https://doi.org/10.14272/reaction/SA-FUHFF-UHFFFADPSC-NQVQLXTURK-UHFFFADPSC-NUHFF-NCVEK-NUHFF-ZZZ
Additional information on the analysis of the target compound is available *via* Chemotion repository:
https://doi.org/10.14272/NQVQLXTURKHERM-WJTDDFOZSA-N.1

(E)-N-((3-(3,3-Diisopropyltriaz-1-en-1-yl)-1-ethyl-1H-pyrazol-4-yl)methyl)acetamide (9e)

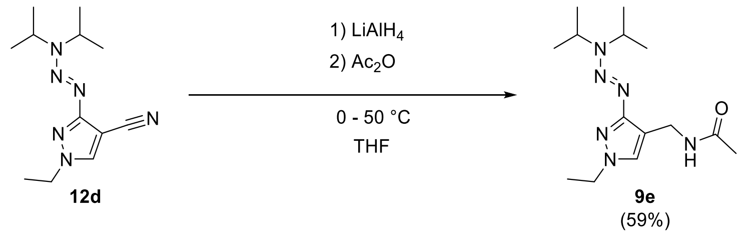

Step 1: (E)-3-(3,3-Diisopropyltriaz-1-en-1-yl)-1-ethyl-1H-pyrazole-4-carbonitrile (**12d**, 88.7 mg, 357 µmol, 1.00 equiv) was dissolved in 60 mL of dry THF under nitrogen atmosphere. The solution was cooled to 0 °C before lithium aluminum hydride (40.7 mg, 1.07 mL, 1.07 mmol, 1.00M, 3.00 equiv) was slowly added. The cooling was removed and the solution was stirred at 21 °C for 14 hours, then additional 5 hours at 50 °C. The solution was cooled to 21 °C and the reaction was quenched with a saturated K/Na-tartrate solution (60 mL). The organic solvent was removed under reduced pressure and the remaining aqueous phase was extracted with methylene chloride (3 x 60 mL). The combined organic layers were washed with brine (150 mL) and dried over Na_2SO_4. The solvent was removed under reduced pressure to give the crude (E)-(3-(3,3-diisopropyltriaz-1-en-1-yl)-1-ethyl-1H-pyrazol-4-yl)methanamine, which was used for the next step without further purification.
Step 2: The crude (E)-(3-(3,3-diisopropyltriaz-1-en-1-yl)-1-ethyl-1H-pyrazol-4-yl)methanamine was dissolved in 60 mL of dry THF under nitrogen atmosphere. The solution was cooled to 0 °C before acetic anhydride (54.7 mg, 50.6 µL, 536 µmol, 1.50 equiv) was slowly added. The cooling was removed and the solution was stirred for 14 hours at 21 °C. The reaction was quenched with a saturated K_2CO_3 solution (60 mL) and the organic solvent was removed under reduced pressure. The remaining aqueous phase was extracted with methylene chloride (3 × 60 mL). The combined organic layers were washed with brine (150 mL) and dried over Na_2SO_4. The solvent was removed under reduced pressure to give the crude product. The obtained crude product was purified *via* flash-chromatography on silica gel using cyclohexane/ethyl acetate 2:1 to 1:1 to pure ethyl acetate, to give (E)-N-((3-(3,3-diisopropyltriaz-1-en-1-yl)-1-ethyl-1H-pyrazol-4-yl)methyl)acetamide (**9e**, 62.1 mg, 211 µmol, 59% yield) as a light-brown oil.

9e: (E)-N-((3-(3,3-diisopropyltriaz-1-en-1-yl)-1-ethyl-1H-pyrazol-4-yl)methyl)acetamide; Formula: $C_{14}H_{26}N_6O$; Exact Mass: 294.2168; Smiles: CCn1cc(c(n1)/N=N/N(C(C)C)C(C)C)CNC(=O)C; InChIKey: SSTDOKZYUWDQQQ-FBMGVBCBSA-N

R_f = 0.15 (pure ethyl acetate). ^{1}H NMR (400 MHz, $CDCl_3$, ppm) δ = 7.24 (s, 1H), 5.94 (t, J = 5.3 Hz, 1H), 5.41 (br.s, 1H), 4.30 (d, J = 5.4 Hz, 2H), 4.11–3.89 (m, 3H), 1.90 (s, 3H), 1.44 (t, J = 7.3 Hz, 3H), 1.32 (br.s, 6H), 1.19 (br.s, 6H); ^{13}C NMR (100 MHz, $CDCl_3$, ppm) δ = 169.4, 157.8, 129.5 (+, CH), 106.6, 48.2 (+, CH), 47.1 (–, CH_2), 45.3 (+, CH), 34.3 (–, CH_2), 23.7 (+, CH_3, 2C), 23.5 (+, CH_3), 19.4 (+, CH_3, 2C), 15.5 (+, CH_3); MS (FAB, Matrix: 3-NBA): m/z (%) = 296 (17) $[M+2H]^+$, 295 (99) $[M+H]^+$, 294 (13) $[M]^+$, 293 (26) $[M-H]^+$, 237 (10), 236 (69), 195 (13), 194 (100), 154 (12), 150 (14), 139 (12), 133 (41), 125 (34), 124 (15), 109 (13), 107 (12), 100 (14), 95 (16), 93 (12), 91 (19). HRMS–FAB *(m/z)*: $[M+H]^+$ Calcd for $C_{14}H_{27}ON_6$, 295.2241; Found 295.2243; IR (ATR, ṽ) = 3360 (w), 3262 (w), 3210 (w), 3077 (w), 2976 (w), 2932 (w), 2873 (w), 2225 (vw), 1650 (vs), 1571 (s), 1462 (w), 1407 (vs), 1380 (s), 1364 (vs), 1344 (vs), 1334 (s), 1302 (m), 1254 (vs), 1238 (vs), 1224 (vs), 1181 (s), 1166 (s), 1153 (vs), 1129 (s), 1096 (s), 1034 (s), 1018 (m), 992 (w), 953 (w), 926 (w), 912 (w), 882 (w), 849 (w), 829 (m), 792 (w), 724 (m), 713 (s), 701 (m), 639 (m), 606 (m) cm^{-1}.

Additional information on the chemical synthesis is available *via* Chemotion repository:
https://doi.org/10.14272/reaction/SA-FUHFF-UHFFFADPSC-SSTDOKZYUW-UHFFFADPSC-NUHFF-NUVCO-NUHFF-ZZZ
Additional information on the analysis of the target compound is available *via* Chemotion repository:
https://doi.org/10.14272/SSTDOKZYUWDQQQ-FBMGVBCBSA-N.1

(*E*)-*N*-((1-Cyclopentyl-3-(3,3-diisopropyltriaz-1-en-1-yl)-1*H*-pyrazol-4-yl)methyl)acetamide (9f)

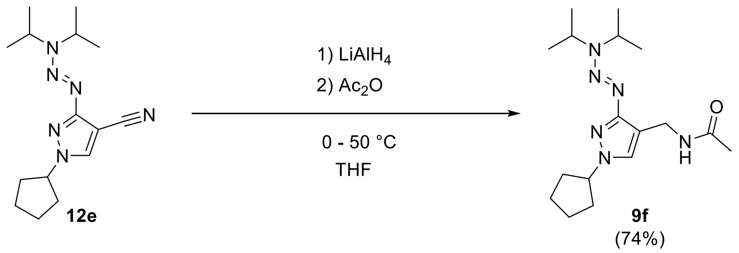

Step 1: 1-Cyclopentyl-3-(3,3-diisopropyltriaz-1-en-1-yl)-1*H*-pyrazole-4-carbonitrile (**12e**, 132 mg, 459 µmol, 1.00 equiv) was dissolved in 75 mL of dry THF under nitrogen atmosphere. The solution was cooled to 0 °C before lithium aluminum hydride (52.2 mg, 1.38 mL, 1.38 mmol, 3.00 equiv) was slowly added. The cooling was removed and the solution was stirred at 21 °C for 14 hours, then additional 5 hours at 50 °C. The solution was cooled to 21 °C and the reaction was quenched with a saturated K/Na-tartrate solution (75 mL). The organic solvent was removed under reduced pressure and the remaining aqueous phase was extracted with methylene chloride (3 x 75 mL). The combined organic layers were washed with brine (200 mL) and dried over Na_2SO_4. The solvent was removed under reduced pressure to give the crude (1-cyclopentyl-3-(3,3-diisopropyltriaz-1-en-1-yl)-1H-pyrazol-4-yl)methanamine, which was used for the next step without further purification.
Step 2: The crude (1-cyclopentyl-3-(3,3-diisopropyltriaz-1-en-1-yl)-1*H*-pyrazol-4-yl)methanamine was dissolved in 75 mL of dry THF under nitrogen atmosphere. The solution was cooled to 0 °C before acetic anhydride (70.3 mg, 65.0 µL, 688 µmol, 1.50 equiv) was slowly added. The cooling was removed and the solution was stirred for 14 hours at 21 °C. The reaction was quenched with a saturated K_2CO_3 solution (75 mL) and the organic solvent was removed under reduced pressure. The remaining aqueous phase was extracted with methylene chloride (3 × 75 mL). The combined organic layers were washed with brine (200 mL) and dried over Na_2SO_4. The solvent was removed under reduced pressure to give the crude product. The obtained crude product was purified *via* flash-chromatography on silica gel using cyclohexane/ethyl acetate 2:1 to 1:1 to pure ethyl acetate, to give *N*-((1-cyclopentyl-3-(3,3-diisopropyltriaz-1-en-1-yl)-1*H*-pyrazol-4-yl)methyl)acetamide (**9f**, 114 mg, 340 µmol, 74% yield) as a light-brown oil.

9f: (E)-N-((1-cyclopentyl-3-(3,3-diisopropyltriaz-1-en-1-yl)-1H-pyrazol-4-yl)methyl)acetamide; Formula: $C_{17}H_{30}N_6O$; Exact Mass: 334.2481; Smiles: CC(=O)NCc1cn(nc1/N=N/N(C(C)C)C(C)C)C1CCCC1; InChIKey: GZXDXORFZUUVGU-XUTLUUPISA-N

R_f = 0.03 (cyclohexane/ethyl acetate 2:1). ^{1}H NMR (400 MHz, $CDCl_3$, ppm) δ = 7.28 (s, 1H), 5.91 (t, *J* = 5.5 Hz, 1H), 5.49 (br.s, 1H), 4.50 (p, *J* = 7.4 Hz, 1H), 4.30 (d, *J* = 5.4 Hz, 2H), 3.97 (br.s, 1H), 2.16–1.96 (m, 4H), 1.91 (s, 3H), 1.88–1.77 (m, 2H), 1.71–1.58 (m, 2H), 1.32 (s, 6H), 1.19 (s, 6H); ^{13}C NMR (100 MHz, $CDCl_3$, ppm) δ = 169.3, 157.7, 128.8 (+, CH), 106.2, 63.2 (+, CH), 48.0 (+, CH), 45.0 (+, CH), 34.4 (–, CH_2), 32.8 (–, CH_2, 2C), 24.1 (–, CH_2, 2C), 23.7 (+, CH_3, 2C), 23.6 (+, CH_3), 19.5 (+, CH_3, 2C); MS (FAB, Matrix: 3-NBA): *m/z* (%) = 235 (14) $[M+2H]^+$, 234 (100) $[M+H]^+$, 233 (6) $[M]^+$, 232 (10) $[M-H]^+$, 192 (10), 155 (15), 154 (48), 139 (10), 138 (20), 137 (30), 136 (34), 109 (11), 107 (13), 97 (11), 95 (15), 91 (13); MS (FAB, Matrix: 3-NBA): *m/z* (%) = 336 (20) $[M+2H]^+$, 335 (100) $[M+H]^+$, 334 (17) $[M]^+$, 333 (17) $[M-H]^+$, 276 (43), 235 (12), 234 (85), 100 (23), 97 (31), 96 (17), 95 (15), 85 (10). HRMS–FAB *(m/z)*: $[M+H]^+$ Calcd for $C_{17}H_{31}ON_6$, 355.2554; Found 335.2555; IR (ATR, ṽ) = 3288 (w), 3274 (w), 3080 (vw), 3070 (vw), 2969 (m), 2938 (w), 2871 (w), 2230 (vw), 1650 (vs), 1543 (s), 1465 (m), 1421 (vs), 1402 (vs), 1363 (vs), 1340 (m), 1319 (w), 1239 (vs), 1224 (vs), 1152 (vs), 1128 (s), 1095 (m), 1033 (s), 1011 (w), 911 (w), 885 (vw), 847 (vw), 806 (w), 728 (m), 643 (w) cm^{-1}.

Additional information on the chemical synthesis is available *via* Chemotion repository:
https://doi.org/10.14272/reaction/SA-FUHFF-UHFFFADPSC-GZXDXORFZU-UHFFFADPSC-NUHFF-NHIGY-NUHFF-ZZZ
Additional information on the analysis of the target compound is available *via* Chemotion repository:
https://doi.org/10.14272/GZXDXORFZUUVGU-XUTLUUPISA-N.1

(*E*)-*N*-((1-Cyclopentyl-3-(3,3-diisopropyltriaz-1-en-1-yl)-1*H*-pyrazol-4-yl)methyl)benzamide (9g)

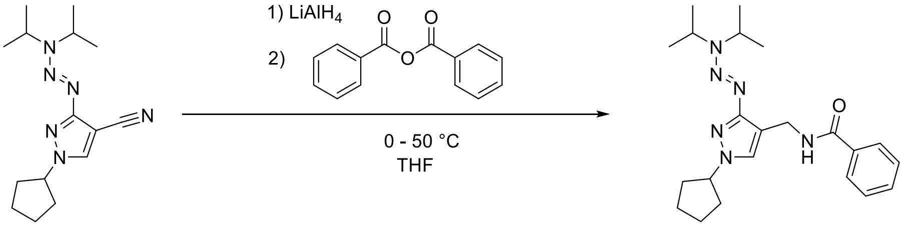

Step 1: (*E*)-1-cyclopentyl-3-(3,3-diisopropyltriaz-1-en-1-yl)-1*H*-pyrazole-4-carbonitrile (**12e**, 327 mg, 1.14 mmol, 1.00 equiv) was dissolved in 180 mL of dry THF under nitrogen atmosphere. The solution was cooled to 0 °C before lithium aluminum hydride (129 mg, 3.41 mL, 3.41 mmol, 3.00 equiv) was slowly added. The cooling was removed and the solution was stirred at 21 °C for 14 hours, then additional 5 hours at 50 °C. The solution was cooled to 21 °C and the reaction was quenched with a saturated K/Na-tartrate solution (150 mL). The organic solvent was removed under reduced pressure and the remaining aqueous phase was extracted with methylene chloride (3 x 150 mL). The combined organic layers were washed with brine (400 mL) and dried over Na_2SO_4. The solvent was removed under reduced pressure to give the crude (*E*)-(1-cyclopentyl-3-(3,3-diisopropyltriaz-1-en-1-yl)-1*H*-pyrazol-4-yl)methanamine, which was used for the next step without further purification.
Step 2: The crude (*E*)-(1-cyclopentyl-3-(3,3-diisopropyltriaz-1-en-1-yl)-1*H*-pyrazol-4-yl)methanamine was dissolved in 150 mL of dry THF under nitrogen atmosphere. The solution was cooled to 0 °C before benzoyl benzoate (385 mg, 1.70 mmol, 1.50 equiv) was slowly added. The cooling was removed and the solution was stirred for 14 hours at 21 °C. The reaction was quenched with a saturated K_2CO_3 solution (150 mL) and the organic solvent was removed under reduced pressure. The remaining aqueous phase was extracted with methylene chloride (3 × 150 mL). The combined organic layers were washed with brine (400 mL) and dried over Na_2SO_4. The solvent was removed under reduced pressure to give the crude product. The obtained crude product was purified *via* flash-chromatography on silica gel using cyclohexane/ethyl acetate 4:1 to 1:1, to give (*E*)-*N*-((1-cyclopentyl-3-(3,3-diisopropyltriaz-1-en-1-yl)-1*H*-pyrazol-4-yl)methyl)benzamide (**9g**, 285 mg, 719 µmol, 63% yield) as a colorless solid.

9g: (E)-N-((1-cyclopentyl-3-(3,3-diisopropyltriaz-1-en-1-yl)-1H-pyrazol-4-yl)methyl)benzamide; Formula: $C_{22}H_{32}N_6O$; Exact Mass: 396.2638; Smiles: CC(N(C(C)C)/N=N/c1nn(cc1CNC(=O)c1ccccc1)C1CCCC1)C; InChIKey: SMDRKVWNQXOQTQ-SHHOIMCASA-N

R_f = 0.50 (cyclohexane/ethyl acetate 2:1). ^{1}H NMR (400 MHz, $CDCl_3$, ppm) δ = 7.75–7.67 (m, 2H), 7.50–7.42 (m, 1H), 7.42–7.34 (m, 3H), 6.47 (br.t, *J* = 5.4 Hz, 1H), 5.51 (br.s, 1H), 4.60–4.46 (m, 3H), 3.95 (br.s, 1H), 2.18–2.00 (m, 4H), 1.92–1.79 (m, 2H), 1.73–1.60 (m, 2H), 1.27–1.14 (m, 12H). ^{13}C NMR (100 MHz, $CDCl_3$, ppm) δ = 167.1, 157.8, 135.0, 131.4 (+, CH), 129.1 (+, CH), 128.6 (+, CH, 2C), 127.0 (+, CH, 2C), 106.1, 63.2 (+, CH), 48.2 (+, CH), 45.1 (+, CH), 35.0 (–, CH_2), 32.8 (–, CH_2, 2C), 24.1 (–, CH_2, 2C), 23.7 (+, CH_3, 2C), 19.5 (+, CH_3, 2C); MS (FAB, Matrix: 3-NBA): *m/z* (%) = 397 (39) $[M+H]^+$, 396 (13) $[M]^+$, 395 (20) $[M-H]^+$, 297 (20), 296 (100), 277 (11), 276 (59), 269 (14), 165 (14), 133 (74), 106 (10), 105 (93), 100 (12), 99 (11), 97 (54), 96 (14). HRMS–FAB *(m/z)*: $[M+H]^+$ Calcd for $C_{22}H_{33}ON_6$, 397.2710; Found 397.2710. IR (ATR, ṽ) = 3360 (w), 3061 (vw), 2973 (m), 2867 (w), 1738 (vw), 1656 (s), 1636 (s), 1599 (w), 1575 (w), 1560 (w), 1534 (vs), 1489 (s), 1463 (m), 1441 (s), 1421 (vs), 1398 (vs), 1361 (vs), 1341 (m), 1327 (m), 1299 (s), 1239 (vs), 1224 (vs), 1190 (w), 1179 (m), 1153 (vs), 1129 (vs), 1098 (m), 1078 (m), 1033 (s), 1011 (m), 1003 (m), 980 (w), 942 (w), 912 (m), 880 (w), 849 (w), 822 (w), 812 (w), 802 (w), 793 (w), 725 (vs), 696 (vs), 670 (w), 632 (m) cm^{-1}.

Additional information on the chemical synthesis is available *via* Chemotion repository:
https://doi.org/10.14272/reaction/SA-FUHFF-UHFFFADPSC-SMDRKVWNQX-UHFFFADPSC-NUHFF-NSKKF-NUHFF-ZZZ
Additional information on the analysis of the target compound is available *via* Chemotion repository:
https://doi.org/10.14272/SMDRKVWNQXOQTQ-SHHOIMCASA-N.1

(*E*)-*N*-((3-(3,3-Diisopropyltriaz-1-en-1-yl)-1-isobutyl-1*H*-pyrazol-4-yl)methyl)acetamide (9h)

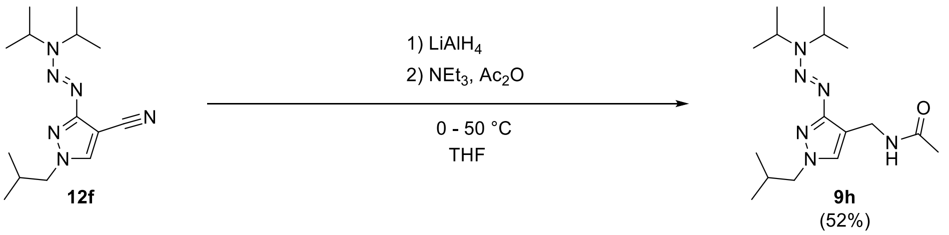

Step 1: (*E*)-3-(3,3-Diisopropyltriaz-1-en-1-yl)-1-isobutyl-1*H*-pyrazole-4-carbonitrile (**12f**, 186 mg, 672 µmol, 1.00 equiv) was dissolved in 27 mL of dry THF under nitrogen atmosphere. The solution was cooled to 0 °C before lithium aluminum hydride (76.5 mg, 2.02 mL, 2.02 mmol, 3.00 equiv) was slowly added. The cooling was removed and the solution was stirred at 21 °C for 14 hours, then additional 5 hours at 50 °C. The solution was cooled to 21 °C and the reaction was quenched with a saturated K/Na-tartrate solution (30 mL). The organic solvent was removed under reduced pressure and the remaining aqueous phase was extracted with methylene chloride (3 x 30 mL). The combined organic layers were washed with brine (100 mL) and dried over Na_2SO_4. The solvent was removed under reduced pressure to give the crude (*E*)-(3-(3,3-diisopropyltriaz-1-en-1-yl)-1-isobutyl-1*H*-pyrazol-4-yl)methanamine, which was used for the next step without further purification.
Step 2: Crude (*E*)-(3-(3,3-diisopropyltriaz-1-en-1-yl)-1-isobutyl-1*H*-pyrazol-4-yl)methanamine was dissolved in 27 mL of dry THF under nitrogen atmosphere. Triethylamine (204 mg, 280 µL, 2.02 mmol, 3.00 equiv) was added and the solution was cooled to 0 °C before acetic anhydride (103 mg, 95.3 µL, 1.01 mmol, 1.50 equiv) was added dropwise. The cooling was removed and the solution was stirred for 14 hours at 21 °C. The reaction was quenched with a saturated K_2CO_3 solution (30 mL) and the organic solvent was removed under reduced pressure. The remaining aqueous phase was extracted with methylene chloride (3 × 30 mL). The combined organic layers were washed with brine (100 mL) and dried over Na_2SO_4. The solvent was removed under reduced pressure to give the crude product. The obtained crude product was purified *via* flash-chromatography on silica gel using methylene chloride/methanol 50:1 to 30:1, giving (*E*)-*N*-((3-(3,3-diisopropyltriaz-1-en-1-yl)-1-isobutyl-1*H*-pyrazol-4-yl)methyl)acetamide (**9h**, 114 mg, 353 µmol, 52% yield) as a colorless oil.

9h: (E)-N-((3-(3,3-diisopropyltriaz-1-en-1-yl)-1-isobutyl-1H-pyrazol-4-yl)methyl)acetamide; Formula: $C_{16}H_{30}N_6O$; Exact Mass: 322.2481; Smiles: CC(=O)NCc1cn(nc1/N=N/N(C(C)C)C(C)C)CC(C)C; InChIKey: RZSNDUJLUYXXQT-CZIZESTLSA-N

R_f = 0.06 (cyclohexane/ethyl acetate 1:1). ^{1}H NMR (400 MHz, $CDCl_3$, ppm) δ = 7.20 (s, 1H), 5.92 (s, 1H), 5.43 (br.s, 1H), 4.30 (d, *J* = 5.4 Hz, 2H), 3.98 (br.s, 1H), 3.75 (d, *J* = 7.3 Hz, 2H), 2.22 (hept, *J* = 6.8 Hz, 1H), 1.90 (s, 3H), 1.32 (br.s, 6H), 1.19 (br.s, 6H), 0.88 (d, *J* = 6.7 Hz, 6H); ^{13}C NMR (100 MHz, $CDCl_3$, ppm) δ = 169.3, 157.8, 130.7 (+, CH), 106.3, 59.9 (–, CH_2), 48.1 (+, CH), 45.2 (+, CH), 34.3 (–, CH_2), 29.4 (+, CH), 23.7 (+, 2C, CH_3), 23.6 (+, 2C, CH_3), 20.1 (+, CH_3), 19.4 (+, 2C, CH_3); MS (FAB, Matrix: 3-NBA): *m/z* (%) = 324 (11) $[M+2H]^+$, 323 (58) $[M+H]^+$, 322 (7) $[M]^+$, 321 (13) $[M-H]^+$, 265 (11), 264 (65), 223 (13), 222 (100), 178 (14), 153 (19), 152 (11), 109 (14), 100 (21), 99 (10), 97 (21), 96 (15), 95 (20), 93 (13). HRMS–FAB *(m/z)*: [M+H]+ Calcd for $C_{16}H_{31}ON_6$, 323.2559; Found 323.2561; IR (ATR, ṽ) = 2968 (m), 2931 (m), 2871 (w), 1650 (vs), 1541 (s), 1465 (s), 1421 (vs), 1402 (vs), 1364 (vs), 1341 (s), 1244 (vs), 1220 (vs), 1153 (vs), 1126 (vs), 1095 (s), 1034 (vs), 1014 (s), 946 (m), 924 (s), 911 (s), 851 (m), 841 (m), 819 (m), 806 (m), 730 (vs), 643 (m), 628 (m) cm^{-1}.

Additional information on the chemical synthesis is available *via* Chemotion repository:
https://doi.org/10.14272/reaction/SA-FUHFF-UHFFFADPSC-RZSNDUJLUY-UHFFFADPSC-NUHFF-NJAII-NUHFF-ZZZ
Additional information on the analysis of the target compound is available *via* Chemotion repository:
https://doi.org/10.14272/RZSNDUJLUYXXQT-CZIZESTLSA-N.1

(*E*)-2-(4-(Acetamidomethyl)-3-(3,3-diisopropyltriaz-1-en-1-yl)-1*H*-pyrazol-1-yl)ethyl acetate (9i)

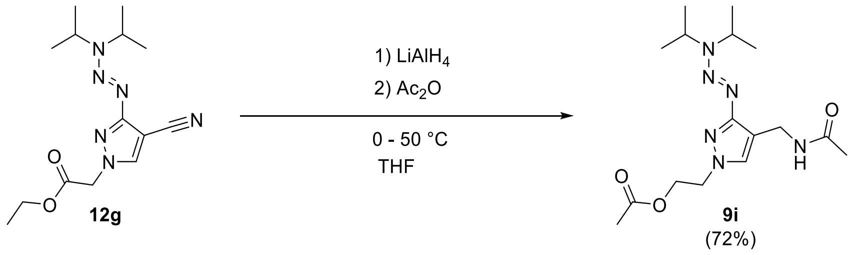

Step 1: Ethyl (*E*)-2-(4-cyano-3-(3,3-diisopropyltriaz-1-en-1-yl)-1*H*-pyrazol-1-yl)acetate (**12g**, 277 mg, 904 µmol, 1.00 equiv) was dissolved in 150 mL of dry THF under nitrogen atmosphere. The solution was cooled to 0 °C before lithium aluminum hydride (103 mg, 2.71 mL, 2.71 mmol, 3.00 equiv) was slowly added. The cooling was removed and the solution was stirred at 21 °C for 14 hours, then additional 5 hours at 50 °C. The solution was cooled to 21 °C and the reaction was quenched with a saturated K/Na-tartrate solution (150 mL). The organic solvent was removed under reduced pressure and the remaining aqueous phase was extracted with methylene chloride (3 x 150 mL). The combined organic layers were washed with brine (400 mL) and dried over Na_2SO_4. The solvent was removed under reduced pressure to give the crude (*E*)-2-(4-(aminomethyl)-3-(3,3-diisopropyltriaz-1-en-1-yl)-1*H*-pyrazol-1-yl)ethan-1-ol, which was used for the next step without further purification.
Step 2: The crude (*E*)-2-(4-(aminomethyl)-3-(3,3-diisopropyltriaz-1-en-1-yl)-1*H*-pyrazol-1-yl)ethan-1-ol was dissolved in 150 mL of dry THF under nitrogen atmosphere. The solution was cooled to 0 °C before acetic anhydride (369 mg, 342 µL, 3.61 mmol, 4.00 equiv) was slowly added. The cooling was removed and the solution was stirred for 14 hours at 21 °C. The reaction was quenched with a saturated K_2CO_3 solution (150 mL) and the organic solvent was removed under reduced pressure. The remaining aqueous phase was extracted with methylene chloride (3 × 150 mL). The combined organic layers were washed with brine (400 mL) and dried over Na_2SO_4. The solvent was removed under reduced pressure to give the crude product. The obtained crude product was purified *via* flash-chromatography on silica gel using cyclohexane/ethyl acetate 2:1 to 1:1 to pure ethyl acetate, giving (*E*)-2-(4-(acetamidomethyl)-3-(3,3-diisopropyltriaz-1-en-1-yl)-1*H*-pyrazol-1-yl)ethyl acetate (**9i**, 229 mg, 650 µmol, 72% yield) as a light-brown oil.

9i: (E)-2-(4-(acetamidomethyl)-3-(3,3-diisopropyltriaz-1-en-1-yl)-1*H*-pyrazol-1-yl)ethyl acetate; Formula: $C_{16}H_{28}N_6O_3$; Exact Mass: 352.2223; Smiles: CC(=O)NCc1cn(nc1/N=N/N(C(C)C)C(C)C)CCOC(=O)C; InChIKey: BBXJSLXPCZXILR-CZIZESTLSA-N

R_f = 0.14 (pure ethyl acetate). ^{1}H NMR (400 MHz, $CDCl_3$, ppm) δ = 7.20 (s, 1H), 6.06 (t, *J* = 5.5 Hz, 1H), 5.31 (s, 1H), 4.32 (t, *J* = 5.4 Hz, 2H), 4.24 (d, *J* = 5.5 Hz, 2H), 4.14 (t, *J* = 5.4 Hz, 2H), 3.92 (s, 1H), 1.93 (s, 3H), 1.84 (s, 3H), 1.25 (s, 6H), 1.13 (s, 6H); ^{13}C NMR (100 MHz, $CDCl_3$, ppm) δ = 170.5, 169.3, 158.1, 130.8 (+, CH), 107.2, 62.6 (–, CH_2), 50.8 (–, CH_2), 48.2 (+, CH), 45.2 (+, CH), 34.2 (–, CH_2), 23.4 (+, CH_3, 2C), 23.2 (+, CH_3), 20.7 (+, CH_3), 19.2 (+, CH_3, 2C); MS (FAB, Matrix: 3-NBA): *m/z* (%) = 354 (13) $[M+2H]^+$, 353 (62) $[M+H]^+$, 352 (10) $[M]^+$, 351 (11) $[M-H]^+$, 295 (10), 294 (50), 253 (15), 252 (100), 208 (18), 182 (16); HRMS–FAB *(m/z)*: $[M+H]^+$ Calcd for $C_{16}H_{29}O_3N_6$, 353.2296; Found 353.2297; IR (ATR, ṽ) = 3284 (w), 3082 (vw), 2973 (w), 2932 (w), 2873 (vw), 1738 (s), 1652 (s), 1541 (m), 1419 (vs), 1364 (vs), 1227 (vs), 1153 (vs), 1128 (s), 1096 (m), 1034 (vs), 952 (w), 932 (m), 909 (m), 885 (w), 849 (w), 813 (m), 790 (m), 769 (w), 730 (m), 714 (m), 701 (m), 667 (m), 630 (m), 602 (s) cm^{-1}.

Additional information on the chemical synthesis is available *via* Chemotion repository:
https://doi.org/10.14272/reaction/SA-FUHFF-UHFFFADPSC-BBXJSLXPCZ-UHFFFADPSC-NUHFF-NJAII-NUHFF-ZZZ
Additional information on the analysis of the target compound is available *via* Chemotion repository:
https://doi.org/10.14272/BBXJSLXPCZXILR-CZIZESTLSA-N.1

1-(6-Benzyl-4,6-dihydro-3*H*-pyrazolo[3,4-*d*][1,2,3]triazin-3-yl)ethan-1-one (5a)

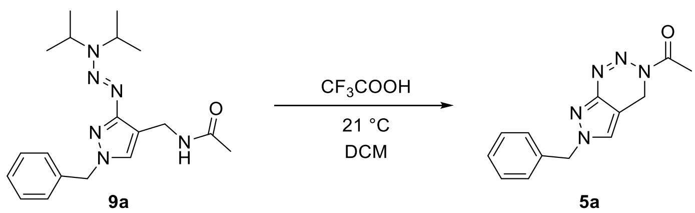

(*E*)-*N*-((1-benzyl-3-(3,3-diisopropyltriaz-1-en-1-yl)-1*H*-pyrazol-4-yl)methyl)acetamide (**9a**, 32.0 mg, 89.8 µmol, 1.00 equiv) was dissolved in 5 mL of dry methylene chloride under nitrogen atmosphere. 2,2,2-Trifluoroacetic acid (30.7 mg, 20.6 µL, 269 µmol, 3.00 equiv) was added and the mixture was stirred at 21 °C for 14 hours. The reaction was diluted with 10 mL of saturated K_2CO_3 solution and the aqueous phase was extracted with 3 × 10 mL of methylene chloride. The combined organic layers were washed with 20 mL of brine and dried over Na_2SO_4. The solvent was removed under reduced pressure to give the crude product. The obtained crude product was purified *via* flash-chromatography on silica gel using cyclohexane/ethyl acetate 4:1 to 2:1, giving 1-(6-benzyl-4,6-dihydro-3*H*-pyrazolo[3,4-*d*][1,2,3]triazin-3-yl)ethan-1-one (**5a**, 13.5 mg, 52.9 µmol, 59% yield) as a colorless solid.

5a: 1-(6-benzyl-4,6-dihydro-3H-pyrazolo[3,4-d][1,2,3]triazin-3-yl)ethan-1-one; Formula: $C_{13}H_{13}N_5O$; Exact Mass: 255.1120; Smiles: CC(=O)N1N=Nc2c(C1)cn(n2)Cc1ccccc1; InChIKey: PGERMRZARQFUFX-UHFFFAOYSA-N

R_f = 0.25 (cyclohexane/ethyl acetate 2:1). ^{1}H NMR (400 MHz, $CDCl_3$, ppm) δ = 7.40–7.26 (m, 5H), 7.15 (s, 1H), 5.32 (s, 2H), 4.84 (s, 2H), 2.56 (s, 3H); ^{13}C NMR (100 MHz, $CDCl_3$, ppm) δ = 174.3, 147.1, 135.2, 129.2 (+, CH, 2C), 128.8 (+, CH), 128.2 (+, CH, 2C), 126.1 (+, CH), 99.9, 57.1 (–, CH_2), 39.0 (–, CH_2), 22.3 (+, CH_3); MS (FAB, Matrix: 3-NBA): *m/z* (%) = 257 (17) $[M+2H]^+$, 256 (82) $[M+H]^+$, 214 (17), 212 (12), 165 (12), 155 (19), 154 (50), 136 (46); HRMS–FAB *(m/z)*: $[M+H]^+$ Calcd for $C_{13}H_14ON_5$, 256.1198; Found 256.1197; IR (ATR, ṽ) = 3383 (vw), 3129 (w), 3091 (vw), 3064 (vw), 3037 (vw), 3007 (vw), 2949 (vw), 2925 (vw), 2167 (vw), 1701 (vs), 1659 (vw), 1635 (vw), 1595 (vw), 1497 (vw), 1469 (s), 1456 (w), 1441 (w), 1424 (w), 1402 (w), 1368 (s), 1317 (vs), 1293 (w), 1268 (w), 1207 (m), 1160 (w), 1142 (w), 1111 (vs), 1054 (w), 1037 (w), 1004 (m), 955 (s), 878 (vs), 815 (m), 764 (w), 724 (w), 697 (vs), 680 (s), 637 (w), 618 (s), 591 (w), 577 (s), 564 (s), 518 (w), 466 (w), 458 (w) cm^{-1}.

Additional information on the chemical synthesis is available *via* Chemotion repository:
https://doi.org/10.14272/reaction/SA-FUHFF-UHFFFADPSC-PGERMRZARQ-UHFFFADPSC-NUHFF-NUHFF-NUHFF-ZZZ.1
Additional information on the analysis of the target compound is available *via* Chemotion repository:
https://doi.org/10.14272/PGERMRZARQFUFX-UHFFFAOYSA-N.2

(6-Benzyl-4,6-dihydro-3*H*-pyrazolo[3,4-*d*][1,2,3]triazin-3-yl)(phenyl)methanone (5b)

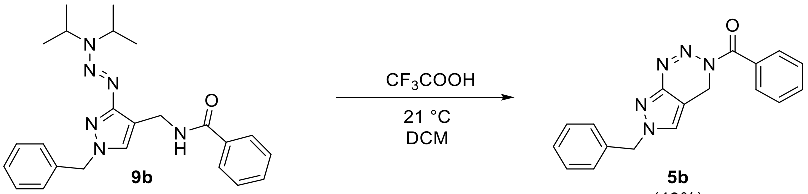

(*E*)-*N*-((1-benzyl-3-(3,3-diisopropyltriaz-1-en-1-yl)-1*H*-pyrazol-4-yl)methyl)benzamide (**9b**, 41.5 mg, 99.2 µmol, 1.00 equiv) was dissolved in 5 mL of dry methylene chloride under nitrogen atmosphere. 2,2,2-Trifluoroacetic acid (33.9 mg, 22.8 µL, 297 µmol, 3.00 equiv) was added and the mixture was stirred at 21 °C for 14 hours. The reaction was diluted with 10 mL of saturated K_2CO_3 solution and the aqueous phase was extracted with 3 × 10 mL of methylene chloride. The combined organic layers were washed with 20 mL of brine and dried over Na_2SO_4. The solvent was removed under reduced pressure to give the crude product. The obtained crude product was

purified *via* flash-chromatography on silica gel using cyclohexane/ethyl acetate 4:1 to 2:1, giving (6-benzyl-4,6-dihydro-3*H*-pyrazolo[3,4-*d*][1,2,3]triazin-3-yl)(phenyl)methanone (**5b**, 13.6 mg, 42.9 µmol, 43% yield) as a colorless solid.

5b: (6-benzyl-4,6-dihydro-3H-pyrazolo[3,4-d][1,2,3]triazin-3-yl)(phenyl)methanone; Formula: $C_{18}H_{15}N_5O$; Exact Mass: 317.1277; Smiles: O=C(N1N=Nc2c(C1)cn(n2)Cc1ccccc1)c1ccccc1; InChIKey: WJOQJVSLWFMJNK-UHFFFAOYSA-N

R_f = 0.31 (cyclohexane/ethyl acetate 2:1). ^{1}H NMR (400 MHz, $CDCl_3$, ppm) δ = 7.79–7.70 (m, 2H), 7.55–7.47 (m, 1H), 7.47–7.28 (m, 7H), 7.21 (s, 1H), 5.34 (s, 2H), 5.01 (s, 2H); ^{13}C NMR (100 MHz, $CDCl_3$, ppm) δ = 172.6, 147.2, 135.2, 133.7, 131.6 (+, CH), 130.3 (+, CH, 2C), 129.2 (+, CH, 2C), 128.8 (+, CH), 128.2 (+, CH, 2C), 128.0 (+, CH, 2C), 126.0 (+, CH), 100.3, 57.2 (–, CH_2), 39.6 (–, CH_2); MS (FAB, Matrix: 3-NBA): *m/z* (%) = 319 (11) $[M+2H]^+$, 318 (48) $[M+H]^+$, 307 (29), 292 (15), 290 (10), 289 (15), 155 (31), 154 (100), 152 (10), 139 (17), 138 (36), 137 (62), 136 (71), 124 (10), 120 (12), 107 (23), 105 (40), 97 (10), 95 (14), 91 (45), 90 (12), 89 (16); HRMS–FAB *(m/z)*: $[M+H]^+$ Calcd for $C_{18}H_{16}ON_5$, 318.1355; Found 318.1356; IR (ATR, ṽ) = 3119 (w), 3030 (w), 2924 (w), 1645 (m), 1598 (w), 1575 (w), 1531 (w), 1479 (m), 1453 (m), 1448 (m), 1408 (w), 1347 (vs), 1324 (m), 1307 (m), 1289 (m), 1213 (m), 1186 (w), 1174 (w), 1157 (m), 1061 (s), 1028 (w), 1001 (m), 950 (w), 912 (s), 870 (vs), 822 (w), 792 (m), 758 (w), 698 (vs), 635 (vs), 618 (m) cm^{-1}.

Additional information on the chemical synthesis is available *via* Chemotion repository:
https://doi.org/10.14272/reaction/SA-FUHFF-UHFFFADPSC-WJOQJVSLWF-UHFFFADPSC-NUHFF-NUHFF-NUHFF-ZZZ
Additional information on the analysis of the target compound is available *via* Chemotion repository:
https://doi.org/10.14272/WJOQJVSLWFMJNK-UHFFFAOYSA-N.1

3-Methyl-1-(6-(4-methylbenzyl)-4,6-dihydro-3*H*-pyrazolo[3,4-*d*][1,2,3]triazin-3-yl)butan-1-one (5c)

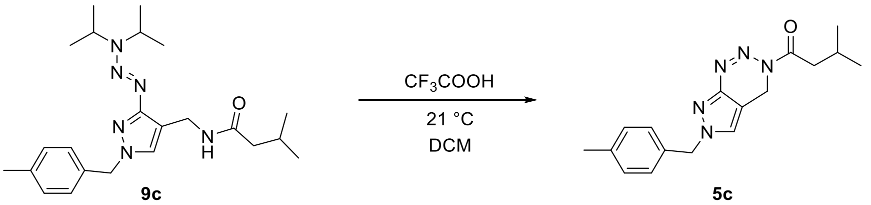

(*E*)-*N*-((3-(3,3-Diisopropyltriaz-1-en-1-yl)-1-(4-methylbenzyl)-1*H*-pyrazol-4-yl)methyl)-3-methylbutanamide (**9c**, 61.7 mg, 150 µmol, 1.00 equiv) was dissolved in 40 mL of dry methylene chloride under nitrogen atmosphere. 2,2,2-Trifluoroacetic acid (34.1 mg, 22.9 µL, 299 µmol, 2.00 equiv) was added and the mixture was stirred at 21 °C for 16 hours. The reaction was diluted with 40 mL of saturated K_2CO_3 solution and the aqueous phase was extracted with 3 × 40 mL of methylene chloride. The combined organic layers were washed with 40 mL of brine and dried over Na_2SO_4. The solvent was removed under reduced pressure to give the crude product.
The obtained crude product was purified *via* flash-chromatography on silica gel using cyclohexane/ethyl acetate 4:1 to 1:1, giving 3-methyl-1-(6-(4-methylbenzyl)-4,6-dihydro-3*H*-pyrazolo[3,4-*d*][1,2,3]triazin-3-yl)butan-1-one (**5c**, 14.1 mg, 45.3 µmol, 30% yield) as an off-colorless solid.

5c: 3-methyl-1-(6-(4-methylbenzyl)-4,6-dihydro-3H-pyrazolo[3,4-d][1,2,3]triazin-3-yl)butan-1-one; Formula: $C_{17}H_{21}N_5O$; Exact Mass: 311.1746; Smiles: CC(CC(=O)N1N=Nc2c(C1)cn(n2)Cc1ccc(cc1)C)C; InChIKey: KEQOQOHNHBUGFW-UHFFFAOYSA-N

R_f = 0.30 (cyclohexane/ethyl acetate 4:1). ^{1}H NMR (400 MHz, $CDCl_3$, ppm) δ = 7.23–7.15 (m, 4H), 7.12 (s, 1H), 5.27 (s, 2H), 4.82 (s, 2H), 2.86 (d, *J* = 7.1 Hz, 2H), 2.35 (s, 3H), 2.25 (thept, *J* = 13.5 Hz, *J* = 6.8 Hz, 1H), 0.99 (d, *J* = 6.6 Hz, 6H); ^{13}C NMR (100MHz, $CDCl_3$, ppm) δ = 176.2, 147.1, 138.8, 132.2, 129.9 (+, CH, 2C), 128.3 (+, CH, 2C), 125.9 (+, CH), 100.0, 56.9 (–, CH_2), 43.0 (–, CH_2), 38.9 (–, CH_2), 25.7 (+, CH), 22.8 (+, CH_3, 2C), 21.3 (+, CH_3); MS (FAB, Matrix: 3-NBA): *m/z* (%) = 313 (19) $[M+2H]^+$, 312 (88) $[M+H]^+$, 311 (6) $[M]^+$, 228 (22), 226 (10), 155 (13), 154 (42), 139 (11), 138 (20), 137 (29), 136 (39), 121 (10), 120 (10), 109 (14), 107 (17), 106 (14), 105 (100), 97 (17), 95 (22), 93 (10), 91 (22), 89 (12); HRMS–FAB *(m/z)*: $[M+H]^+$ Calcd for $C_{17}H_{22}ON_5$,

312.1819; Found 312.1821.IR (ATR, ṽ) = 3125 (w), 3031 (w), 3012 (w), 2956 (w), 2925 (w), 2867 (w), 2730 (vw), 1672 (vs), 1618 (vw), 1596 (w), 1514 (w), 1476 (vs), 1463 (m), 1438 (w), 1431 (w), 1405 (m), 1391 (m), 1377 (vs), 1361 (vs), 1346 (m), 1337 (m), 1317 (vs), 1312 (vs), 1288 (w), 1261 (w), 1228 (w), 1207 (s), 1167 (m), 1128 (w), 1116 (w), 1061 (vs), 1010 (vs), 946 (m), 902 (w), 875 (vs), 867 (vs), 833 (vs), 806 (s), 768 (w), 749 (vs), 725 (m), 717 (m), 680 (s), 645 (m), 637 (m), 619 (vs), 575 (vs), 564 (m), 535 (vs), 509 (w), 470 (s), 433 (w), 414 (w), 402 (w), 394 (w) cm^{-1}.

Additional information on the chemical synthesis is available *via* Chemotion repository:
https://doi.org/10.14272/reaction/SA-FUHFF-UHFFFADPSC-KEQOQOHNHB-UHFFFADPSC-NUHFF-NUHFF-NUHFF-ZZZ
Additional information on the analysis of the target compound is available *via* Chemotion repository:
https://doi.org/10.14272/KEQOQOHNHBUGFW-UHFFFAOYSA-N.1

1-(6-(3,5-Difluorobenzyl)-4,6-dihydro-3*H*-pyrazolo[3,4-*d*][1,2,3]triazin-3-yl)ethan-1-one (5d)

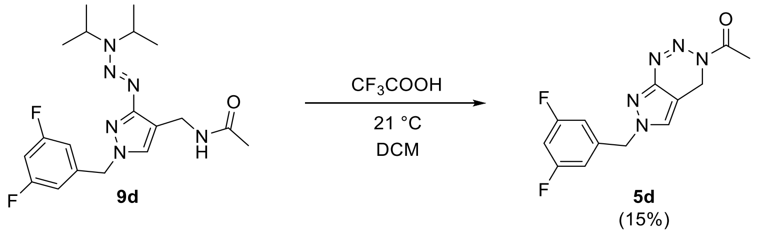

(*E*)-*N*-((1-(3,5-Difluorobenzyl)-3-(3,3-diisopropyltriaz-1-en-1-yl)-1*H*-pyrazol-4-yl)methyl)acetamide (**9d**, 12.9 mg, 32.9 µmol, 1.00 equiv) was dissolved in 5 mL of dry methylene chloride under nitrogen atmosphere. 2,2,2-Trifluoroacetic acid (7.87 mg, 5.29 µL, 69.0 µmol, 2.10 equiv) was added and the mixture was stirred at 21 °C for 14 hours. The reaction was diluted with 10 mL of saturated K_2CO_3 solution and the aqueous phase was extracted with 3 × 10 mL of methylene chloride. The combined organic layers were washed with 20 mL of brine and dried over Na_2SO_4. The solvent was removed under reduced pressure to give the crude product.
The obtained crude product was purified *via* flash-chromatography on silica gel using cyclohexane/ethyl acetate 4:1 to 2:1, giving 1-(6-(3,5-difluorobenzyl)-4,6-dihydro-3*H*-pyrazolo[3,4-*d*][1,2,3]triazin-3-yl)ethan-1-one (**5d**, 1.40 mg, 4.81 µmol, 15% yield) as a colorless solid.

5d: 1-(6-(3,5-difluorobenzyl)-4,6-dihydro-3H-pyrazolo[3,4-d][1,2,3]triazin-3-yl)ethan-1-one; Formula: $C_{13}H_{11}F_2N_5O$; Exact Mass: 291.0932; Smiles: Fc1cc(cc(c1)F)Cn1cc2c(n1)N=NN(C2)C(=O)C; InChIKey: CEFFSZUXPZAWPN-UHFFFAOYSA-N

R_f = 0.27 (cyclohexane/ethyl acetate 2:1). ^{1}H NMR (400 MHz, $CDCl_3$, ppm) δ = 7.22 (s, 1H), 6.84–6.71 (m, 3H), 5.30 (s, 2H), 4.88 (s, 2H), 2.59 (s, 3H); ^{13}C NMR (100 MHz, $CDCl_3$, ppm) δ = 169.5, 163.3 (2C; dd, *J* = 249.5 Hz, *J* = 12.6 Hz), 158.5, 140.6 (t, *J* = 8.9 Hz), 130.7 (+, CH), 110.6 (+, CH, 2C; dd, *J* = 18.6 Hz, *J* = 7.2 Hz), 108.2, 103.5 (+, CH; t, *J* = 25.3 Hz), 55.2 (–, CH_2; t, *J* = 2.3 Hz), 48.4 (+, CH), 45.5 (+, CH), 34.3 (–, CH_2), 23.8 (+, CH_3, 2C), 23.6 (+, CH_3), 19.5 (+, CH_3, 2C); ^{19}F NMR (375 MHz, $CDCl_3$, ppm) δ = –108.08; MS (FAB, Matrix: 3-NBA): *m/z* (%) = 293 (7) $[M+2H]^+$, 292 (37) $[M+H]^+$, 191 (11) $[M]^+$, 163 (10), 159 (12), 155 (21), 154 (43), 137 (30), 136 (40), 125 (20), 123 (36), 111 (48); HRMS–FAB *(m/z)*: $[M+H]^+$ Calcd for $C_{13}H_{12}ON_5F_2$, 292.1010; Found 292.1012; IR (ATR, ṽ) = 3125 (w), 3102 (vw), 3041 (w), 2951 (w), 2922 (w), 2870 (w), 2853 (w), 1701 (vs), 1666 (w), 1622 (m), 1596 (vs), 1538 (vw), 1519 (vw), 1468 (vs), 1446 (s), 1443 (s), 1424 (m), 1402 (w), 1368 (s), 1344 (m), 1315 (vs), 1275 (w), 1258 (w), 1241 (w), 1205 (s), 1159 (w), 1143 (w), 1109 (vs), 1055 (m), 1038 (m), 1004 (vs), 976 (w), 955 (vs), 941 (m), 875 (vs), 856 (vs), 829 (m), 819 (s), 793 (w), 752 (w), 737 (vs), 722 (s), 671 (w), 647 (m), 619 (m) cm^{-1}.

Additional information on the chemical synthesis is available *via* Chemotion repository:
https://doi.org/10.14272/reaction/SA-FUHFF-UHFFFADPSC-CEFFSZUXPZ-UHFFFADPSC-NUHFF-NUHFF-NUHFF-ZZZ
Additional information on the analysis of the target compound is available *via* Chemotion repository:
https://doi.org/10.14272/CEFFSZUXPZAWPN-UHFFFAOYSA-N.1

1-(6-Ethyl-4,6-dihydro-3*H*-pyrazolo[3,4-*d*][1,2,3]triazin-3-yl)ethan-1-one (5e)

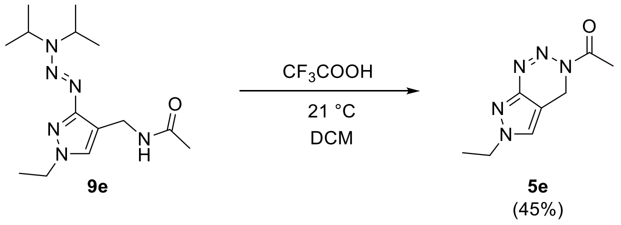

(*E*)-*N*-((3-(3,3-diisopropyltriaz-1-en-1-yl)-1-ethyl-1*H*-pyrazol-4-yl)methyl)acetamide (**9e**, 57.7 mg, 196 µmol, 1.00 equiv) was dissolved in 20 mL of dry methylene chloride under nitrogen atmosphere. 2,2,2-Trifluoroacetic acid (67.0 mg, 45.0 µL, 588 µmol, 3.00 equiv) was added and the mixture was stirred at 21 °C for 16 hours. The reaction was diluted with 20 mL of saturated K_2CO_3 solution and the aqueous phase was extracted with 3 × 20 mL of methylene chloride. The combined organic layers were washed with 20 mL of brine and dried over Na_2SO_4. The solvent was removed under reduced pressure to give the crude product. The obtained crude product was purified *via* flash-chromatography on silica gel using cyclohexane/ethyl acetate 4:1 to 2:1, giving 1-(6-ethyl-4,6-dihydro-3*H*-pyrazolo[3,4-*d*][1,2,3]triazin-3-yl)ethan-1-one (**5e**, 16.9 mg, 87.5 µmol, 45% yield) as colorless solid.

5e: 1-(6-ethyl-4,6-dihydro-3H-pyrazolo[3,4-d][1,2,3]triazin-3-yl)ethan-1-one; Formula: $C_8H_{11}N_5O$; Exact Mass: 193.0964; Smiles: CCn1nc2c(c1)CN(N=N2)C(=O)C; InChIKey: AHUQBMJJIBAFGD-UHFFFAOYSA-N

R_f = 0.05 (cyclohexane/ethyl acetate 4:1). ^{1}H NMR (400 MHz, $CDCl_3$, ppm) δ = 7.21 (s, 1H), 4.86 (s, 2H), 4.19 (q, *J* = 7.3 Hz, 2H), 2.55 (s, 3H), 1.51 (t, *J* = 7.3 Hz, 3H); ^{13}C NMR (100 MHz, $CDCl_3$, ppm) δ = 174.2, 147.0, 125.4 (+, CH), 99.1, 48.1 (–, CH_2), 39.0 (–, CH_2), 22.3 (+, CH_3), 15.5 (+, CH_3); MS (FAB, Matrix: 3-NBA): *m/z* (%) = 195 (13) $[M+2H]^+$, 194 (100) $[M+H]^+$, 193 (6) $[M]^+$, 192 (11) $[M-H]^+$, 154 (8), 152 (26), 150 (18), 136 (12), 133 (33), 124 (24), 123 (10), 109 (14), 107 (11), 97 (11), 96 (10), 95 (16), 93 (10), 91 (16); HRMS–FAB *(m/z)*: $[M+H]^+$ Calcd for $C_8H_{12}ON_5$, 194.1036; Found 194.1036; IR (ATR, ṽ) = 3417 (w), 3363 (w), 3125 (w), 2990 (w), 2955 (w), 2915 (w), 2884 (w), 2854 (w), 2766 (w), 1687 (vs), 1628 (w), 1589 (w), 1574 (w), 1537 (w), 1475 (vs), 1421 (m), 1377 (vs), 1350 (s), 1327 (vs), 1255 (w), 1201 (s), 1173 (s), 1103 (vs), 1050 (s), 1034 (m), 1013 (s), 973 (m), 955 (vs), 875 (vs), 833 (vs), 788 (m), 754 (m), 725 (s), 652 (w), 618 (s) cm^{-1}.

Additional information on the chemical synthesis is available *via* Chemotion repository:
https://doi.org/10.14272/reaction/SA-FUHFF-UHFFFADPSC-AHUQBMJJIB-UHFFFADPSC-NUHFF-NUHFF-NUHFF-ZZZ
Additional information on the analysis of the target compound is available *via* Chemotion repository:
https://doi.org/10.14272/AHUQBMJJIBAFGD-UHFFFAOYSA-N.1

1-(6-Cyclopentyl-4,6-dihydro-3*H*-pyrazolo[3,4-*d*][1,2,3]triazin-3-yl)ethan-1-one (5f)

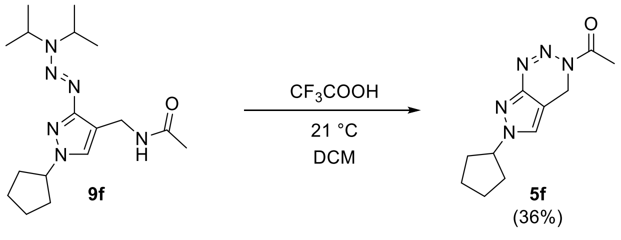

(*E*)-*N*-((1-cyclopentyl-3-(3,3-diisopropyltriaz-1-en-1-yl)-1*H*-pyrazol-4-yl)methyl)acetamide (**9f**, 116 mg, 347 µmol, 1.00 equiv) was dissolved in 35 mL of dry methylene chloride under nitrogen atmosphere. 2,2,2-Trifluoroacetic acid (119 mg, 79.7 µL, 1.04 mmol, 3.00 equiv) was added and the mixture was stirred at 21 °C for 16 hours. The reaction was diluted with 35 mL of saturated K_2CO_3 solution and the aqueous phase was extracted with 3 × 35 mL of methylene chloride. The combined organic layers were washed with 35 mL of brine and dried over Na_2SO_4. The solvent was removed under reduced pressure to give the crude product. The obtained crude product was purified *via* flash-chromatography on silica gel using cyclohexane/ethyl acetate 4:1 to 2:1, giving 1-(6-cyclopentyl-4,6-dihydro-3*H*-pyrazolo[3,4-*d*][1,2,3]triazin-3-yl)ethan-1-one (**5f**, 29.2 mg, 125 µmol, 36% yield) as a light-brown solid.

5f: 1-(6-cyclopentyl-4,6-dihydro-3H-pyrazolo[3,4-d][1,2,3]triazin-3-yl)ethan-1-one; Formula: $C_{11}H_{15}N_5O$; Exact Mass: 233.1277; Smiles: CC(=O)N1N=Nc2c(C1)cn(n2)C1CCCC1; InChIKey: QFPQAGCPTOKOBM-UHFFFAOYSA-N

R_f = 0.21 (cyclohexane/ethyl acetate 4:1). ^{1}H NMR (400 MHz, $CDCl_3$, ppm) δ = 7.23 (s, 1H), 4.84 (s, 2H), 4.63 (p, J = 7.0 Hz, 1H), 2.54 (s, 3H), 2.22–2.10 (m, 2H), 2.09–1.97 (m, 2H), 1.97–1.80 (m, 2H), 1.77–1.63 (m, 2H); ^{13}C NMR (100 MHz, $CDCl_3$, ppm) δ = 174.2, 146.8, 124.8 (+, CH), 98.8, 64.2 (+, CH), 39.0 (–, CH_2), 33.2 (–, CH_2, 2C), 24.3 (–, CH_2, 2C), 22.3 (+, CH_3); MS (FAB, Matrix: 3-NBA): *m/z* (%) = 235 (14) $[M+2H]^+$, 234 (100) $[M+H]^+$, 233 (6) $[M]^+$, 232 (10) $[M-H]^+$, 192 (10), 155 (15), 154 (48), 139 (10), 138 (20), 137 (30), 136 (34), 109 (11), 107 (13), 97 (11), 95 (15), 91 (13); HRMS–FAB *(m/z)*: $[M+H]^+$ Calcd for $C_{11}H_{16}ON_5$, 234.1349; Found 234.1350; IR (ATR, ṽ) = 3370 (vw), 3129 (w), 2970 (w), 2944 (w), 2921 (w), 2873 (w), 1693 (vs), 1656 (w), 1632 (w), 1591 (w), 1538 (vw), 1503 (vw), 1475 (vs), 1456 (w), 1448 (m), 1418 (w), 1374 (vs), 1357 (s), 1332 (vs), 1286 (m), 1248 (w), 1204 (s), 1176 (w), 1159 (w), 1109 (vs), 1052 (m), 1034 (m), 1004 (vs), 953 (vs), 912 (w), 877 (vs), 827 (vs), 741 (m), 722 (s), 623 (m), 594 (w), 578 (vs), 562 (vs), 537 (m), 496 (m), 470 (w), 436 (w), 418 (w), 394 (m) cm^{-1}.

Additional information on the chemical synthesis is available *via* Chemotion repository:
https://doi.org/10.14272/reaction/SA-FUHFF-UHFFFADPSC-QFPQAGCPTO-UHFFFADPSC-NUHFF-NUHFF-NUHFF-ZZZ
Additional information on the analysis of the target compound is available *via* Chemotion repository:
https://doi.org/10.14272/QFPQAGCPTOKOBM-UHFFFAOYSA-N.1

(6-Cyclopentyl-4,6-dihydro-3*H*-pyrazolo[3,4-*d*][1,2,3]triazin-3-yl)(phenyl)methanone (5g)

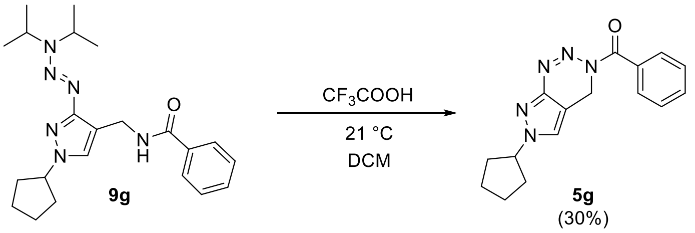

(*E*)-*N*-((1-cyclopentyl-3-(3,3-diisopropyltriaz-1-en-1-yl)-1*H*-pyrazol-4-yl)methyl)benzamide (**9g**, 250 mg, 631 µmol, 1.00 equiv) was dissolved in 50 mL of dry methylene chloride under nitrogen atmosphere. 2,2,2-Trifluoroacetic acid (216 mg, 145 µL, 1.89 mmol, 3.00 equiv) was added and the mixture was stirred at 21 °C for 16 hours. The reaction was diluted with 50 mL of saturated K_2CO_3 solution and the aqueous phase was extracted with 3 × 50 mL of methylene chloride. The combined organic layers were washed with 100 mL of brine and dried over Na_2SO_4. The solvent was removed under reduced pressure to give the crude product. The obtained crude product was purified *via* flash-chromatography on silica gel using cyclohexane/ethyl acetate 4:1 to 1:1, to give (6-cyclopentyl-4,6-dihydro-3*H*-pyrazolo[3,4-*d*][1,2,3]triazin-3-yl)(phenyl)methanone (**5g**, 55.4 mg, 188 µmol, 30% yield). Comment: the reaction was stopped after 16 h, remaining starting material (136 mg) was re-isolated. Based on the consumed starting material, the yield of the reaction is 65%.

5g: (6-cyclopentyl-4,6-dihydro-3H-pyrazolo[3,4-d][1,2,3]triazin-3-yl)(phenyl)methanone; Formula: $C_{16}H_{17}N_5O$; Exact Mass: 295.1433; Smiles: O=C(c1ccccc1)N1N=Nc2c(C1)cn(n2)C1CCCC1; InChIKey: JUJUWEBLNYJZJO-UHFFFAOYSA-N

R_f = 0.47 (cyclohexane/ethyl acetate 2:1). ^{1}H NMR (400 MHz, Chloroform-d [7.27 ppm], ppm) δ = 7.75–7.73 (m, 2H), 7.53–7.48 (m, 1H), 7.45–7.41 (m, 2H), 7.29 (s, 1H), 5.03 (s, 2H), 4.67 (t, J = 7.1 Hz, 1H), 2.22–2.15 (m, 2H), 2.11–2.03 (m, 2H), 1.94–1.86 (m, 2H), 1.77–1.70 (m, 2H); ^{13}C NMR (100 MHz, Chloroform-d [77.0 ppm], ppm) δ = 172.5, 146.9, 133.8, 131.4 (+, CH), 130.2 (+, CH, 2C), 127.9 (+, CH, 2C), 124.6 (+, CH), 99.2, 64.1 (+, CH), 39.5 (–, CH_2), 33.2 (–, CH_2, 2C), 24.2 (–, CH_2, 2C); MS (EI, 70 eV), m/z (%): 296 (61), 270 (67), 154 (62), 149 (33), 137 (43), 136 (58), 105 (100), 95 (38). IR (ATR, ṽ) = 3114 (w), 2953 (w), 2876 (w), 1677 (s), 1663 (vs), 1448 (m), 1341 (vs), 1315 (s), 1207 (m), 1068 (vs), 904 (s), 871 (vs), 826 (vs), 783 (m), 714 (vs), 700 (vs), 693 (vs), 636 (s), 601 (vs) cm^{-1}.

Additional information on the chemical synthesis is available *via* Chemotion repository:

https://doi.org/10.14272/reaction/SA-FUHFF-UHFFFADPSC-JUJUWEBLNY-UHFFFADPSC-NUHFF-NUHFF-NUHFF-ZZZ
Additional information on the analysis of the target compound is available *via* Chemotion repository:
https://doi.org/10.14272/JUJUWEBLNYJZJO-UHFFFAOYSA-N.1

1-(6-Isobutyl-4,6-dihydro-3*H*-pyrazolo[3,4-*d*][1,2,3]triazin-3-yl)ethan-1-one (5h)

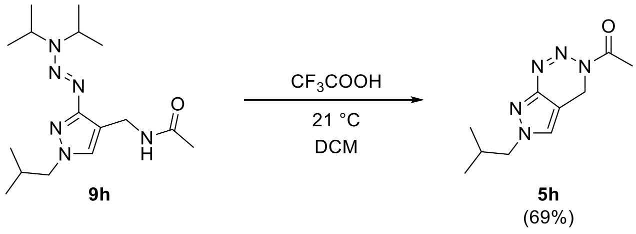

N-((3-(3,3-diisopropyltriaz-1-en-1-yl)-1-isobutyl-1*H*-pyrazol-4-yl)methyl)acetamide (**9h**, 95.4 mg, 296 µmol, 1.00 equiv) was dissolved in 10 mL of dry methylene chloride under nitrogen atmosphere. 2,2,2-Trifluoroacetic acid (101 mg, 68.0 µL, 888 µmol, 3.00 equiv) was added and the mixture was stirred at 21 °C for 14 hours. The reaction was diluted with 10 mL of saturated K_2CO_3 solution and the aqueous phase was extracted with 3 × 10 mL of methylene chloride. The combined organic layers were washed with 20 mL of brine and dried over Na_2SO_4. The solvent was removed under reduced pressure to give the crude product. The obtained crude product was purified *via* flash-chromatography on silica gel using cyclohexane/ethyl acetate 4:1 to 2:1, giving 1-(6-isobutyl-4,6-dihydro-3*H*-pyrazolo[3,4-*d*][1,2,3]triazin-3-yl)ethan-1-one (**5h**, 45.1 mg, 204 µmol, 69% yield) as a colorless solid.

5h: 1-(6-isobutyl-4,6-dihydro-3H-pyrazolo[3,4-d][1,2,3]triazin-3-yl)ethan-1-one; Formula: $C_{10}H_{15}N_5O$; Exact Mass: 221.1277; Smiles: CC(Cn1cc2c(n1)N=NN(C2)C(=O)C)C; InChIKey: WDXQCIDOHPSJHM-UHFFFAOYSA-N

R_f = 0.17 (cyclohexane/ethyl acetate 4:1). ^{1}H NMR (400 MHz, $CDCl_3$, ppm) δ = 7.16 (s, 1H), 4.86 (s, 2H), 3.91 (d, *J* = 7.3 Hz, 2H), 2.55 (s, 3H), 2.23 (hept, *J* = 7.3 Hz, *J* = 6.8 Hz, 1H), 0.91 (d, *J* = 6.7 Hz, 6H); ^{13}C NMR (100 MHz, $CDCl_3$, ppm) δ = 174.2, 147.0, 126.5 (+, CH), 98.9, 60.6 (–, CH_2), 39.0 (–, CH_2), 29.7 (+, CH), 22.3 (+, CH_3), 19.9 (+, CH_3, 2C); MS (FAB, Matrix: 3-NBA): *m/z* (%) = 222 (42) $[M+H]^+$, 196 (82), 194 (72), 192 (22), 178 (26), 167 (34), 166 (24), 165 (26), 153 (25), 152 (57), 151 (38), 150 (22), 137 (100), 136 (35), 135 (24), 133 (59), 121 (25), 109 (23), 108 (22), 107 (24), 97 (23), 96 (36), 95 (34), 93 (24), 91 (29); HRMS–FAB *(m/z)*: $[M+H]^+$ Calcd for $C_{10}H_{16}ON_5$, 222.1355; Found 222.1353; IR (ATR, $\tilde{\nu}$) = 3265 (w), 3057 (w), 2961 (m), 2934 (w), 2873 (w), 1655 (vs), 1541 (vs), 1466 (vs), 1434 (vs), 1405 (s), 1390 (vs), 1370 (vs), 1271 (vs), 1221 (s), 1162 (vs), 1115 (s), 1091 (s), 1020 (s), 992 (s), 946 (m), 926 (m), 894 (m), 849 (s), 819 (s), 769 (s), 732 (vs), 700 (vs), 662 (s), 619 (s), 594 (vs), 540 (s), 489 (s), 435 (m), 409 (m), 398 (m) cm^{-1}.

Additional information on the chemical synthesis is available *via* Chemotion repository:
https://doi.org/10.14272/reaction/SA-FUHFF-UHFFFADPSC-WDXQCIDOHP-UHFFFADPSC-NUHFF-NUHFF-NUHFF-ZZZ
Additional information on the analysis of the target compound is available *via* Chemotion repository:
https://doi.org/10.14272/WDXQCIDOHPSJHM-UHFFFAOYSA-N.1

2-(3-Acetyl-3,4-dihydro-6H-pyrazolo[3,4-*d*][1,2,3]triazin-6-yl)ethyl acetate (5i)

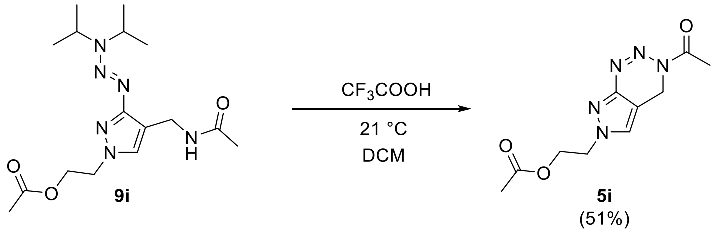

(E)-2-(4-(Acetamidomethyl)-3-(3,3-diisopropyltriaz-1-en-1-yl)-1H-pyrazol-1-yl)ethyl acetate (**9i**, 136 mg, 385 µmol, 1.00 equiv) was dissolved in 40 mL of dry methylene chloride under nitrogen atmosphere. 2,2,2-Trifluoroacetic acid (132 mg, 88.5 µL, 1.16 mmol, 3.00 equiv) was added and the mixture was stirred at 21 °C for 16 hours. The reaction was diluted with 40 mL of saturated K_2CO_3 solution and the aqueous phase was extracted with 3 × 40 mL of methylene chloride. The combined organic layers were washed with 40 mL of brine and dried over Na_2SO_4. The solvent was removed under reduced pressure to give the crude product. The obtained crude product was purified *via* flash-chromatography on silica gel using cyclohexane/ethyl acetate 4:1 to 1:1, giving 2-(3-acetyl-3,4-dihydro-6H-pyrazolo[3,4-*d*][1,2,3]triazin-6-yl)ethyl acetate (**5i**, 49.5 mg, 197 µmol, 51% yield) as a colorless solid.

5i: 2-(3-acetyl-3,4-dihydro-6H-pyrazolo[3,4-d][1,2,3]triazin-6-yl)ethyl acetate; Formula: $C_{10}H_{13}N_5O_3$; Exact Mass: 251.1018; Smiles: CC(=O)OCCn1nc2c(c1)CN(N=N2)C(=O)C; InChIKey: KPRSIIAWIWZXCG-UHFFFAOYSA-N

R_f = 0.17 (cyclohexane/ethyl acetate 4:1). ^{1}H NMR (400 MHz, $CDCl_3$, ppm) δ = 7.25 (s, 1H), 4.86 (s, 2H), 4.45 (t, J = 5.2 Hz, 2H), 4.37 (t, J = 5.4 Hz, 2H), 2.55 (s, 3H), 2.03 (s, 3H); ^{13}C NMR (100 MHz, $CDCl_3$, ppm) δ = 174.2, 170.5, 147.4, 126.9 (+, CH), 99.5, 62.5 (–, CH_2), 51.9 (–, CH_2), 38.9 (–, CH_2), 22.2 (+, CH_3), 20.8 (+, CH_3); MS (FAB, Matrix: 3-NBA): *m/z* (%) = 253 (13) $[M+2H]^+$, 252 (100) $[M+H]^+$, 251 (5) $[M]^+$, 155 (16), 154 (52), 138 (20), 137 (35), 136 (38), 107 (13), 89 (10); HRMS–FAB *(m/z)*: $[M+H]^+$ Calcd for $C_{10}H_{14}O_3N_5$, 252.1091; Found 252.1093; IR (ATR, ṽ) = 3459 (vw), 3376 (vw), 3122 (w), 2992 (vw), 2968 (vw), 2953 (vw), 2925 (vw), 2887 (vw), 2851 (vw), 1871 (vw), 1745 (s), 1734 (vs), 1697 (vs), 1657 (w), 1589 (vw), 1574 (vw), 1476 (s), 1460 (w), 1436 (w), 1422 (w), 1409 (w), 1371 (vs), 1368 (vs), 1347 (w), 1327 (vs), 1313 (s), 1276 (w), 1254 (w), 1234 (vs), 1204 (s), 1188 (m), 1162 (m), 1128 (w), 1102 (vs), 1050 (s), 1038 (vs), 1013 (s), 996 (w), 972 (w), 956 (vs), 936 (s), 868 (vs), 827 (s), 738 (w), 725 (w), 659 (m), 635 (w), 611 (vs) cm^{-1}.

Additional information on the chemical synthesis is available *via* Chemotion repository:
https://doi.org/10.14272/reaction/SA-FUHFF-UHFFFADPSC-KPRSIIAWIW-UHFFFADPSC-NUHFF-NUHFF-NUHFF-ZZZ
Additional information on the analysis of the target compound is available *via* Chemotion repository:
https://doi.org/10.14272/KPRSIIAWIWZXCG-UHFFFAOYSA-N.1

3. Amidation of triazenes 13

Table S1: Synthesis of amides **10** *via* reduction of nitriles **13** to pyrazolo-ortho-methylamines **18** and subsequent conversion with aliphatic anhydrides or chlorides **11**.

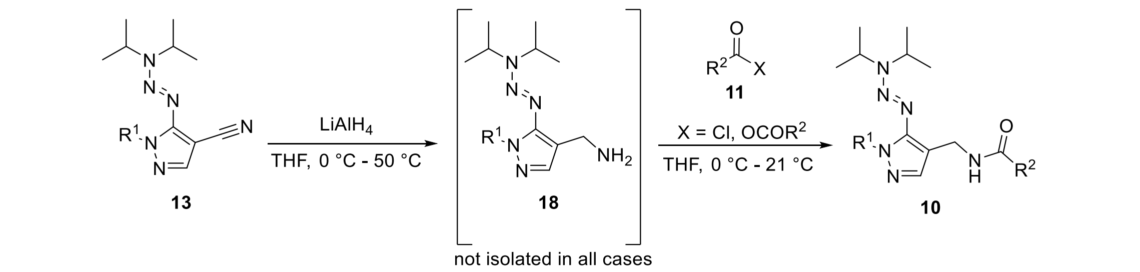

Entry[a]	13	R[1]	18	11	10	R[2]	Yield 10 (%)
1	**13a**	Bn	**18a**	**11d**	**10a**	Me	40 (from 13)
2	**13b**	-CH_2tolyl	**18b**	**11a**	**10c**	Me	77 (from 17)
3	**13f**	^{i}Bu	**18c**	**11a**	**10h**	Me	88 (from 17)
4	**13f**	^{i}Bu	**18c**	**11b**	**10i**	Ph	88 (from 17)

11a = Acetic anhydride, **11b** = Benzoyl anhydride, **11d** = Acetyl chloride

(*E*)-*N*-((1-Benzyl-5-(3,3-diisopropyltriaz-1-en-1-yl)-1*H*-pyrazol-4-yl)methyl)acetamide (10a)

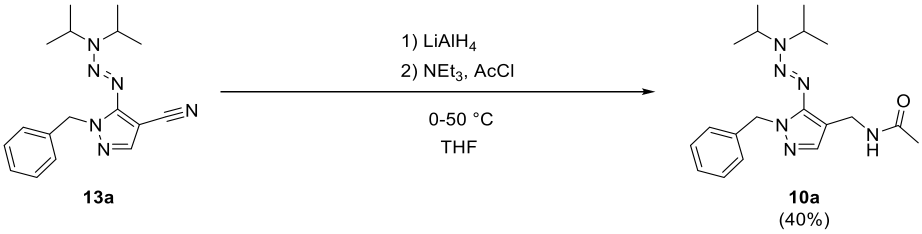

Step1: (*E*)-1-benzyl-5-(3,3-diisopropyltriaz-1-en-1-yl)-1*H*-pyrazole-4-carbonitrile (**13a**, 120 mg, 385 µmol, 1.00 equiv) was dissolved in 40 mL of dry THF under nitrogen atmosphere. The solution was cooled to 0 °C before lithium aluminum hydride (43.9 mg, 1.16 mL, 1.16 mmol, 3.00 equiv) was slowly added. The cooling was removed and the solution was stirred at 21 °C for 14 hours, then additional 5 hours at 50 °C. The solution was cooled to 21 °C and the reaction was quenched with a saturated K/Na-tartrate solution (40 mL). The organic solvent was removed under reduced pressure and the remaining aqueous phase was extracted with methylene chloride (3 × 40 mL). The combined organic layers were washed with brine (100 mL) and dried over Na_2SO_4. The solvent was removed under reduced pressure to give the crude (*E*)-(1-benzyl-3-(3,3-diisopropyltriaz-1-en-1-yl)-1*H*-pyrazol-4-yl)methanamine (**18a**), which was used for the next step without further purification.

Step 2: The crude (*E*)-(1-benzyl-3-(3,3-diisopropyltriaz-1-en-1-yl)-1*H*-pyrazol-4-yl)methanamine (**18a**) was dissolved in 40 mL of dry THF under nitrogen atmosphere. Triethylamine (117 mg, 161 µL, 1.16 mmol, 3.00 equiv) was added and the solution was cooled to 0 °C before acetyl chloride (45.4 mg, 41.1 µL, 578 µmol, 1.50 equiv) was added dropwise. The cooling was removed and the solution was stirred for 14 hours at 21 °C. The reaction was quenched with a saturated K_2CO_3 solution (40 mL) and the organic solvent was removed under reduced pressure. The remaining aqueous phase was extracted with methylene chloride (3 × 40 mL). The combined organic layers were washed with brine (100 mL) and dried over Na_2SO_4. The solvent was removed under reduced pressure to give the crude product. The obtained crude product was purified *via* flash-chromatography on silica gel using methylene chloride/methanol 50:1 to 30:1, giving (*E*)-*N*-((1-benzyl-3-(3,3-diisopropyltriaz-1-en-1-yl)-1*H*-pyrazol-4-yl)methyl)acetamide (**10a**, 55.2 mg, 155 µmol, 40% yield) as a brown oil.

10a: (E)-N-((1-benzyl-5-(3,3-diisopropyltriaz-1-en-1-yl)-1H-pyrazol-4-yl)methyl)acetamide; Formula: $C_{19}H_{28}N_6O$; Exact Mass: 356.2325; Smiles: CC(=O)NCc1cnn(c1/N=N/N(C(C)C)C(C)C)Cc1ccccc1
InChIKey: MPRWSIYLAUTZRJ-GHVJWSGMSA-N

R_f = 0.06 (cyclohexane/ethyl acetate 2:1). ^{1}H NMR (400 MHz, $CDCl_3$, ppm) δ = 7.33–7.23 (m, 5H), 7.20 (s, 1H), 5.96 (t, J = 5.4 Hz, 1H), 5.40 (s, 1H), 5.16 (s, 2H), 4.29 (d, J = 5.3 Hz, 2H), 3.99 (s, 1H), 1.89 (s, 3H), 1.32 (s, 6H), 1.20 (s, 6H); ^{13}C NMR (100 MHz, $CDCl_3$, ppm) δ = 169.4, 157.9, 136.3, 130.2 (+, CH), 128.8 (+, CH, 2C), 128.1 (+, CH), 128.1 (+, CH, 2C), 107.5, 56.2 (–, CH_2), 48.3 (+, CH_3), 45.3 (+, CH_3), 34.4 (–, CH_2), 23.6 (+, CH_3, 2C), 23.5 (+, CH_3), 19.4 (+, CH_3, 2C); MS (FAB, Matrix: 3-NBA): *m/z* (%) = 358 (22) $[M+2H]^+$, 357 (100) $[M+H]^+$, 356 (21) $[M]^+$, 355 (13) $[M-H]^+$, 298 (31), 257 (12), 256 (79), 100 (13), 91 (60); HRMS–FAB *(m/z)*: $[M+H]^+$ Calcd for $C_{19}H_{29}ON_6$, 357.2403; Found 357.2402; IR (ATR, ṽ) = 3285 (w), 3080 (vw), 3065 (vw), 3033 (vw), 2973 (w), 2931 (w), 2871 (vw), 2228 (vw), 1650 (s), 1543 (m), 1497 (w), 1465 (m), 1455 (s), 1419 (vs), 1402 (vs), 1364 (vs), 1341 (s), 1244 (vs), 1224 (vs), 1150 (vs), 1128 (s), 1096 (m), 1033 (s), 1011 (m), 969 (w), 909 (m), 851 (w), 841 (w), 813 (w), 727 (vs), 704 (vs), 643 (m), 630 (w) cm^{-1}.

Additional information on the chemical synthesis is available *via* Chemotion repository:
https://doi.org/10.14272/reaction/SA-FUHFF-UHFFFADPSC-MPRWSIYLAU-UHFFFADPSC-NUHFF-NOHLW-NUHFF-ZZZ
Additional information on the analysis of the target compound is available *via* Chemotion repository:
https://doi.org/10.14272/MPRWSIYLAUTZRJ-GHVJWSGMSA-N.1

(*E*)-(5-(3,3-Diisopropyltriaz-1-en-1-yl)-1-(4-methylbenzyl)-1*H*-pyrazol-4-yl)methanamine (18b)

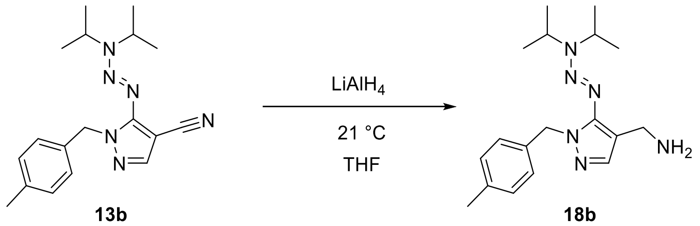

13b 18b 63%

(*E*)-5-(3,3-diisopropyltriaz-1-en-1-yl)-1-(4-methylbenzyl)-1*H*-pyrazole-4-carbonitrile (**13b**, 177 mg, 545 µmol, 1.00 equiv) was dissolved in dry THF under nitrogen-atmosphere. The solution was cooled to 0 °C before lithium aluminum hydride (70.2 mg, 1.85 mL, 1.85 mmol, 3.39 equiv) was added. The cooling was removed and the solution was stirred at 21 °C for 14 hours. The reaction was quenched with K/Na-tartrate solution and the aqueous layer was extracted with DCM. The organic solvent was removed in vacuo and (*E*)-(5-(3,3-diisopropyltriaz-1-en-1-yl)-1-(4-methylbenzyl)-1*H*-pyrazol-4-yl)methanamine (**18b**, 113 mg, 345 µmol, 63% yield) was obtained.

18b: (E)-(5-(3,3-diisopropyltriaz-1-en-1-yl)-1-(4-methylbenzyl)-1*H*-pyrazol-4-yl)methanamine; Formula: $C_{18}H_{28}N_6$; Exact Mass: 328.2375; Smiles: NCc1cnn(c1/N=N/N(C(C)C)C(C)C)Cc1ccc(cc1)C
InChIKey: KQBXWZPQDCVAGS-QURGRASLSA-N

^{1}H NMR (400 MHz, Chloroform-d [7.27 ppm], ppm) δ = 7.20–7.18 (m, 2H), 7.14–7.12 (m, 3H), 5.44 (s, 1H), 5.16 (s, 2H), 3.98 (s, 1H), 3.73 (s, 2H), 2.32 (s, 3H), 2.03 (s, 2H), 1.35 (bs, 6H), 1.22 (bs, 6H). ^{13}C NMR (100 MHz, $CDCl_3$, ppm) δ = 157.6, 137.8, 133.6, 129.5 (+, CH, 2C), 128.7 (+, CH), 128.2 (+, CH, 2C), 113.4, 55.9 (–, CH_2), 48.4 (+, CH), 45.1 (+, CH), 37.2 (–, CH_2), 23.7 (+, CH_3, 2C), 21.2 (+, CH_3), 19.5 (+, CH_3, 2C); MS (FAB, Matrix: 3-NBA): *m/z* (%) = 329 (10) $[M+H]^+$, 328 (4) $[M]^+$, 313 (19), 312 (91), 228 (25), 105 (100); HRMS–FAB *(m/z)*: $[M+H]^+$ Calcd for $C_{18}H_{29}N_6$, 329.2454; Found 329.2453; IR (ATR, ṽ) = 2972 (m), 2928 (m), 2867 (w), 1558 (w), 1516 (w), 1465 (m), 1421 (vs), 1402 (vs), 1380 (m), 1363 (vs), 1237 (vs), 1180 (m), 1150 (vs), 1126 (s), 1098 (m), 1033 (s), 1009 (m), 925 (w), 908 (w), 878 (w), 824 (m), 816 (m), 792 (m), 752 (m), 738 (m), 720 (w), 558 (w), 523 (w), 475 (w) cm^{-1}

Additional information on the chemical synthesis is available *via* Chemotion repository:
https://doi.org/10.14272/reaction/SA-FUHFF-UHFFFADPSC-KQBXWZPQDC-UHFFFADPSC-NUHFF-NBDJD-NUHFF-ZZZ
Additional information on the analysis of the target compound is available *via* Chemotion repository:
https://doi.org/10.14272/KQBXWZPQDCVAGS-QURGRASLSA-N.1
https://dx.doi.org/10.14272/KQBXWZPQDCVAGS-QURGRASLSA-N.2

(*E*)-*N*-((5-(3,3-Diisopropyltriaz-1-en-1-yl)-1-(4-methylbenzyl)-1*H*-pyrazol-4-yl)methyl)acetamide (10c)

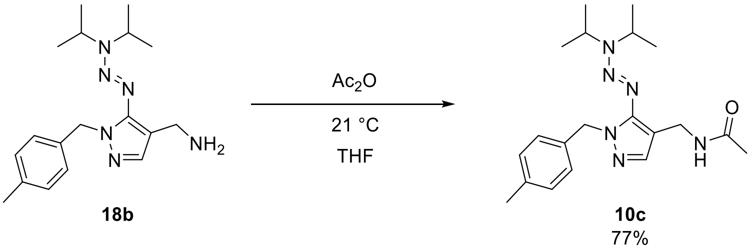

To a solution of (*E*)-(5-(3,3-diisopropyltriaz-1-en-1-yl)-1-(4-methylbenzyl)-1*H*-pyrazol-4-yl)methanamine (**18b**, 90.5 mg, 276 µmol, 1.00 equiv) in THF (2.8 mL), acetic anhydride (56.3 mg, 52.1 µL, 551 µmol, 2.00 equiv) were added at 21 °C. The mixture was stirred at room temperature. After 14 hours, 2 mL of a 2M NaOH solution were added and the organic solvent was removed under reduced pressure. The remaining aqueous layer was extracted with methylene chloride. The combined organic layers were washed with a saturated $NaHCO_3$ solution and brine, dried over $MgSO_4$ and the solvent was removed under reduced pressure. The obtained crude product was purified *via* flash-chromatography on silica gel using methylene chloride/methanol 50:1 to 30:1, to give (*E*)-*N*-((5-(3,3-diisopropyltriaz-1-en-1-yl)-1-(4-methylbenzyl)-1*H*-pyrazol-4-yl)methyl)acetamide (**10c**, 78.8 mg, 213 µmol, 77% yield) as a colorless oil.

10c: (E)-N-((5-(3,3-diisopropyltriaz-1-en-1-yl)-1-(4-methylbenzyl)-1H-pyrazol-4-yl)methyl)acetamide; Formula: $C_{20}H_{30}N_6O$; Exact Mass: 370.2481; Smiles: CC(=O)NCc1cnn(c1/N=N/N(C(C)C)C(C)C)Cc1ccc(cc1)C
InChIKey: AATCYLWHXDDRIG-WCWDXBQESA-N

R_f = 0.06 (cyclohexane/ethyl acetate 2:1). ^{1}H NMR (400 MHz, $CDCl_3$, ppm) δ = 7.20–7.03 (m, 5H), 5.95 (br.t, *J* = 5.4 Hz, 1H), 5.40 (br.s, 1H), 5.10 (s, 2H), 4.27 (d, *J* = 5.4 Hz, 2H), 3.97 (br.s, 1H), 2.29 (s, 3H), 1.87 (s, 3H), 1.31 (br.s, 6H), 1.20 (br.s, 6H); ^{13}C NMR (100 MHz, $CDCl_3$, ppm) δ = 169.2, 157.8, 137.8, 133.2, 129.9 (+, CH), 129.4 (+, CH, 2C), 128.1 (+, CH, 2C), 107.4, 55.9 (–, CH_2), 48.2 (+, CH), 45.2 (+, CH), 34.3 (–, CH_2), 23.6 (+, CH_3, 2C), 23.4 (+, CH_3), 21.1 (+, CH_3), 19.3 (+, CH_3, 2C); MS (FAB, Matrix: 3-NBA): *m/z* (%) = 372 (13) $[M+2H]^+$, 371 (53) $[M+H]^+$, 370 (9) $[M]^+$, 312 (21), 270 (48), 106 (11), 105 (100), 100 (13), 95 (10); HRMS–FAB *(m/z)*: $[M+H]^+$ Calcd for $C_{20}H_{31}ON_6$, 371.2559; Found 371.2558; IR (ATR, ṽ) = 3284 (w), 3085 (vw), 3053 (vw), 2973 (m), 2929 (w), 2870 (w), 1650 (vs), 1541 (m), 1516 (m), 1421 (vs), 1364 (vs), 1244 (vs), 1225 (vs), 1150 (vs), 1128 (s), 1096 (m), 1033 (s), 1010 (m), 921 (w), 911 (m), 796 (s), 751 (w), 728 (vs), 643 (w), 594 (w), 558 (w), 524 (w), 469 (m) cm^{-1}.

Additional information on the chemical synthesis is available *via* Chemotion repository:
https://doi.org/10.14272/reaction/SA-FUHFF-UHFFFADPSC-AATCYLWHXD-UHFFFADPSC-NUHFF-NWIMK-NUHFF-ZZZ
Additional information on the analysis of the target compound is available *via* Chemotion repository:
https://doi.org/10.14272/AATCYLWHXDDRIG-WCWDXBQESA-N.1

(*E*)-(5-(3,3-Diisopropyltriaz-1-en-1-yl)-1-isobutyl-1*H*-pyrazol-4-yl)methanamine (18c)

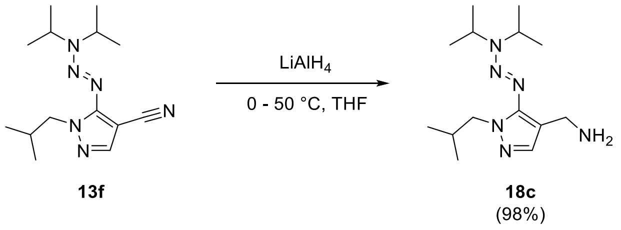

(*E*)-5-(3,3-diisopropyltriaz-1-en-1-yl)-1-isobutyl-1*H*-pyrazole-4-carbonitrile (**13f**, 245 mg, 885 µmol, 1.00 equiv) was dissolved in 26 mL of dry THF under nitrogen-atmosphere. The solution was cooled to 0 °C before lithium aluminum hydride (101 mg, 2.65 mL, 2.65 mmol, 3.00 equiv) was added. The cooling was removed and the solution was stirred at 21 °C for 14 hours, then additional 5 hours at 50 °C. The reaction was quenched with K/Na-tartrate solution, THF was removed under reduced pressure and the remaining aqueous layer was extracted with

CH_2Cl_2 (3 × 25 mL). The combined organic layers were washed with brine and dried over $MgSO_4$. The solvent was removed under reduced pressure to give (*E*)-(5-(3,3-diisopropyltriaz-1-en-1-yl)-1-isobutyl-1*H*-pyrazol-4-yl)methanamine (**18c**, 243 mg, 866 µmol, 98% yield) as crude product which was used without further purification.

18c: (E)-(5-(3,3-diisopropyltriaz-1-en-1-yl)-1-isobutyl-1H-pyrazol-4-yl)methanamine; Formula: $C_{14}H_{28}N_6$; Exact Mass: 280.2375; Smiles: NCc1cnn(c1/N=N/N(C(C)C)C(C)C)CC(C)C
InChIKey: SGMNZUYLCBPNEE-ISLYRVAYSA-N

^{1}H NMR (400 MHz, $CDCl_3$, ppm) δ = 7.30 (s, 1H), 5.08 (br.s, 1H), 4.11–3.91 (m, 3H), 3.70 (s, 2H), 2.20 (hept, *J* = 6.7 Hz, 1H), 1.64 (br.s, 2H), 1.35 (br.s, 6H), 1.26 (br.s, 6H), 0.88 (d, *J* = 6.7 Hz, 6H); ^{13}C NMR (100 MHz, $CDCl_3$, ppm) δ = 146.9, 137.7 (+, CH), 109.9, 55.7 (–, CH_2), 49.5 (+, CH), 46.6 (+, CH), 37.4 (–, CH_2), 29.7 (+, CH), 23.6 (+, CH_3, 2C), 20.3 (+, CH_3, 2C), 19.2 (+, CH_3, 2C); MS (FAB, Matrix: 3-NBA): *m/z* (%) = 282 (13) $[M+2H]^+$, 281 (69) $[M+H]^+$, 280 (6) $[M]^+$, 265 (17), 264 (100), 115 (13), 109 (18), 107 (13), 105 (11), 97 (20), 96 (17), 95 (32), 93 (17), 91 (21); HRMS–FAB *(m/z)*: $[M+H]^+$ Calcd for $C_{14}H_{29}N_6$, 281.2454; Found 281.2453; IR (ATR, ṽ) = 3370 (vw), 2966 (m), 2931 (m), 2870 (w), 1632 (w), 1585 (w), 1550 (w), 1465 (s), 1411 (vs), 1363 (vs), 1315 (m), 1298 (m), 1259 (s), 1238 (vs), 1225 (vs), 1159 (vs), 1125 (s), 1096 (s), 1060 (m), 1018 (vs), 945 (m), 924 (m), 907 (s), 892 (s), 877 (s), 846 (s), 820 (m), 785 (m), 735 (m), 717 (m), 694 (m), 656 (m) cm^{-1}.

Additional information on the chemical synthesis is available *via* Chemotion repository:
https://doi.org/10.14272/reaction/SA-FUHFF-UHFFFADPSC-SGMNZUYLCB-UHFFFADPSC-NUHFF-NBSLK-NUHFF-ZZZ
Additional information on the analysis of the target compound is available *via* Chemotion repository:
https://doi.org/10.14272/SGMNZUYLCBPNEE-ISLYRVAYSA-N.1

(*E*)-*N*-((5-(3,3-Diisopropyltriaz-1-en-1-yl)-1-isobutyl-1*H*-pyrazol-4-yl)methyl)acetamide (10h)

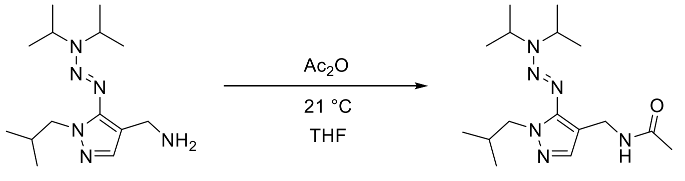

18c → **10h** (88%)

To a solution of (*E*)-(5-(3,3-diisopropyltriaz-1-en-1-yl)-1-isobutyl-1*H*-pyrazol-4-yl)methanamine (**18c**, 117 mg, 418 µmol, 1.00 equiv) in 4.45 mL of THF, acetic anhydride (85.3 mg, 79.0 µL, 836 µmol, 2.00 equiv) was added at 21 °C. The mixture was stirred at 21 °C. After 14 hours, 2 mL of a 2 M NaOH solution were added and the organic solvent was removed under reduced pressure. The remaining aqueous layer was extracted with methylene chloride (3 x 10 mL). The combined organic layers were washed with a saturated $NaHCO_3$ solution and brine, dried over $MgSO_4$ and the solvent was removed under reduced pressure. The obtained crude product was purified *via* flash-chromatography on silica gel using methylene chloride/methanol 50:1 to 20:1, giving (*E*)-*N*-((5-(3,3-diisopropyltriaz-1-en-1-yl)-1-isobutyl-1*H*-pyrazol-4-yl)methyl)acetamide (**10h**, 119 mg, 369 µmol, 88% yield).

10h: (E)-N-((5-(3,3-diisopropyltriaz-1-en-1-yl)-1-isobutyl-1H-pyrazol-4-yl)methyl)acetamide; Formula: $C_{16}H_{30}N_6O$; Exact Mass: 322.2481; Smiles: CC(Cn1ncc(c1/N=N/N(C(C)C)C(C)C)CNC(=O)C)C
InChIKey: NFQAOFXQAVIJOU-FMQUCBEESA-N

R_f = 0.16 (cyclohexane/ethyl acetate 2:1). ^{1}H NMR (400 MHz, $CDCl_3$, ppm) δ = 7.35 (s, 1H), 5.68 (br.s, 1H), 5.18–4.99 (m, 1H), 4.33 (d, *J* = 5.0 Hz, 2H), 4.07–4.03 (m, 1H), 4.01 (d, *J* = 7.3 Hz, 2H), 2.22 (sept, *J* = 6.9 Hz, 1H), 1.95 (s, 3H), 1.35 (br.d, *J* = 6.6 Hz, 6H), 1.27 (br.d, *J* = 6.8 Hz, 6H), 0.90 (d, *J* = 6.7 Hz, 6H); ^{13}C NMR (100 MHz, $CDCl_3$, ppm) δ = 169.3, 147.8, 138.7 (+, CH), 103.3, 55.8 (–, CH_2), 49.7 (+, CH), 46.9 (+, CH), 35.0 (–, CH_2), 29.7 (+, CH), 23.6 (+, CH_3), 23.5 (+, CH_3, 2C), 20.3 (+, CH_3, 2C), 19.2 (+, CH_3, 2C); MS (FAB, Matrix: 3-NBA): *m/z* (%) = 324 (18) $[M+2H]^+$, 323 (100) $[M+H]^+$, 322 (12) $[M]^+$, 265 (10), 264 (54), 222 (20), 209 (19), 167 (13), 100 (13), 97 (10), 96 (12), 95 (11), 91 (11); HRMS–FAB *(m/z)*: $[M+H]^+$ Calcd for $C_{16}H_{31}ON_6$, 323.2559; Found 323.2559; IR (ATR, ṽ) = 3272 (w), 3078 (vw), 2969 (m), 2931 (w), 2871 (w), 1649 (s), 1541 (s), 1466 (s), 1409 (vs), 1381 (s), 1363 (vs), 1313 (m), 1259 (s), 1239 (vs), 1222 (vs), 1159 (vs), 1119 (s), 1096 (s), 1021 (vs), 945 (m), 925 (m), 911 (m), 891 (m), 846 (m), 820 (w), 789 (m), 731 (m), 694 (m), 670 (m), 647 (m), 588 (m), 557 (s), 534 (m), 469 (m), 452 (w), 426 (m), 394 (w), 387 (w) cm^{-1}.

Additional information on the chemical synthesis is available *via* Chemotion repository:
https://doi.org/10.14272/reaction/SA-FUHFF-UHFFFADPSC-NFQAOFXQAV-UHFFFADPSC-NUHFF-NGHTP-NUHFF-ZZZ
Additional information on the analysis of the target compound is available *via* Chemotion repository:
https://doi.org/10.14272/NFQAOFXQAVIJOU-FMQUCBEESA-N.1

(*E*)-*N*-((5-(3,3-Diisopropyltriaz-1-en-1-yl)-1-isobutyl-1*H*-pyrazol-4-yl)methyl)benzamide (10i)

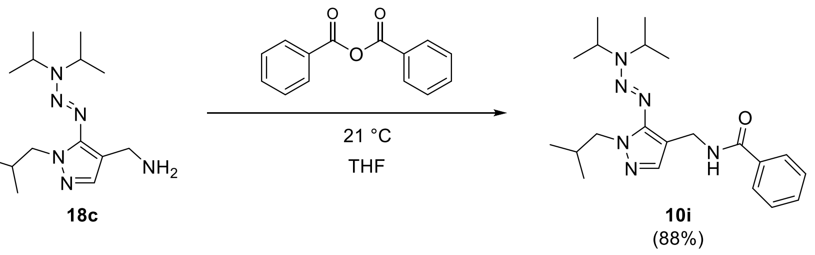

To a solution of (*E*)-(5-(3,3-diisopropyltriaz-1-en-1-yl)-1-isobutyl-1*H*-pyrazol-4-yl)methanamine (**18c**, 106 mg, 377 µmol, 1.00 equiv) in 5 mL of THF, benzoyl benzoate (170 mg, 142 µL, 753 µmol, 2.00 equiv) was added at 21 °C. The mixture was stirred at 21 °C. After 14 hours, 2 mL of a 2 M NaOH solution were added and the organic solvent was removed under reduced pressure. The remaining aqueous layer was extracted with methylene chloride (3 × 10 mL). The combined organic layers were washed with a saturated $NaHCO_3$ solution and brine. It was then dried over $MgSO_4$ and the solvent was removed under reduced pressure. The obtained crude product was purified *via* flash-chromatography on silica gel using cyclohexane/ethyl acetate 10:1 to 2:1, giving (*E*)-*N*-((5-(3,3-diisopropyltriaz-1-en-1-yl)-1-isobutyl-1*H*-pyrazol-4-yl)methyl)benzamide (**10i**, 128 mg, 332 µmol, 88% yield) as pale-yellow fluffy crystals.

10i: (E)-N-((5-(3,3-diisopropyltriaz-1-en-1-yl)-1-isobutyl-1H-pyrazol-4-yl)methyl)benzamide; Formula: $C_{21}H_{32}N_6O$; Exact Mass: 384.2638; Smiles: CC(Cn1ncc(c1/N=N/N(C(C)C)C(C)C)CNC(=O)c1ccccc1)C
InChIKey: MPFTVRYGHBGKIC-OCOZRVBESA-N

R_f = 0.19 (cyclohexane/ethyl acetate 4:1). ^{1}H NMR (400 MHz, $CDCl_3$, ppm) δ = 7.76–7.68 (m, 2H), 7.51–7.33 (m, 4H), 6.28 (br.s, 1H), 5.15–4.96 (m, 1H), 4.54 (d, *J* = 4.8 Hz, 2H), 4.06–3.93 (m, 3H), 2.25 (sept, *J* = 13.8 Hz, *J* = 7.0 Hz, 1H), 1.28 (br.d, *J* = 6.7 Hz, 12H), 0.91 (d, *J* = 6.7 Hz, 6H); ^{13}C NMR (100 MHz, $CDCl_3$, ppm) δ = 167.1, 147.9, 138.9 (+, CH), 135.1, 131.3 (+, CH), 128.6 (+, CH, 2C), 127.0 (+, CH, 2C), 103.2, 55.8 (–, CH_2), 50.0 (+, CH), 46.9 (+, CH), 35.5 (–, CH_2), 29.7 (+, CH), 23.4 (+, CH_3, 2C), 20.3 (+, CH_3, 2C), 19.2 (+, CH_3, 2C); MS (FAB, Matrix: 3-NBA): *m/z* (%) = 386 (26) $[M+2H]^+$, 385 (100) $[M+H]^+$, 384 (12) $[M]^+$, 383 (11), 284 (26), 271 (29), 265 (12), 264 (65), 105 (51), 97 (10), 95 (11); HRMS–FAB *(m/z)*: $[M+H]^+$ Calcd for $C_{21}H_{33}ON_6$, 385.2716; Found 385.2715; IR (ATR, ṽ) = 3315 (w), 3071 (vw), 3053 (vw), 2968 (w), 2928 (w), 2868 (w), 1635 (vs), 1601 (w), 1577 (w), 1543 (vs), 1492 (m), 1466 (m), 1446 (w), 1418 (vs), 1405 (vs), 1380 (s), 1363 (vs), 1324 (m), 1299 (vs), 1252 (vs), 1238 (vs), 1224 (vs), 1193 (w), 1153 (s), 1125 (s), 1109 (s), 1095 (m), 1081 (w), 1052 (w), 1017 (vs), 1001 (m), 980 (m), 945 (w), 928 (w), 912 (w), 892 (w), 882 (w), 853 (w), 822 (w), 809 (m), 781 (s), 739 (w), 715 (s), 697 (vs), 669 (m), 646 (s), 616 (m) cm^{-1}.

Additional information on the chemical synthesis is available *via* Chemotion repository:
https://doi.org/10.14272/reaction/SA-FUHFF-UHFFFADPSC-MPFTVRYGHB-UHFFFADPSC-NUHFF-NWMXW-NUHFF-ZZZ
Additional information on the analysis of the target compound is available *via* Chemotion repository:
https://doi.org/10.14272/MPFTVRYGHBGKIC-OCOZRVBESA-N.1

4. Crystal structure determinations of compounds 12h and 13c

The single-crystal X-ray diffraction studies were carried out on a Bruker D8 Venture diffractometer with PhotonII detector at 123(2) K using CuKα radiation (λ = 1.54178 Å). Dual space methods (SHELXT) [G. M. Sheldrick, *Acta Crystallogr.* 2015, **A71**, 3-8] were used for structure solution and refinement was carried out using SHELXL-2014 (full-matrix least-squares on F^2) [G. M. Sheldrick, *Acta Crystallogr.* 2015, **C71**, 3-8]. Hydrogen atoms were localized by difference electron density determination and refined using a riding model. Semi-empirical absorption corrections were applied. For **12h** an extinction correction was applied.

12h: colourless crystals, $C_{17}H_{21}BrN_6$, M_r = 389.31, crystal size 0.18 × 0.108× 0.04 mm, monoclinic, space group *P*2$_1$/c (No. 14), a = 13.0525(10) Å, b = 14.3306(11) Å, c = 10.0264(8) Å, β = 97.128(2)°, V = 1860.9(3) Å^3, Z = 4, ρ = 1.390 Mg/m^{-3}, μ(Cu-K$_\alpha$) = 3.09 mm^{-1}, F(000) = 800, $2\theta_{max}$ = 144.4°, 33389 reflections, of which 3665 were independent (R_{int} = 0.024), 218 parameters, R_1 = 0.022 (for 3621 I > 2σ(I)), wR_2 = 0.056 (all data), S = 1.03, largest diff. peak / hole = 0.30 / -0.28 e Å^{-3}.

13c: colourless crystals, $C_{17}H_{20}F_2N_6$, M_r = 346.39, crystal size 0.20 × 0.16 × 0.08 mm, monoclinic, space group *P*2$_1$/c (No. 14), a = 9.7201(3) Å, b = 11.9699(4) Å, c = 15.3002(5) Å, β = 100.614(1)°, V = 1749.70(10) Å^3, Z = 4, ρ = 1.315 Mg/m^{-3}, μ(Cu-K$_\alpha$) = 0.82 mm^{-1}, F(000) = 728, $2\theta_{max}$ = 144.6°, 19144 reflections, of which 3428 were independent (R_{int} = 0.023), 226 parameters, R_1 = 0.037 (for 3330 I > 2σ(I)), wR_2 = 0.095 (all data), S = 1.05, largest diff. peak / hole = 0.53 / -0.24 e Å^{-3}.

CCDC 2054633 (**12h**), and 2054634 (**13c**) contain the supplementary crystallographic data for this paper. These data can be obtained free of charge from The Cambridge Crystallographic Data Centre *via* www.ccdc.cam.ac.uk/data_request/cif.

Table X-Ray-S1: Crystallographic and refinement data.

Compound	**12h**	**13c**
Emp. formula	$C_{17}H_{21}BrN_6$	$C_{17}H_{20}F_2N_6$
Molar mass	389.31	346.39
Temperature/K	123	123
Crystal system	monoclinic	monoclinic
Space group	*P*2$_1$/*c*	*P*2$_1$/*c*
a/Å	13.0525(10)	9.7201(3)
b/Å	14.3306(11)	11.9699(4)
c/Å	10.0264(8)	15.3002(5)
α/°	90	90
β/°	97.128(2)	100.614(1)
γ/°	90	90
Volume/Å^3	1860.9(3)	1749.70(10)
Z	4	4
ρ_{calc}g/cm^3	1.390	1.3125
μ/mm^{-1}	3.09	0.82
F(000)	800	2312
Radiation	CuKα (λ = 1.54178)	CuKα (λ = 1.54178)
2Θ range /°	6.8–144.4	9.2–144.6
Refl. collected	33389	19114
Independent refl.	3665 [R_{int} = 0.024]	3428 [R_{int} = 0.023]
Ind. refl. I ≥ 2σ(I)	3621	3330
Data/rest./param.	3665/0/218	3428/0/226
Gof	1.03	1.05
R indexes [I ≥ 2σ(I)]	R_1 = 0.022, wR_2 = 0.056	R_1 = 0.037, wR_2 = 0.094
R indexes [all data]	R_1 = 0.022, wR_2 = 0.056	R_1 = 0.038, wR_2 = 0.095
Diff. peak/hole /eÅ^{-3}	0.30/–0.28	0.53/–0.24
CCDC no.	2054633	2054634

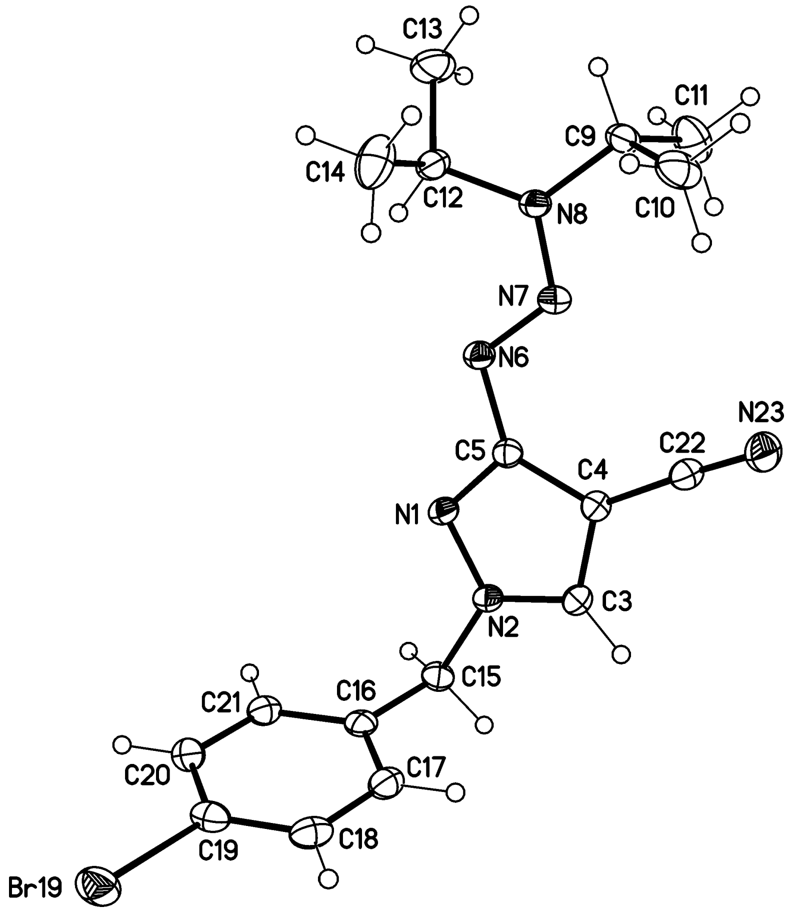

Figure S1. Molecular structure of **12h** (displacement parameters are drawn at 50 % probability level).

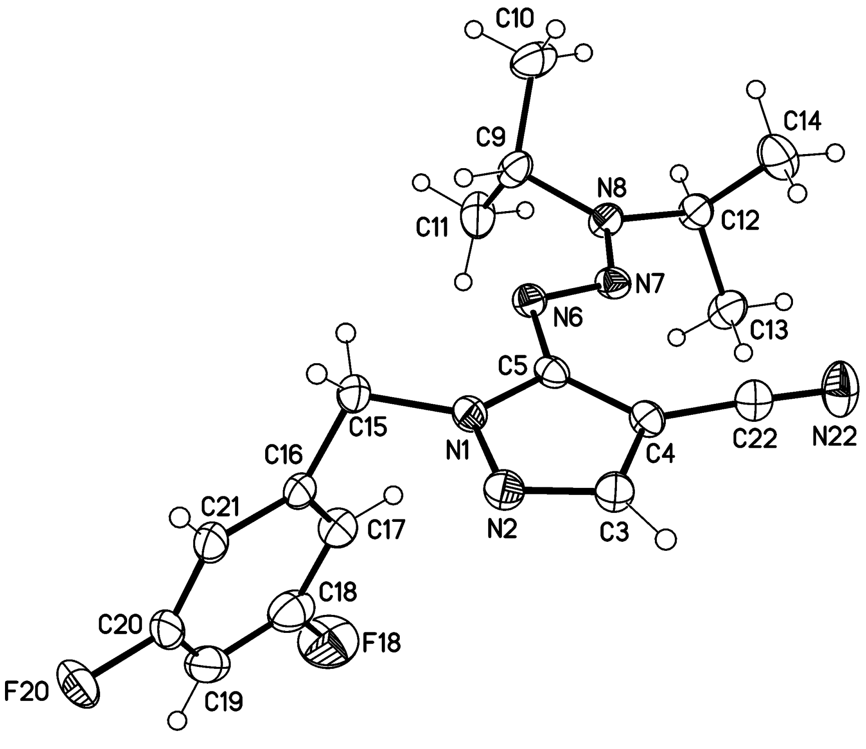

Figure S2. Molecular structure of **13c** (displacement parameters are drawn at 50 % probability level).

5. Biological assays

Biological assays were carried out under sterile conditions using the sterile benches LaminAir® HB2448 (HERAEUS GmbH), Laminar-Flow Werkbank (BDK LUFT- UND REINRAUMTECHNIK GmbH) or Clean Air Technik (CLEAN AIR). Cells were incubated at 37 °C, 5% CO_2, and 85% humidity using a BINDER Ex-Demo Units Incubator.

Cell culture

Human epithelial cervix carcinoma (HeLa) cells were cultivated in *Dulbecco´s Modified Eagle Medium-high glucose* (DMEM, GIBCO™) supplemented with 10% fetal calf serum (FCS) and 1% penicillin/streptomycin (P/S). At 80% confluency, the medium was removed, the cells were washed with *Dulbecco's Phosphate Buffered Saline* (DPBS, GIBCO™) and treated with trypsin/EDTA (GIBCO™) to passage the cells. The detachment process was stopped with culture medium (DMEM + 10% FBS + 1% P/S) and the cells were seeded according to the desired cell number/area.

MTT assay[1]

In a 96-well plate (CORNING® Costar® 3596 96-Well Cell Culture Cluster), 1.0×10^4 HeLa cells per well of a 96-well plate were cultivated for 16 h. Stock solutions of the single compounds in dimethylsulfoxide were diluted to 0.50 µM, 1.00 µM, 5.00 µM, 10.0 µM, 25.0 µM, and 50.0 µM with cell culture medium (DMEM + 10% FBS + 1% P/S) to ensure a final concentration of DMSO below 0.5%. Then, 100 µL of the prepared DMEM-diluted compounds were added to each well and the cells were incubated for 72 h. All experiments were performed in triplicates. Cells that were solely treated with the culture medium containing 0.5% of DMSO served as a negative control. After incubation, the positive control (cells in DMEM plus 0.5% DMSO) was treated with 1% Triton-X. To determine the viability of the cells 20 µL of a freshly prepared solution of 3-(4,5-dimethylthiazol-2-yl)-2,5-diphenyltetrazolium bromide (MTT, 2.73 mg/mL, SIGMA-ALDRICH®) in culture medium were added to each well and incubated at 37 °C for 4 h. The reaction was stopped using 100 µL of *Solubilization Solution / Stop Mix* (CellTiter 96® Non-Radioactive Cell Proliferation Assay, PROMEGA). After 72 h, the absorption (A) of every well at 595 nm was monitored using a 96-well plate reader (Ultra Microplate Reader ELx808, BIOTECH INSTRUMENTS INC.). Graphic evaluation using a dose response fitting was carried out with the help of the software OriginPro® 2021. IC_{50} values were calculated with Excel.

Table S1. Selected pyrazolo[1,2,3]triazines **5** and intermediates **9**, **12**, **13** and **16** and their calculated IC_{50} values after treatment of HeLa cells with different concentrations of the respective compounds.

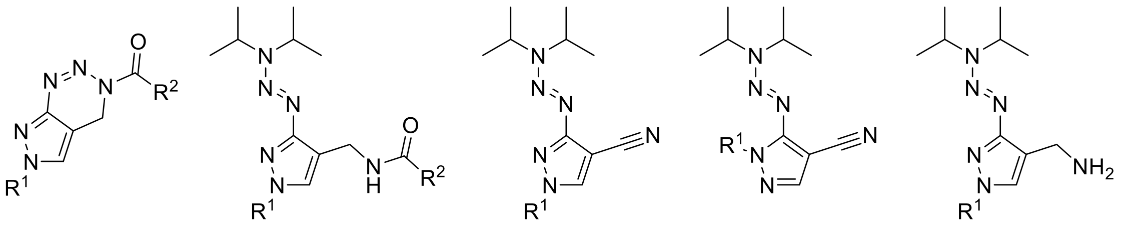

5 **9** **12** **13** **16**

Entry	Compound	R^1	R^2	IC_{50} [µM]
1	**5a**	Bn	Me	>50
2	**5d**	3,5-Difluorobenzyl	Me	>50
3	**5e**	Ethyl	Me	>50
4	**5f**	Cyclopentyl	Me	>50
5	**5h**	^{i}Butyl	Me	>50
6	**9a**	Bn	Me	>50
7	**9b**	Bn	Ph	17

8	**9c**	Tolyl-CH_2	^{i}Butyl	>50
9	**9d**	3,5-Difluorobenzyl	Me	>50
10	**9e**	Ethyl	Me	>50
11	**9f**	Cyclopentyl	Me	>50
12	**9g**	Cyclopentyl	Me	>50
13	**9h**	^{i}Butyl	Ph	>50
14	**9i**	$MeCO_2(CH_2)_2$	Me	>50
15	**12a**	Bn	–	>50
16	**12b**	Tolyl-CH_2	–	41
17	**12c**	3,5-Difluorobenzyl	–	49
18	**12d**	Ethyl	–	>50
19	**12e**	Cyclopentyl	–	>50
20	**12f**	^{i}Butyl	–	>50
21	**12g**	$EtCO_2CH_2$	–	>50
22	**12h**	4-Bromobenzyl	–	>50
23	**13a**	Bn	–	47
24	**13b**	Tolyl-CH_2	–	45
25	**13c**	3,5-Difluorobenzyl	–	>50
26	**13d**	Ethyl	–	20
27	**13e**	Cyclopentyl	–	>50
28	**13f**	^{i}Butyl	–	46
29	**13g**	$EtCO_2CH_2$	–	21
30	**13h**	4-Bromobenzyl	–	29
31	**16f**	^{i}Butyl	–	>50

Table S2: Graphical representation of the MTT assays conducted for selected compounds and their respective calculated IC_{50} values.

Compound	IC_{50} [µM]	Graph
**5a**	>50	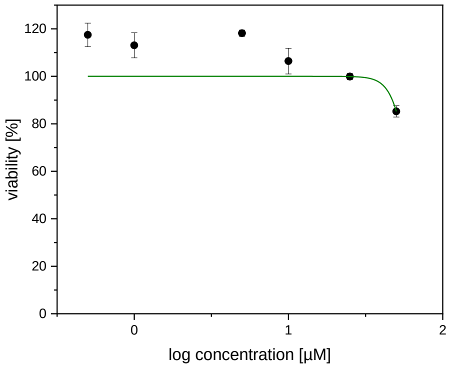

**5d**	>50	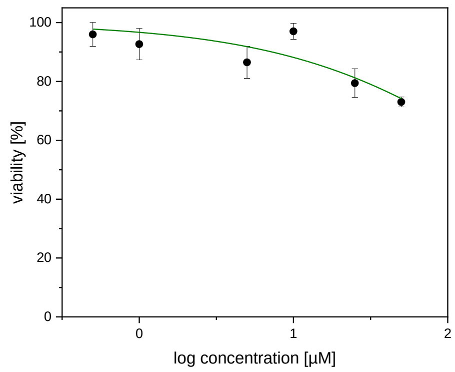
**5e**	>50	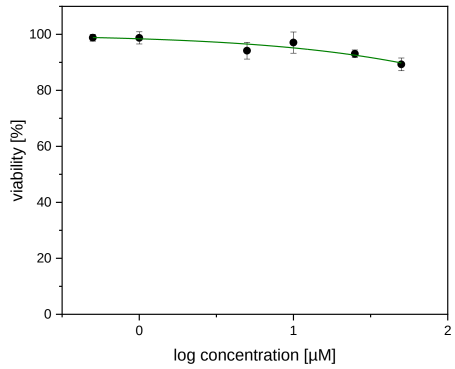
**5f**	>50	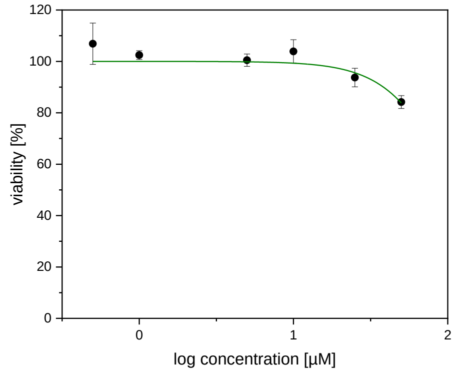
**5h**	>50	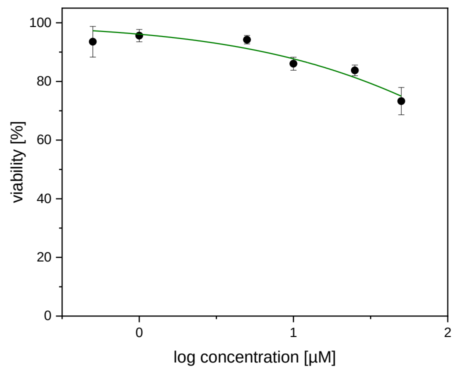

**9a**	>50	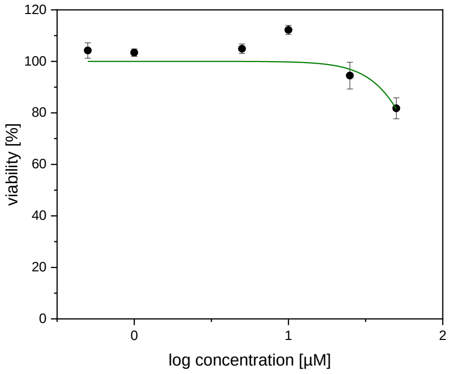
**9b**	17	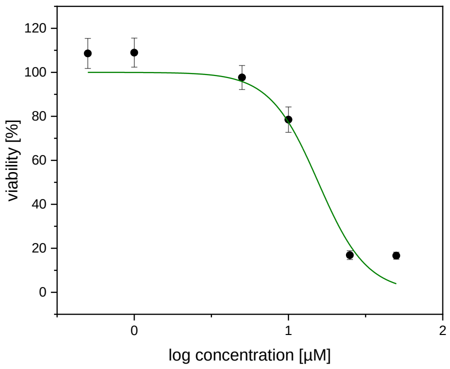
**9c**	>50	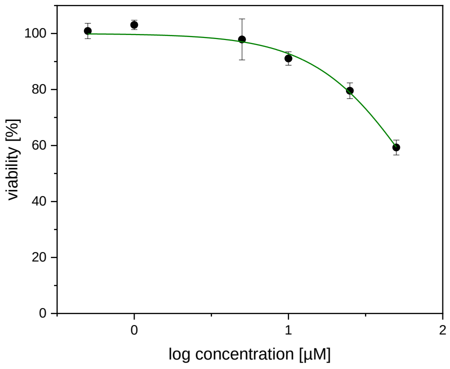
**9d**	>50	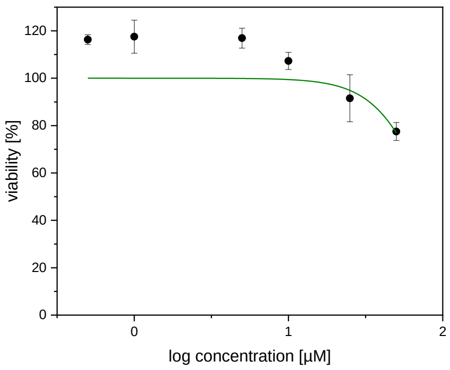

9e >50

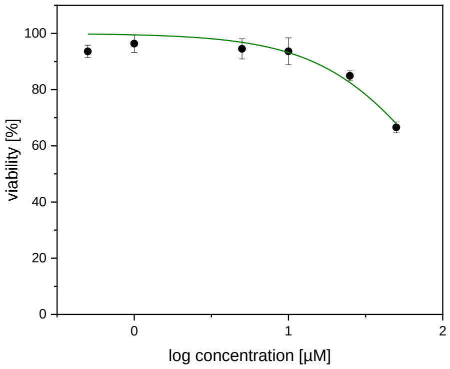

9f >50

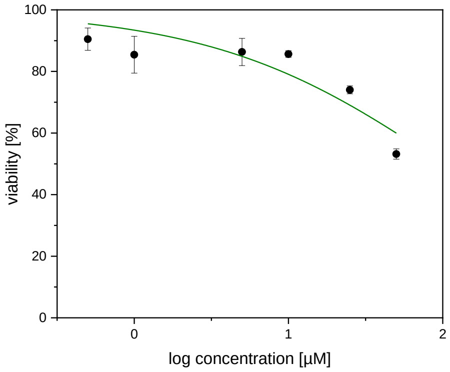

9g >50

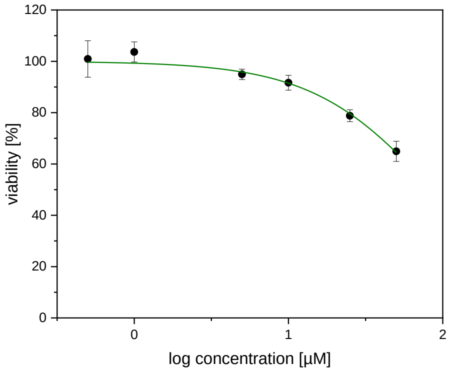

9h >50

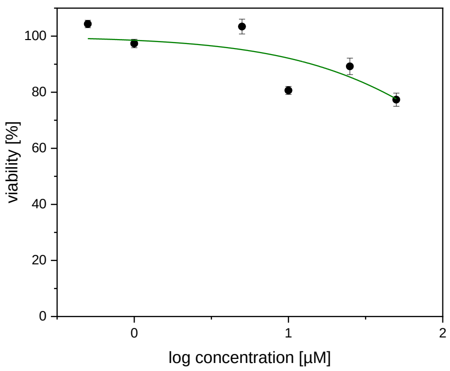

9i >50

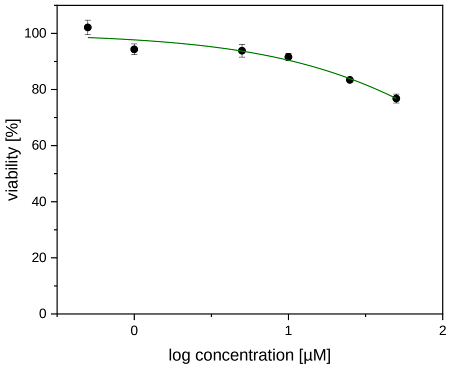

10h >50

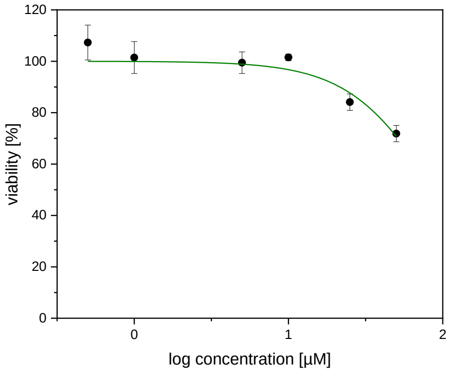

12a >50

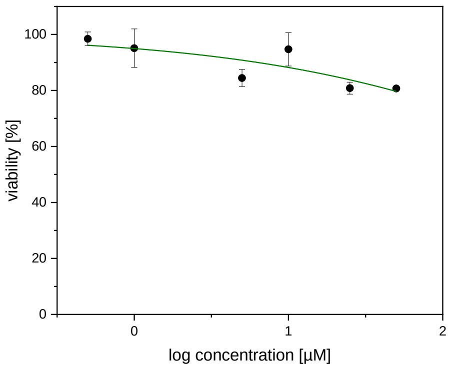

12b 41

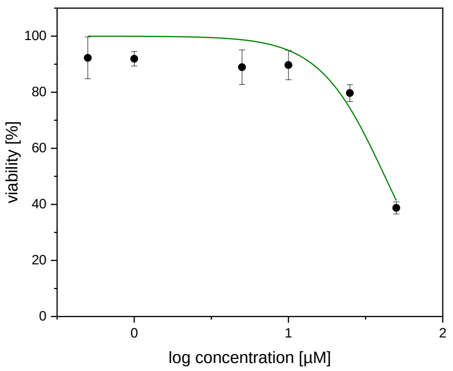

12c 49

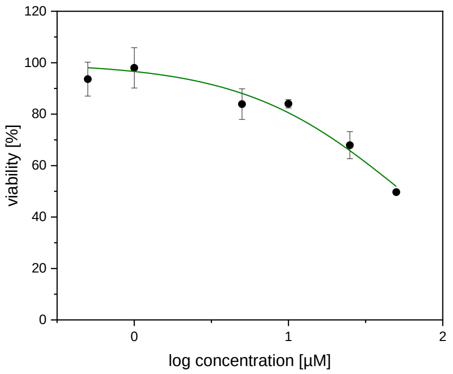

12d >50

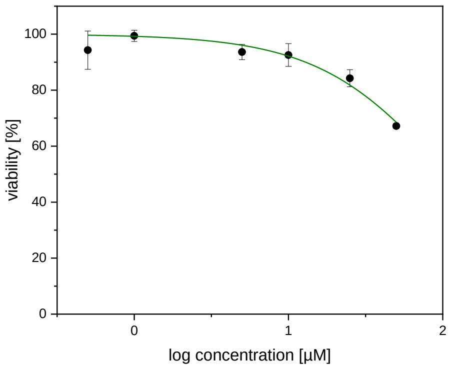

12e >50

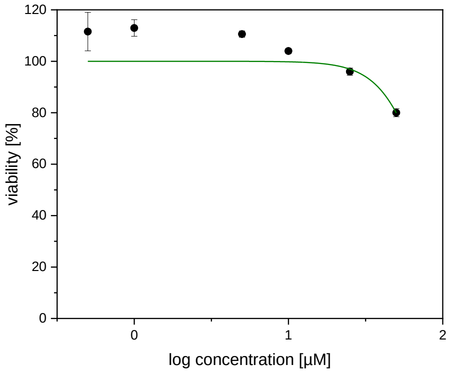

12f >50

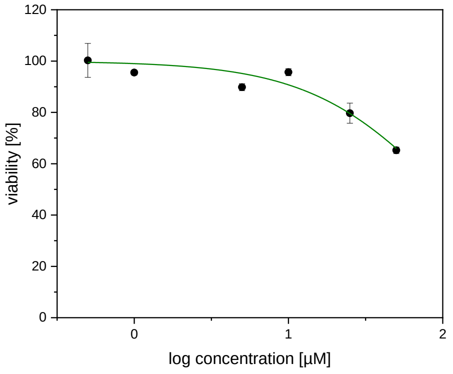

12g >50

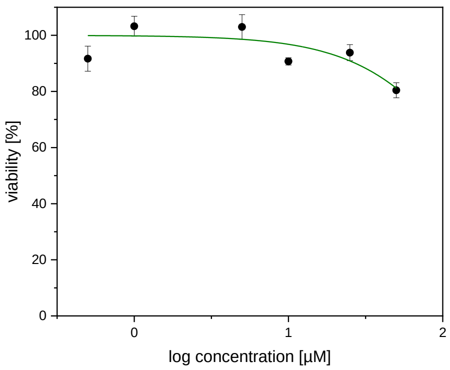

12h >50

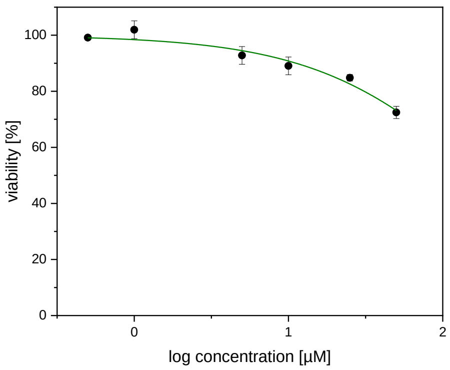

13a 47

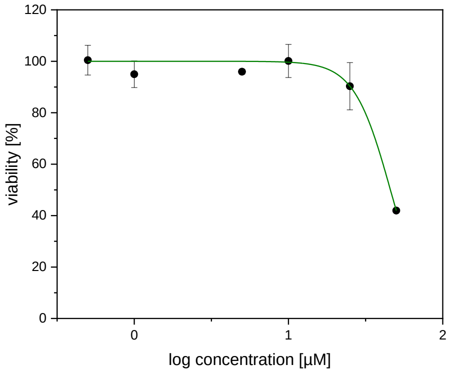

13b 45

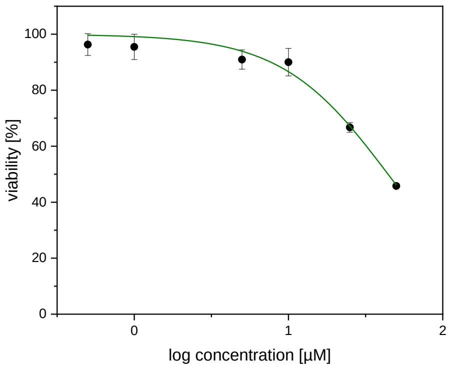

**13c**	>50	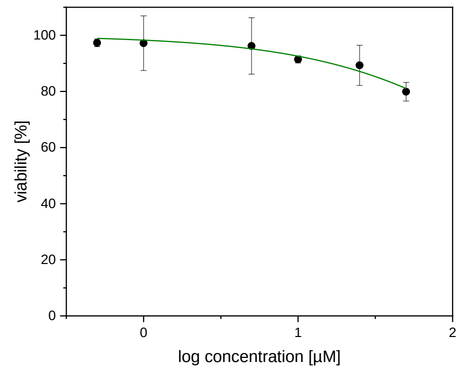
**13d**	20	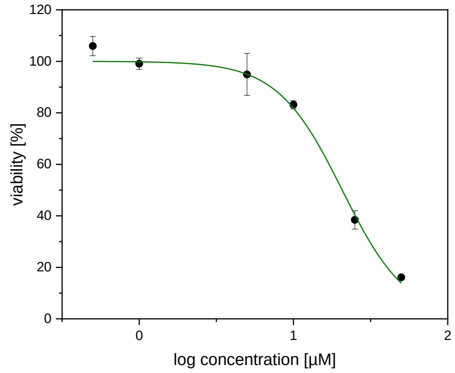
**13e**	>50	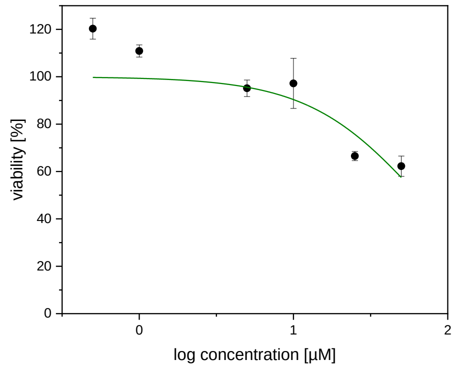
**13f**	46	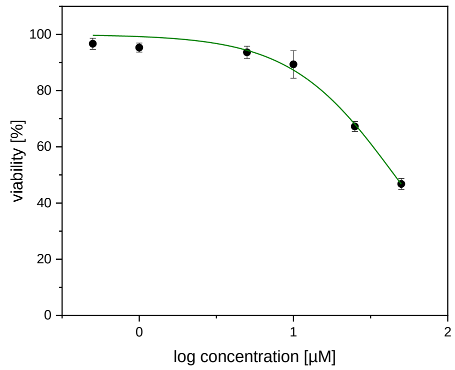

**13g**	21	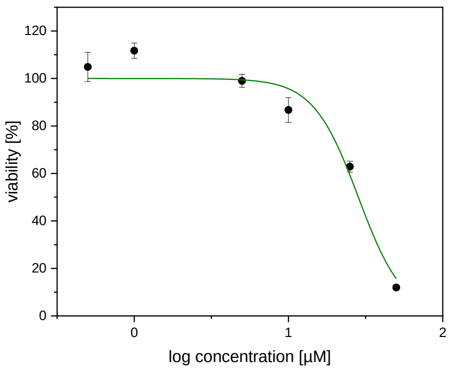
**13h**	29	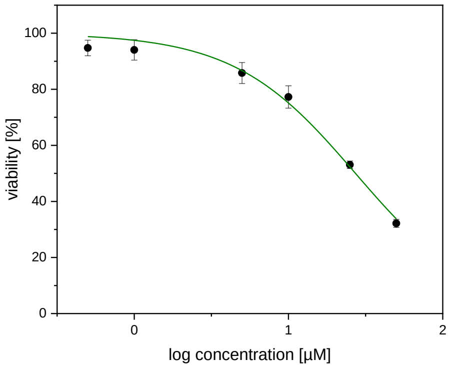
**16f**	>50	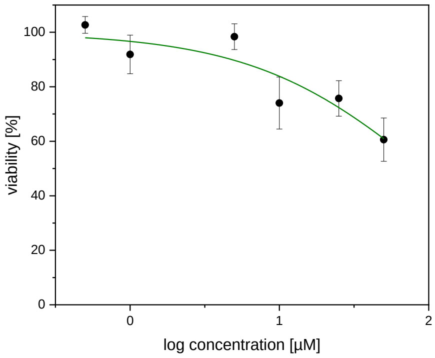

Table S3. Graphical overview about selected pyrazolo[1,2,3]triazines **5** and intermediates **9**, **12** and **13** and their calculated IC_{50} values after treatment of HeLa cells with different concentrations of the respective compounds.

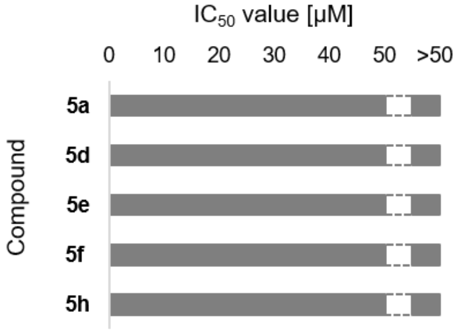

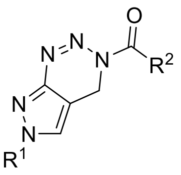

5

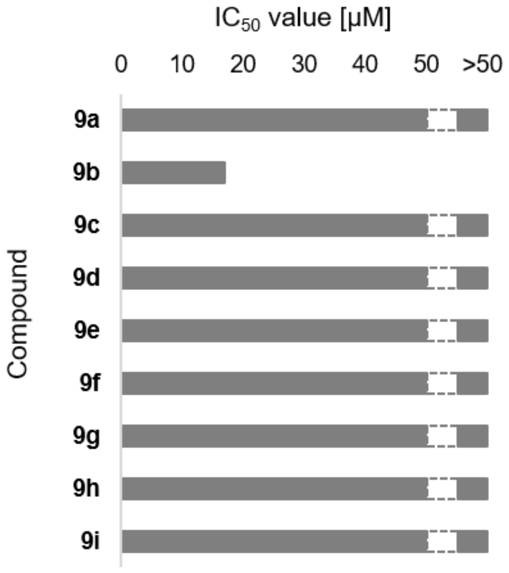
IC50 value [µM]
0 10 20 30 40 50 >50
Compound
9a
9b
9c
9d
9e
9f
9g
9h
9i

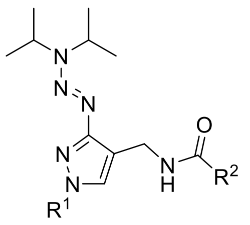
N
N
N
O
N
N
N
H
R1
R2

9

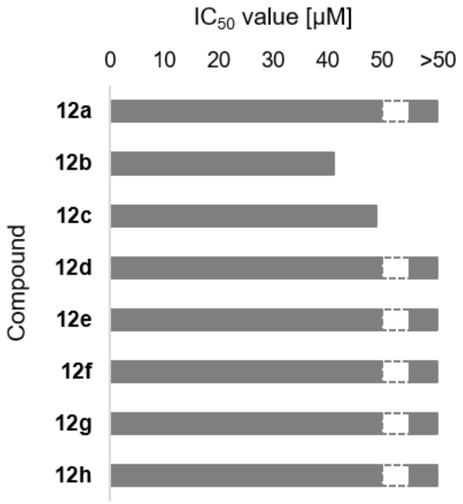
IC50 value [µM]
0 10 20 30 40 50 >50
Compound
12a
12b
12c
12d
12e
12f
12g
12h

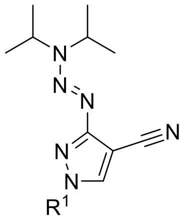
N
N
N
N
N
N
R1

12

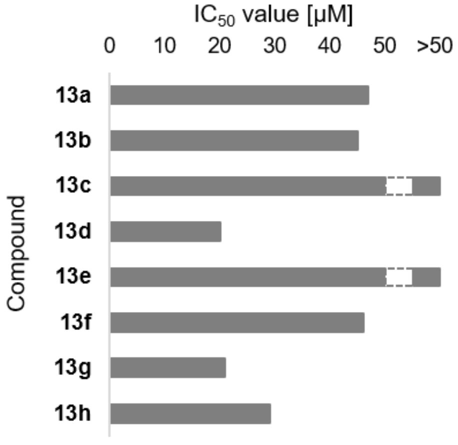
IC50 value [µM]
0 10 20 30 40 50 >50
Compound
13a
13b
13c
13d
13e
13f
13g
13h

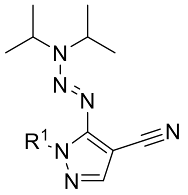
N
N
N
R1
N
N
N

13

6. References

1. Mosmann, T. *J. Immunol. Methods* **1983**, *65*, 55-63.
