# Supplementary material for: Synthesis of new pyrazolo[1,2,3]triazines by cyclative cleavage of pyrazolyltriazenes
Source: Beilstein J Org Chem. 2021 Nov 22;17:2773–80. doi: 10.3762/bjoc.17.187 (PMC8630434; doi:10.3762/bjoc.17.187)

BEILSTEIN JOURNAL OF ORGANIC CHEMISTRY

Supporting Information

for

Synthesis of new pyrazolo[1,2,3]triazines by cyclative cleavage of pyrazolyltriazenes

Nicolai Wippert, Martin Nieger, Claudine Herlan, Nicole Jung and Stefan Bräse

Beilstein J. Org. Chem. **2021,** *17,* 2773–2780. doi:10.3762/bjoc.17.187

Copies of spectra

License and Terms: This is a supporting information file under the terms of the Creative Commons Attribution License (https://creativecommons.org/licenses/by/4.0). Please note that the reuse, redistribution and reproduction in particular requires that the author(s) and source are credited and that individual graphics may be subject to special legal provisions.
The license is subject to the *Beilstein Journal of Organic Chemistry* terms and conditions: (https://www.beilstein-journals.org/bjoc/terms)

Table of contents

(*E*)-1-Benzyl-3-(3,3-diisopropyltriaz-1-en-1-yl)-1*H*-pyrazole-4-carbonitrile (**12a**) S2

(*E*)-1-Benzyl-5-(3,3-diisopropyltriaz-1-en-1-yl)-1*H*-pyrazole-4-carbonitrile (**13a**) S4

(*E*)-3-(3,3-Diisopropyltriaz-1-en-1-yl)-1-(4-methylbenzyl)-1*H*-pyrazole-4-carbonitrile (**12b**) S6

(*E*)-5-(3,3-Diisopropyltriaz-1-en-1-yl)-1-(4-methylbenzyl)-1*H*-pyrazole-4-carbonitrile (**13b**) S8

(*E*)-1-(3,5-Difluorobenzyl)-3-(3,3-diisopropyltriaz-1-en-1-yl)-1*H*-pyrazole-4-carbonitrile (**12c**) S10

(*E*)-1-(3,5-Difluorobenzyl)-5-(3,3-diisopropyltriaz-1-en-1-yl)-1*H*-pyrazole-4-carbonitrile (**13c**) S13

(*E*)-3-(3,3-Diisopropyltriaz-1-en-1-yl)-1-ethyl-1*H*-pyrazole-4-carbonitrile (**12d**) S16

(*E*)-5-(3,3-Diisopropyltriaz-1-en-1-yl)-1-ethyl-1*H*-pyrazole-4-carbonitrile (**13d**) S18

(*E*)-1-Cyclopentyl-3-(3,3-diisopropyltriaz-1-en-1-yl)-1*H*-pyrazole-4-carbonitrile (**12e**) S20

(*E*)-1-Cyclopentyl-5-(3,3-diisopropyltriaz-1-en-1-yl)-1*H*-pyrazole-4-carbonitrile (**13e**) S22

(*E*)-3-(3,3-Diisopropyltriaz-1-en-1-yl)-1-isobutyl-1*H*-pyrazole-4-carbonitrile (**12f**) S24

(*E*)-5-(3,3-Diisopropyltriaz-1-en-1-yl)-1-isobutyl-1*H*-pyrazole-4-carbonitrile (**13f**) S26

Ethyl (*E*)-2-(4-cyano-3-(3,3-diisopropyltriaz-1-en-1-yl)-1*H*-pyrazol-1-yl)acetate (**12g**) S28

Ethyl (*E*)-2-(4-cyano-5-(3,3-diisopropyltriaz-1-en-1-yl)-1*H*-pyrazol-1-yl)acetate (**13g**) S30

(*E*)-1-(4-Bromobenzyl)-3-(3,3-diisopropyltriaz-1-en-1-yl)-1*H*-pyrazole-4-carbonitrile (**12h**) S32

(*E*)-1-(4-Bromobenzyl)-5-(3,3-diisopropyltriaz-1-en-1-yl)-1*H*-pyrazole-4-carbonitrile (**13h**) S34

(*E*)-*N*-((1-Benzyl-3-(3,3-diisopropyltriaz-1-en-1-yl)-1*H*-pyrazol-4-yl)methyl)acetamido (**9a**) S36

(*E*)-*N*-((1-Benzyl-3-(3,3-diisopropyltriaz-1-en-1-yl)-1*H*-pyrazol-4-yl)methyl)benzamide (**9b**) S38

(*E*)-*N*-((3-(3,3-Diisopropyltriaz-1-en-1-yl)-1-(4-methylbenzyl)-1*H*-pyrazol-4-yl)methyl)-3-methy-lbutanamide (**9c**) S40

(*E*)-*N*-((1-(3,5-Difluorobenzyl)-3-(3,3-diisopropyltriaz-1-en-1-yl)-1*H*-pyrazol-4-yl)methyl)acetamido (**9d**) S42

(*E*)-*N*-((3-(3,3-Diisopropyltriaz-1-en-1-yl)-1-ethyl-1*H*-pyrazol-4-yl)methyl)acetamido (**9e**) S45

(*E*)-*N*-((1-Cyclopentyl-3-(3,3-diisopropyltriaz-1-en-1-yl)-1*H*-pyrazol-4-yl)methyl)acetamido (**9f**) S47

(*E*)-*N*-((1-Cyclopentyl-3-(3,3-diisopropyltriaz-1-en-1-yl)-1*H*-pyrazol-4-yl)methyl)benzamide (**9g**) S49

(*E*)-*N*-((3-(3,3-Diisopropyltriaz-1-en-1-yl)-1-isobutyl-1*H*-pyrazol-4-yl)methyl)acetamido (**9h**) S51

(*E*)-2-(4-(Acetamidomethyl)-3-(3,3-diisopropyltriaz-1-en-1-yl)-1*H*-pyrazol-1-yl)ethyl acetate (**9i**) S53

1-(6-Benzyl-4,6-dihydro-3*H*-pyrazolo[3,4-*d*][1,2,3]triazin-3-yl)ethan-1-one (**5a**) S55

(6-Benzyl-4,6-dihydro-3*H*-pyrazolo[3,4-*d*][1,2,3]triazin-3-yl)(phenyl)methanone (**5b**) S57

3-Methyl-1-(6-(4-methylbenzyl)-4,6-dihydro-3*H*-pyrazolo[3,4-*d*][1,2,3]triazin-3-yl)butan-1-one (**5c**) S59

1-(6-(3,5-Difluorobenzyl)-4,6-dihydro-3*H*-pyrazolo[3,4-*d*][1,2,3]triazin-3-yl)ethan-1-one (**5d**) S61

1-(6-Ethyl-4,6-dihydro-3*H*-pyrazolo[3,4-*d*][1,2,3]triazin-3-yl)ethan-1-one (**5e**) S63

1-(6-Cyclopentyl-4,6-dihydro-3*H*-pyrazolo[3,4-*d*][1,2,3]triazin-3-yl)ethan-1-one (**5f**) S65

(6-Cyclopentyl-4,6-dihydro-3*H*-pyrazolo[3,4-*d*][1,2,3]triazin-3-yl)(phenyl)methanone (**5g**) S67

1-(6-Isobutyl-4,6-dihydro-3*H*-pyrazolo[3,4-*d*][1,2,3]triazin-3-yl)ethan-1-one (**5h**) S69

2-(3-Acetyl-3,4-dihydro-6*H*-pyrazolo[3,4-*d*][1,2,3]triazin-6-yl)ethyl acetate (**5i**) S71

(*E*)-1-Benzyl-3-(3,3-diisopropyltriaz-1-en-1-yl)-1*H*-pyrazole-4-carbonitrile (12a)

CHMO:0000593 | 1H nuclear magnetic resonance spectroscopy (1H NMR)

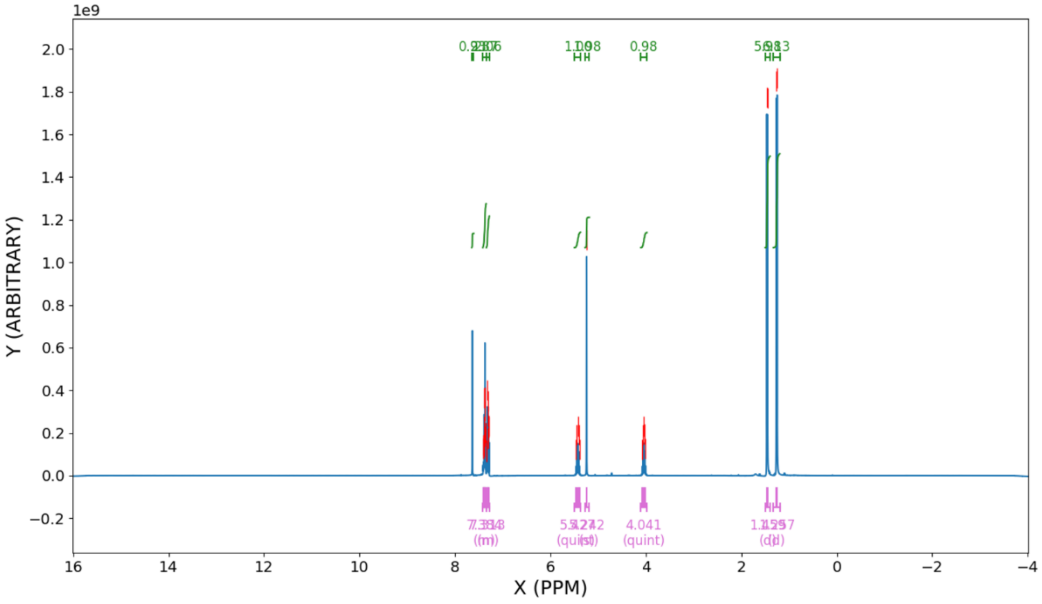

CHMO:0000595 | 13C nuclear magnetic resonance spectroscopy (13C NMR)

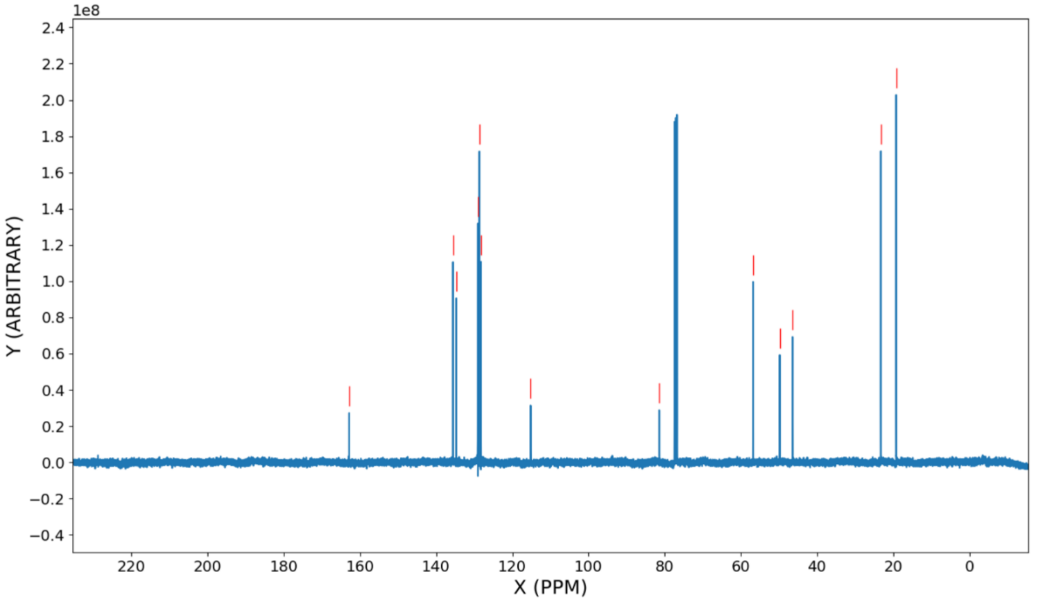

CHMO:0000470 | mass spectrometry (MS)

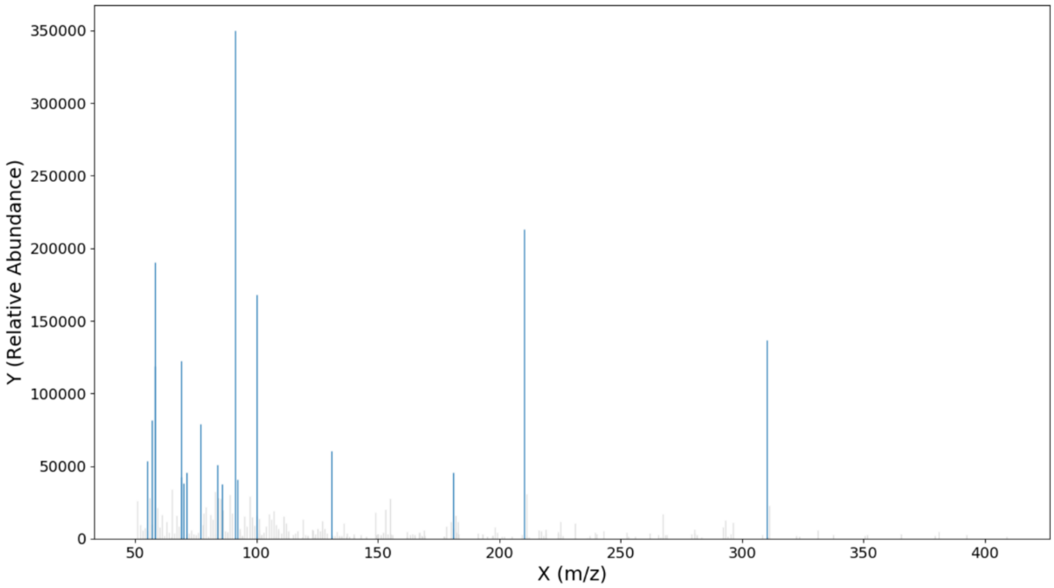

CHMO:0000630 | infrared absorption spectroscopy (IR)

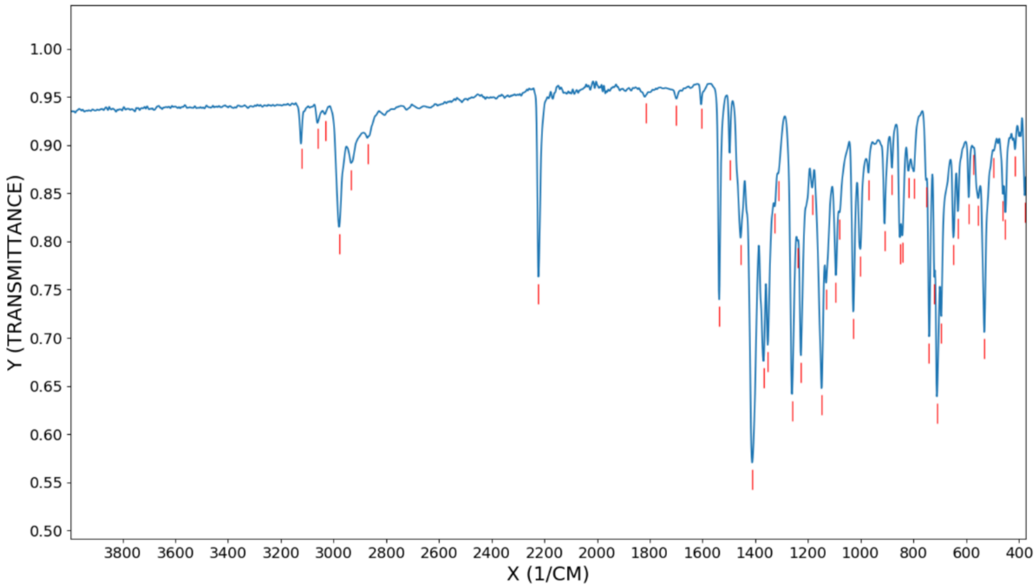

(*E*)-1-Benzyl-5-(3,3-diisopropyltriaz-1-en-1-yl)-1*H*-pyrazole-4-carbonitrile (13a)

CHMO:0000593 | 1H nuclear magnetic resonance spectroscopy (1H NMR)

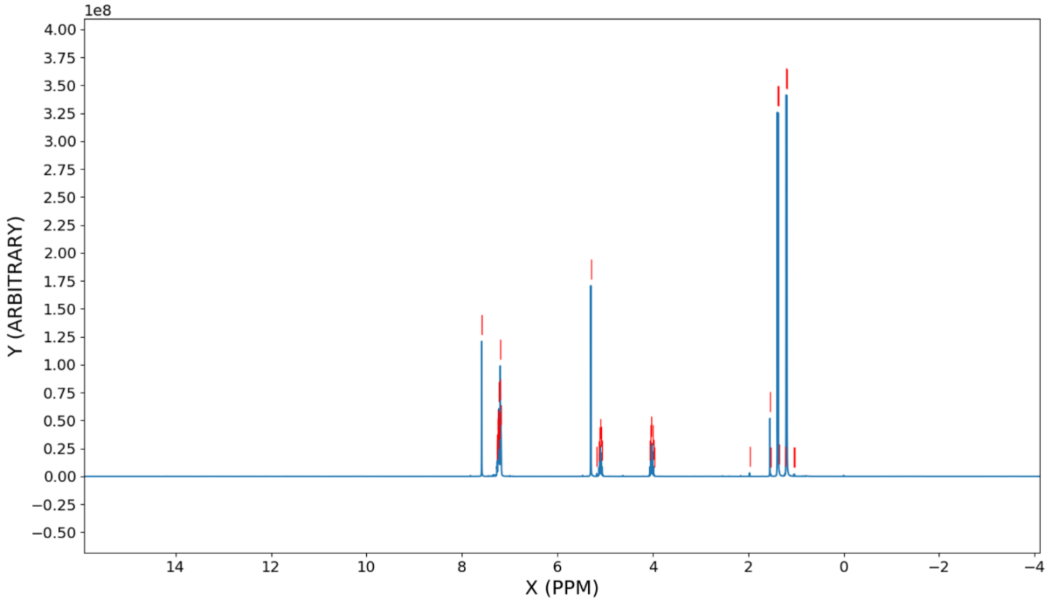

CHMO:0000595 | 13C nuclear magnetic resonance spectroscopy (13C NMR)

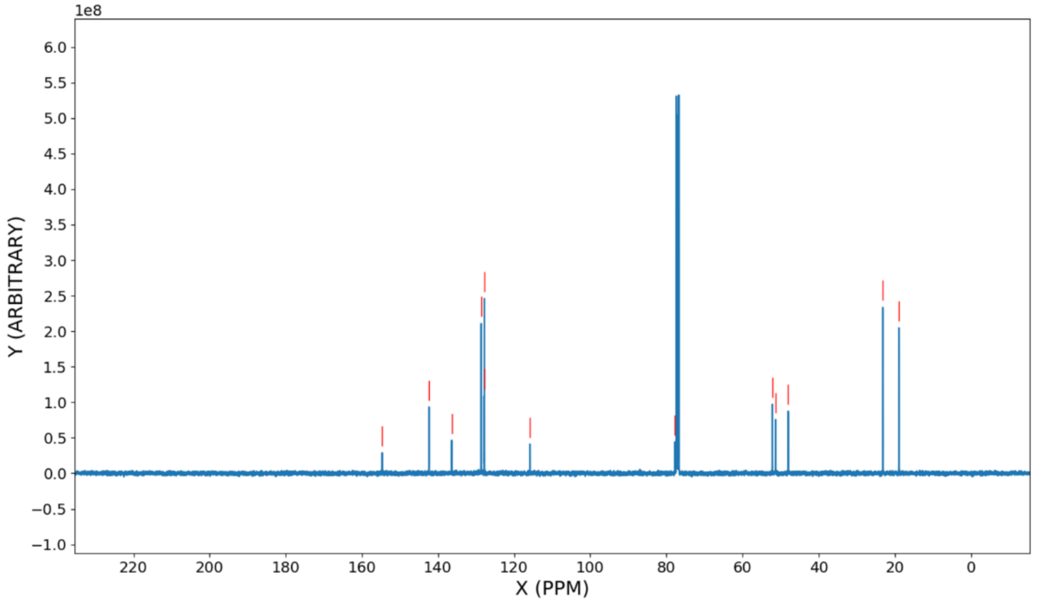

CHMO:0000480 | electron ionisation mass spectrometry (EI-MS)

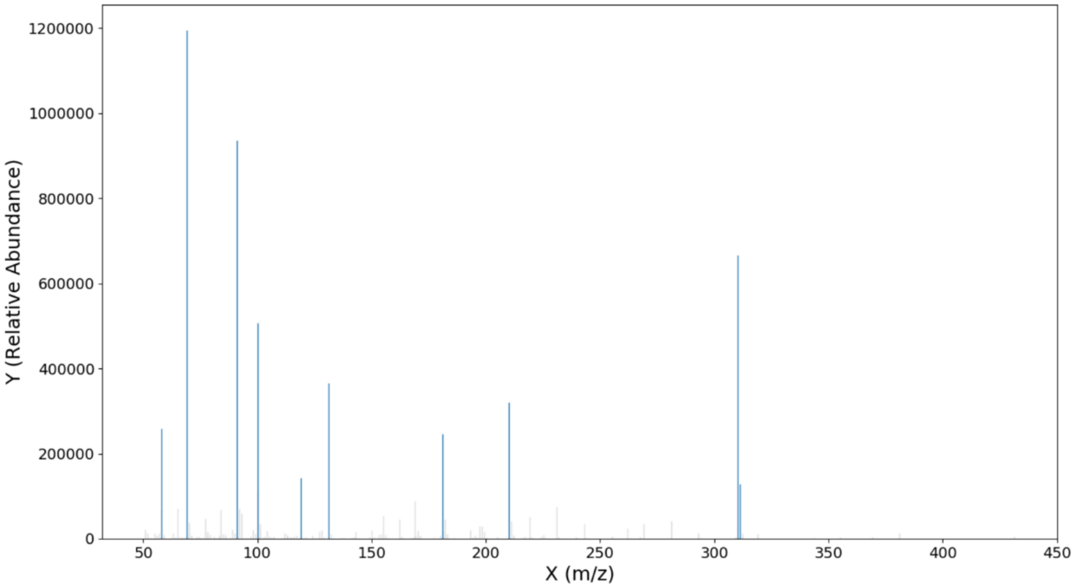

CHMO:0000630 | infrared absorption spectroscopy (IR)

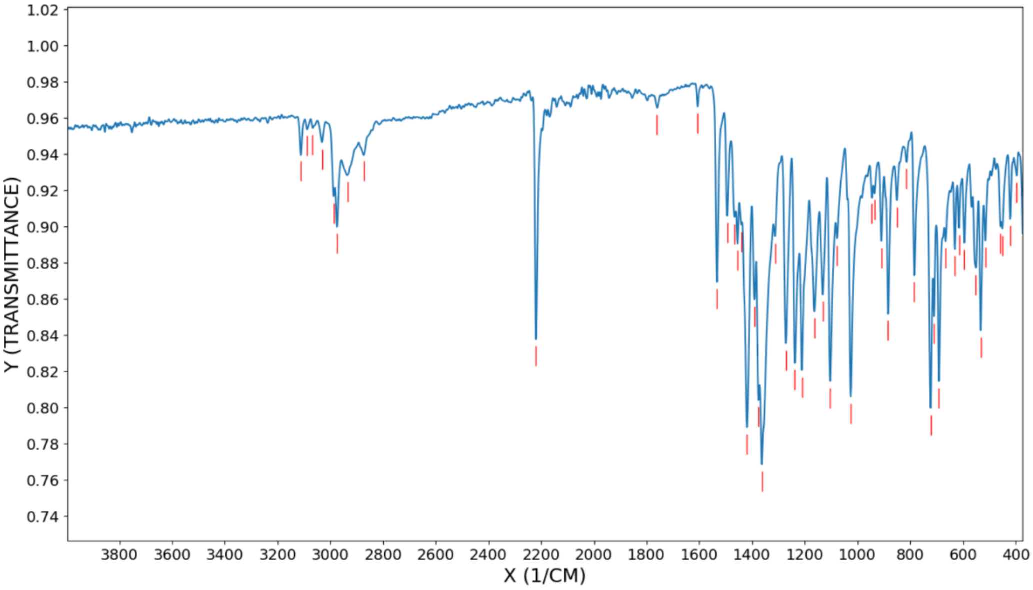

(*E*)-3-(3,3-Diisopropyltriaz-1-en-1-yl)-1-(4-methylbenzyl)-1*H*-pyrazole-4-carbonitrile (12b)

CHMO:0000593 | 1H nuclear magnetic resonance spectroscopy (1H NMR)

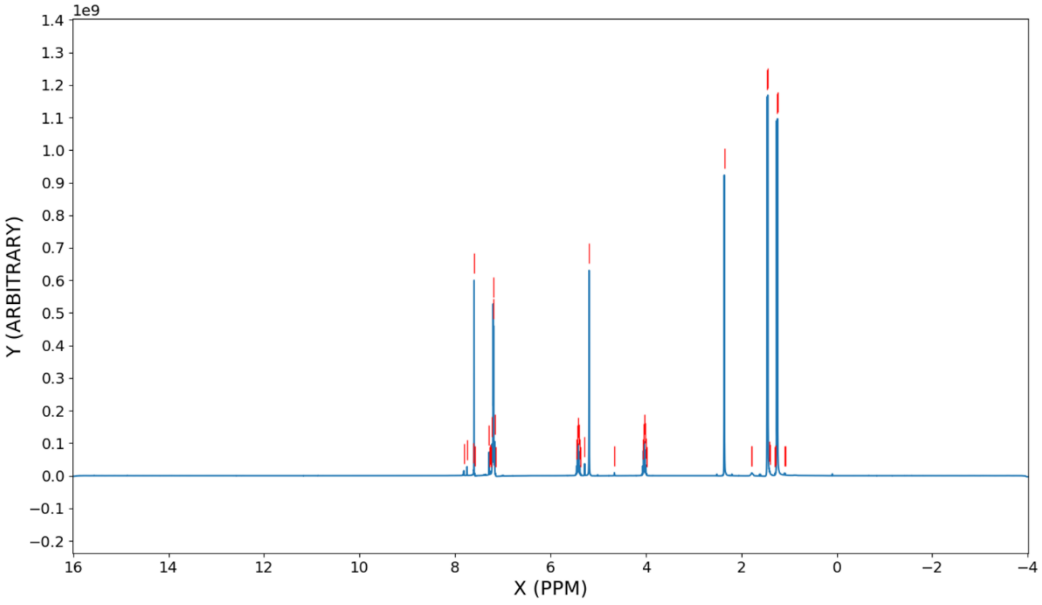

CHMO:0000595 | 13C nuclear magnetic resonance spectroscopy (13C NMR)

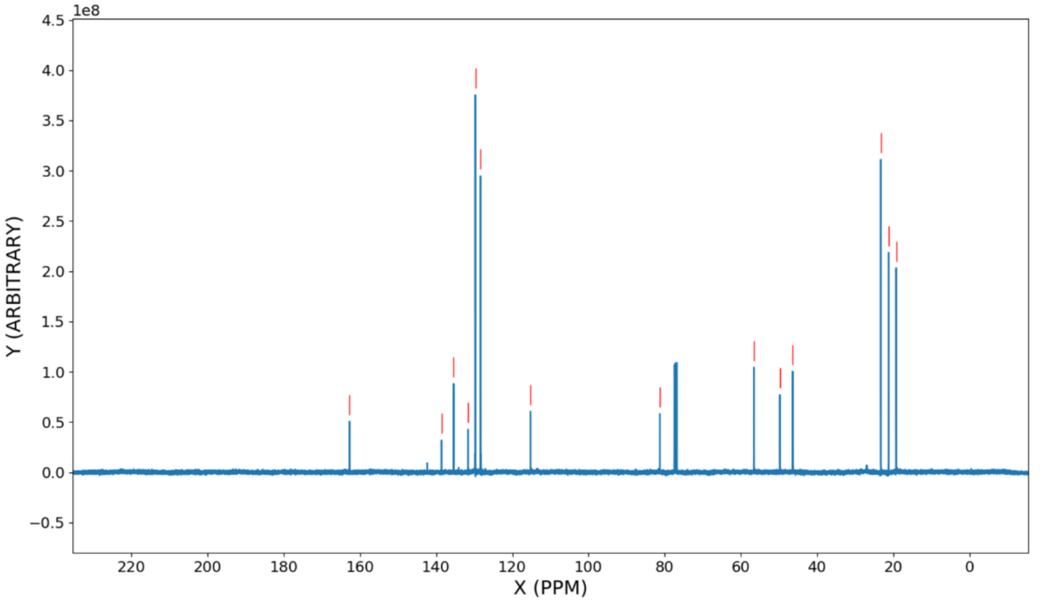

CHMO:0000563 | fast-atom bombardment mass spectrometry (FABMS)

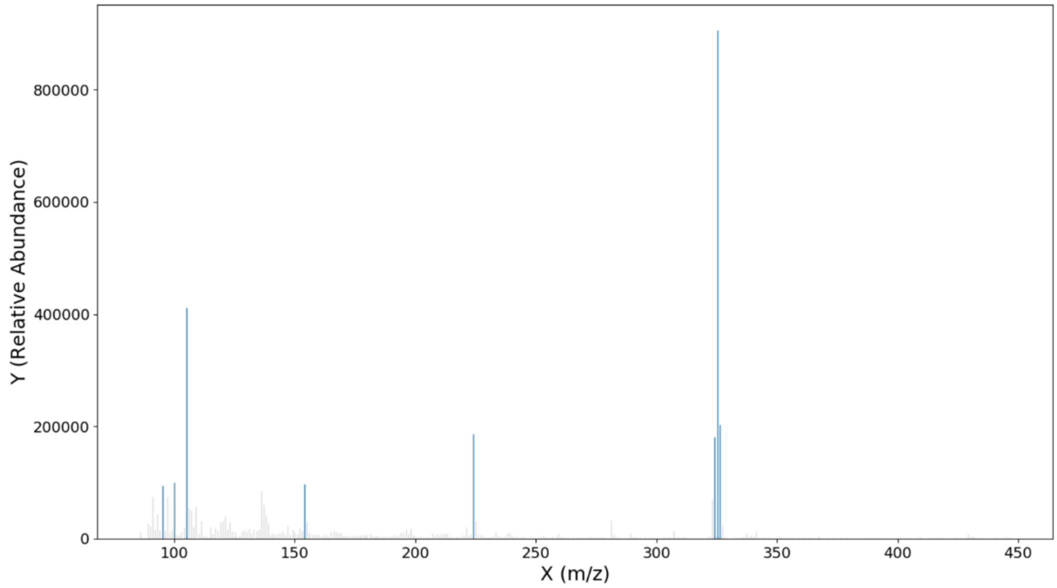

CHMO:0000630 | infrared absorption spectroscopy (IR)

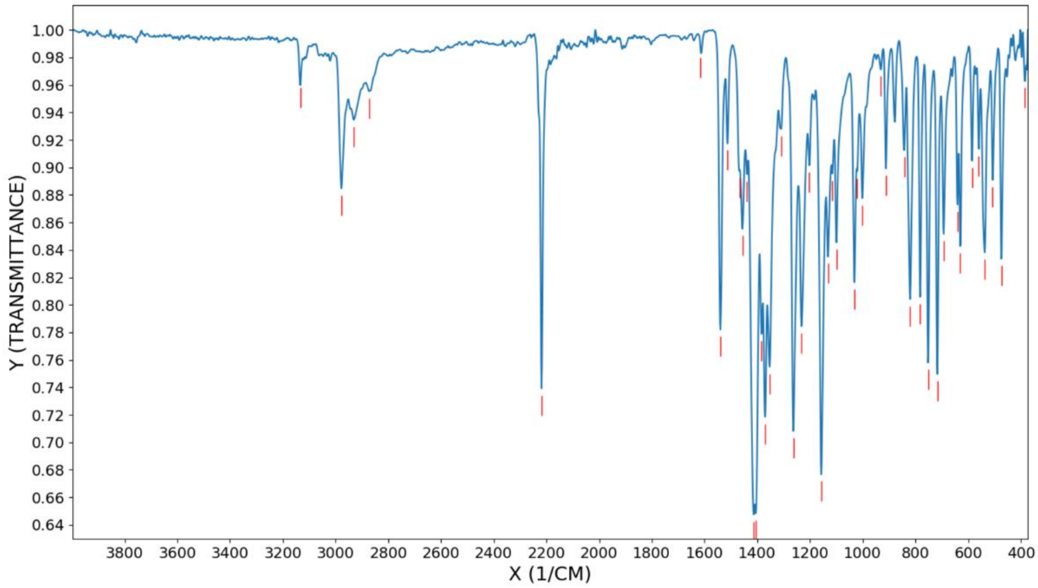

(*E*)-5-(3,3-Diisopropyltriaz-1-en-1-yl)-1-(4-methylbenzyl)-1*H*-pyrazole-4-carbonitrile (13b)

CHMO:0000593 | 1H nuclear magnetic resonance spectroscopy (1H NMR)

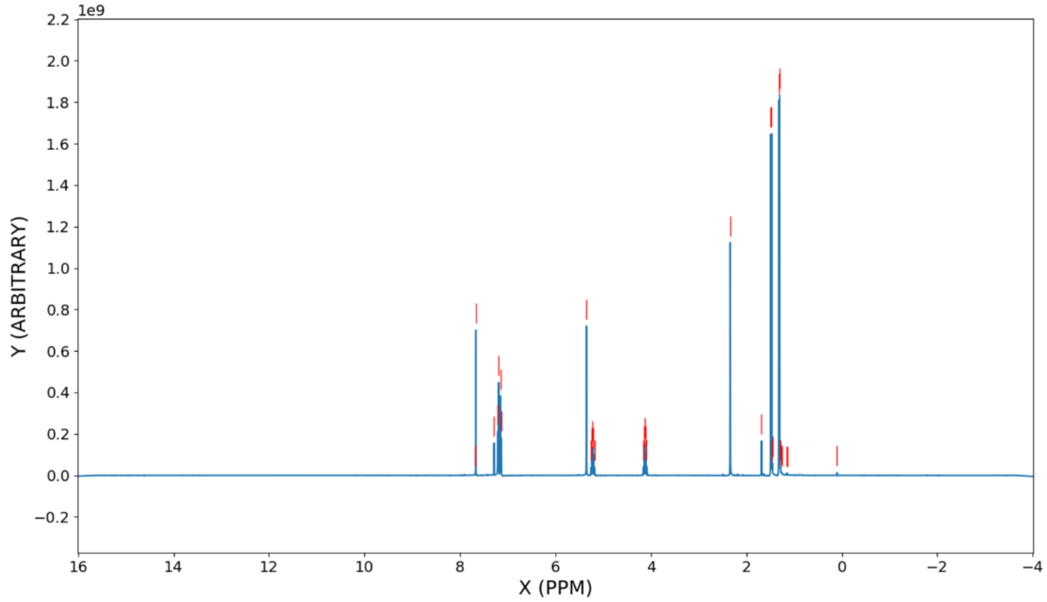

CHMO:0000595 | 13C nuclear magnetic resonance spectroscopy (13C NMR)

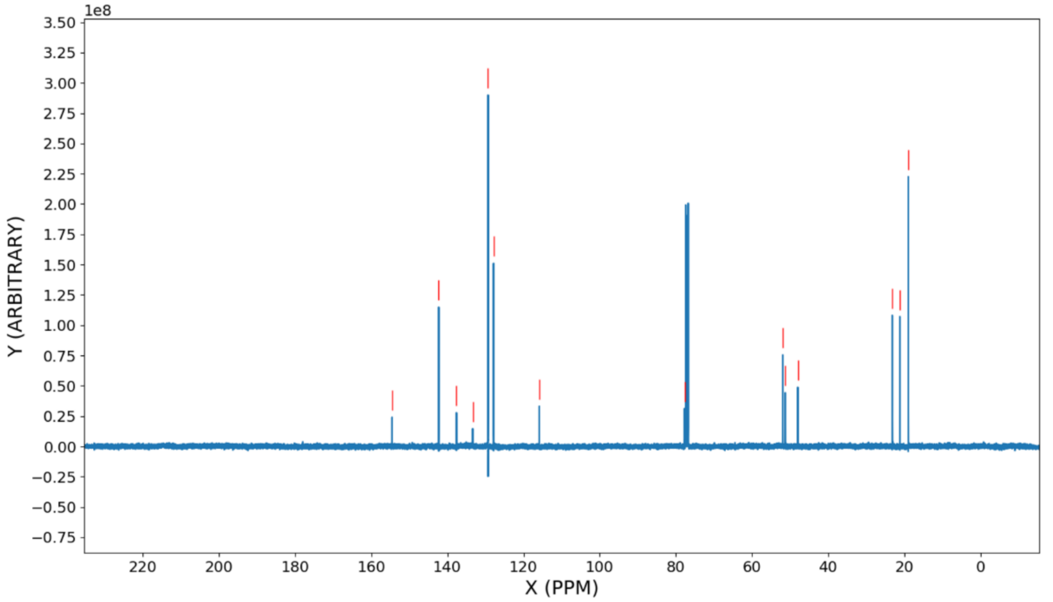

CHMO:0000563 | fast-atom bombardment mass spectrometry (FABMS)

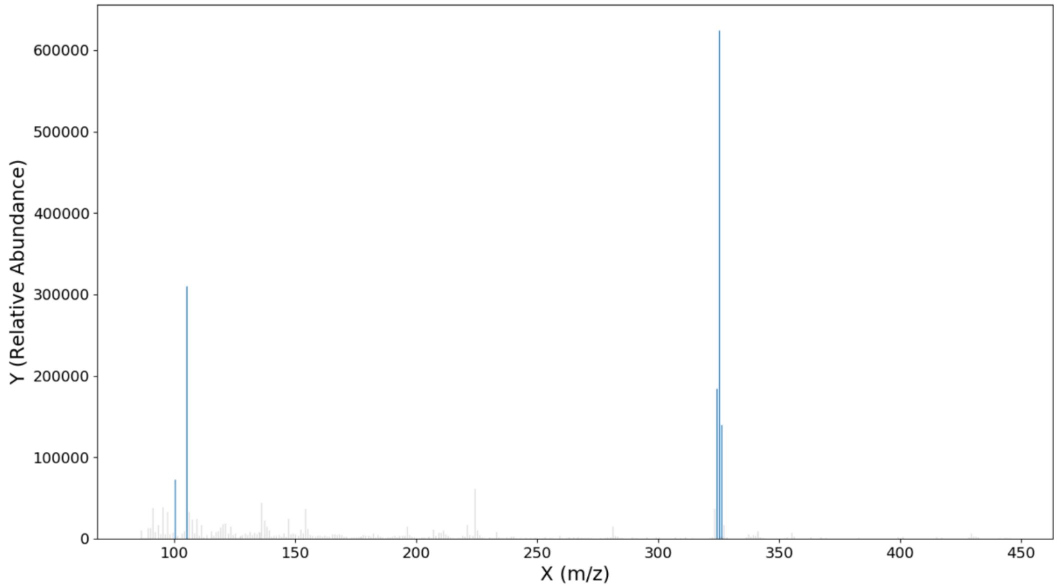

CHMO:0000630 | infrared absorption spectroscopy (IR)

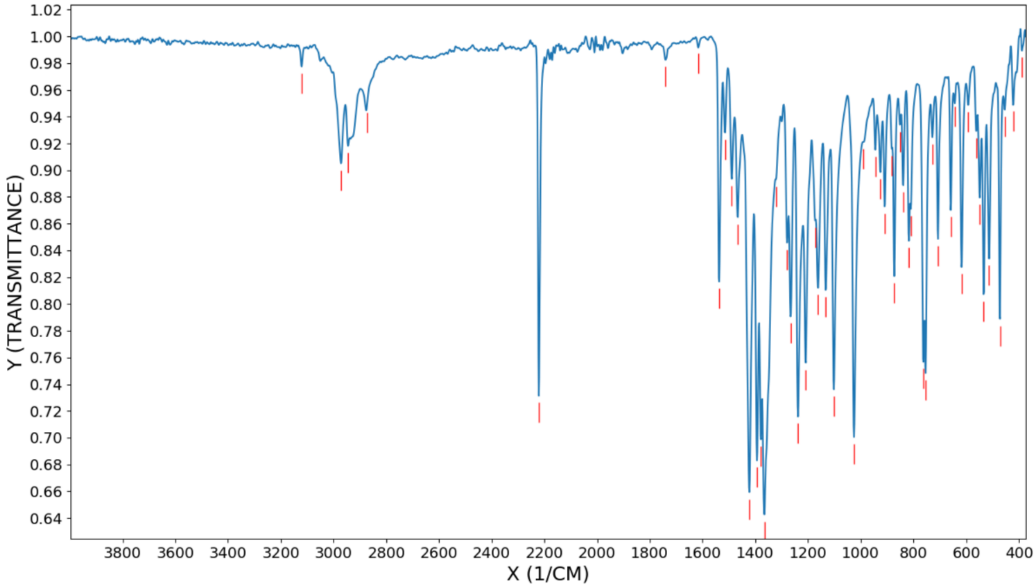

(*E*)-1-(3,5-Difluorobenzyl)-3-(3,3-diisopropyltriaz-1-en-1-yl)-1*H*-pyrazole-4-carbonitrile (12c)

CHMO:0000593 | 1H nuclear magnetic resonance spectroscopy (1H NMR)

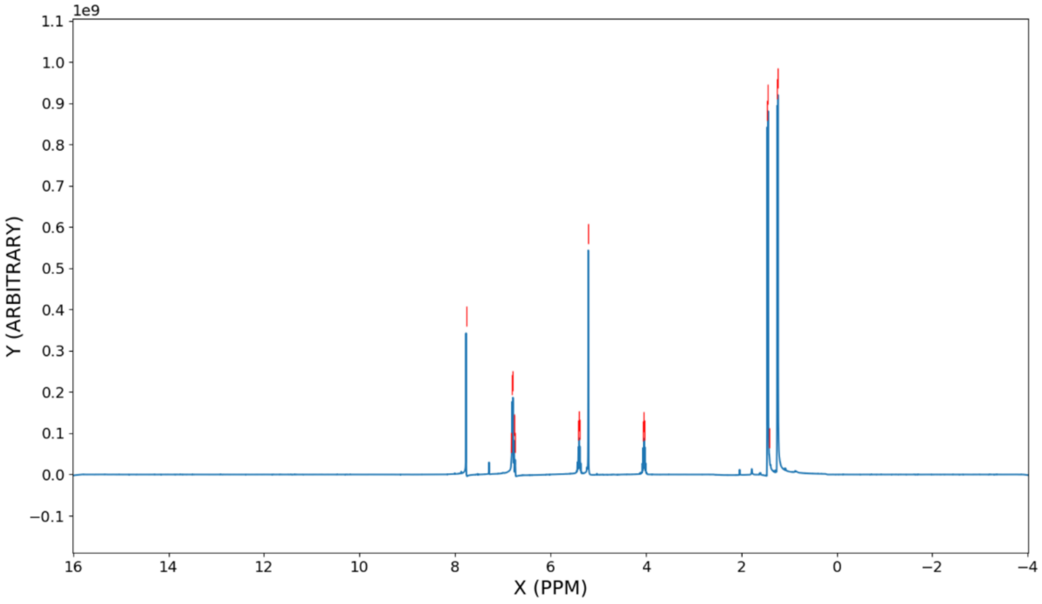

CHMO:0000595 | 13C nuclear magnetic resonance spectroscopy (13C NMR)

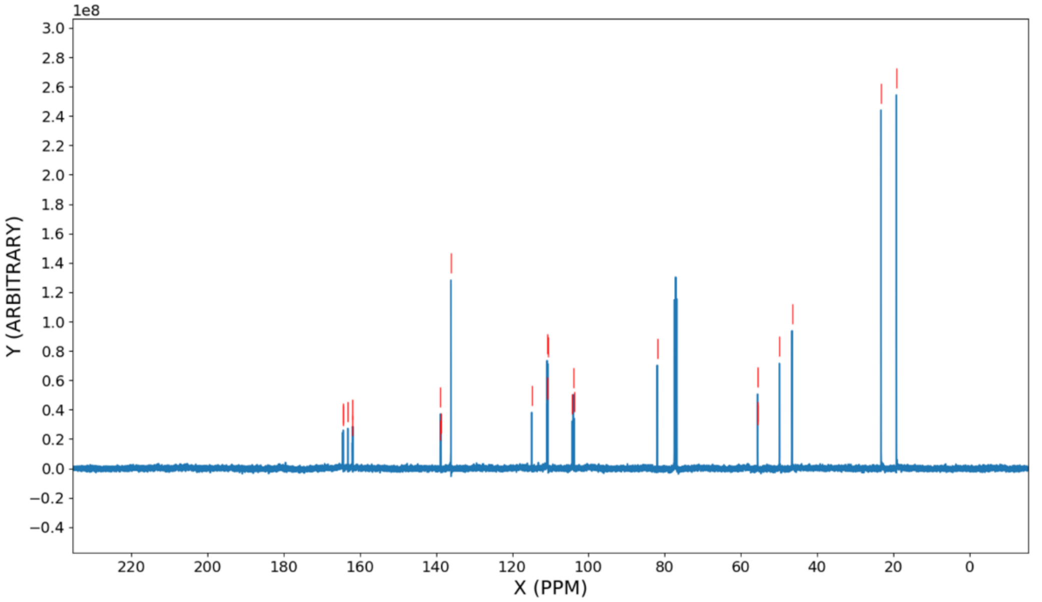

19F nuclear magnetic resonance spectroscopy (19F NMR)

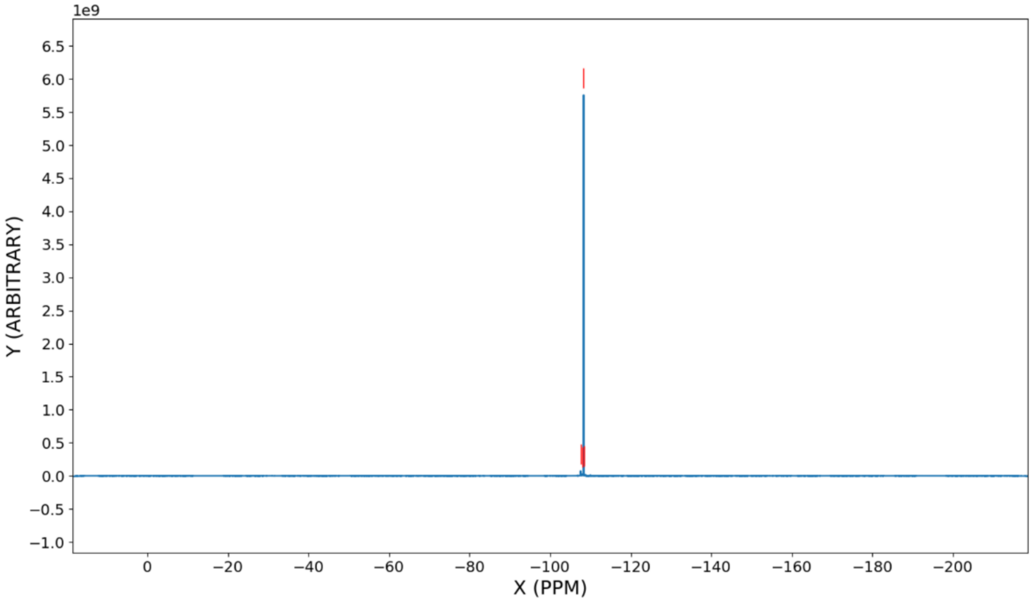

CHMO:0000480 | electron ionisation mass spectrometry (EI-MS)

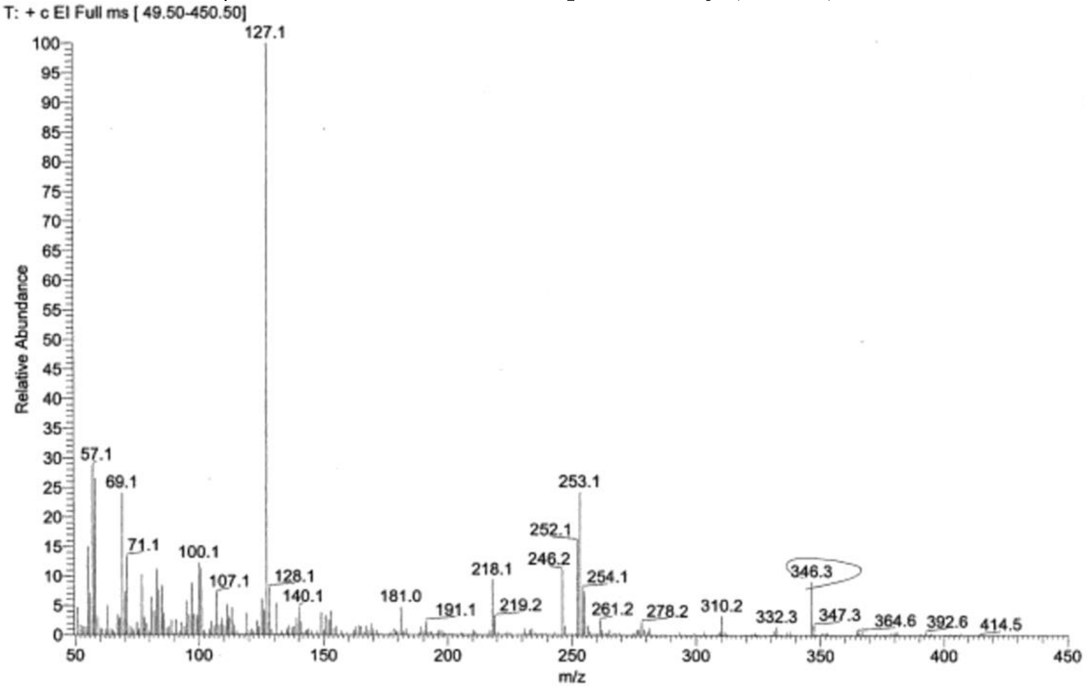

CHMO:0000630 | infrared absorption spectroscopy (IR)

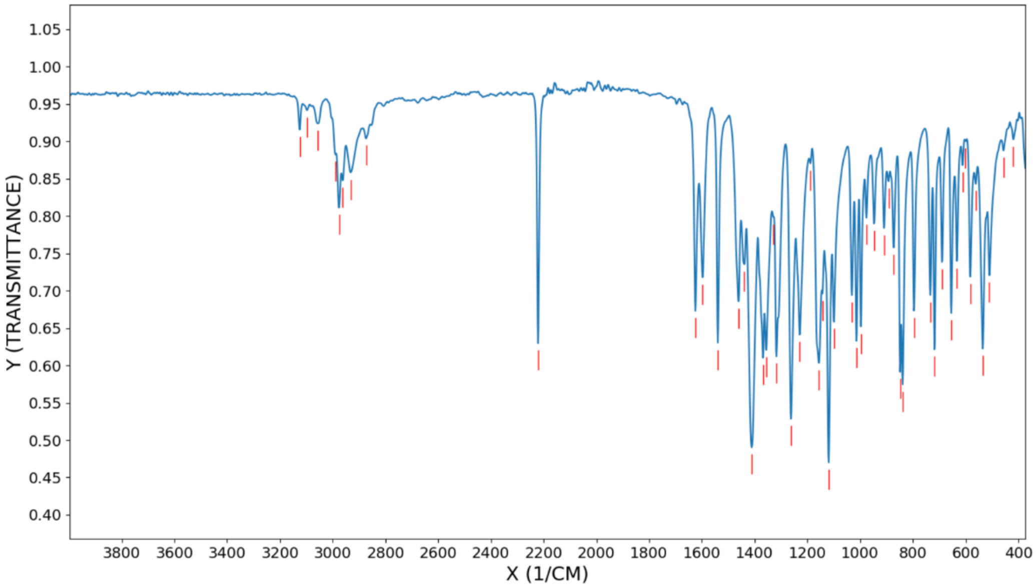

(E)-1-(3,5-Difluorobenzyl)-5-(3,3-diisopropyltriaz-1-en-1-yl)-1H-pyrazole-4-carbonitrile (13c)

CHMO:0000593 | 1H nuclear magnetic resonance spectroscopy (1H NMR)

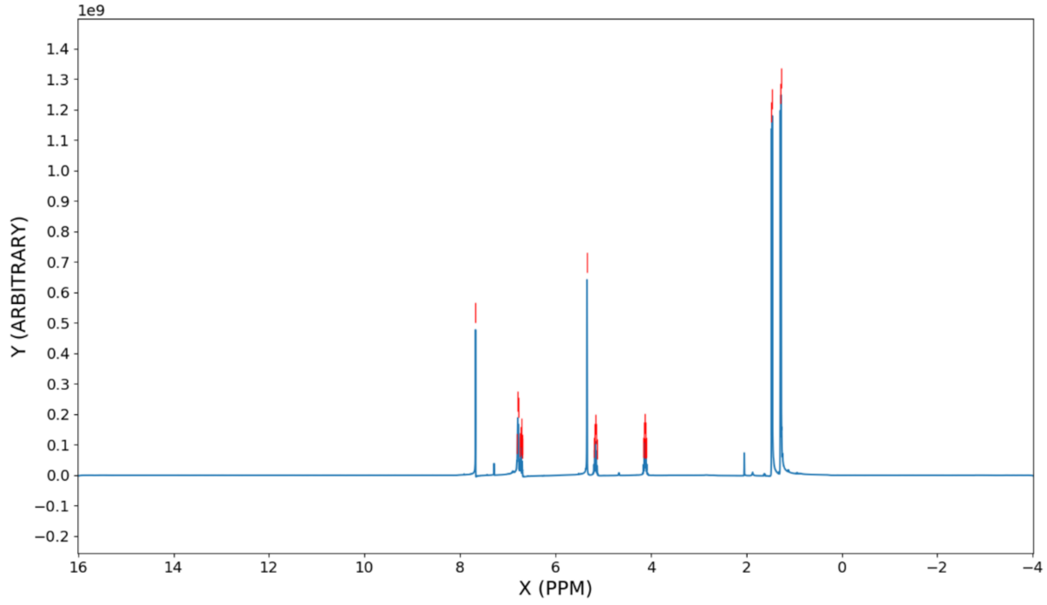

CHMO:0000595 | 13C nuclear magnetic resonance spectroscopy (13C NMR)

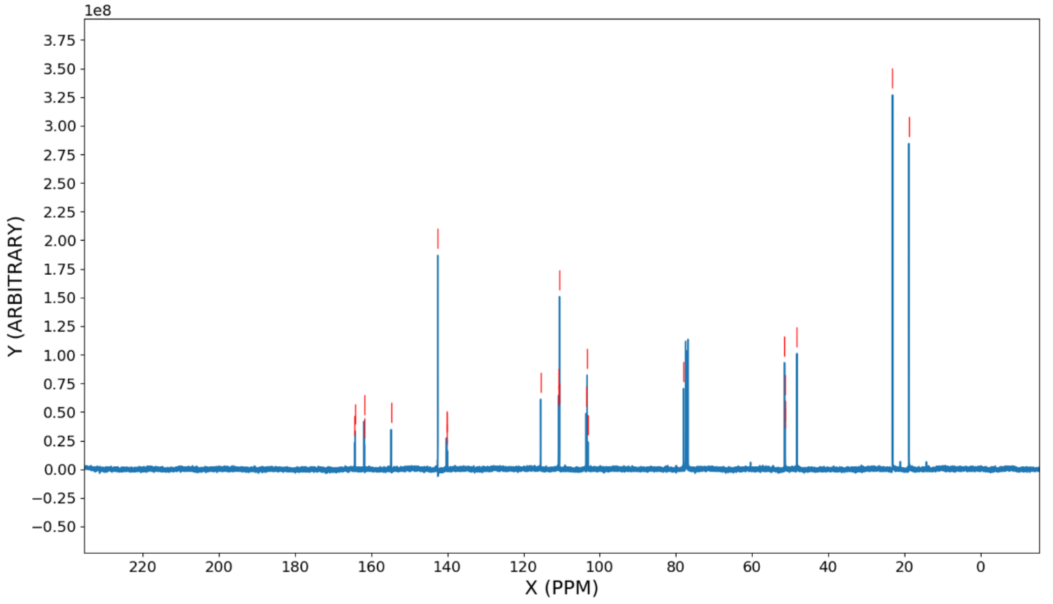

19F nuclear magnetic resonance spectroscopy (19F NMR)

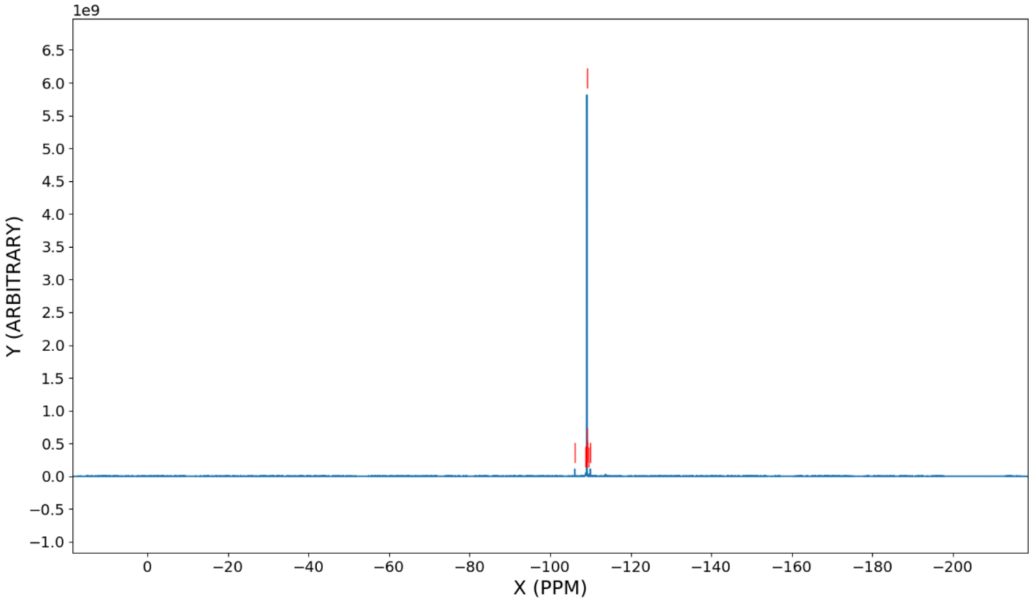

CHMO:0000480 | electron ionisation mass spectrometry (EI-MS)

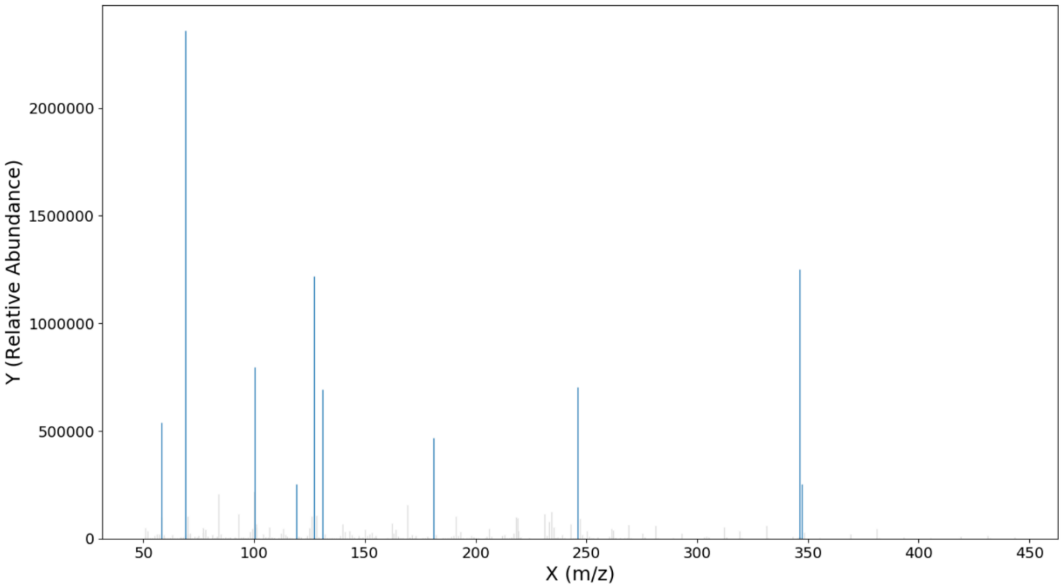

CHMO:0000630 | infrared absorption spectroscopy (IR)

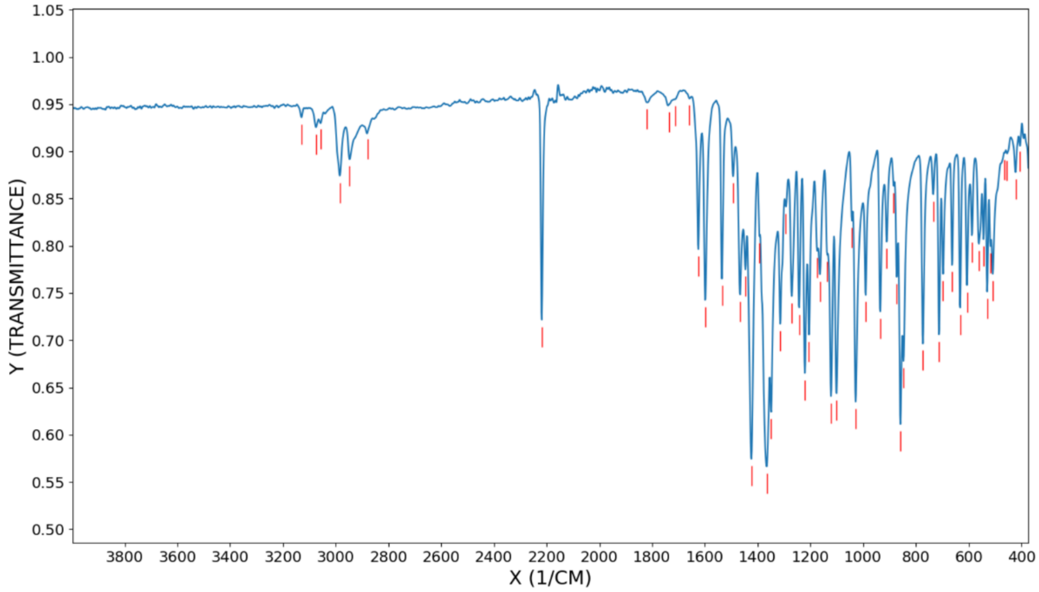

(E)-3-(3,3-Diisopropyltriaz-1-en-1-yl)-1-ethyl-1H-pyrazole-4-carbonitrile (12d)

CHMO:0000593 | 1H nuclear magnetic resonance spectroscopy (1H NMR)

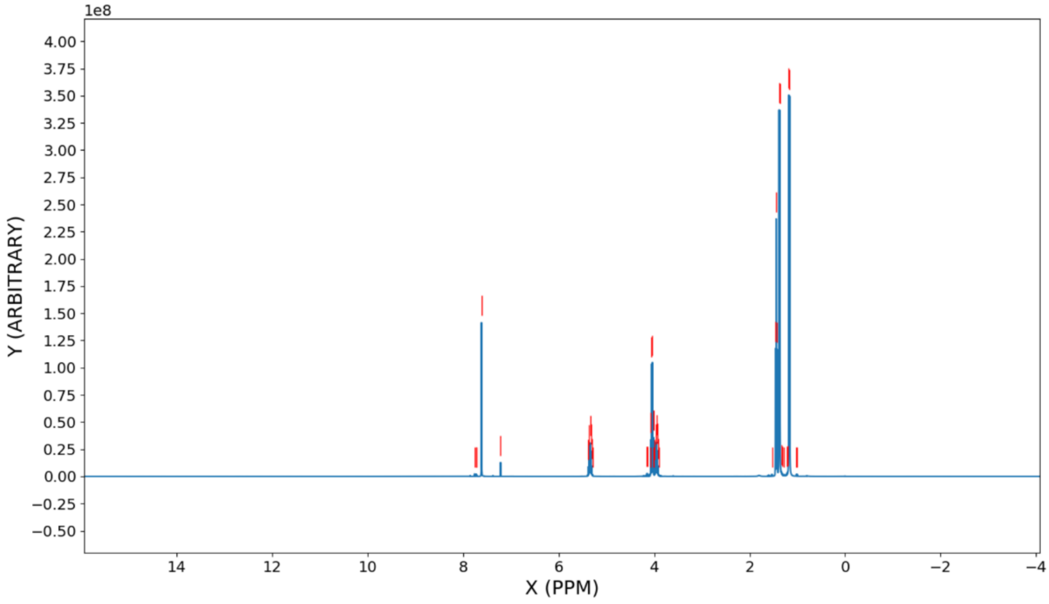

CHMO:0000595 | 13C nuclear magnetic resonance spectroscopy (13C NMR)

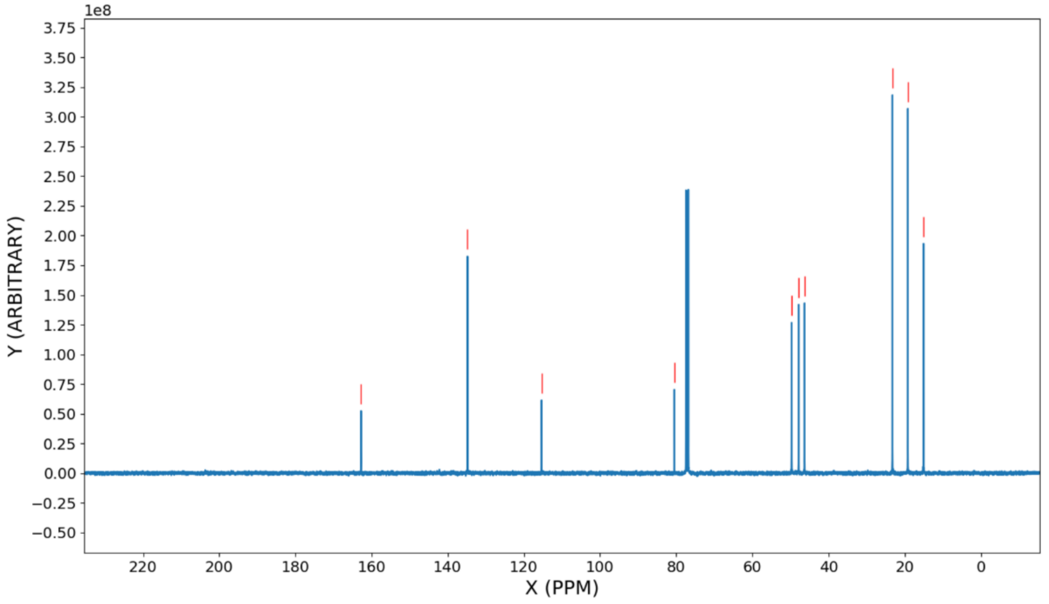

CHMO:0000563 | fast-atom bombardment mass spectrometry (FABMS)

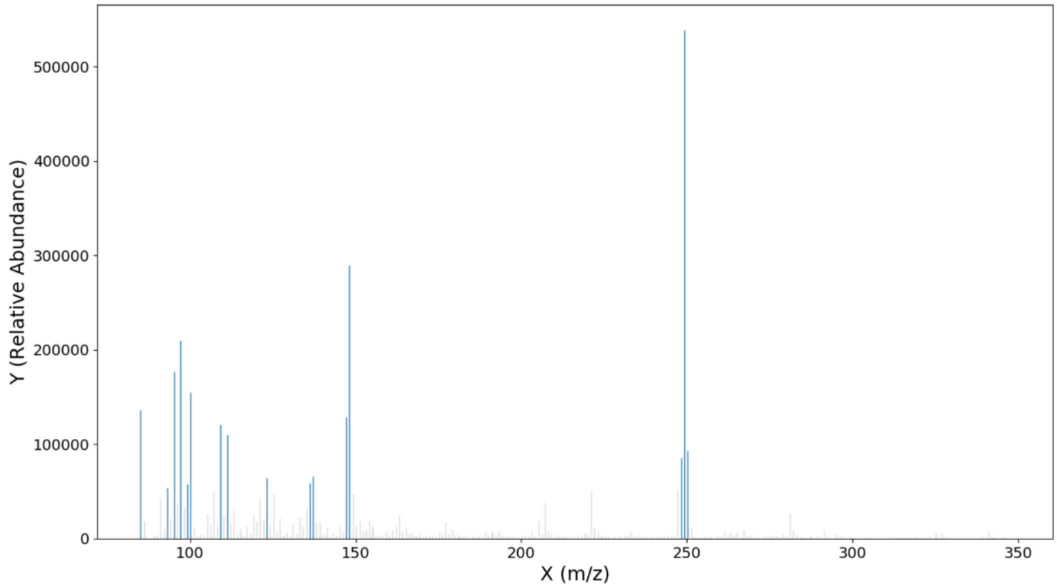

CHMO:0000630 | infrared absorption spectroscopy (IR)

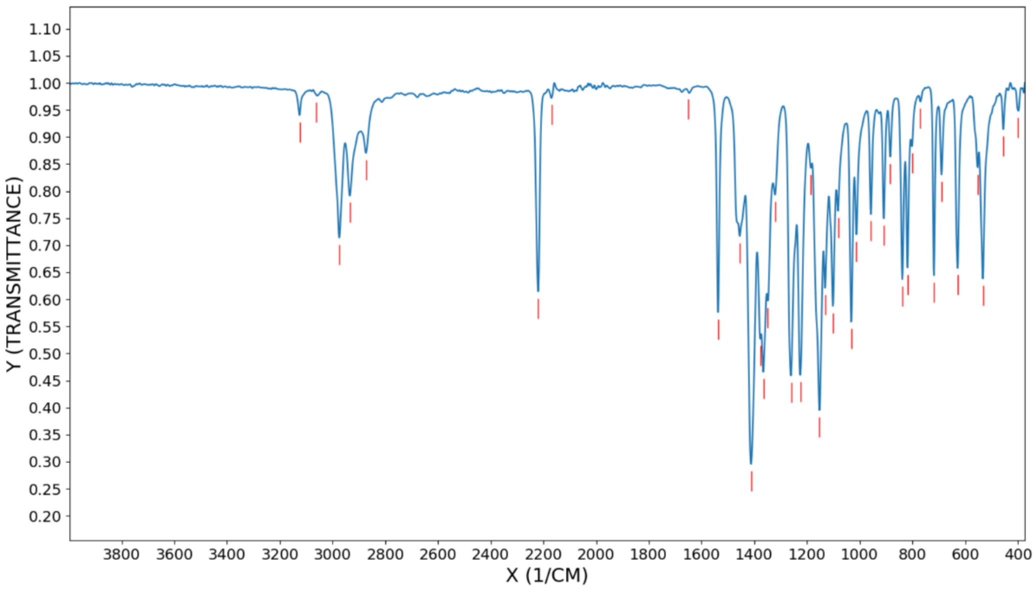

(*E*)-5-(3,3-Diisopropyltriaz-1-en-1-yl)-1-ethyl-1*H*-pyrazole-4-carbonitrile (13d)

CHMO:0000593 | 1H nuclear magnetic resonance spectroscopy (1H NMR)

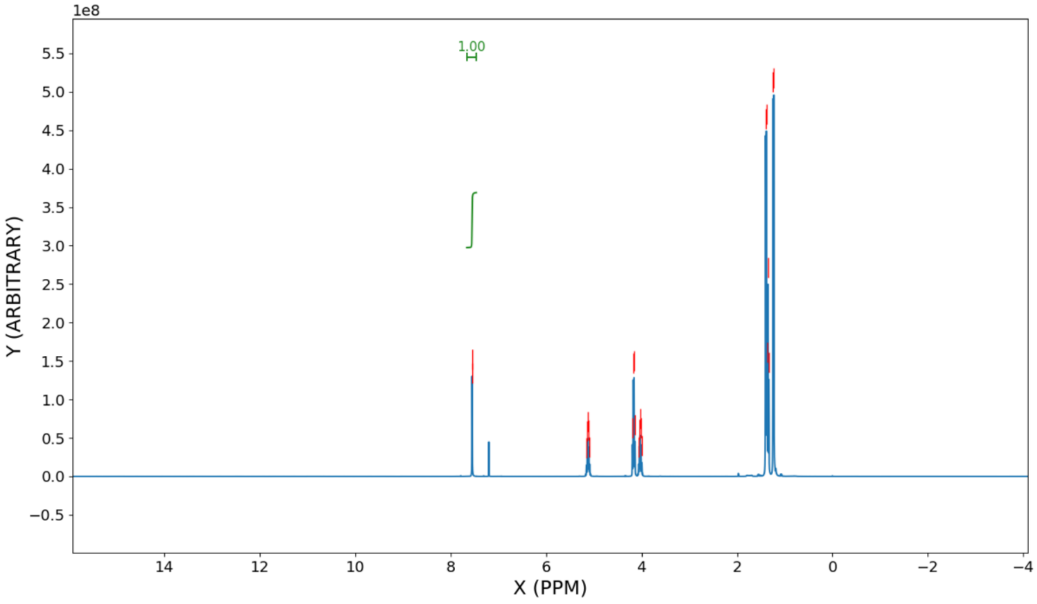

CHMO:0000595 | 13C nuclear magnetic resonance spectroscopy (13C NMR)

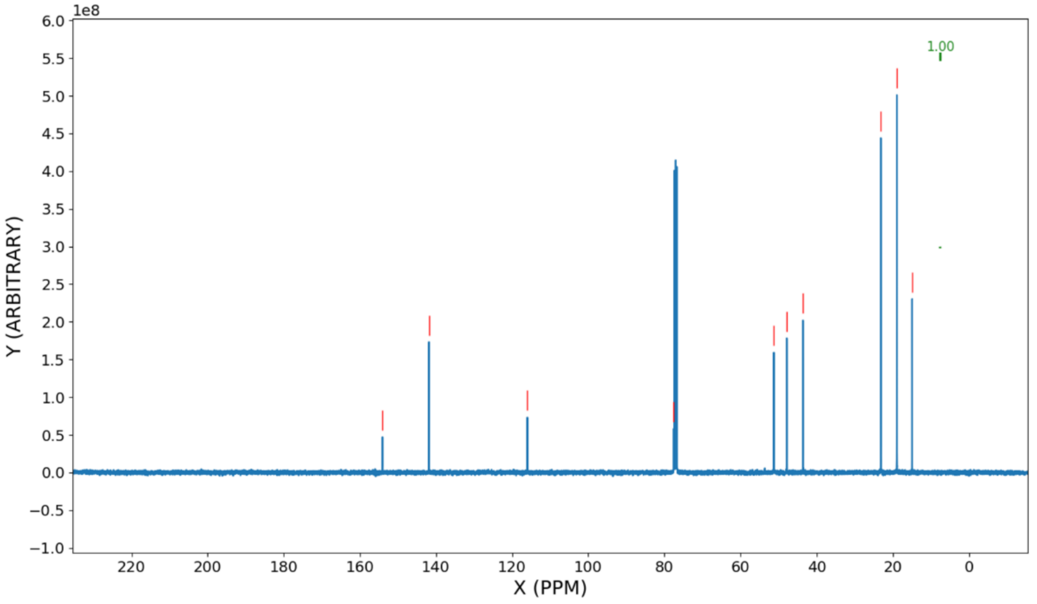

CHMO:0000563 | fast-atom bombardment mass spectrometry (FABMS)

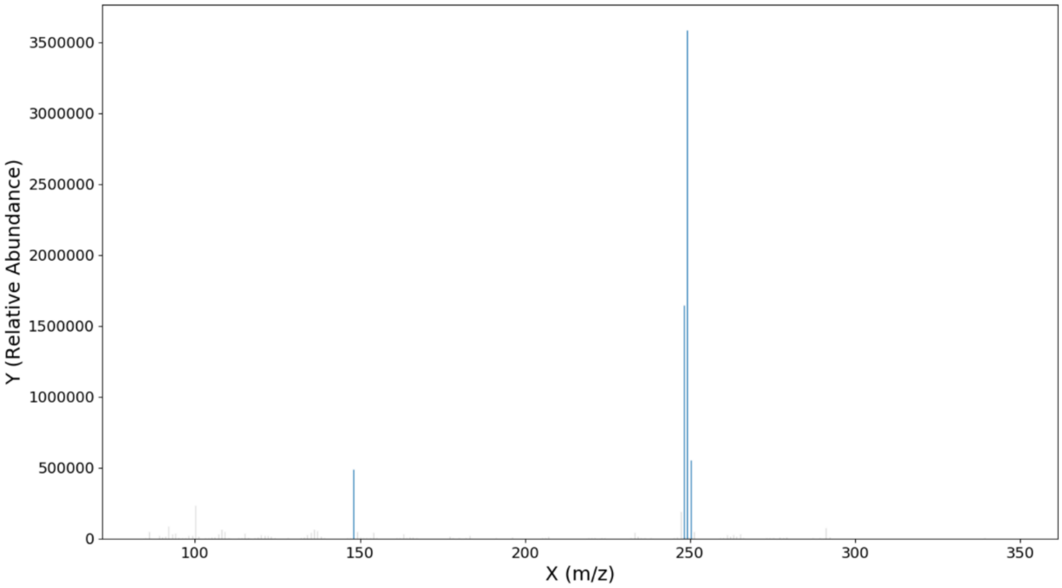

CHMO:0000630 | infrared absorption spectroscopy (IR)

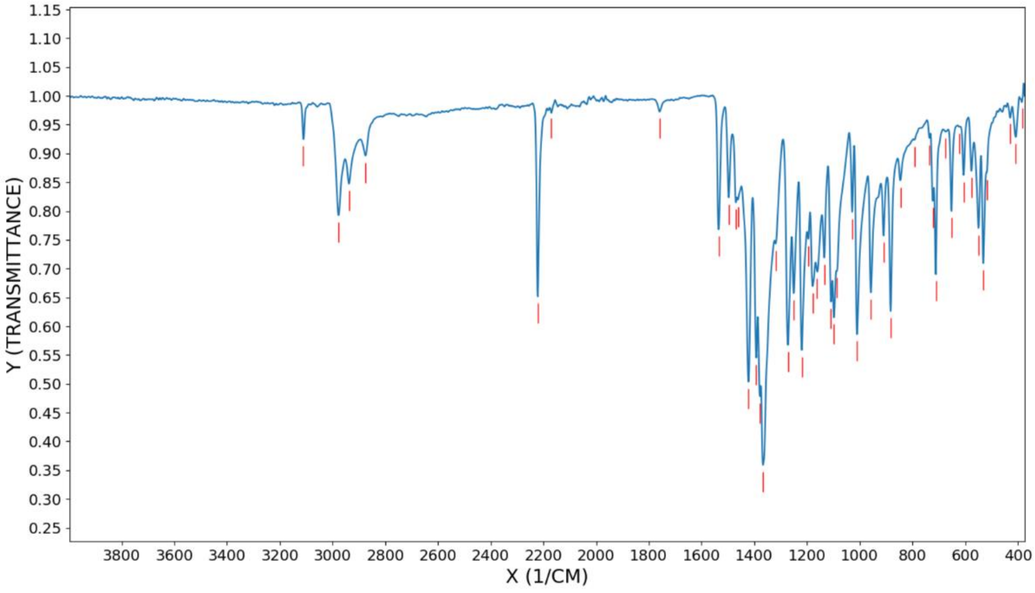

(*E*)-1-Cyclopentyl-3-(3,3-diisopropyltriaz-1-en-1-yl)-1*H*-pyrazole-4-carbonitrile (12e)

CHMO:0000593 | 1H nuclear magnetic resonance spectroscopy (1H NMR)

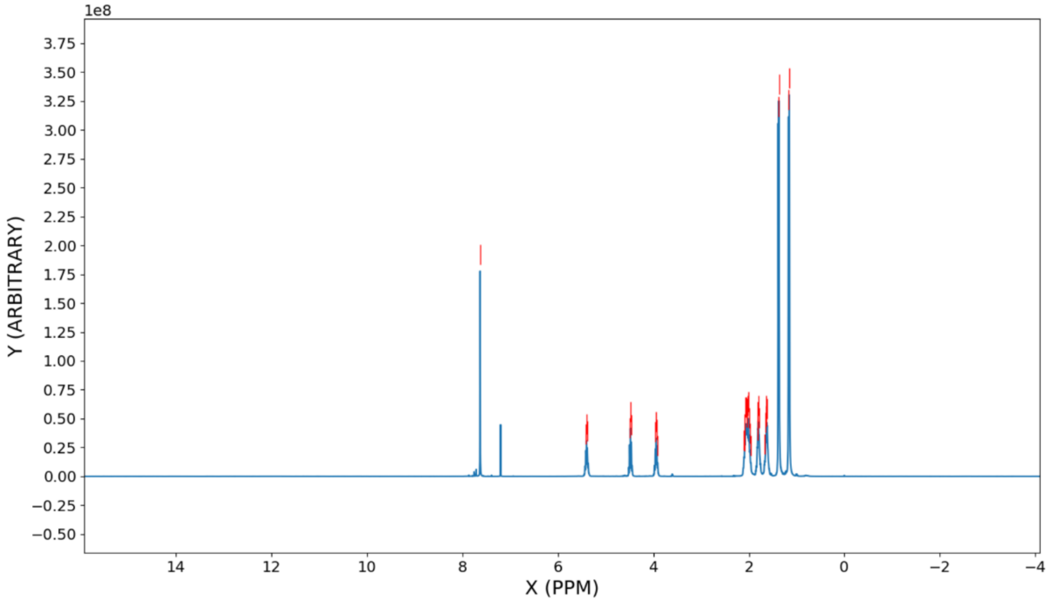

CHMO:0000595 | 13C nuclear magnetic resonance spectroscopy (13C NMR)

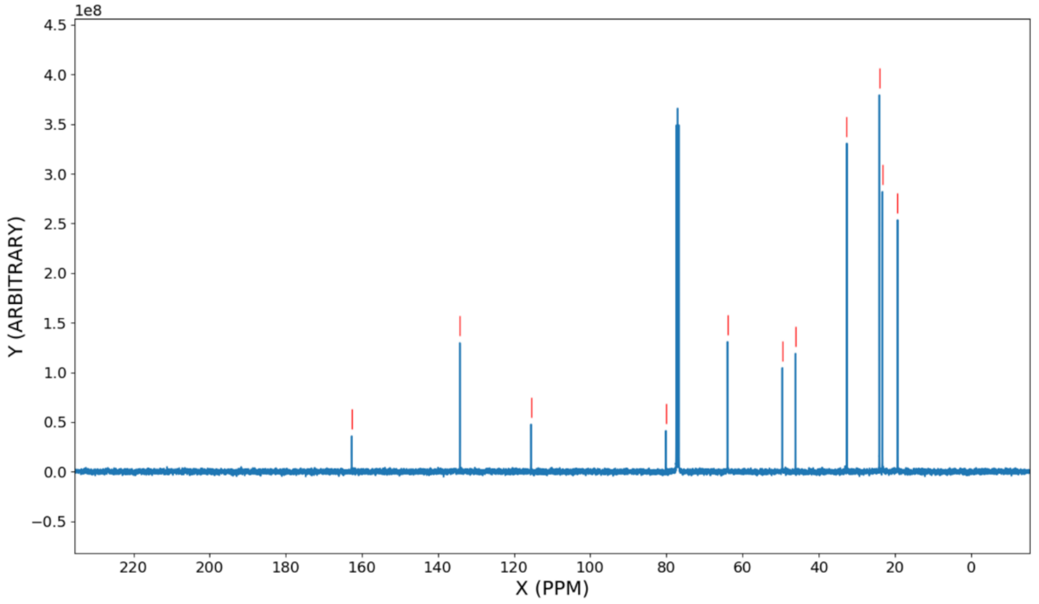

CHMO:0000563 | fast-atom bombardment mass spectrometry (FABMS)

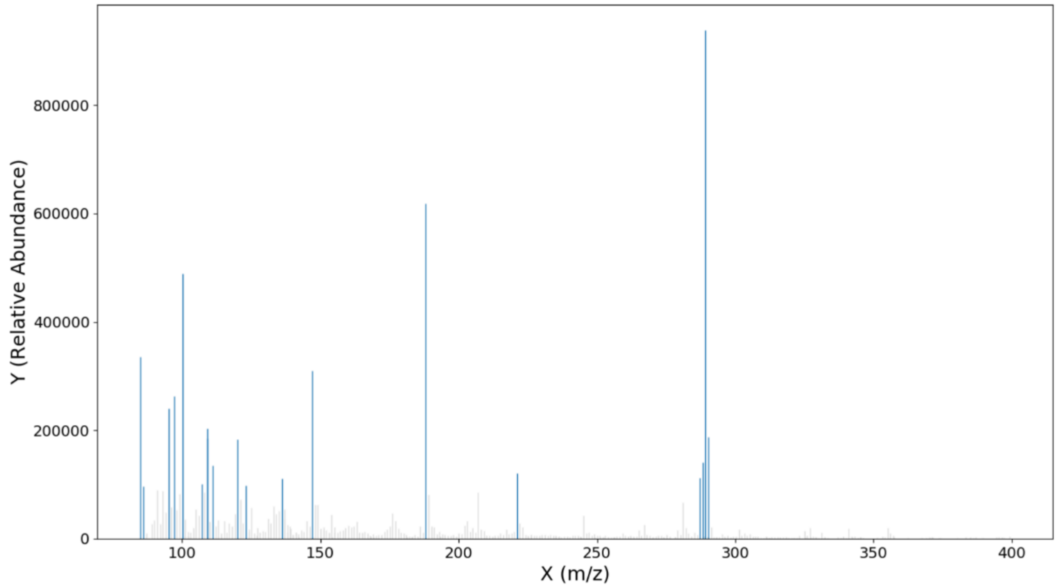

CHMO:0000630 | infrared absorption spectroscopy (IR)

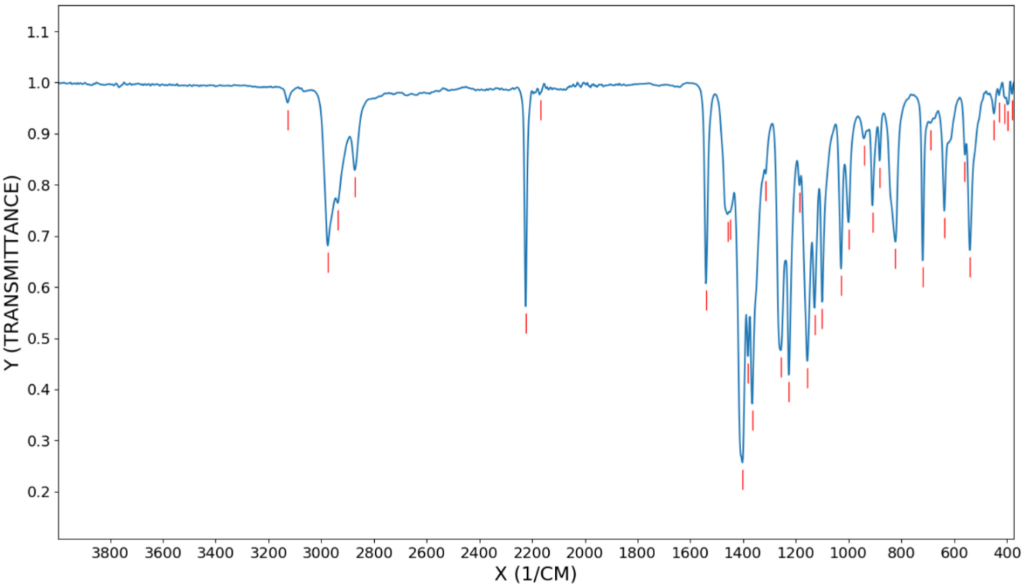

(*E*)-1-Cyclopentyl-5-(3,3-diisopropyltriaz-1-en-1-yl)-1*H*-pyrazole-4-carbonitrile (13e)

CHMO:0000593 | 1H nuclear magnetic resonance spectroscopy (1H NMR)

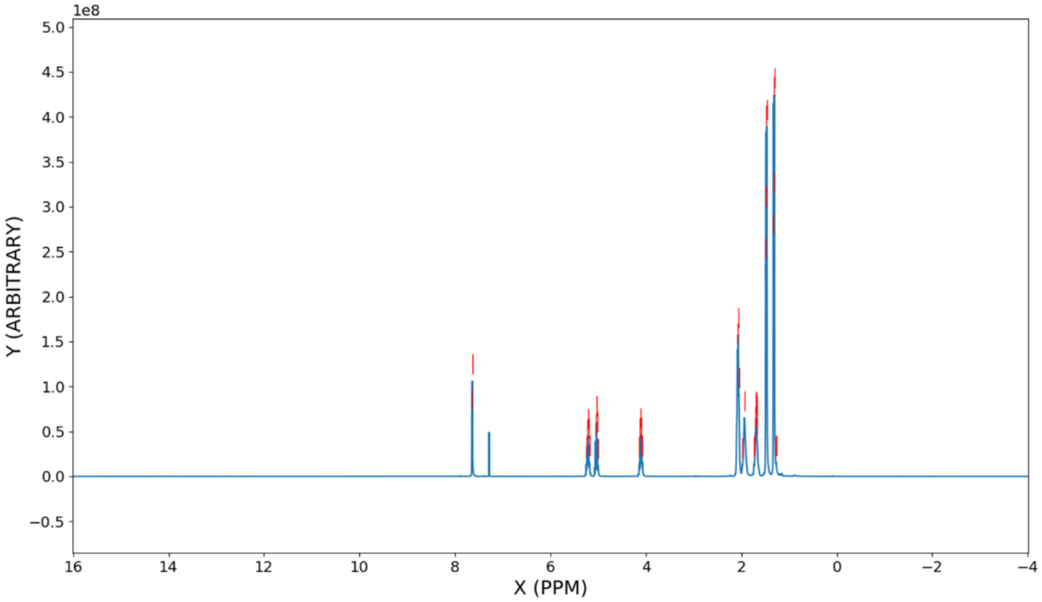

CHMO:0000595 | 13C nuclear magnetic resonance spectroscopy (13C NMR)

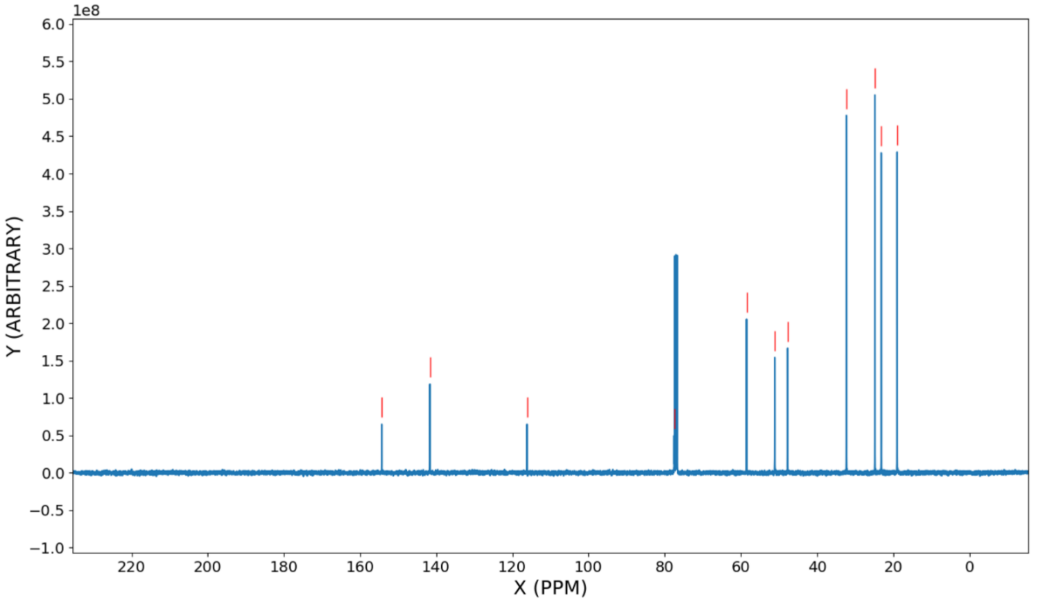

CHMO:0000563 | fast-atom bombardment mass spectrometry (FABMS)

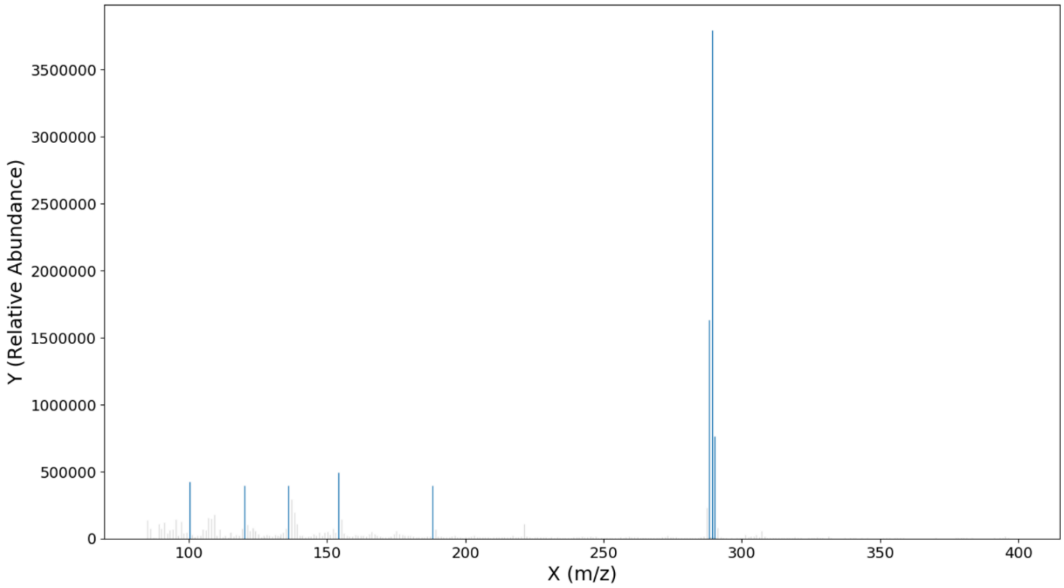

CHMO:0000630 | infrared absorption spectroscopy (IR)

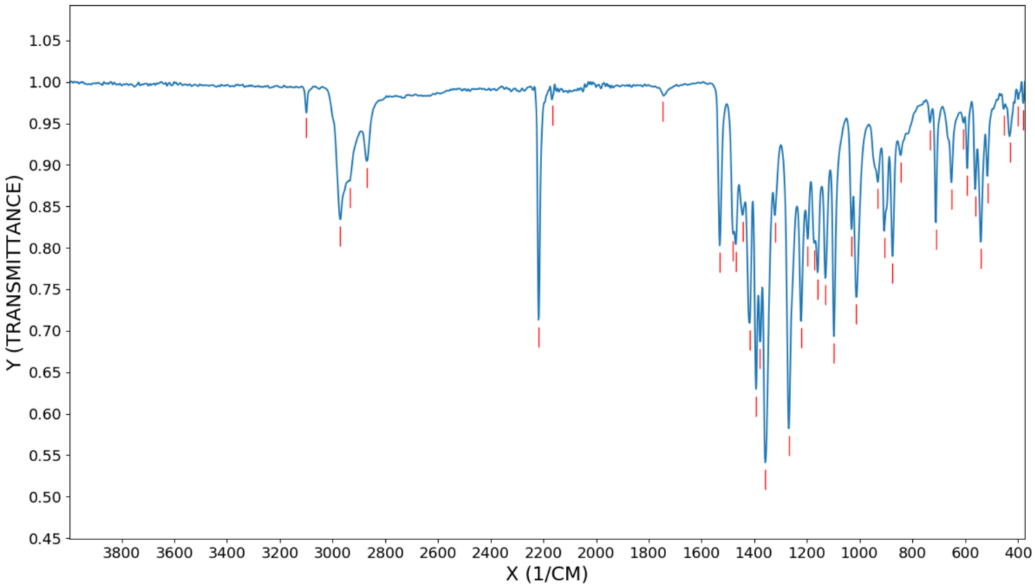

(*E*)-3-(3,3-Diisopropyltriaz-1-en-1-yl)-1-isobutyl-1*H*-pyrazole-4-carbonitrile (12f)

CHMO:0000593 | 1H nuclear magnetic resonance spectroscopy (1H NMR)

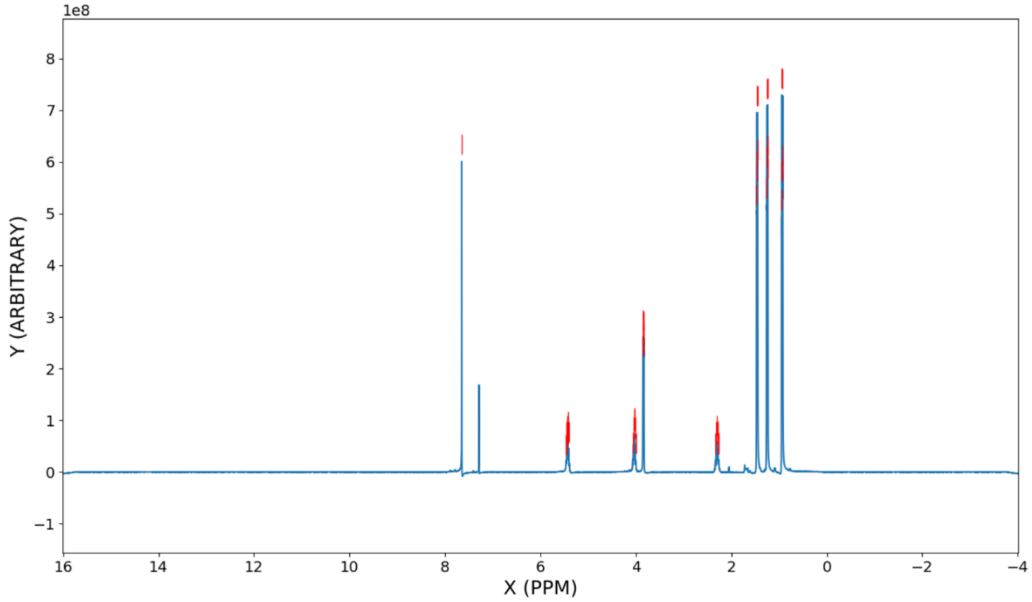

CHMO:0000595 | 13C nuclear magnetic resonance spectroscopy (13C NMR)

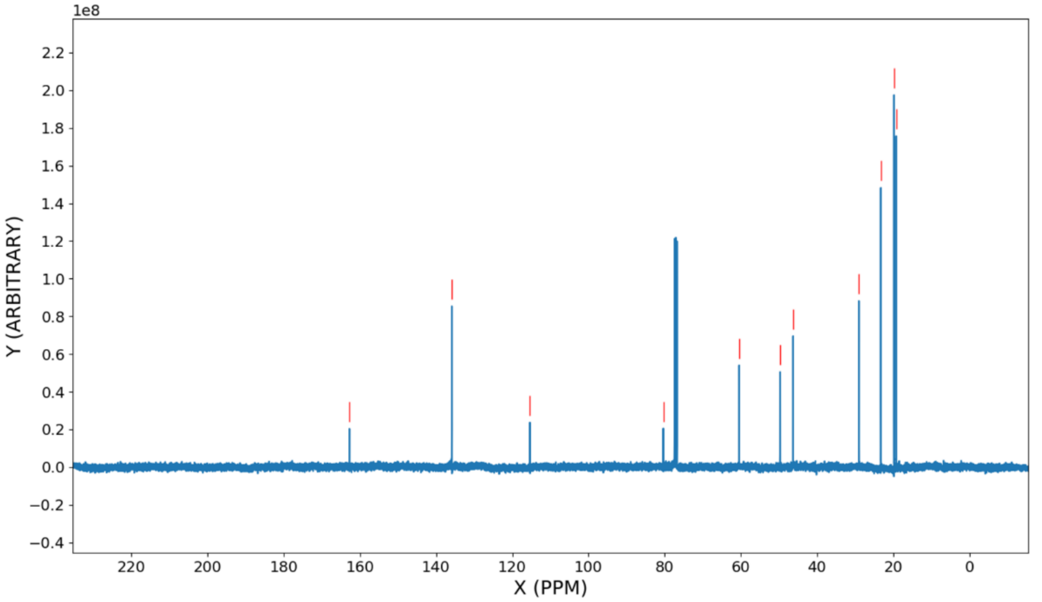

CHMO:0000563 | fast-atom bombardment mass spectrometry (FABMS)

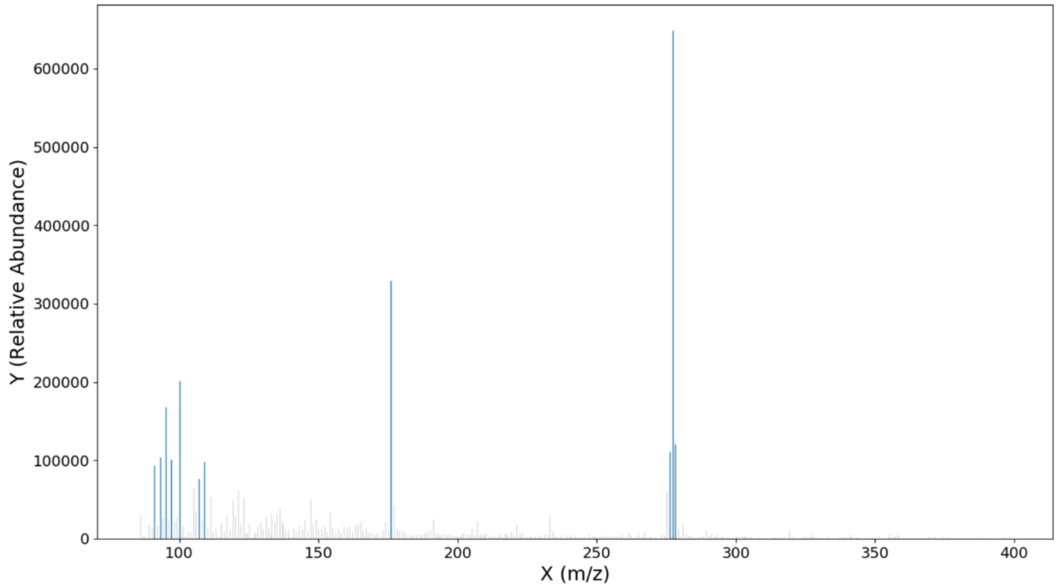

CHMO:0000630 | infrared absorption spectroscopy (IR)

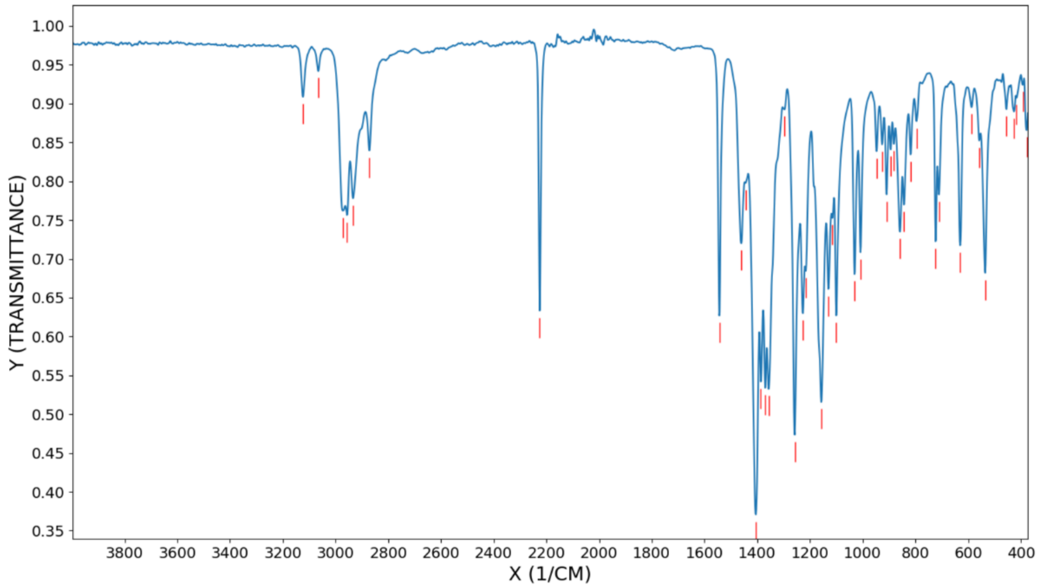

(*E*)-5-(3,3-Diisopropyltriaz-1-en-1-yl)-1-isobutyl-1*H*-pyrazole-4-carbonitrile (13f)

CHMO:0000593 | 1H nuclear magnetic resonance spectroscopy (1H NMR)

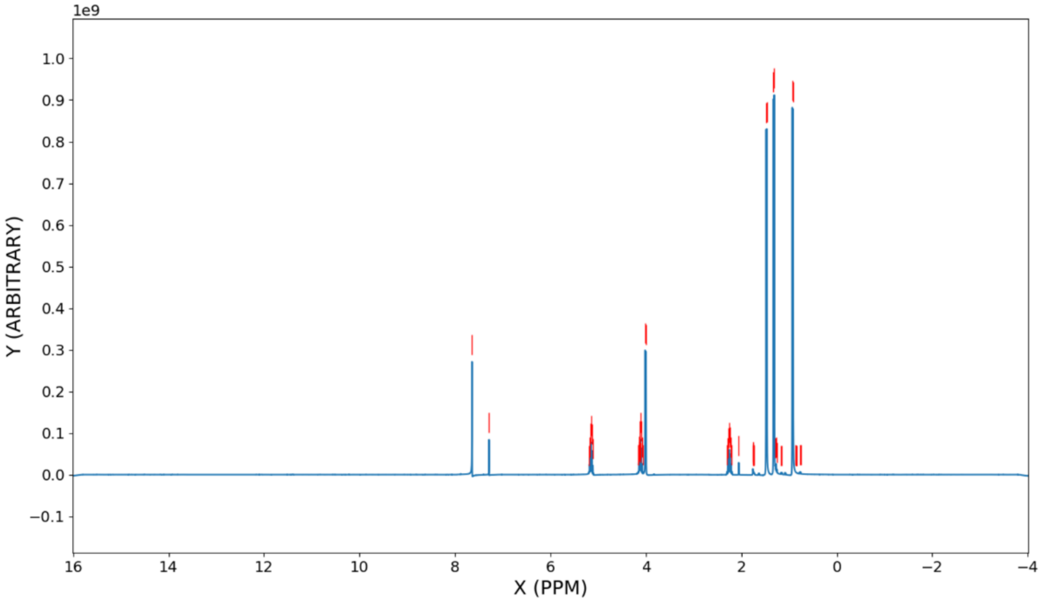

CHMO:0000595 | 13C nuclear magnetic resonance spectroscopy (13C NMR)

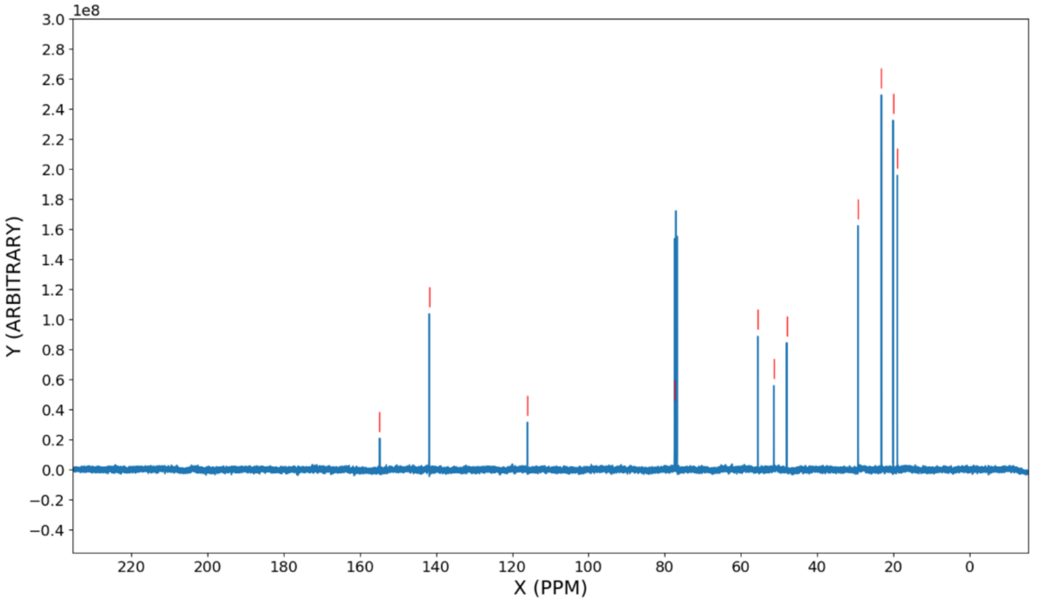

CHMO:0000563 | fast-atom bombardment mass spectrometry (FABMS)

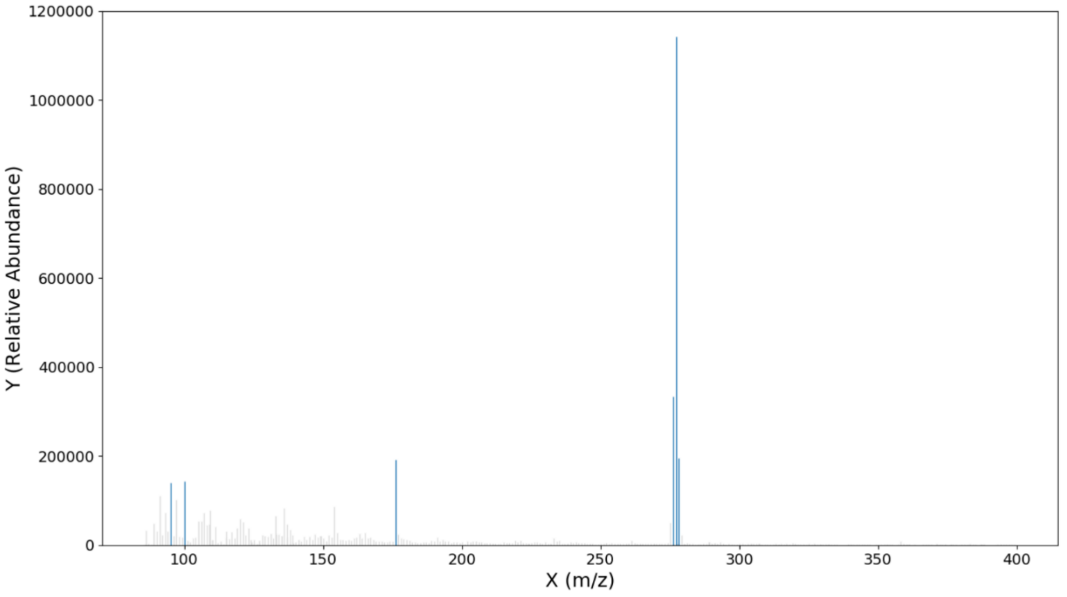

CHMO:0000630 | infrared absorption spectroscopy (IR)

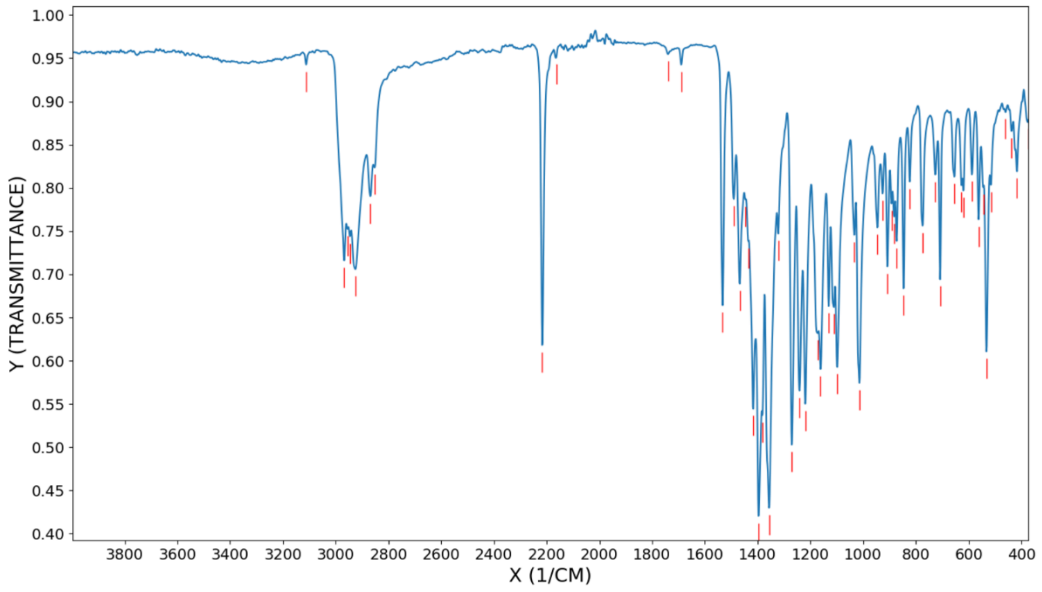

Ethyl (*E*)-2-(4-cyano-3-(3,3-diisopropyltriaz-1-en-1-yl)-1*H*-pyrazol-1-yl)acetate (12g)

CHMO:0000593 | 1H nuclear magnetic resonance spectroscopy (1H NMR)

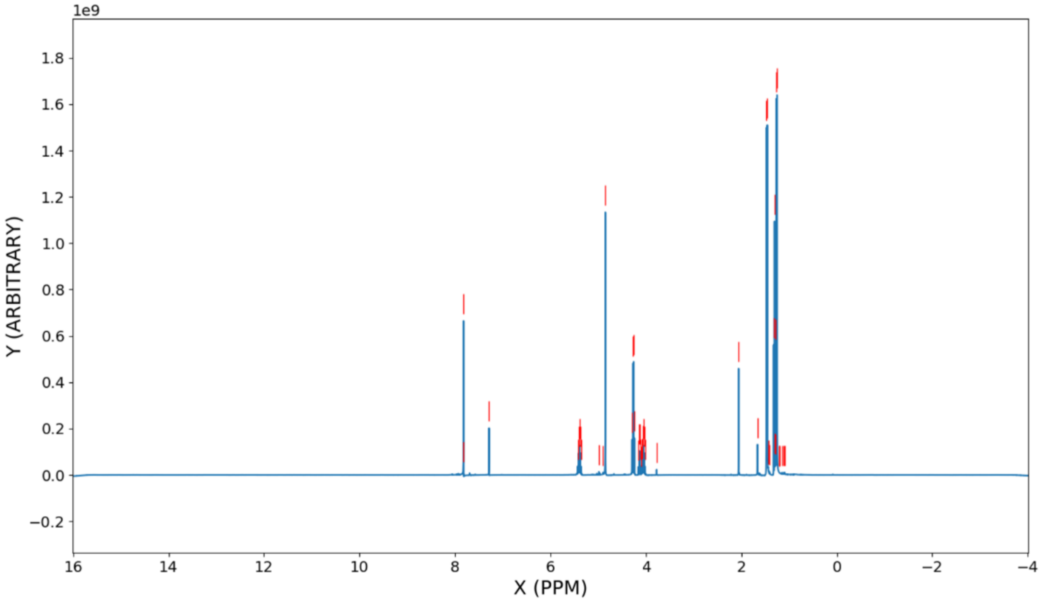

CHMO:0000595 | 13C nuclear magnetic resonance spectroscopy (13C NMR)

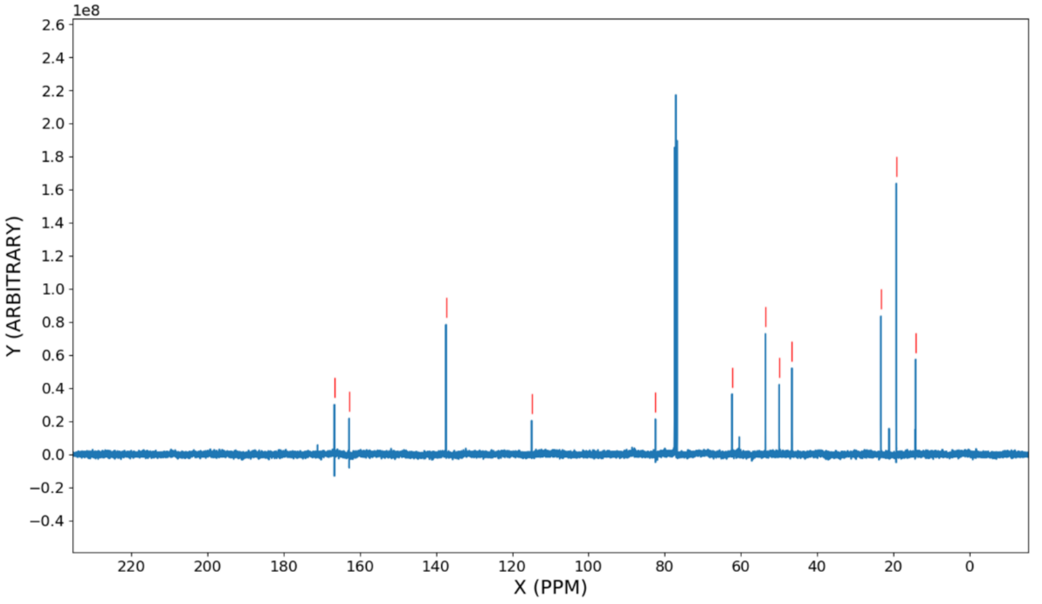

CHMO:0000563 | fast-atom bombardment mass spectrometry (FABMS)

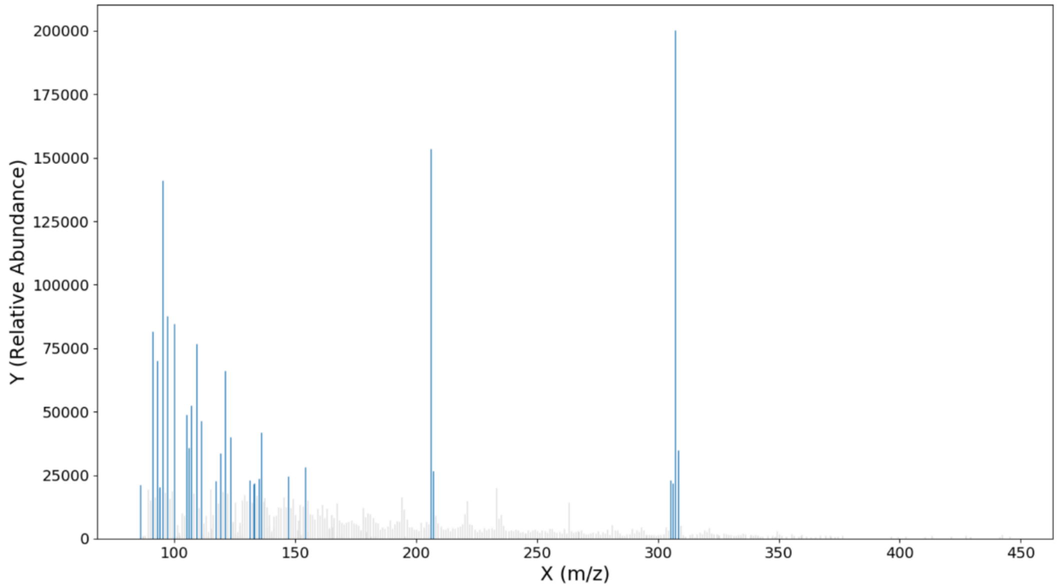

CHMO:0000630 | infrared absorption spectroscopy (IR)

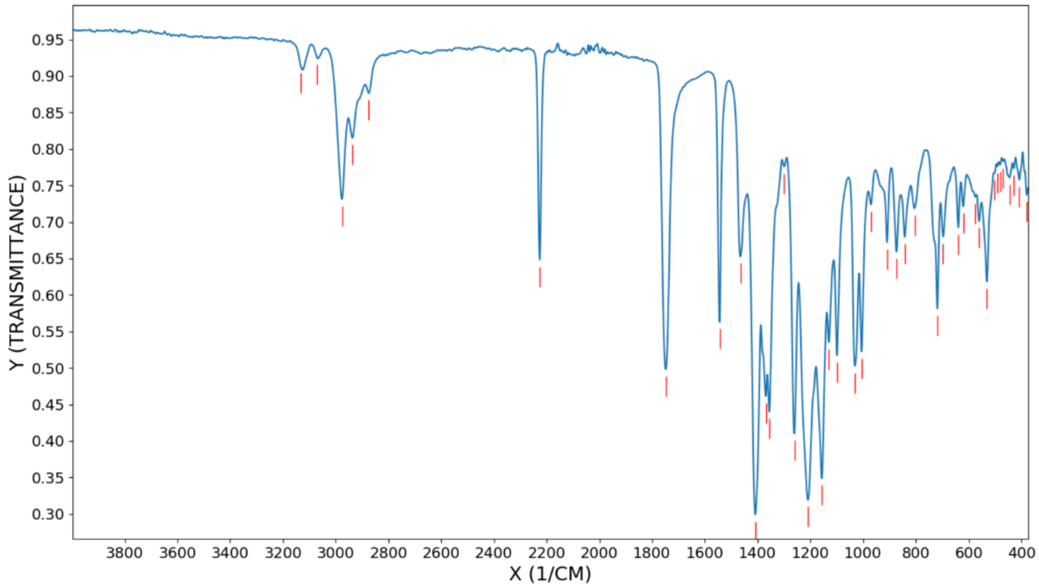

Ethyl (*E*)-2-(4-cyano-5-(3,3-diisopropyltriaz-1-en-1-yl)-1*H*-pyrazol-1-yl)acetate (13g)

CHMO:0000593 | 1H nuclear magnetic resonance spectroscopy (1H NMR)

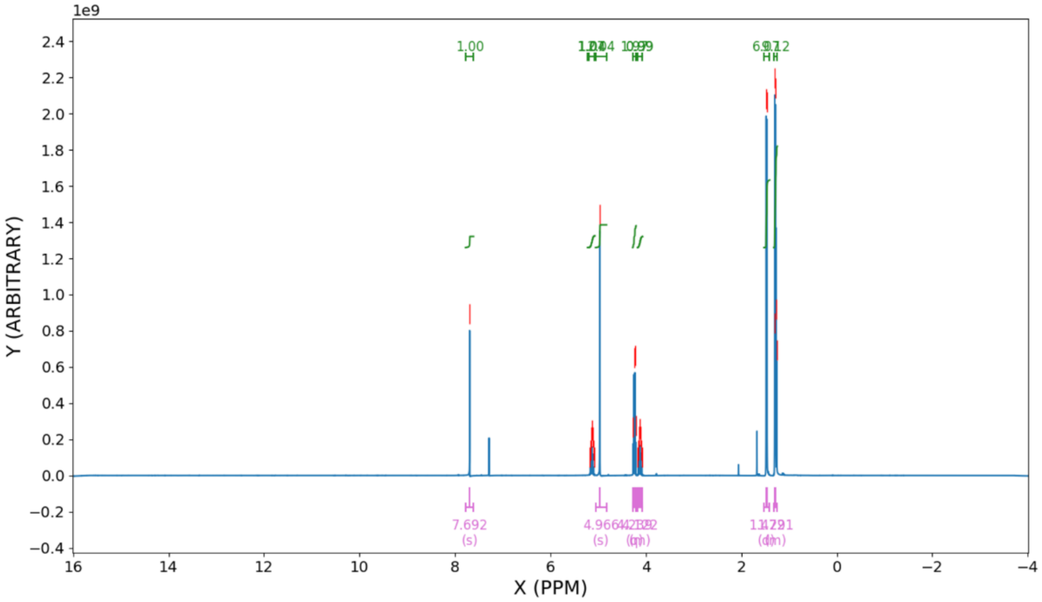

CHMO:0000595 | 13C nuclear magnetic resonance spectroscopy (13C NMR)

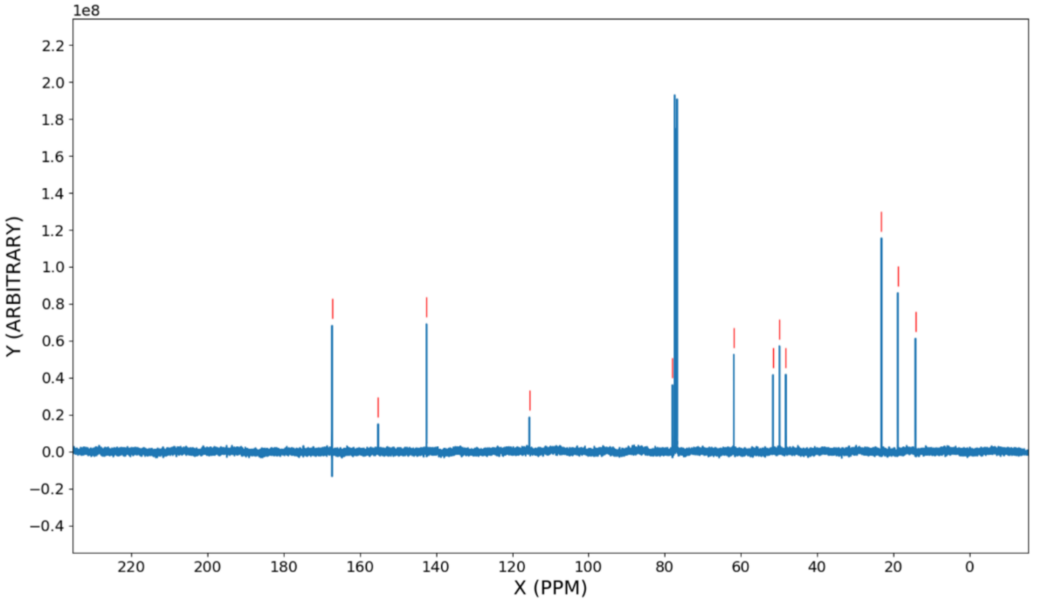

CHMO:0000563 | fast-atom bombardment mass spectrometry (FABMS)

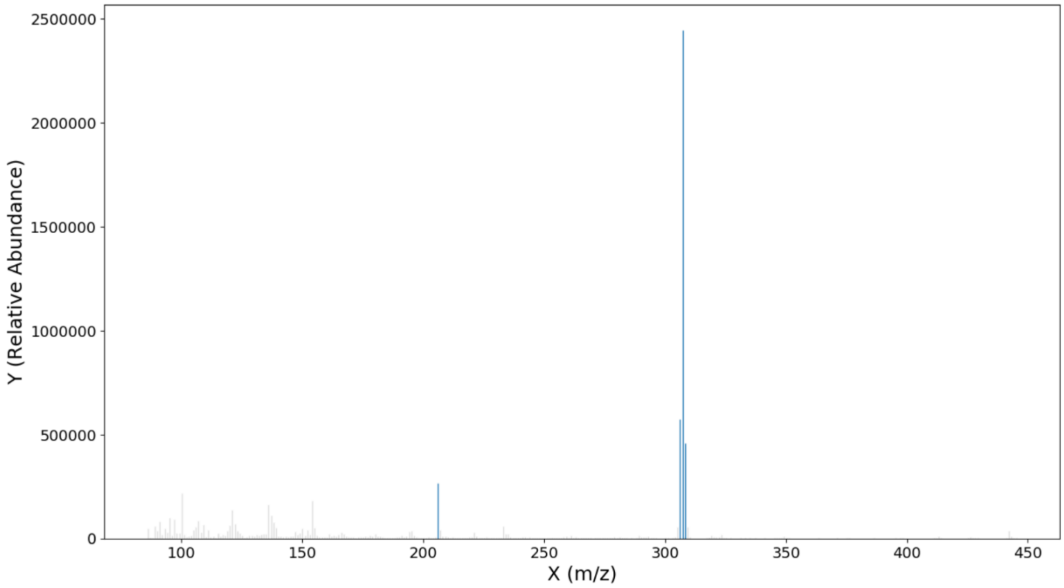

CHMO:0000630 | infrared absorption spectroscopy (IR)

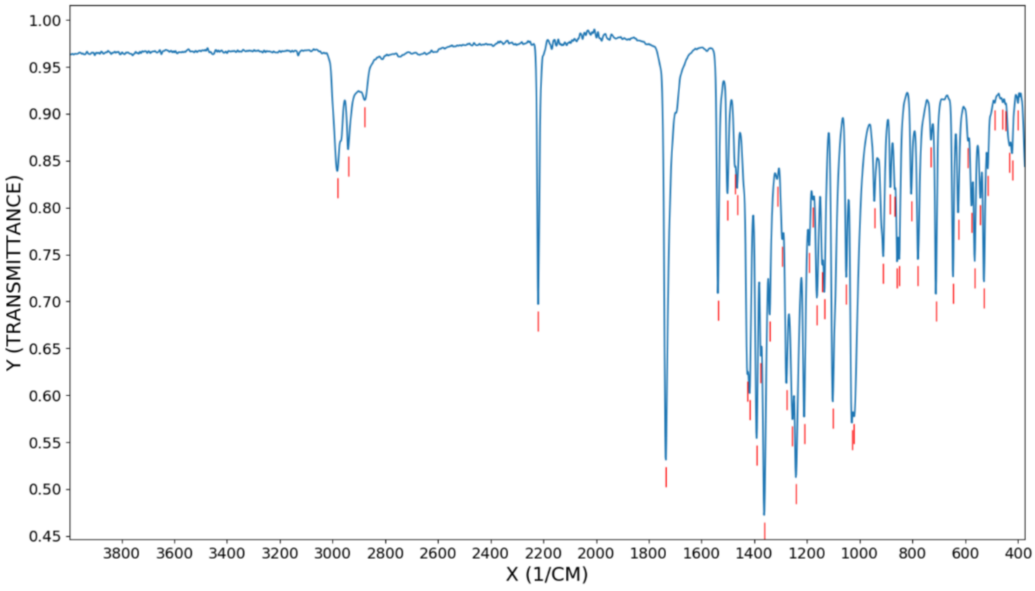

(*E*)-1-(4-Bromobenzyl)-3-(3,3-diisopropyltriaz-1-en-1-yl)-1*H*-pyrazole-4-carbonitrile (12h)

CHMO:0000593 | 1H nuclear magnetic resonance spectroscopy (1H NMR)

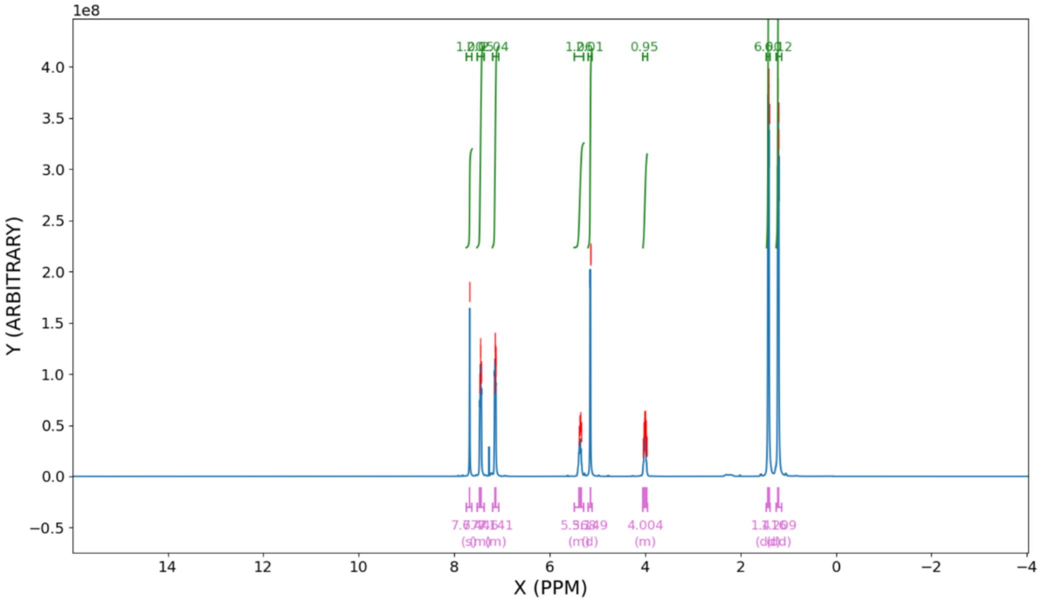

CHMO:0000595 | 13C nuclear magnetic resonance spectroscopy (13C NMR)

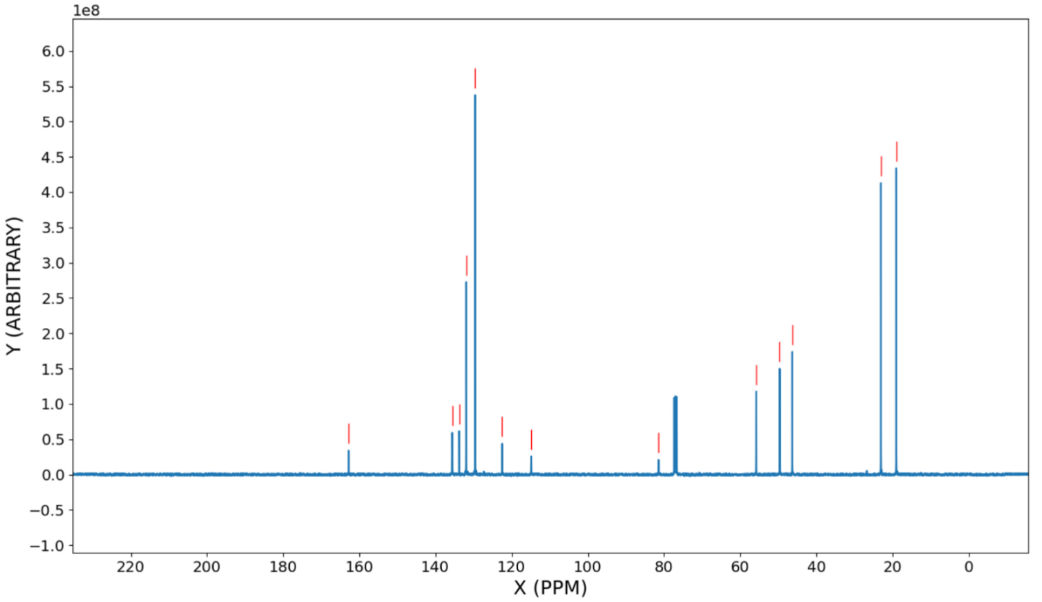

CHMO:0000470 | mass spectrometry (MS)

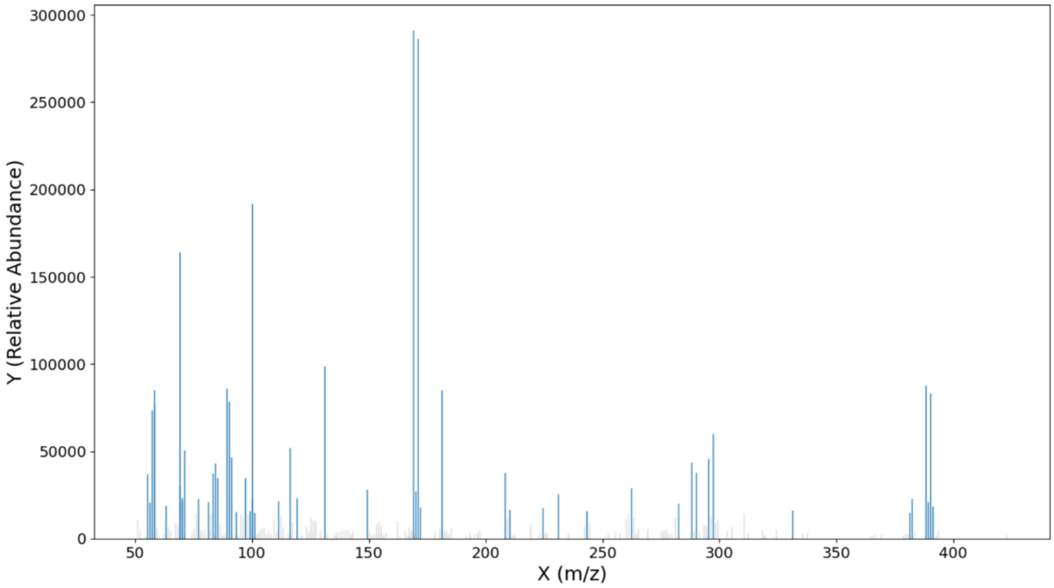

(*E*)-1-(4-Bromobenzyl)-5-(3,3-diisopropyltriaz-1-en-1-yl)-1*H*-pyrazole-4-carbonitrile (13h)

CHMO:0000593 | 1H nuclear magnetic resonance spectroscopy (1H NMR)

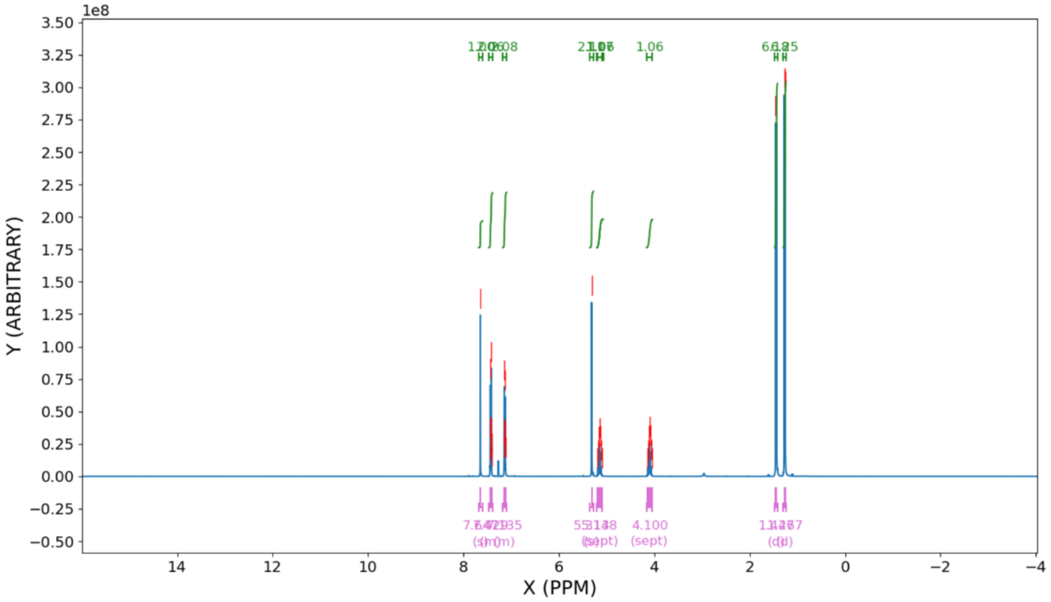

CHMO:0000595 | 13C nuclear magnetic resonance spectroscopy (13C NMR)

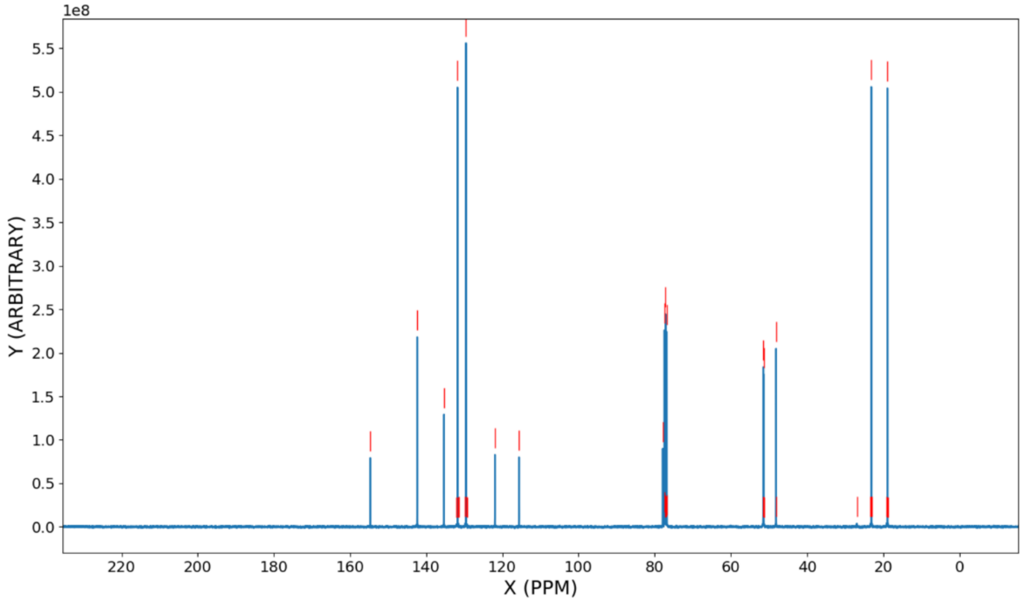

CHMO:0000470 | mass spectrometry (MS)

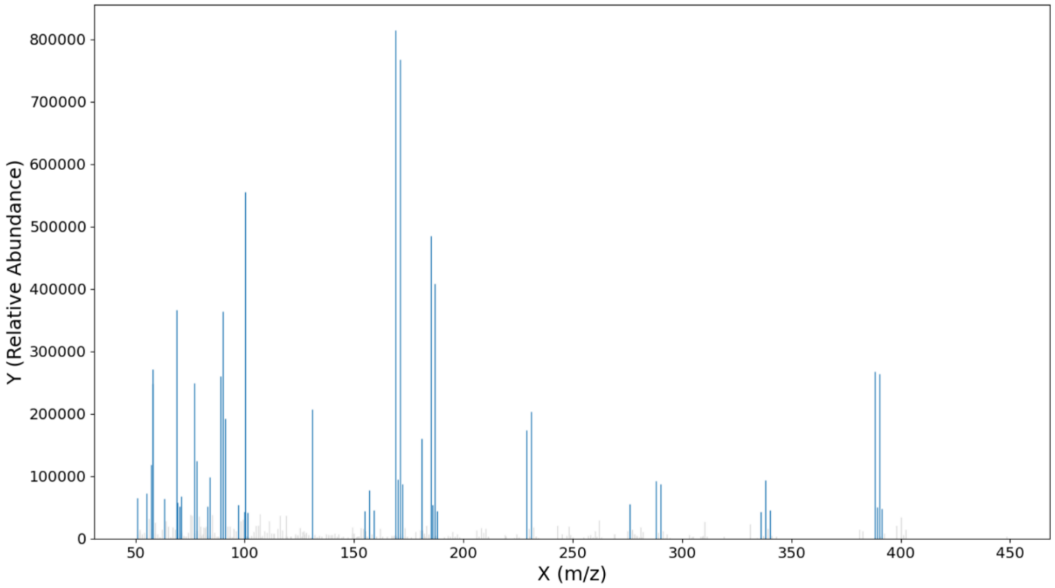

(*E*)-*N*-((1-Benzyl-3-(3,3-diisopropyltriaz-1-en-1-yl)-1*H*-pyrazol-4-yl)methyl)acetamido (9a)

CHMO:0000593 | 1H nuclear magnetic resonance spectroscopy (1H NMR)

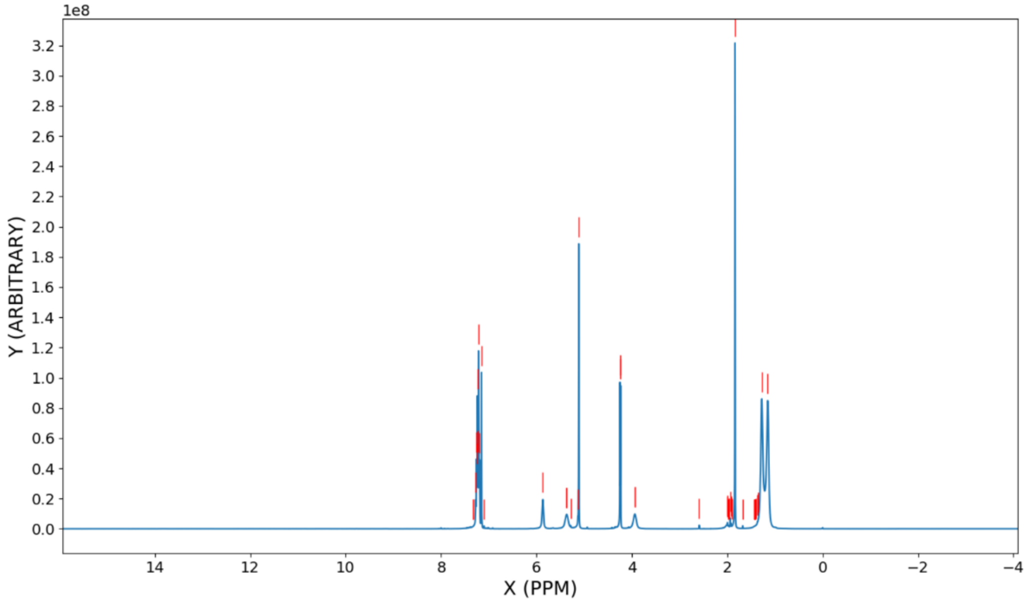

CHMO:0000595 | 13C nuclear magnetic resonance spectroscopy (13C NMR)

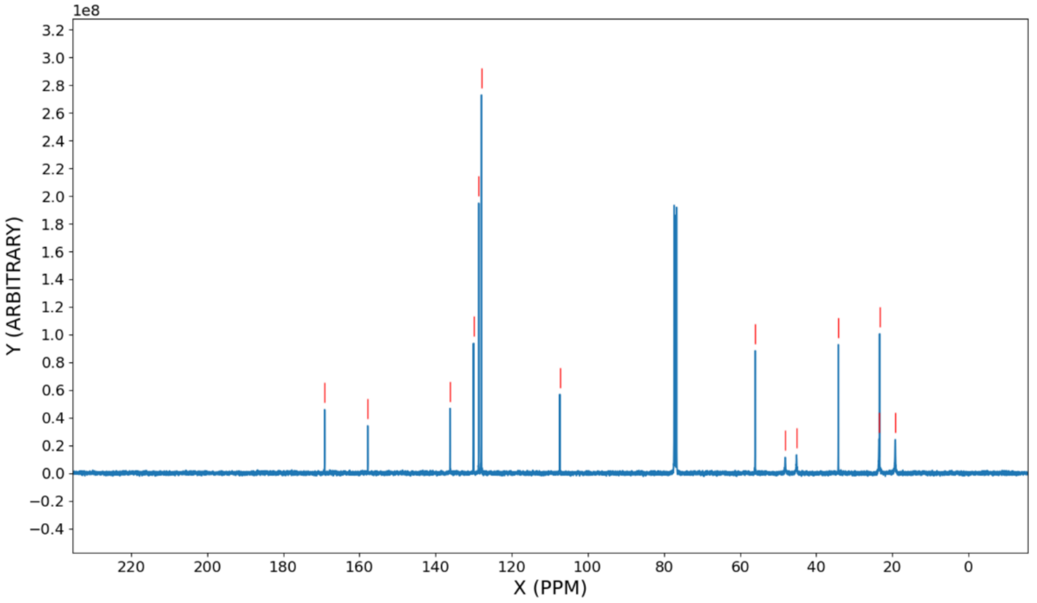

CHMO:0000563 | fast-atom bombardment mass spectrometry (FABMS)

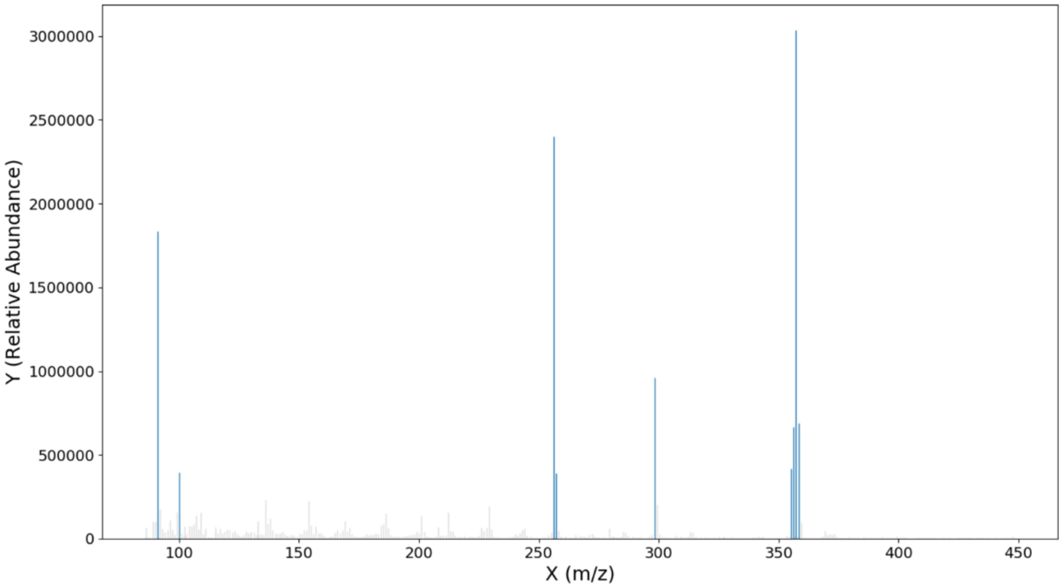

CHMO:0000630 | infrared absorption spectroscopy (IR)

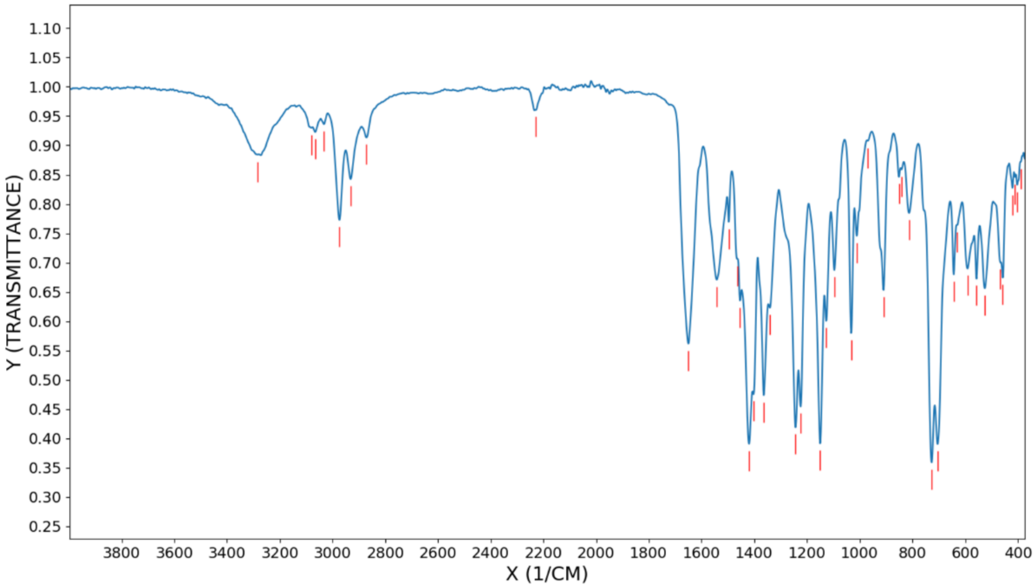

(*E*)-*N*-((1-Benzyl-3-(3,3-diisopropyltriaz-1-en-1-yl)-1*H*-pyrazol-4-yl)methyl)benzamide (9b)

CHMO:0000593 | 1H nuclear magnetic resonance spectroscopy (1H NMR)

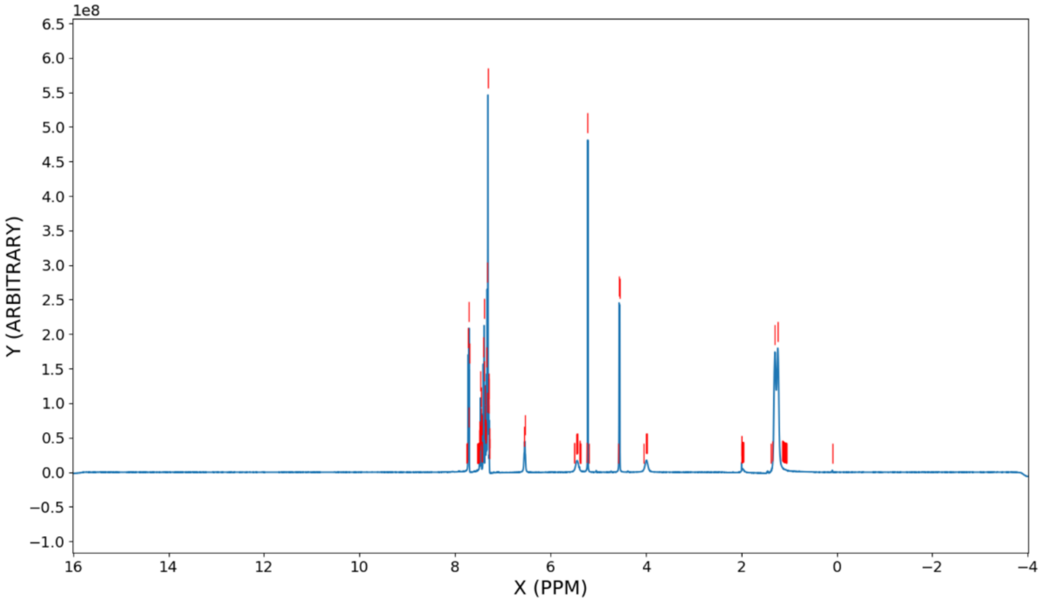

CHMO:0000595 | 13C nuclear magnetic resonance spectroscopy (13C NMR)

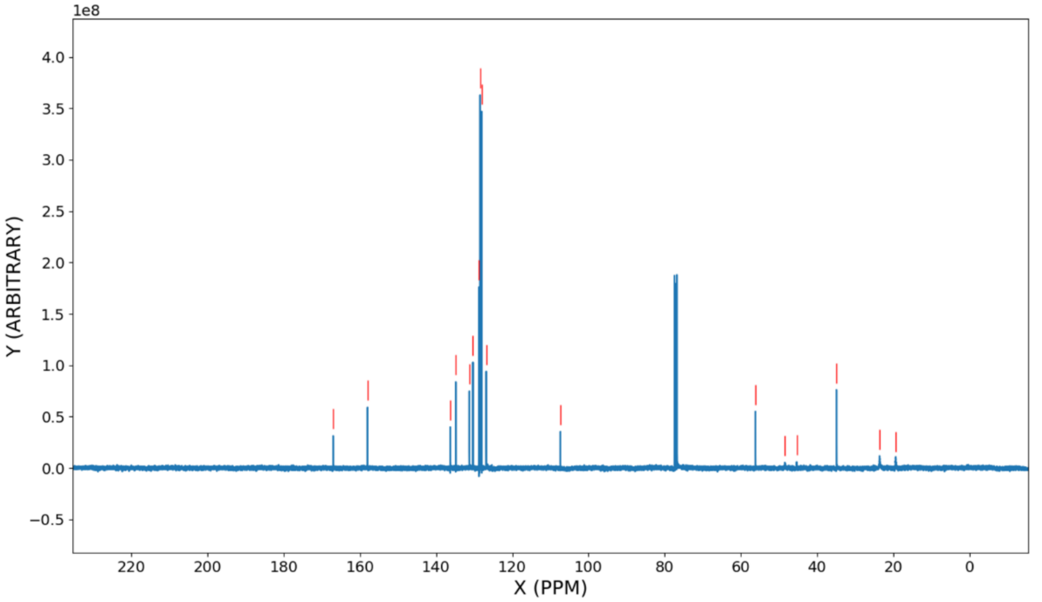

CHMO:0000563 | fast-atom bombardment mass spectrometry (FABMS)

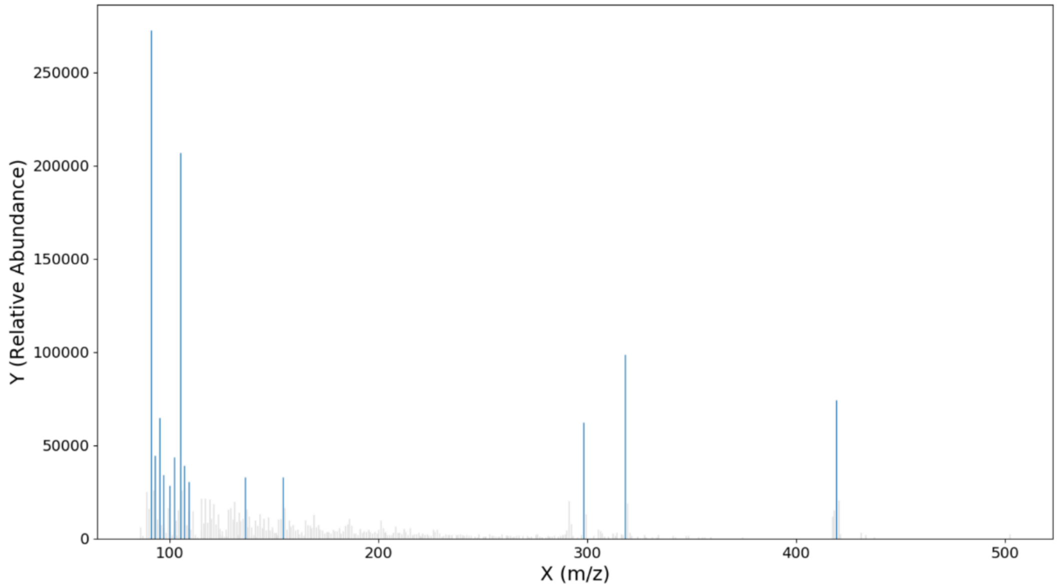

CHMO:0000630 | infrared absorption spectroscopy (IR)

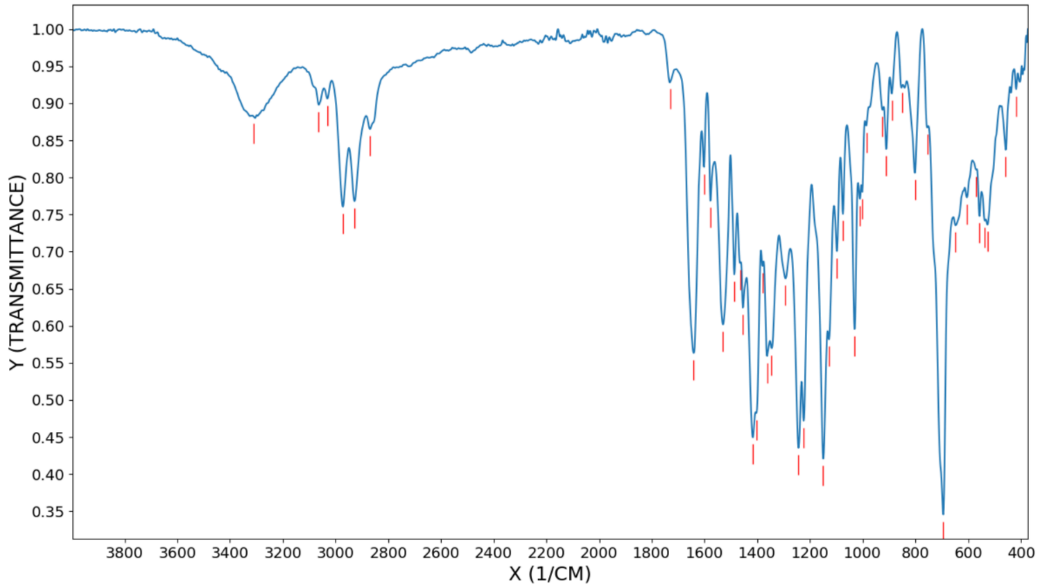

(*E*)-*N*-((3-(3,3-Diisopropyltriaz-1-en-1-yl)-1-(4-methylbenzyl)-1*H*-pyrazol-4-yl)methyl)-3-methylbutanamide (9c)

CHMO:0000593 | 1H nuclear magnetic resonance spectroscopy (1H NMR)

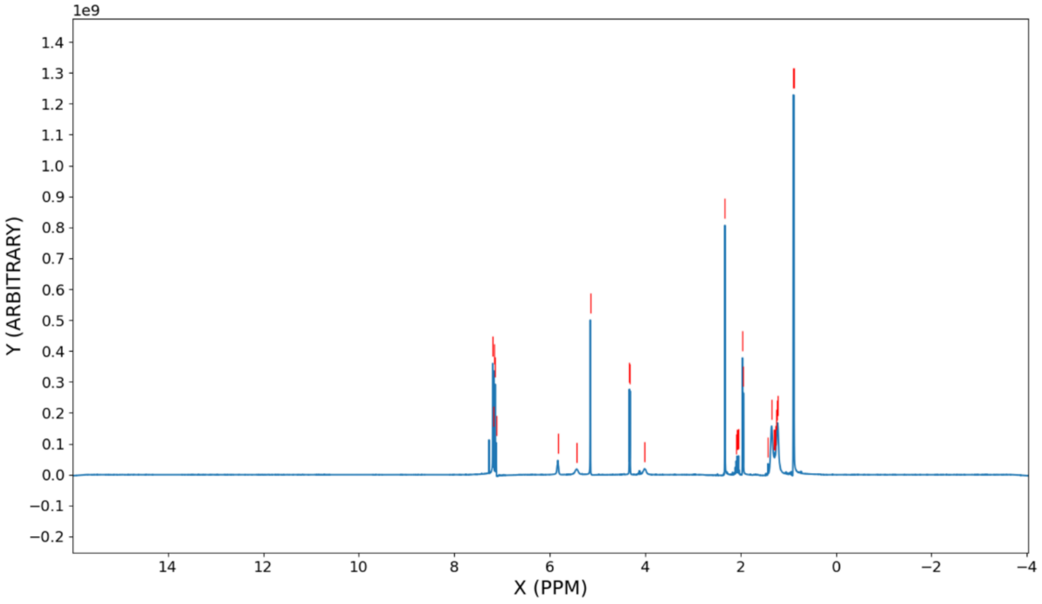

CHMO:0000595 | 13C nuclear magnetic resonance spectroscopy (13C NMR)

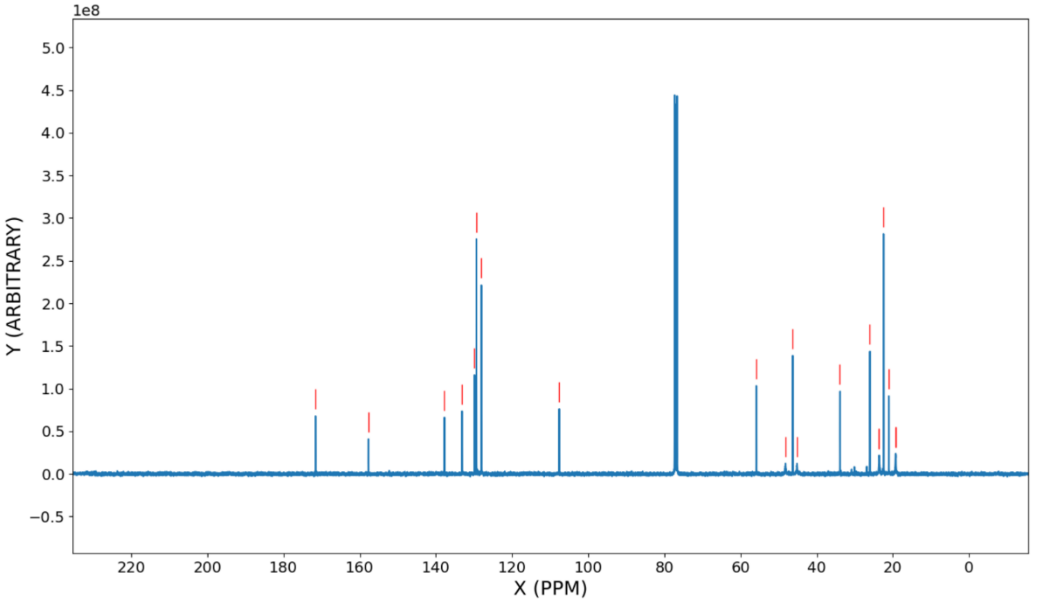

CHMO:0000563 | fast-atom bombardment mass spectrometry (FABMS)

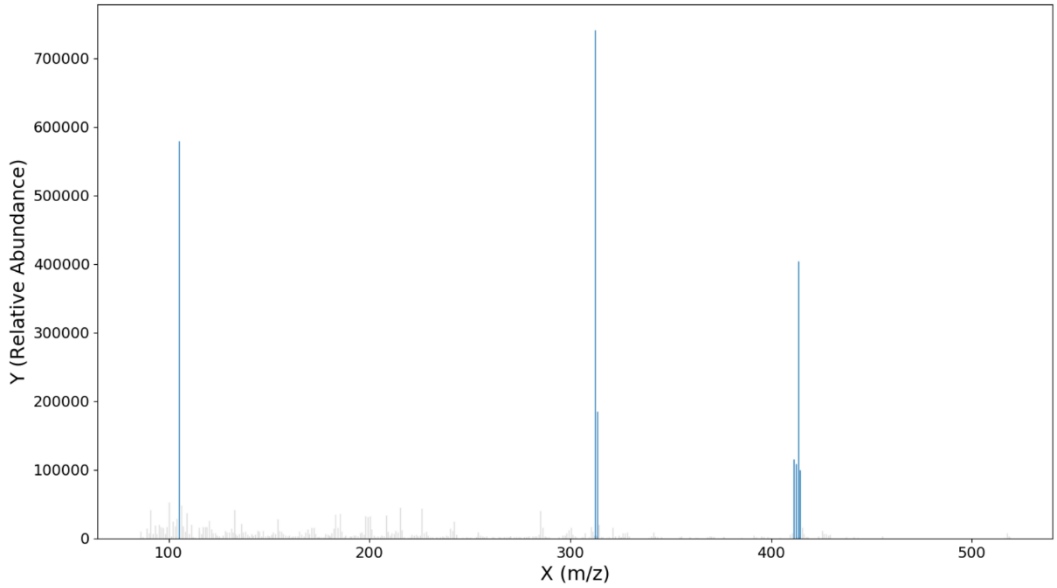

CHMO:0000630 | infrared absorption spectroscopy (IR)

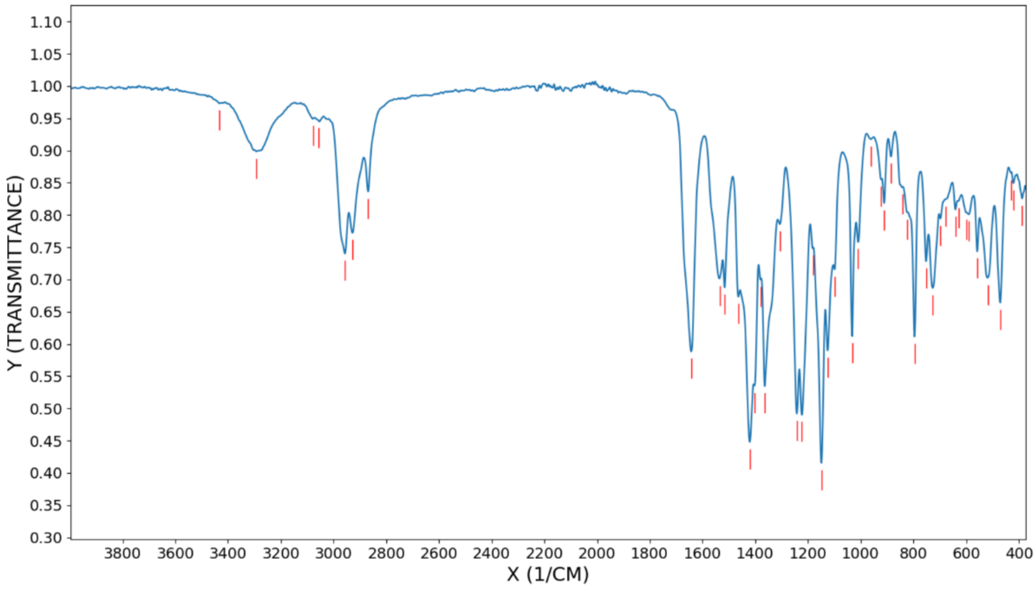

(*E*)-*N*-((1-(3,5-Difluorobenzyl)-3-(3,3-diisopropyltriaz-1-en-1-yl)-1*H*-pyrazol-4-yl)methyl)acetamido (9d)

CHMO:0000593 | 1H nuclear magnetic resonance spectroscopy (1H NMR)

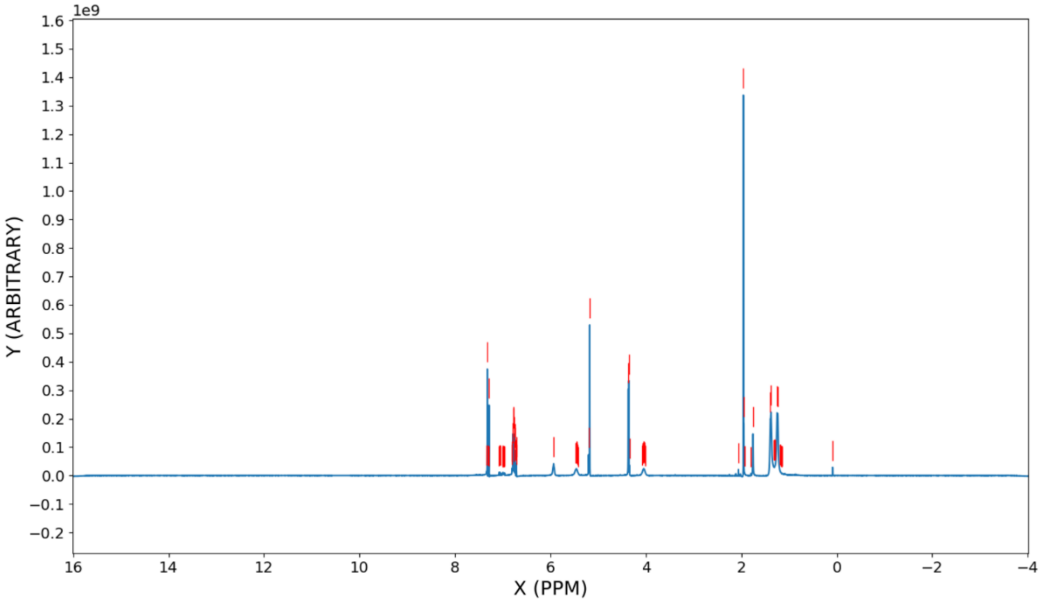

CHMO:0000595 | 13C nuclear magnetic resonance spectroscopy (13C NMR)

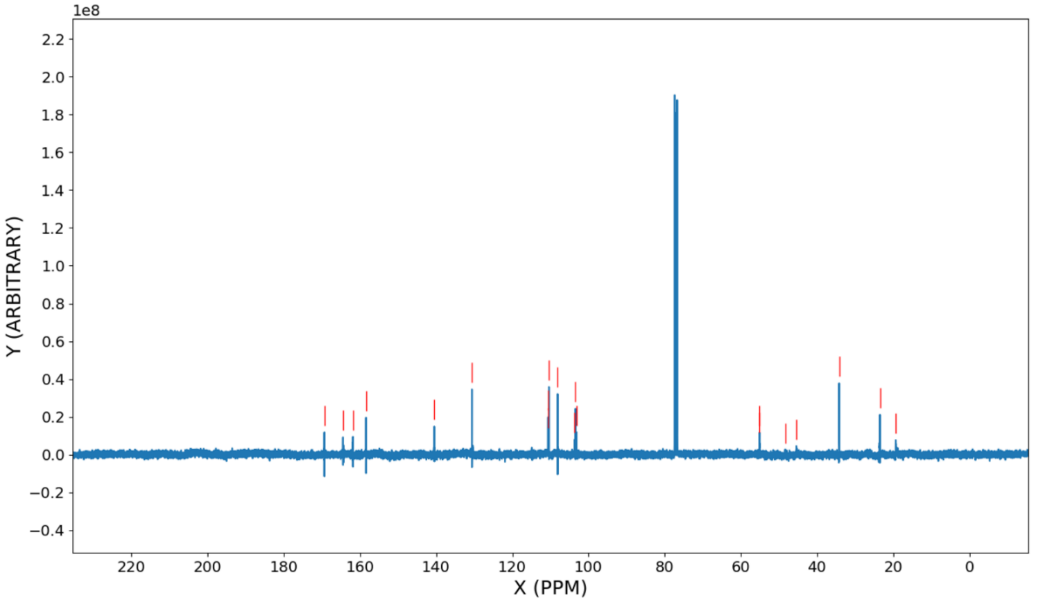

CHMO:0000597 | 19F nuclear magnetic resonance spectroscopy (19F NMR)

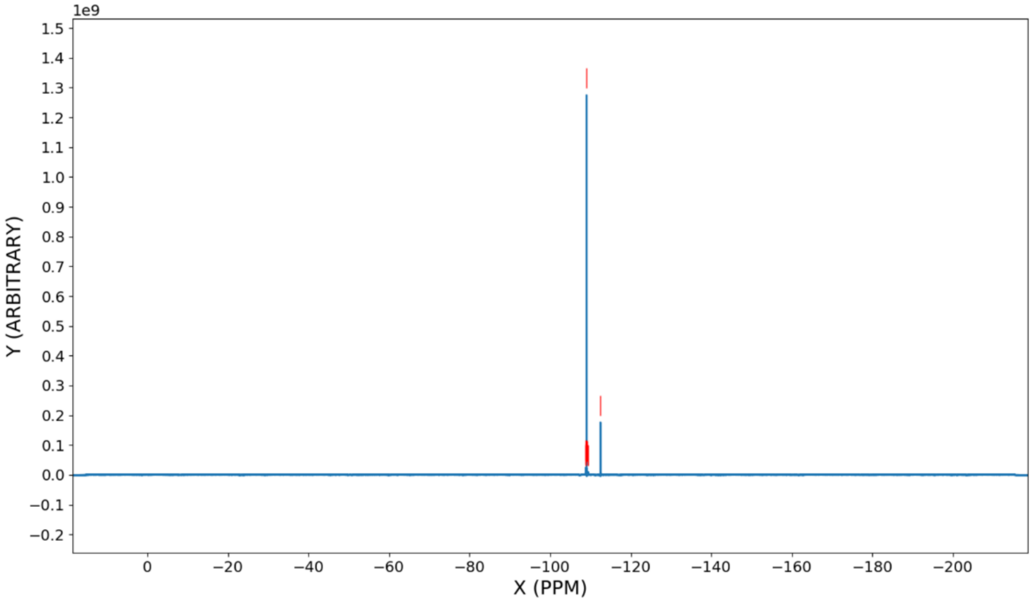

CHMO:0000563 | fast-atom bombardment mass spectrometry (FABMS)

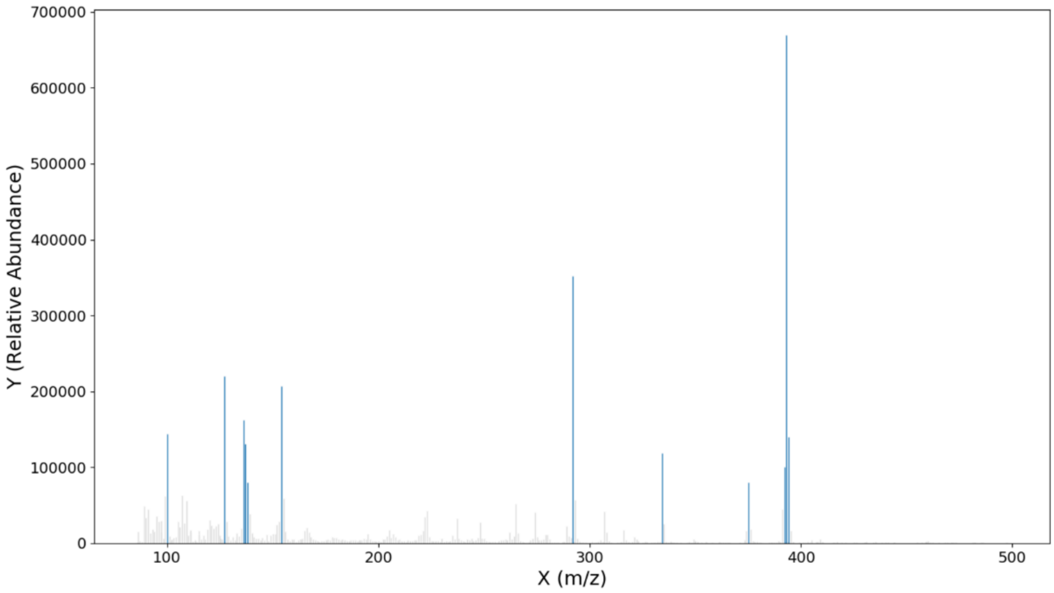

CHMO:0000630 | infrared absorption spectroscopy (IR)

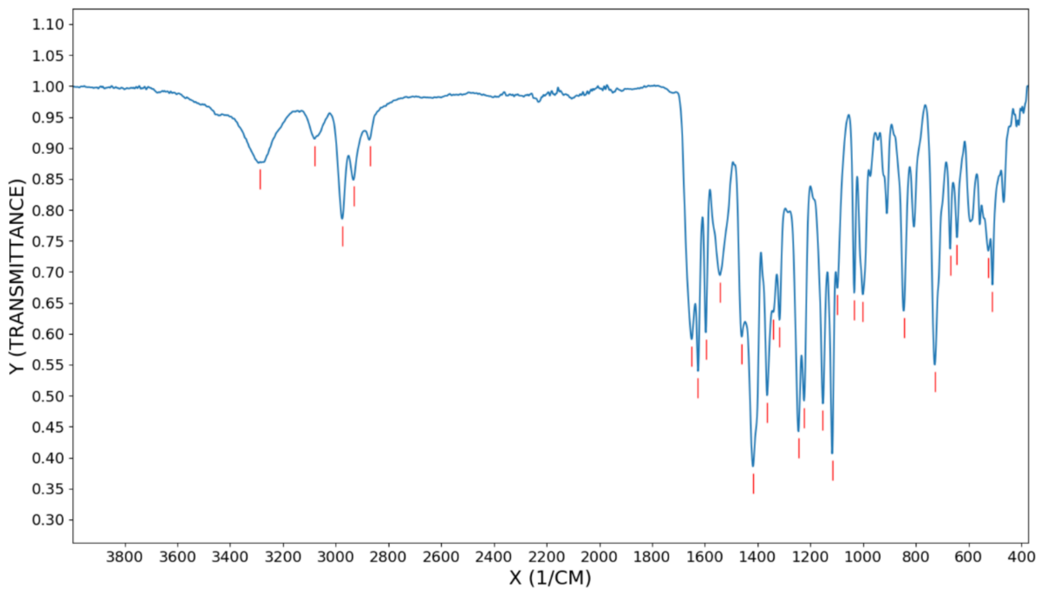

(*E*)-*N*-((3-(3,3-Diisopropyltriaz-1-en-1-yl)-1-ethyl-1*H*-pyrazol-4-yl)methyl)acetamido (9e)

CHMO:0000593 | 1H nuclear magnetic resonance spectroscopy (1H NMR)

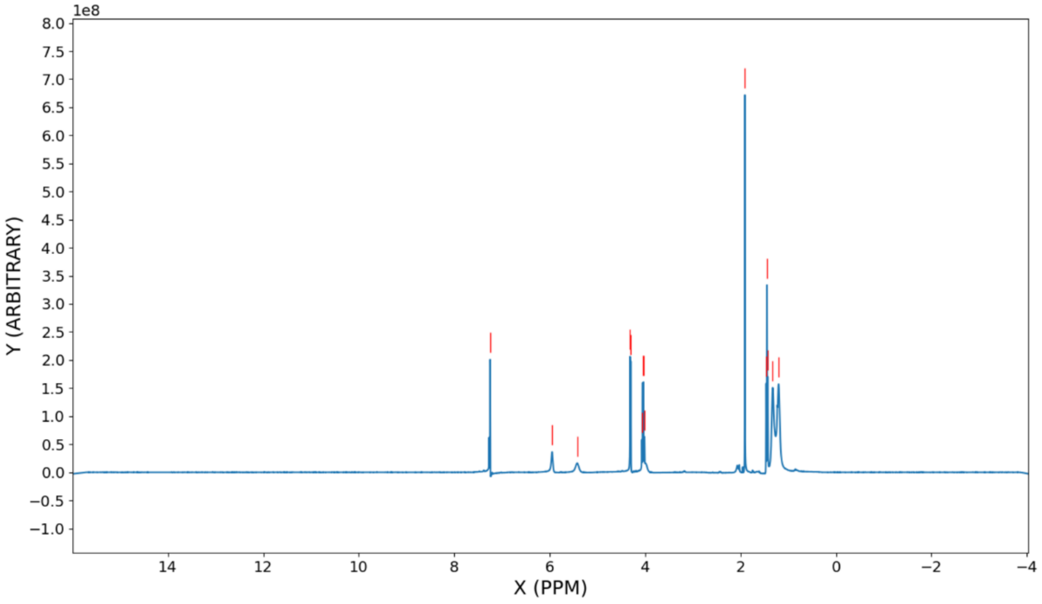

CHMO:0000595 | 13C nuclear magnetic resonance spectroscopy (13C NMR)

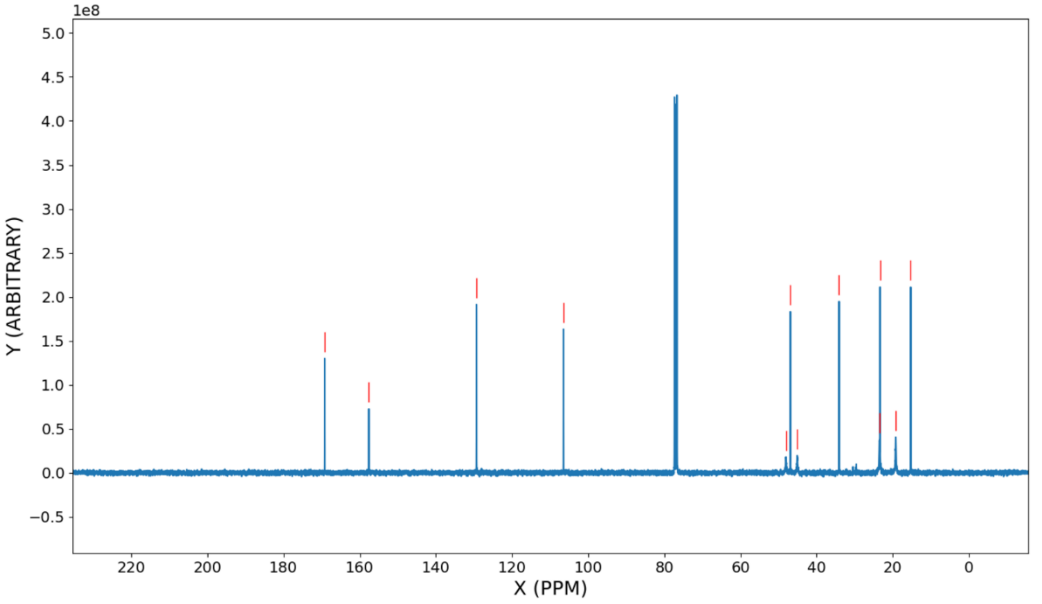

CHMO:0000563 | fast-atom bombardment mass spectrometry (FABMS)

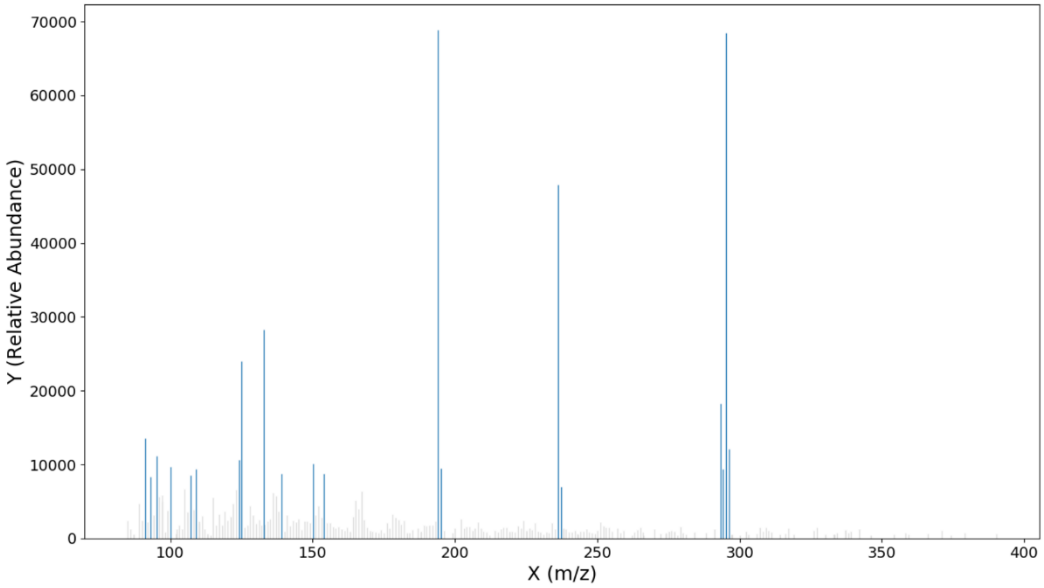

CHMO:0000630 | infrared absorption spectroscopy (IR)

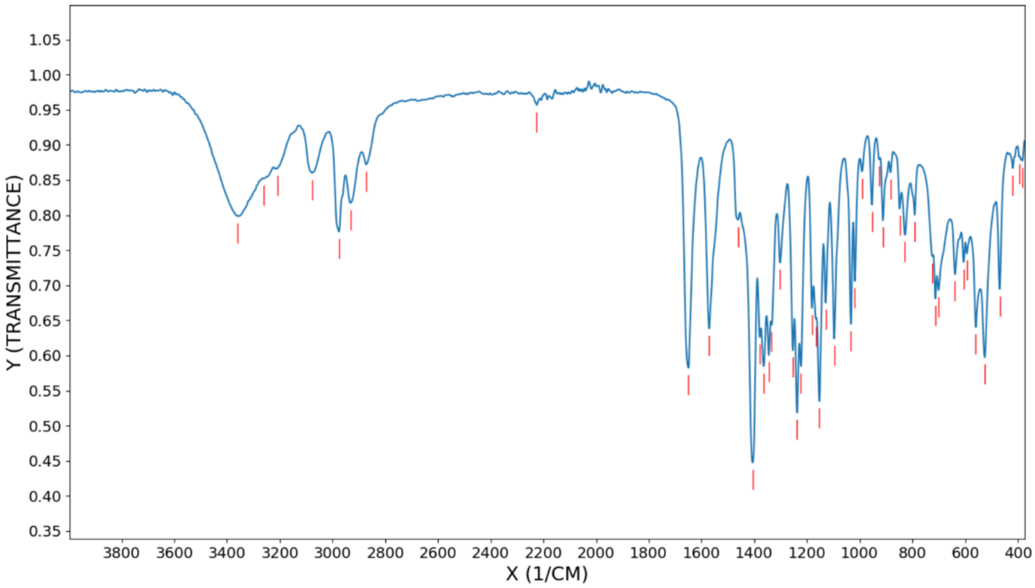

(*E*)-*N*-((1-Cyclopentyl-3-(3,3-diisopropyltriaz-1-en-1-yl)-1*H*-pyrazol-4-yl)methyl)acetamido (9f)

CHMO:0000593 | 1H nuclear magnetic resonance spectroscopy (1H NMR)

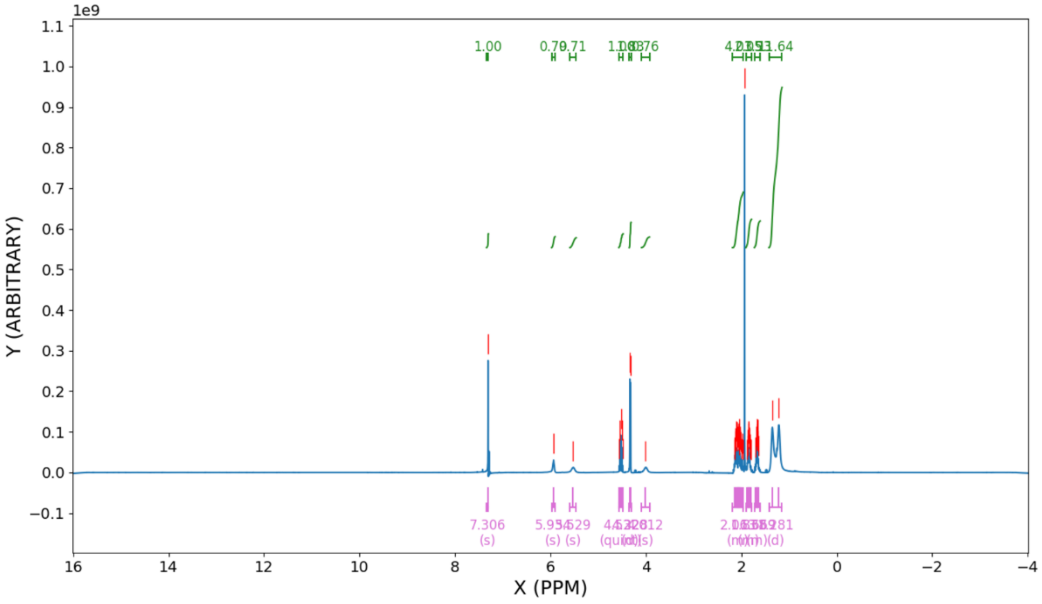

CHMO:0000595 | 13C nuclear magnetic resonance spectroscopy (13C NMR)

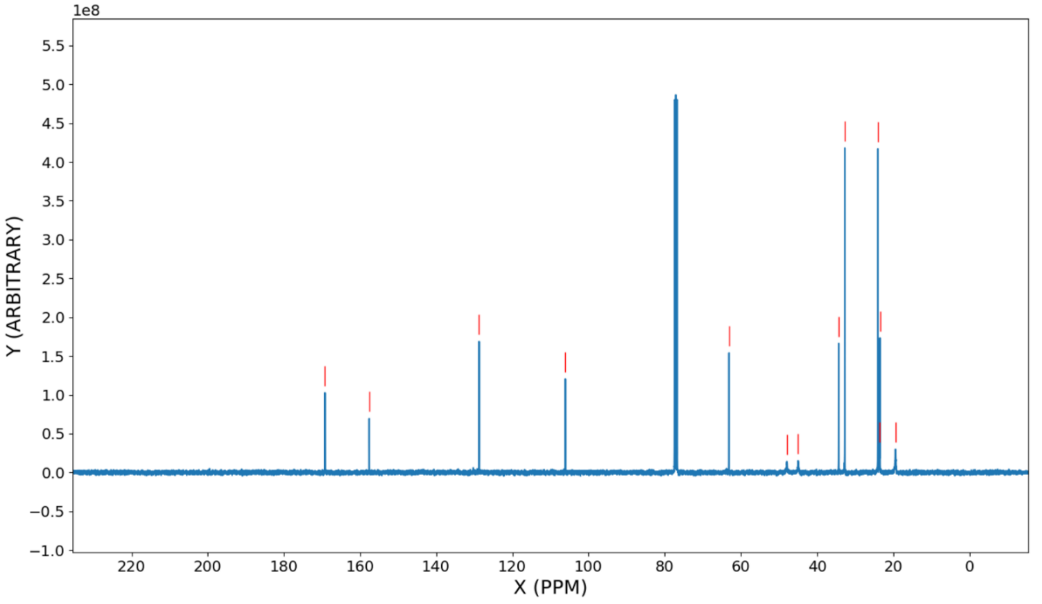

CHMO:0000563 | fast-atom bombardment mass spectrometry (FABMS)

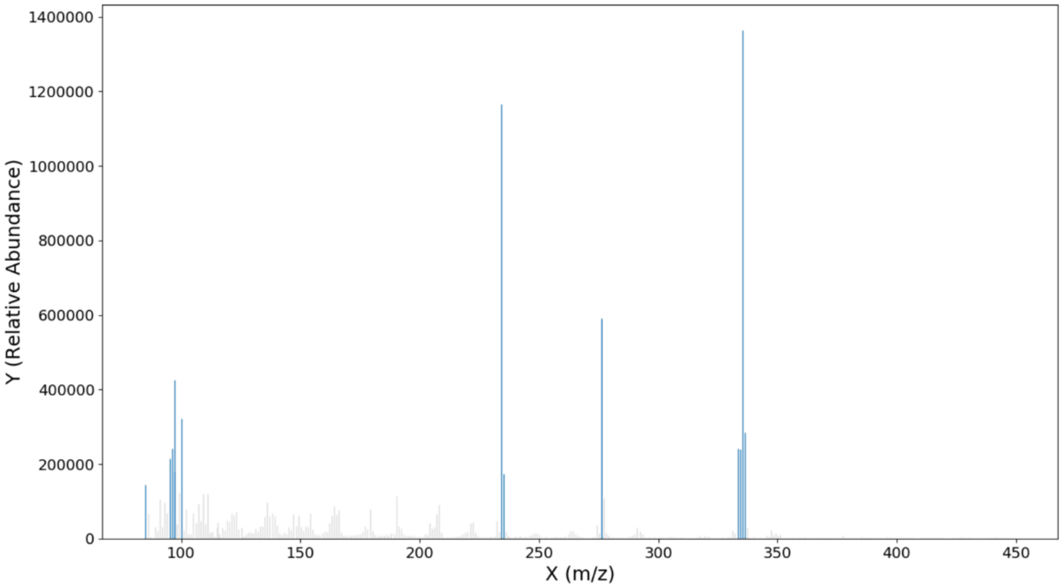

CHMO:0000630 | infrared absorption spectroscopy (IR)

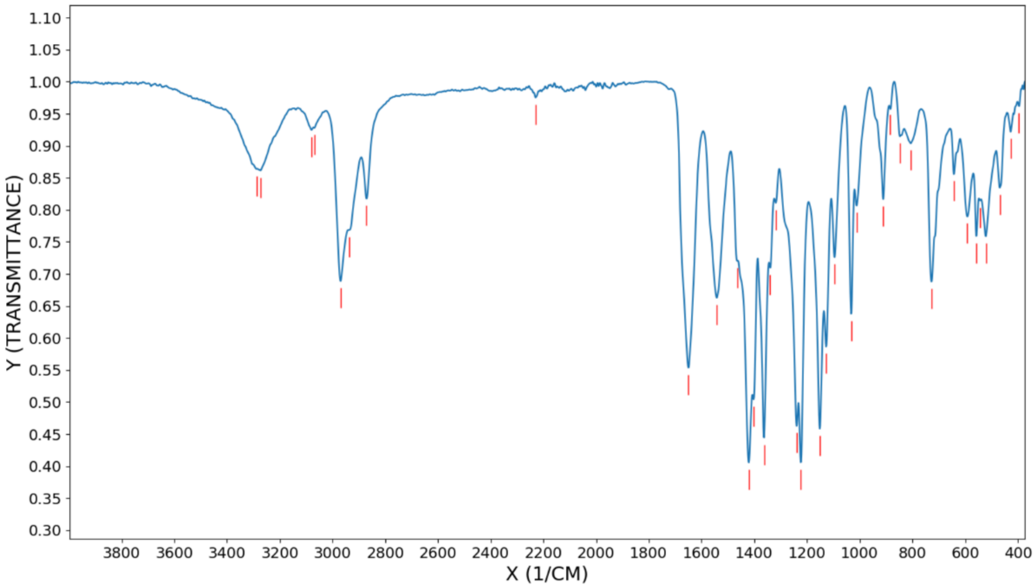

(*E*)-*N*-((1-Cyclopentyl-3-(3,3-diisopropyltriaz-1-en-1-yl)-1*H*-pyrazol-4-yl)methyl)benzamide (9g)

CHMO:0000593 | 1H nuclear magnetic resonance spectroscopy (1H NMR)

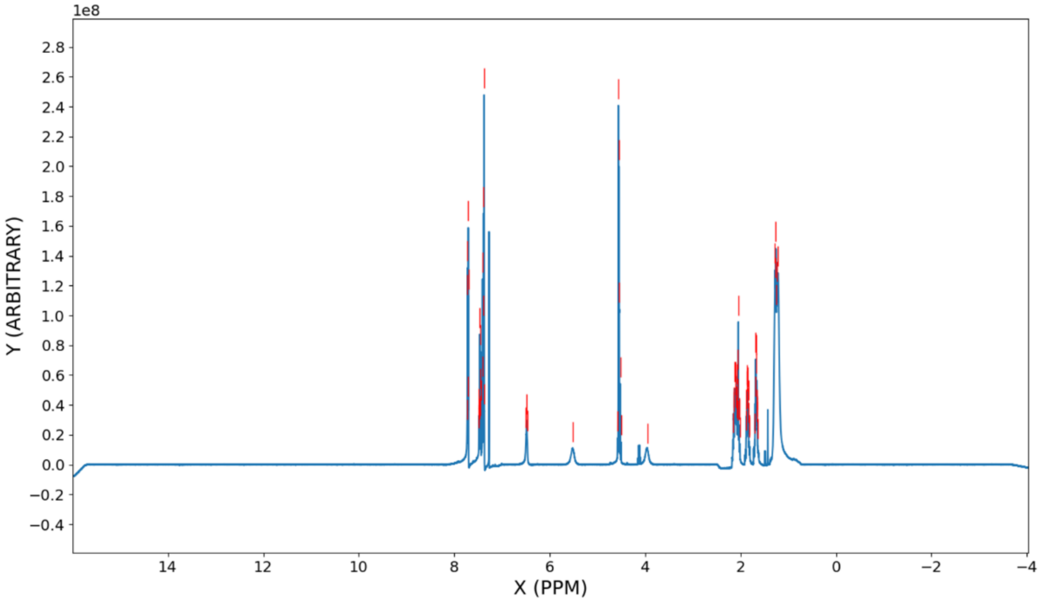

CHMO:0000595 | 13C nuclear magnetic resonance spectroscopy (13C NMR)

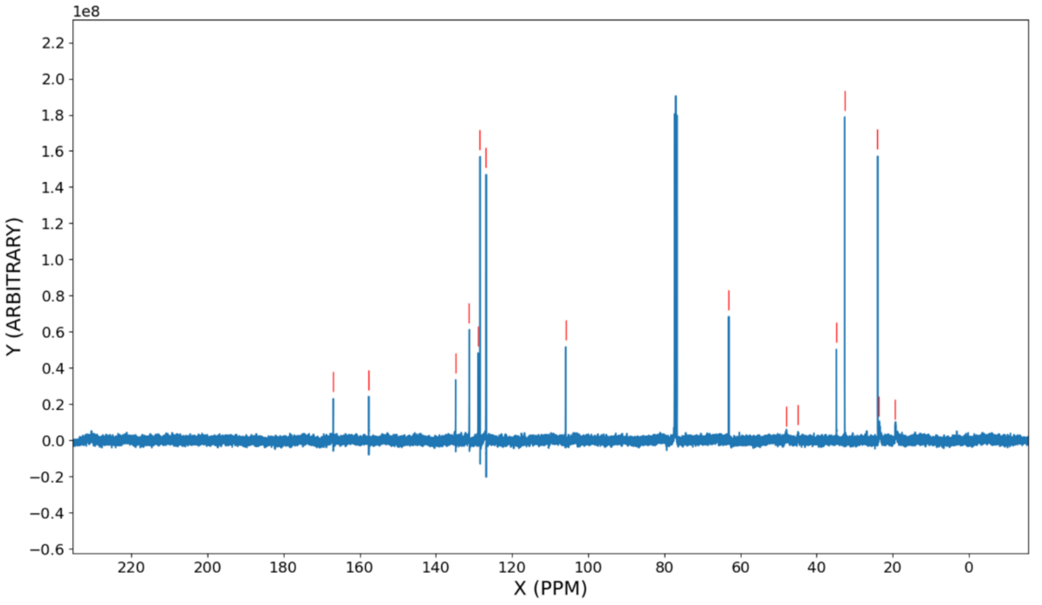

CHMO:0000563 | fast-atom bombardment mass spectrometry (FABMS)

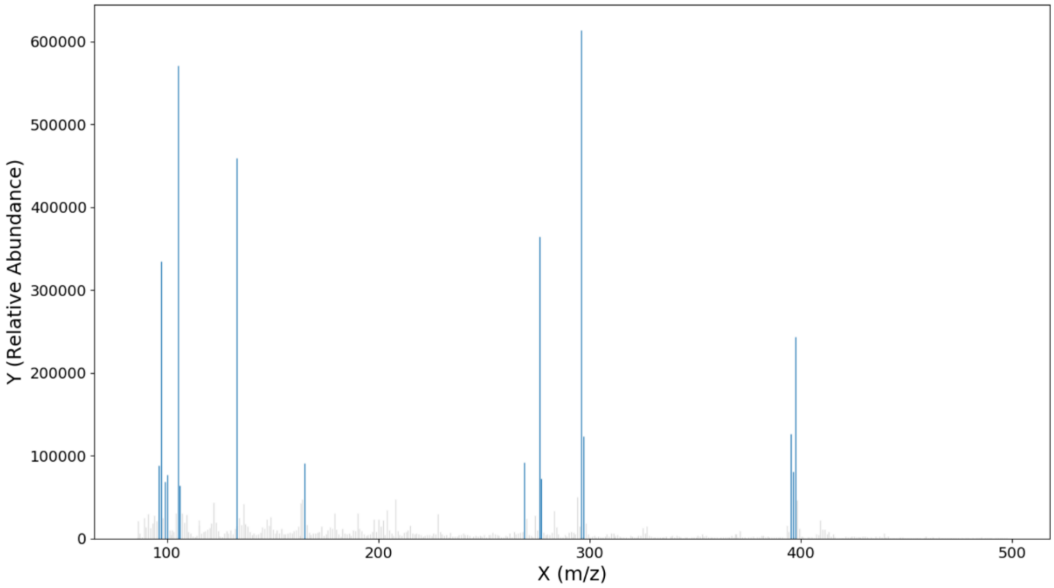

CHMO:0000630 | infrared absorption spectroscopy (IR)

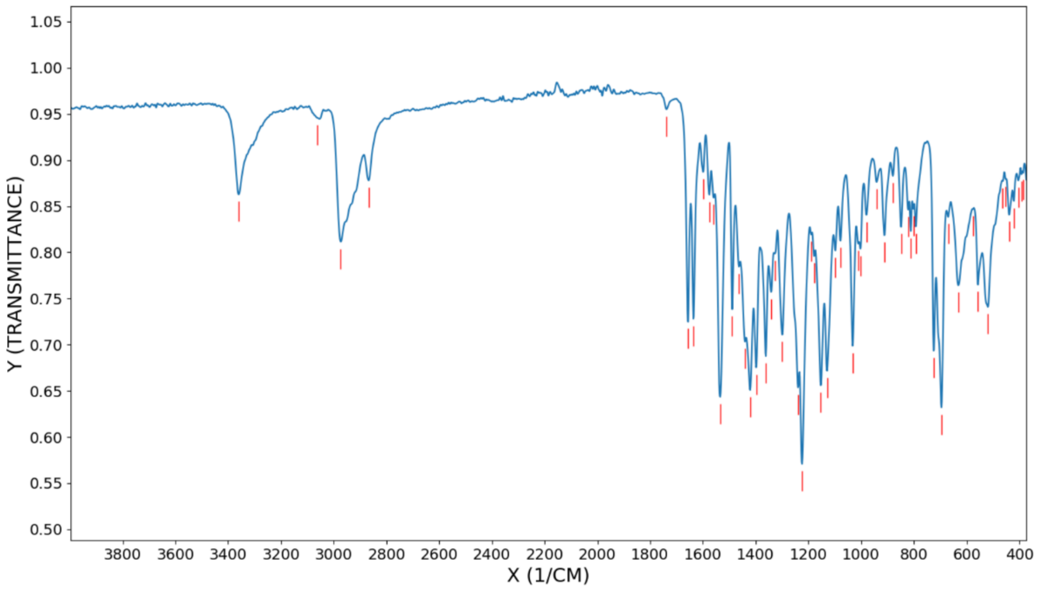

(*E*)-*N*-((3-(3,3-Diisopropyltriaz-1-en-1-yl)-1-isobutyl-1*H*-pyrazol-4-yl)methyl)acetamido (9h)

CHMO:0000593 | 1H nuclear magnetic resonance spectroscopy (1H NMR)

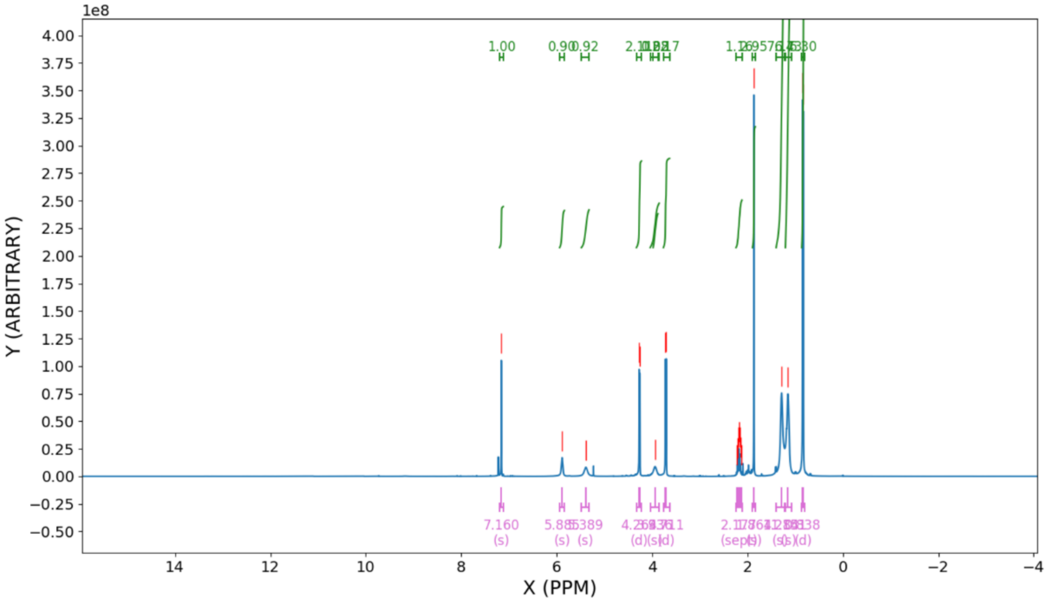

CHMO:0000595 | 13C nuclear magnetic resonance spectroscopy (13C NMR)

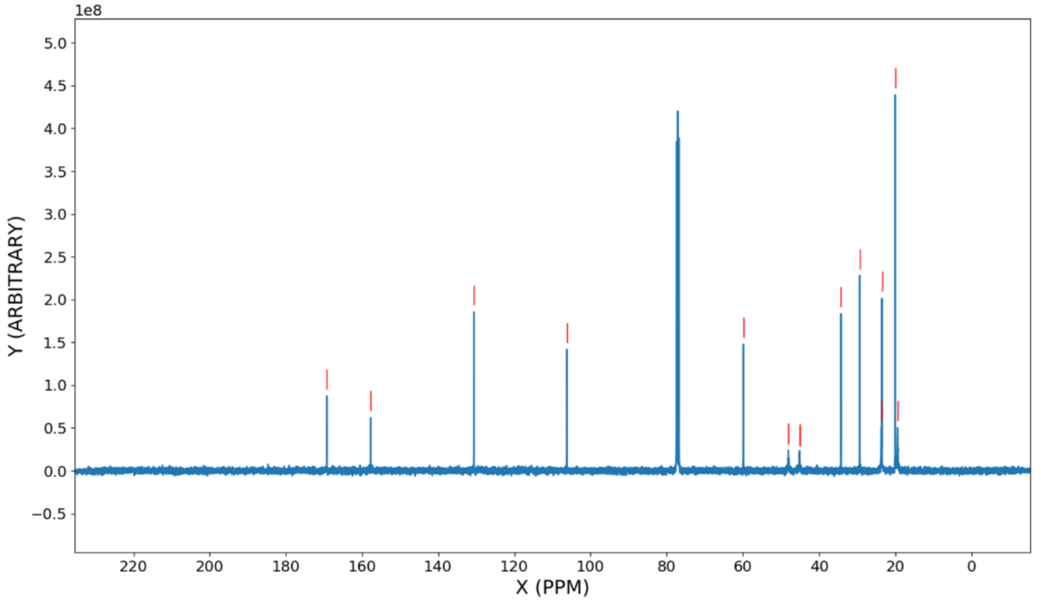

CHMO:0000563 | fast-atom bombardment mass spectrometry (FABMS)

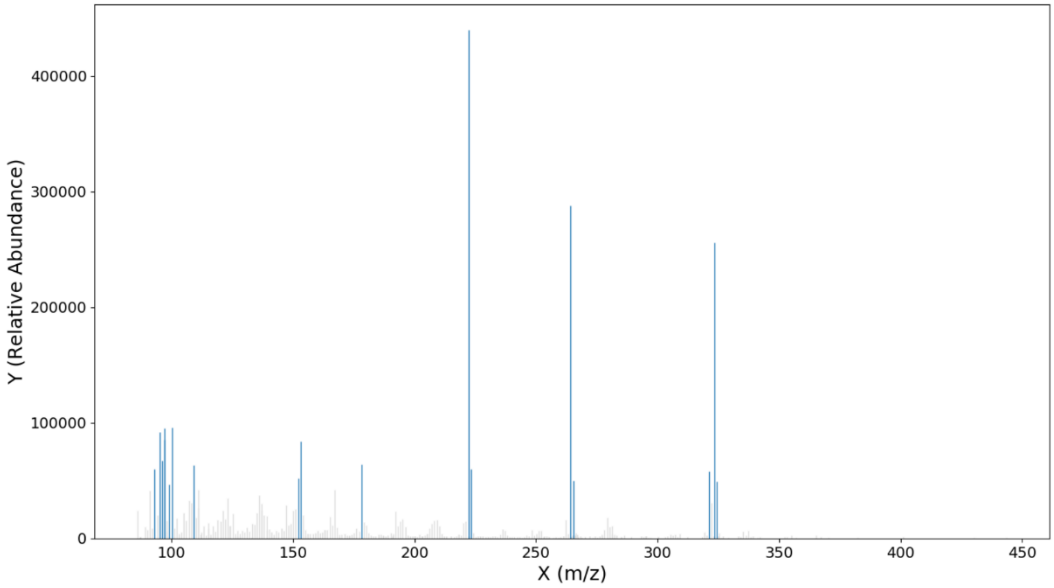

CHMO:0000630 | infrared absorption spectroscopy (IR)

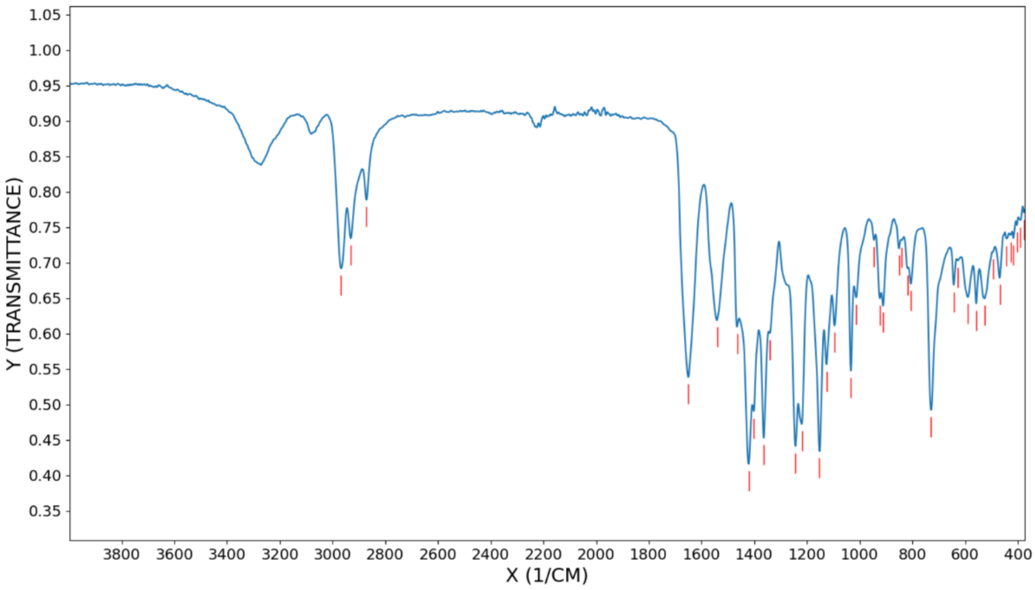

(*E*)-2-(4-(Acetamidomethyl)-3-(3,3-diisopropyltriaz-1-en-1-yl)-1*H*-pyrazol-1-yl)ethyl acetate (9i)

CHMO:0000593 | 1H nuclear magnetic resonance spectroscopy (1H NMR)

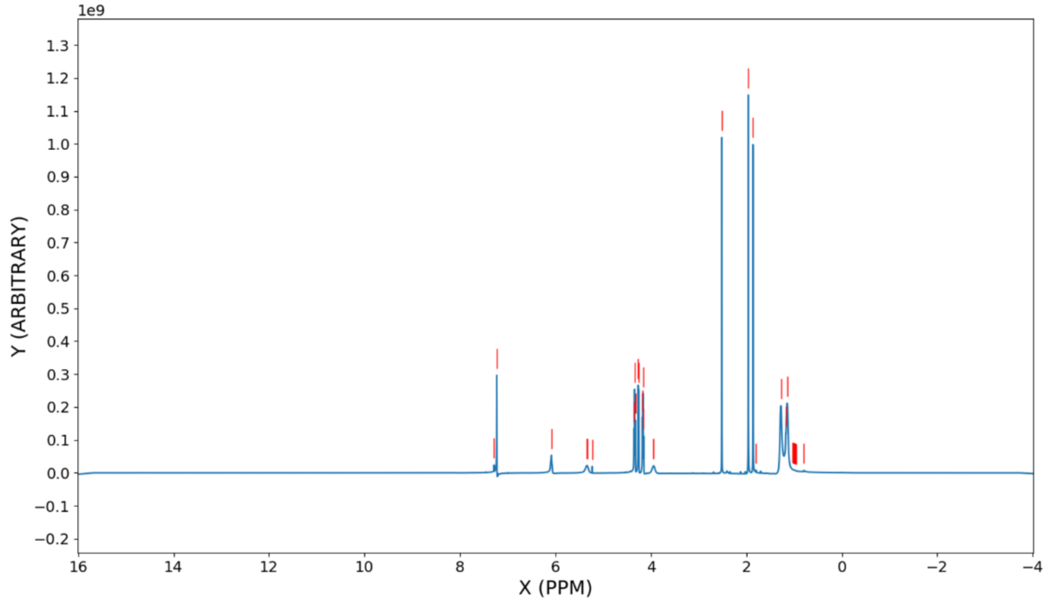

CHMO:0000595 | 13C nuclear magnetic resonance spectroscopy (13C NMR)

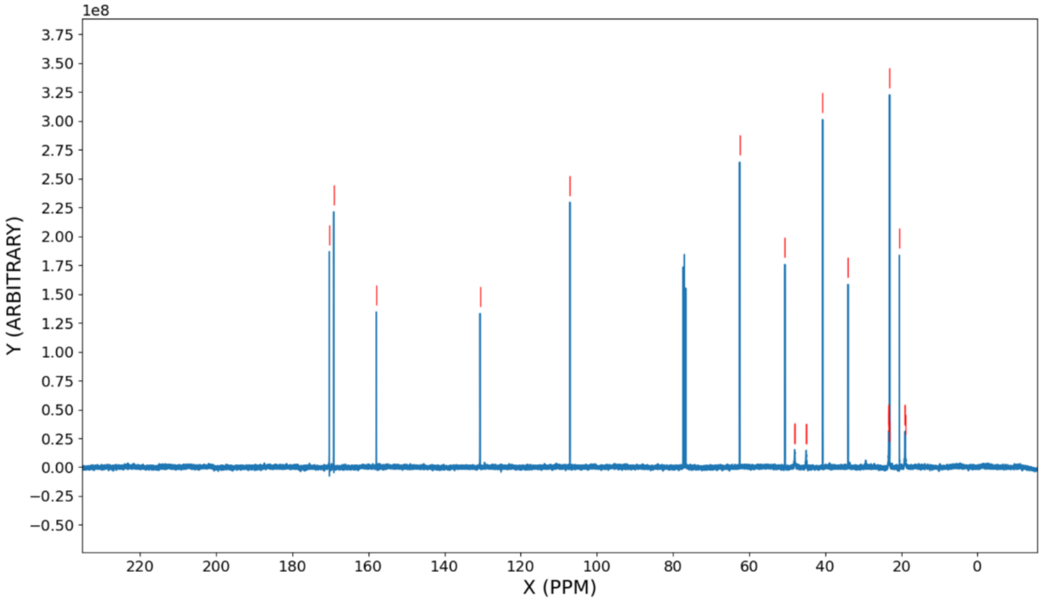

CHMO:0000563 | fast-atom bombardment mass spectrometry (FABMS)

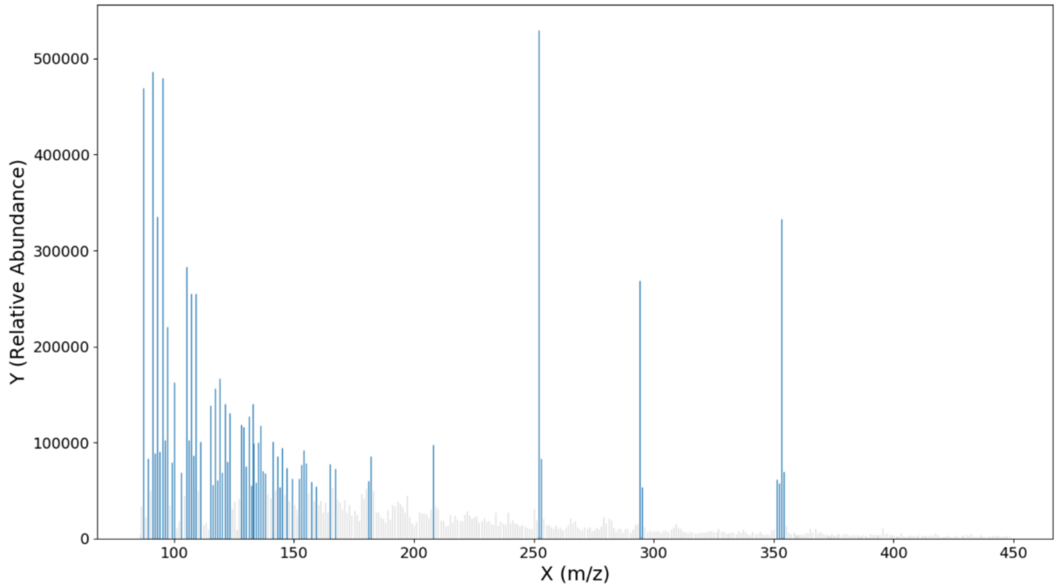

CHMO:0000630 | infrared absorption spectroscopy (IR)

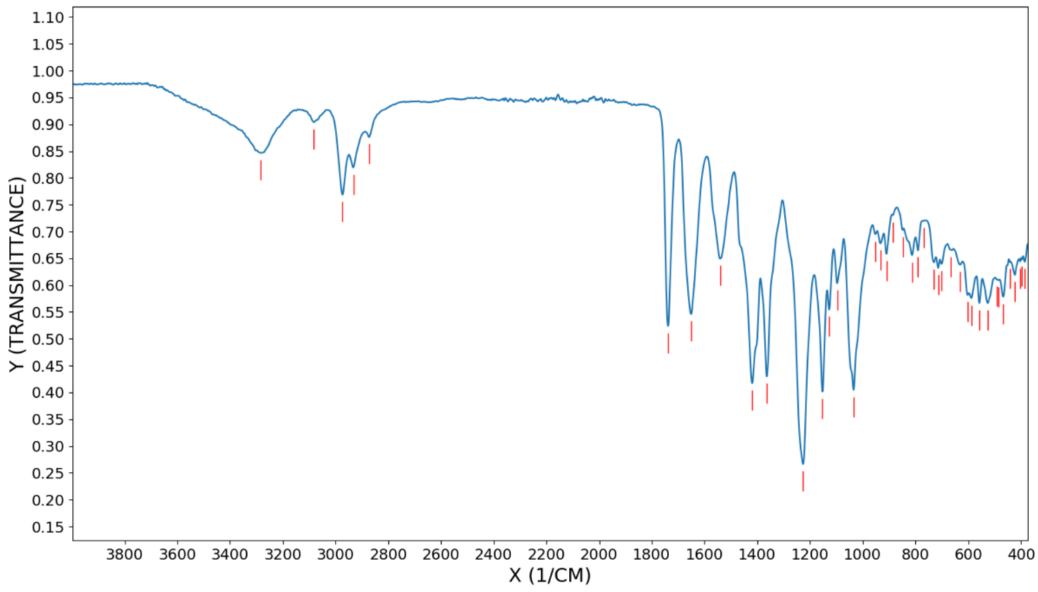

1-(6-Benzyl-4,6-dihydro-3*H*-pyrazolo[3,4-*d*][1,2,3]triazin-3-yl)ethan-1-one (5a)

CHMO:0000593 | 1H nuclear magnetic resonance spectroscopy (1H NMR)

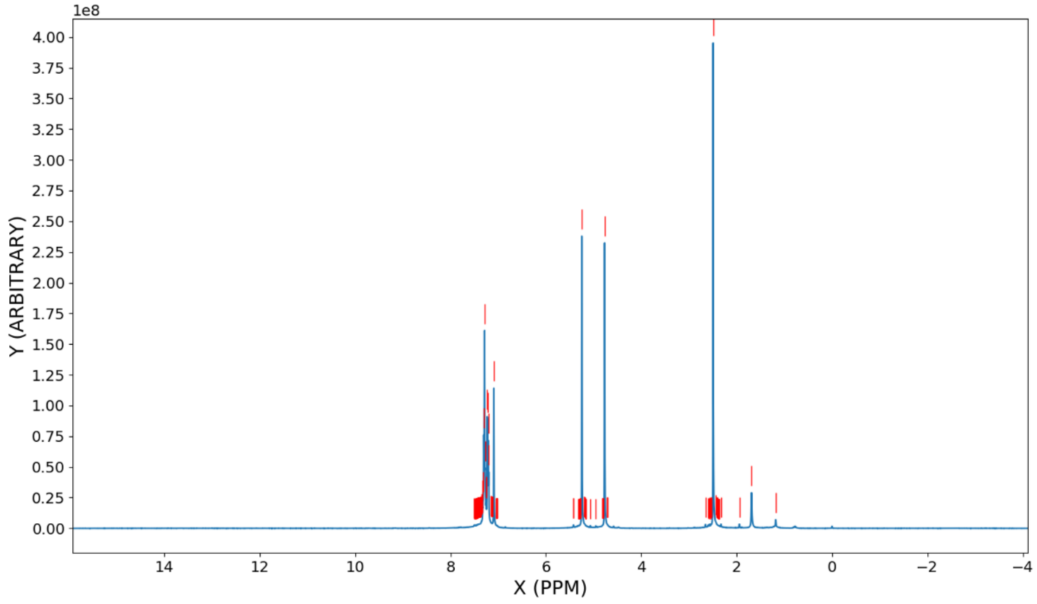

CHMO:0000595 | 13C nuclear magnetic resonance spectroscopy (13C NMR)

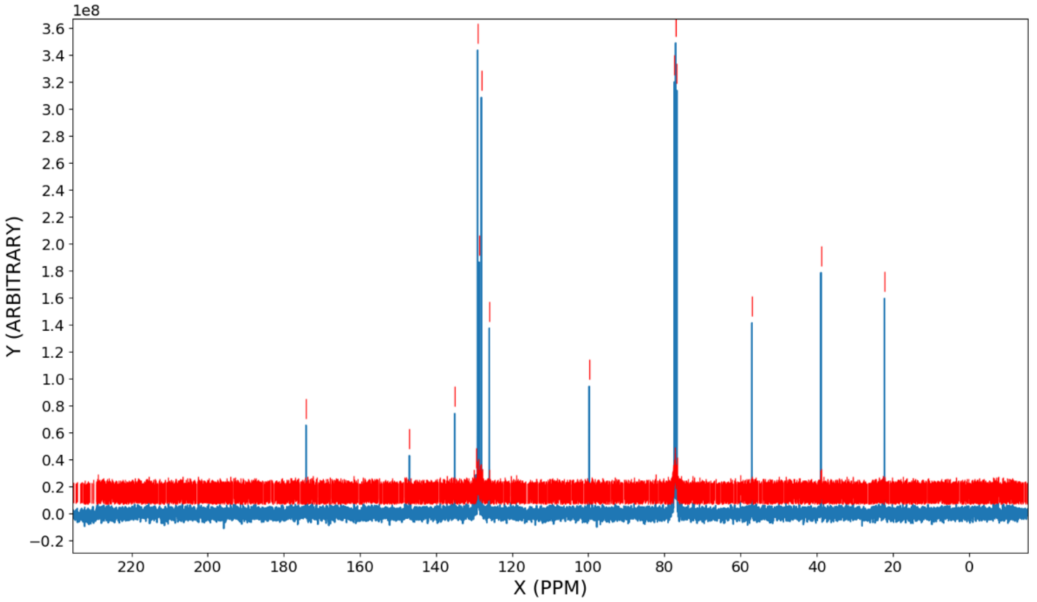

CHMO:0000630 | infrared absorption spectroscopy (IR)

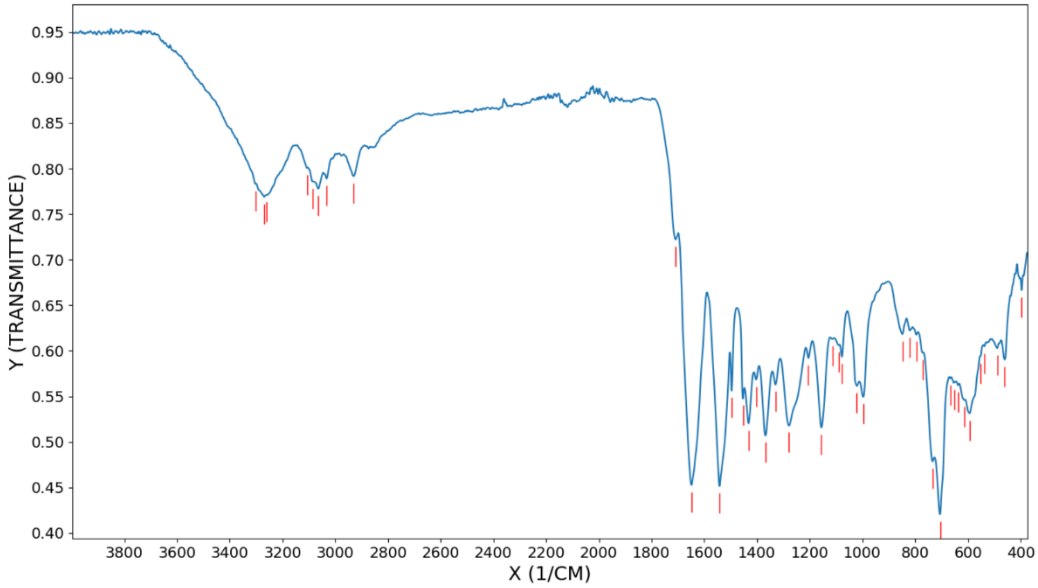

HMO:0000563 | fast-atom bombardment mass spectrometry (FABMS)

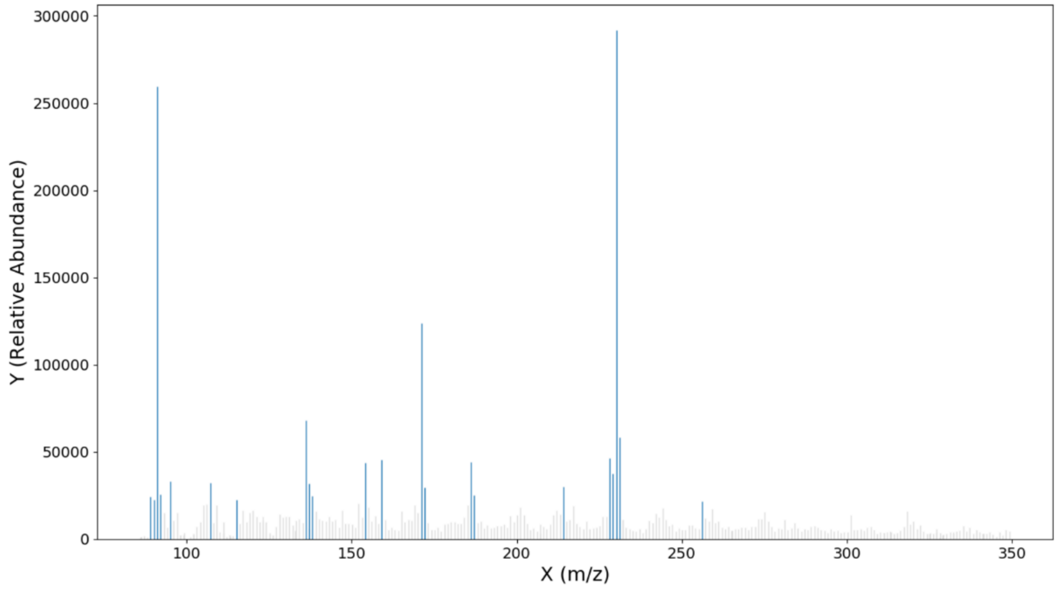

(6-Benzyl-4,6-dihydro-3*H*-pyrazolo[3,4-*d*][1,2,3]triazin-3-yl)(phenyl)methanone (5b)

CHMO:0000593 | 1H nuclear magnetic resonance spectroscopy (1H NMR)

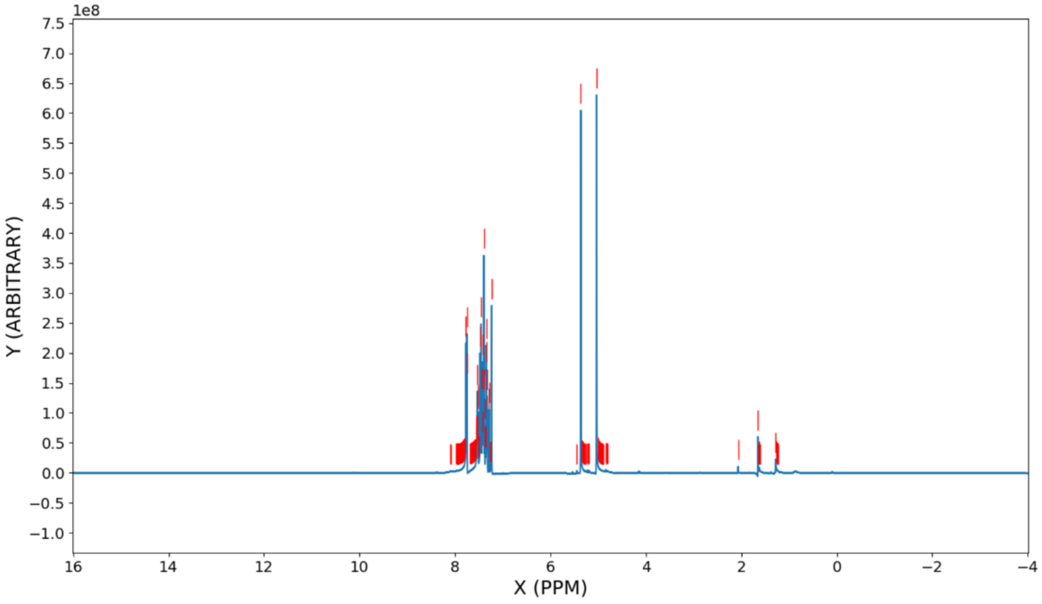

CHMO:0000595 | 13C nuclear magnetic resonance spectroscopy (13C NMR)

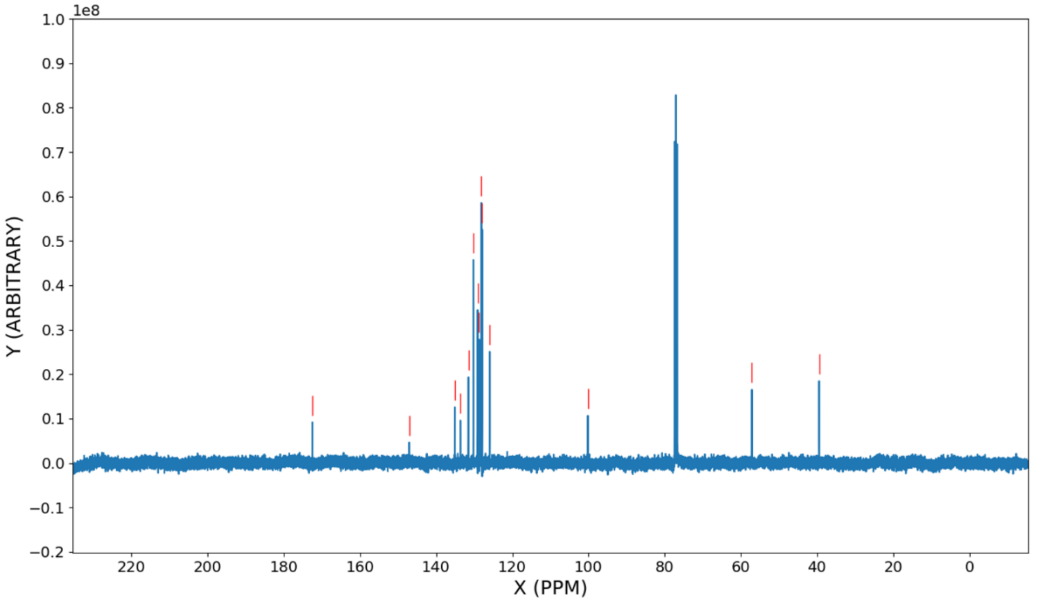

CHMO:0000563 | fast-atom bombardment mass spectrometry (FABMS)

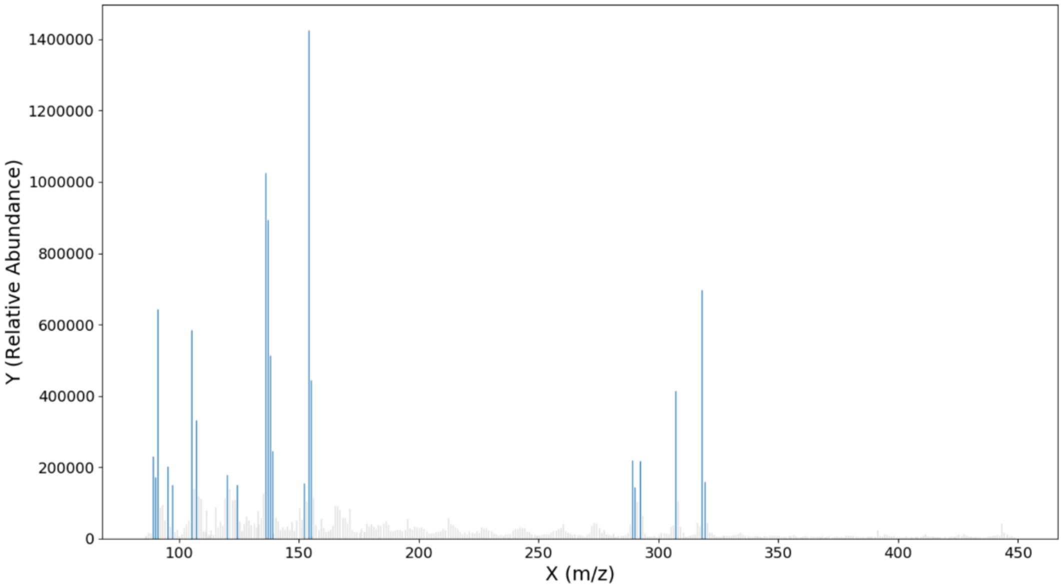

CHMO:0000630 | infrared absorption spectroscopy (IR)

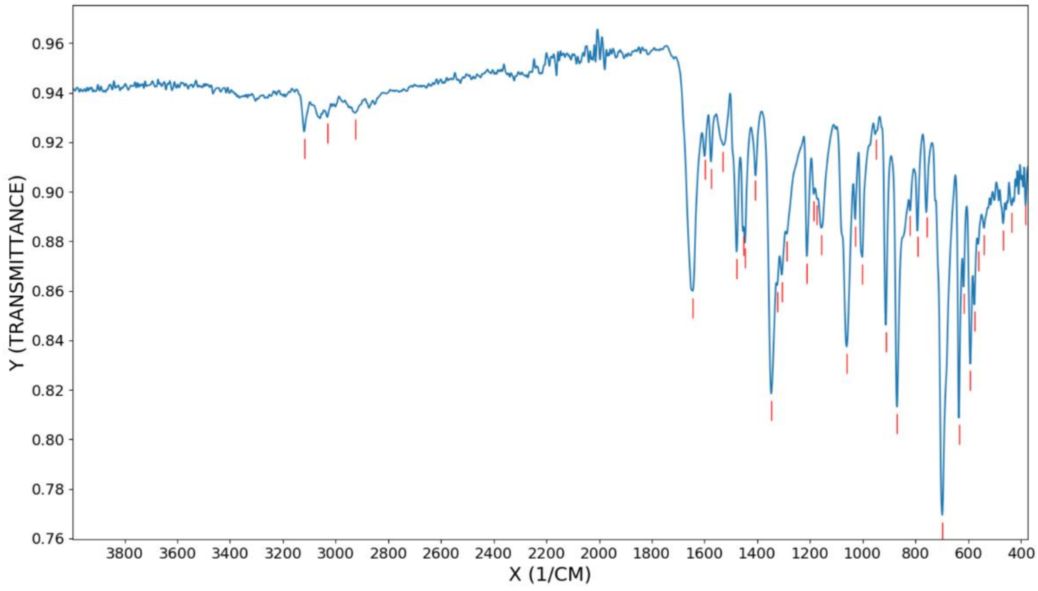

3-Methyl-1-(6-(4-methylbenzyl)-4,6-dihydro-3*H*-pyrazolo[3,4-*d*][1,2,3]triazin-3-yl)butan-1-one (5c)

CHMO:0000593 | 1H nuclear magnetic resonance spectroscopy (1H NMR)

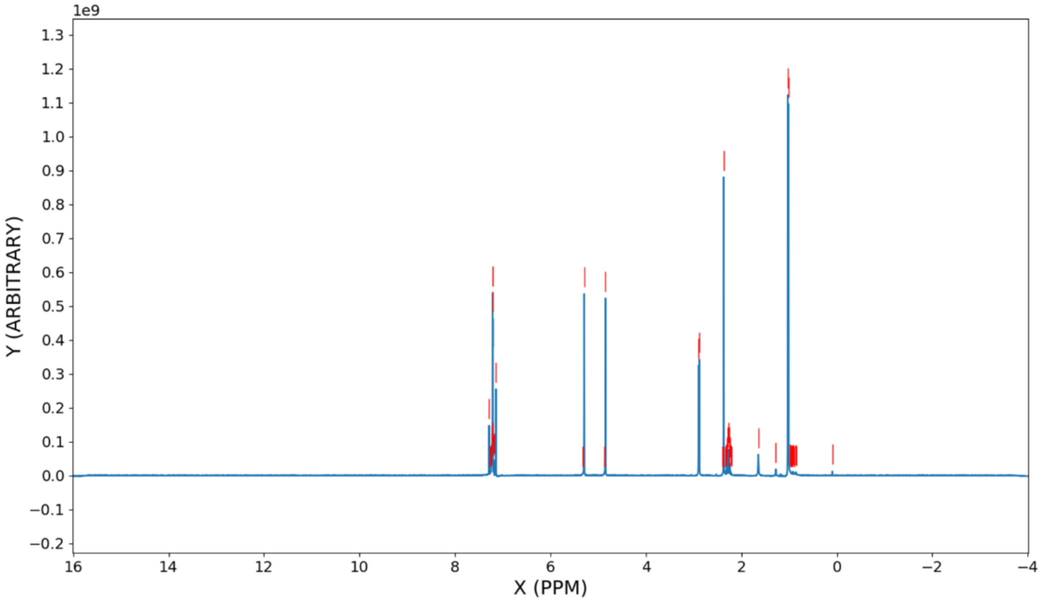

CHMO:0000595 | 13C nuclear magnetic resonance spectroscopy (13C NMR)

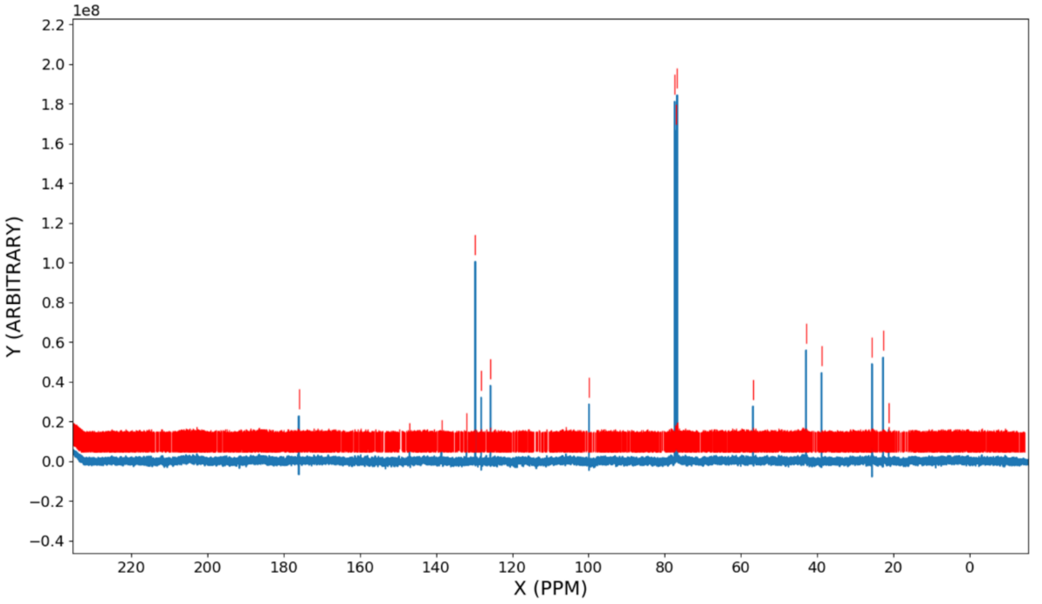

CHMO:0000563 | fast-atom bombardment mass spectrometry (FABMS)

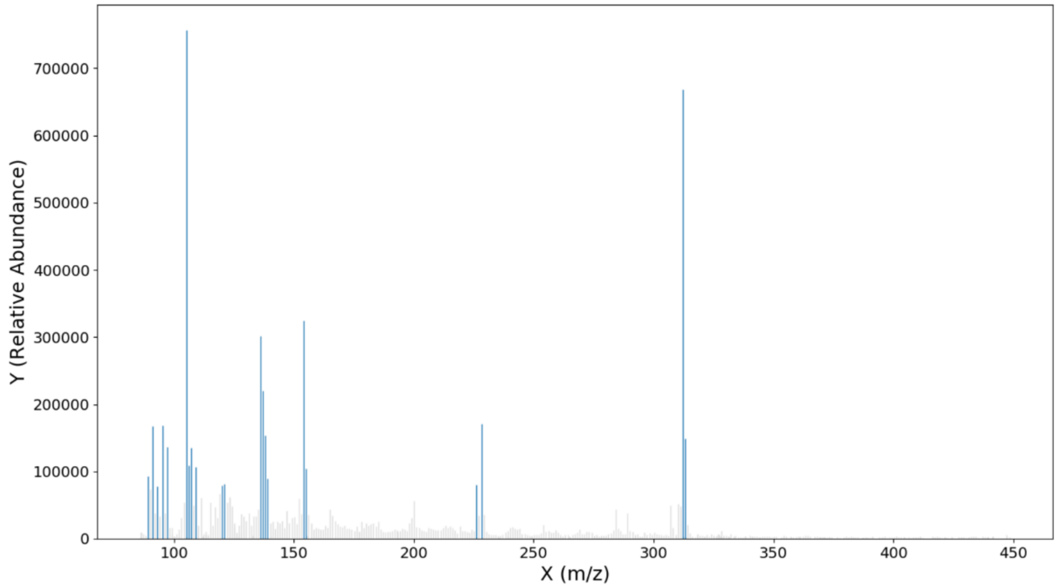

CHMO:0000630 | infrared absorption spectroscopy (IR)

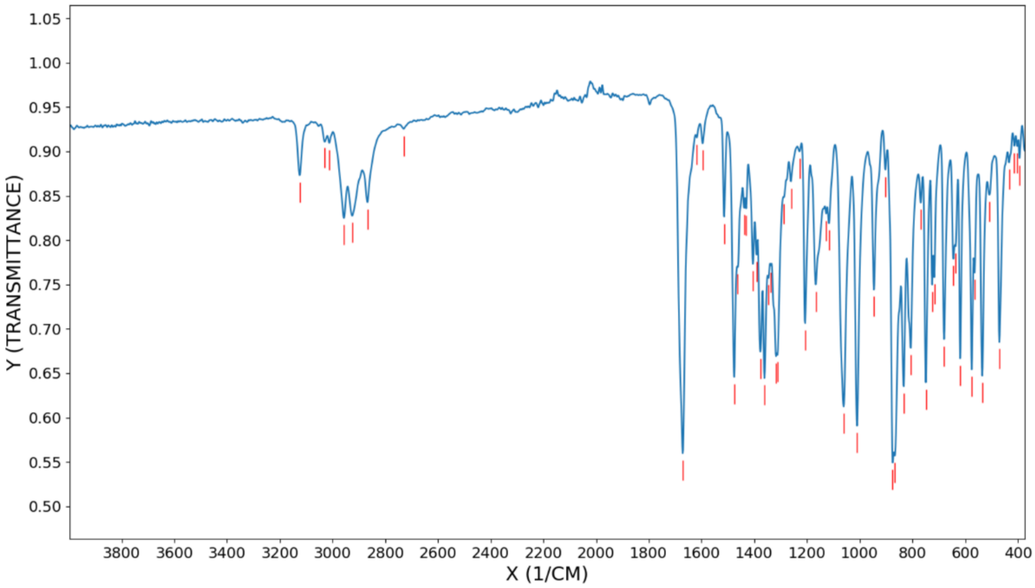

1-(6-(3,5-Difluorobenzyl)-4,6-dihydro-3*H*-pyrazolo[3,4-*d*][1,2,3]triazin-3-yl)ethan-1-one (5d)

CHMO:0000593 | 1H nuclear magnetic resonance spectroscopy (1H NMR)

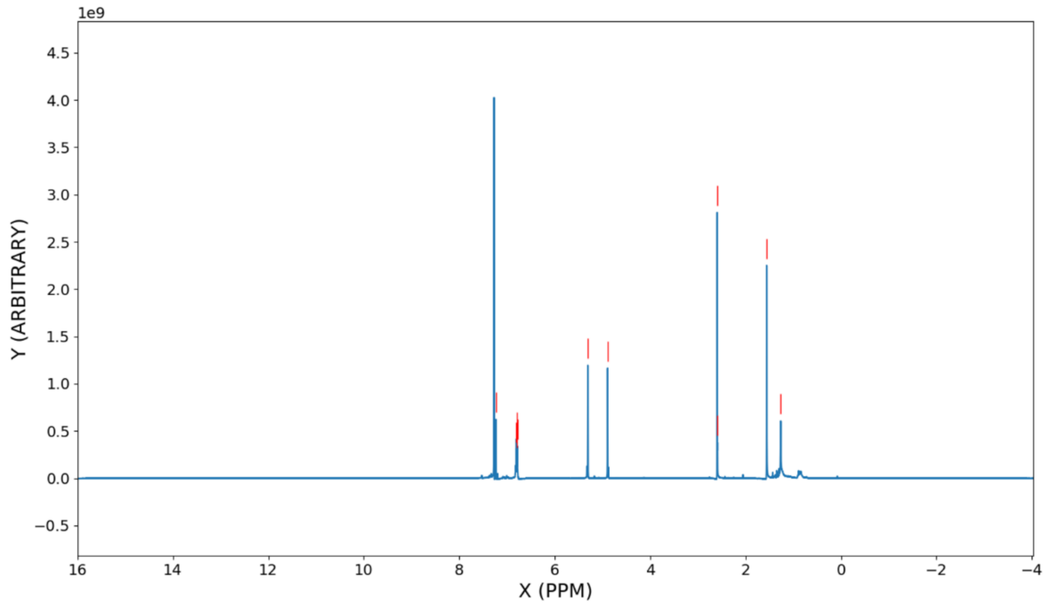

CHMO:0000597 | 19F nuclear magnetic resonance spectroscopy (19F NMR)

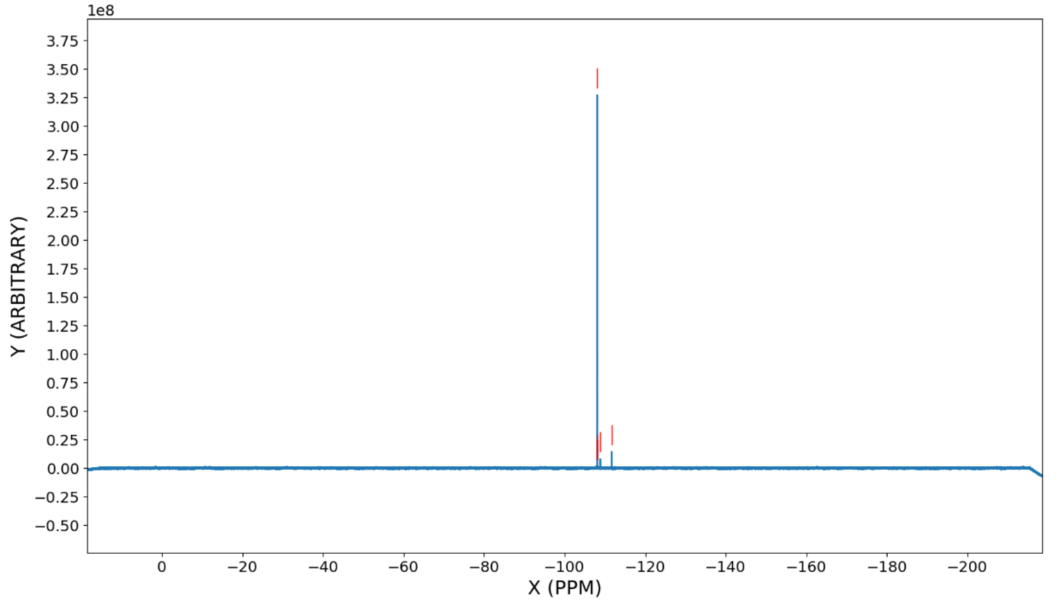

CHMO:0000630 | infrared absorption spectroscopy (IR)

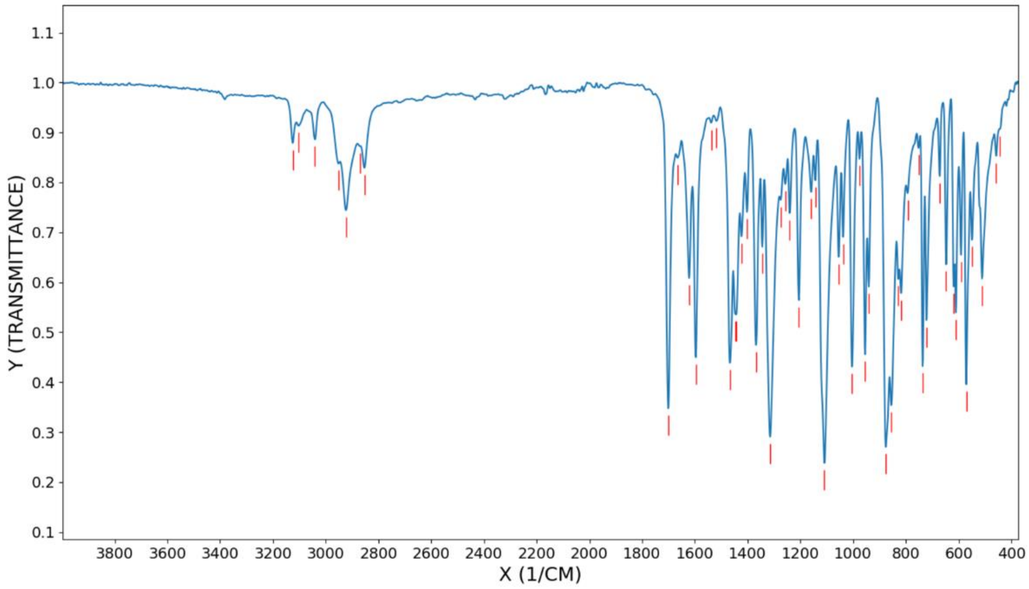

CHMO:0000563 | fast-atom bombardment mass spectrometry (FABMS)

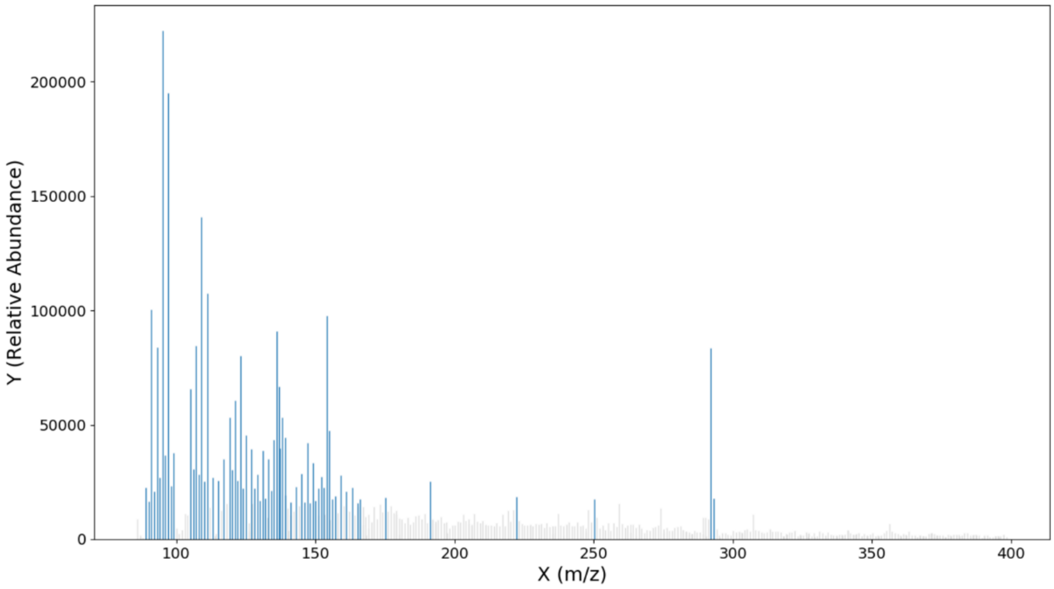

1-(6-Ethyl-4,6-dihydro-3*H*-pyrazolo[3,4-*d*][1,2,3]triazin-3-yl)ethan-1-one (5e)

CHMO:0000593 | 1H nuclear magnetic resonance spectroscopy (1H NMR)

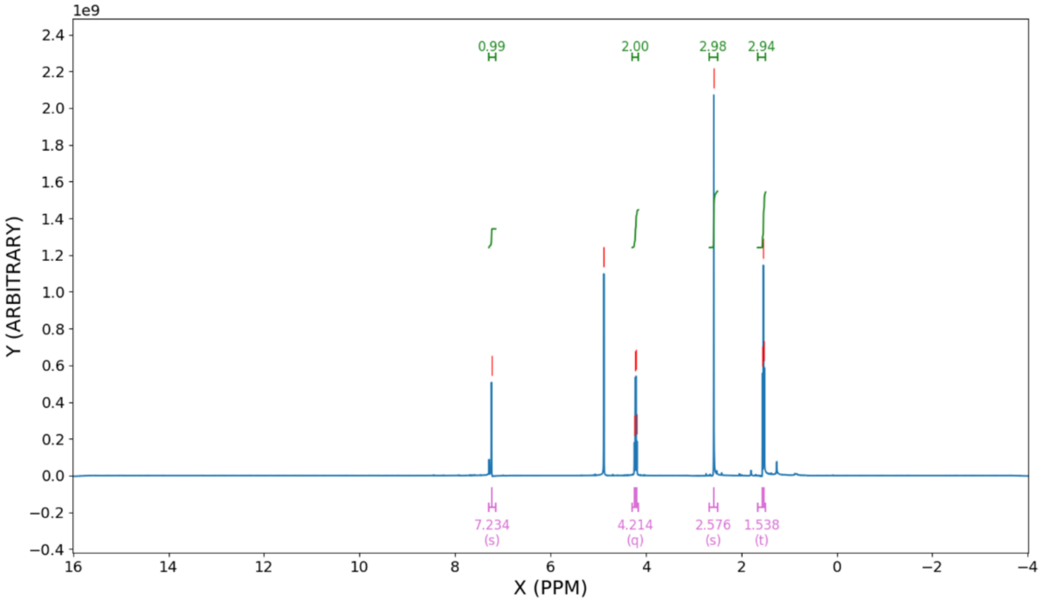

CHMO:0000595 | 13C nuclear magnetic resonance spectroscopy (13C NMR)

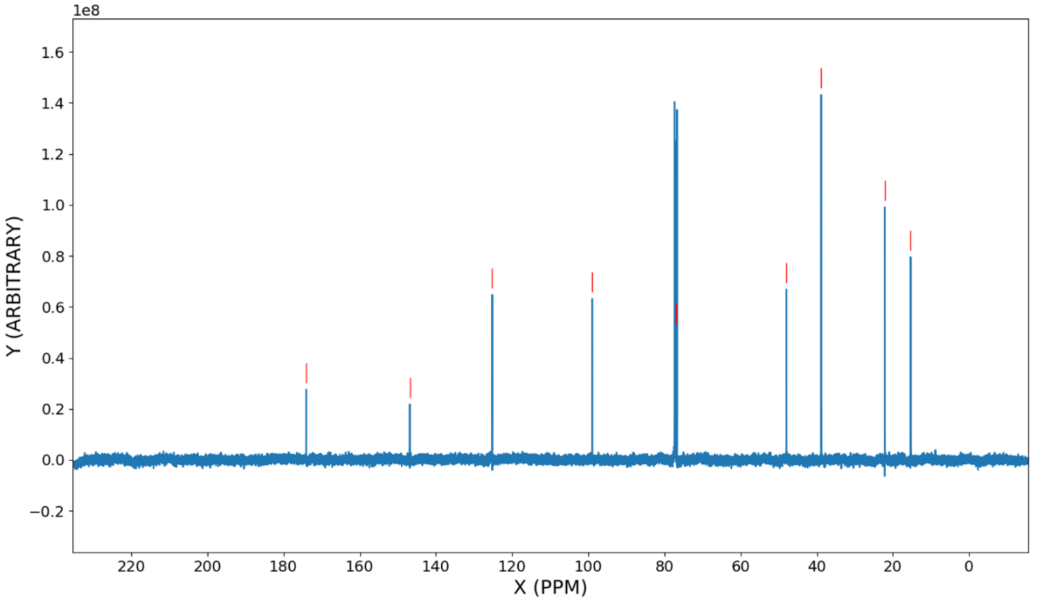

CHMO:0000563 | fast-atom bombardment mass spectrometry (FABMS)

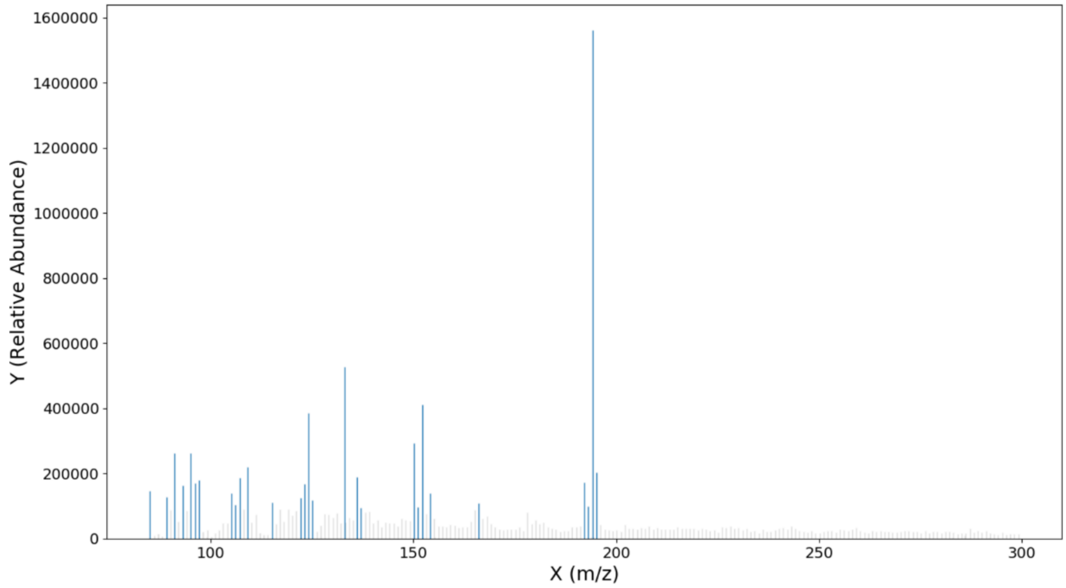

CHMO:0000630 | infrared absorption spectroscopy (IR)

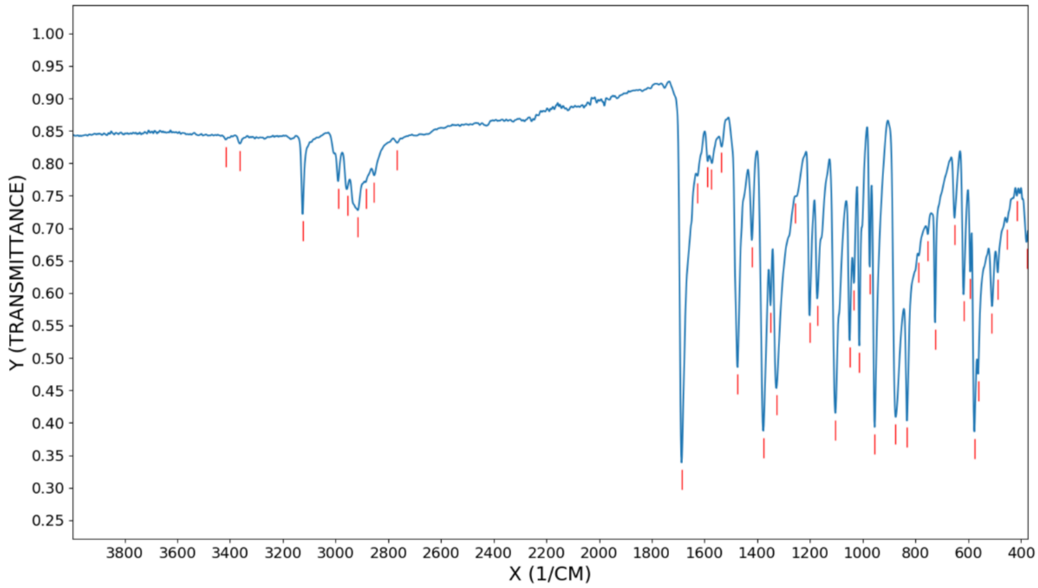

1-(6-Cyclopentyl-4,6-dihydro-3*H*-pyrazolo[3,4-*d*][1,2,3]triazin-3-yl)ethan-1-one (5f)

CHMO:0000593 | 1H nuclear magnetic resonance spectroscopy (1H NMR)

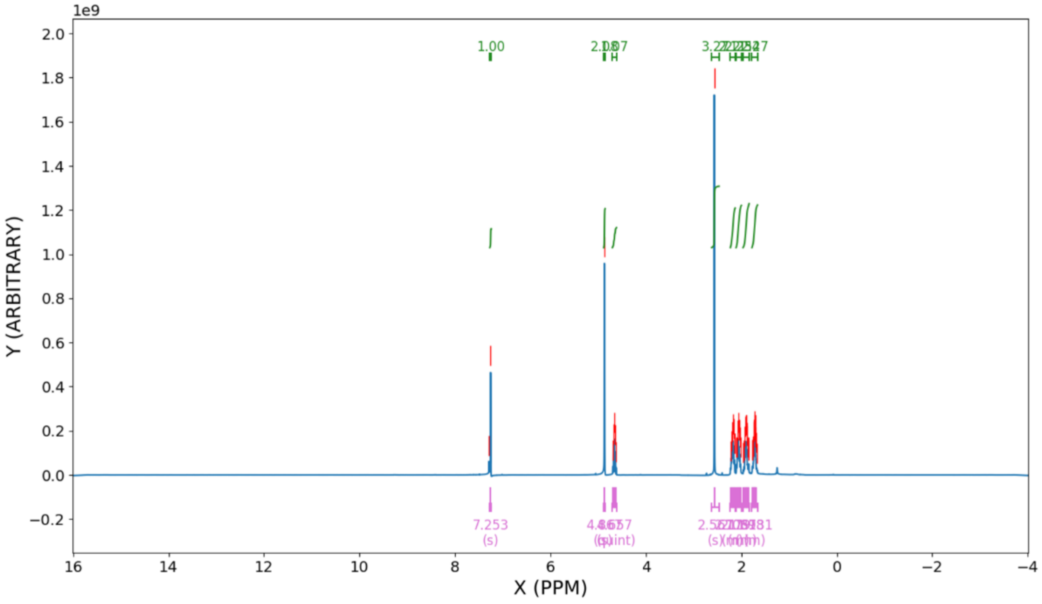

CHMO:0000595 | 13C nuclear magnetic resonance spectroscopy (13C NMR)

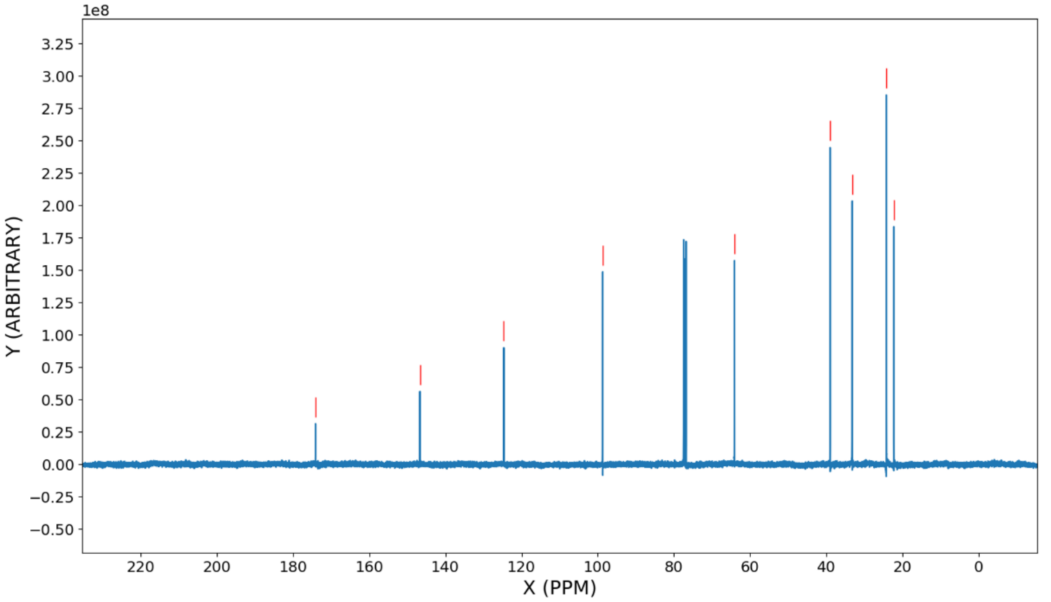

CHMO:0000563 | fast-atom bombardment mass spectrometry (FABMS)

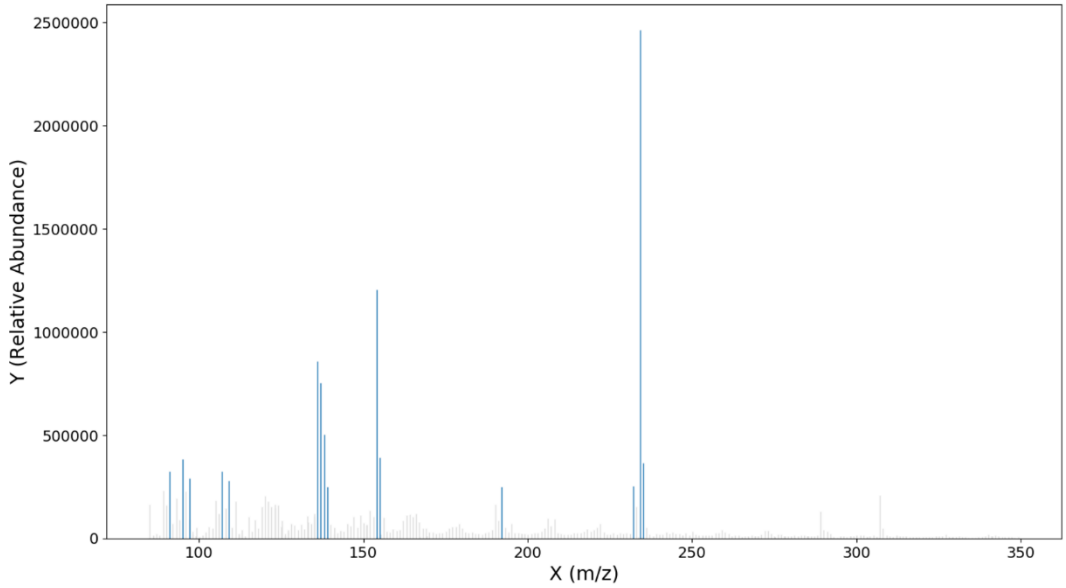

CHMO:0000630 | infrared absorption spectroscopy (IR)

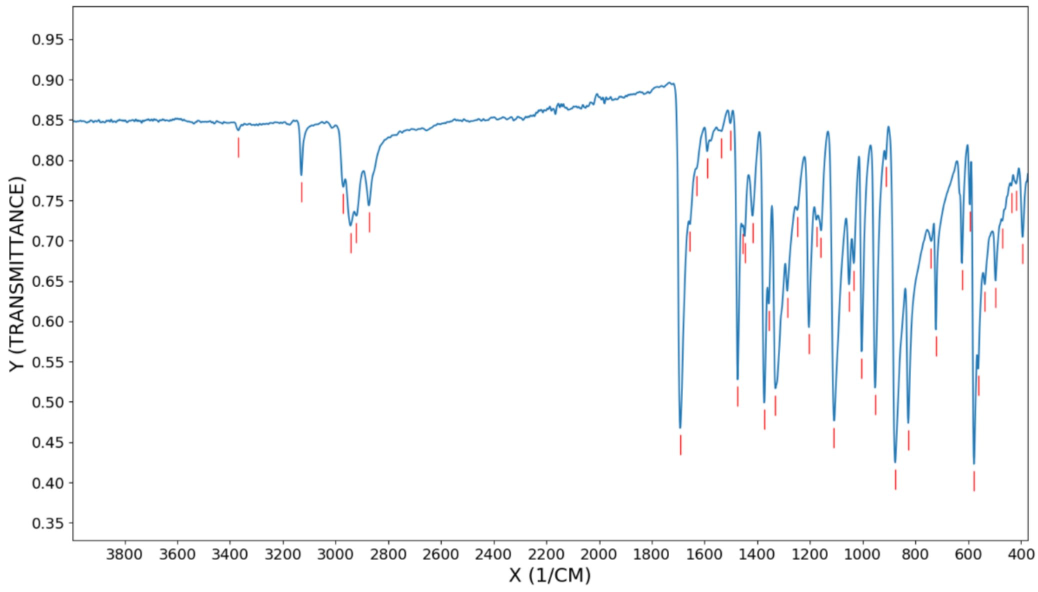

(6-Cyclopentyl-4,6-dihydro-3*H*-pyrazolo[3,4-*d*][1,2,3]triazin-3-yl)(phenyl)methanone (5g)

CHMO:0000593 | 1H nuclear magnetic resonance spectroscopy (1H NMR)

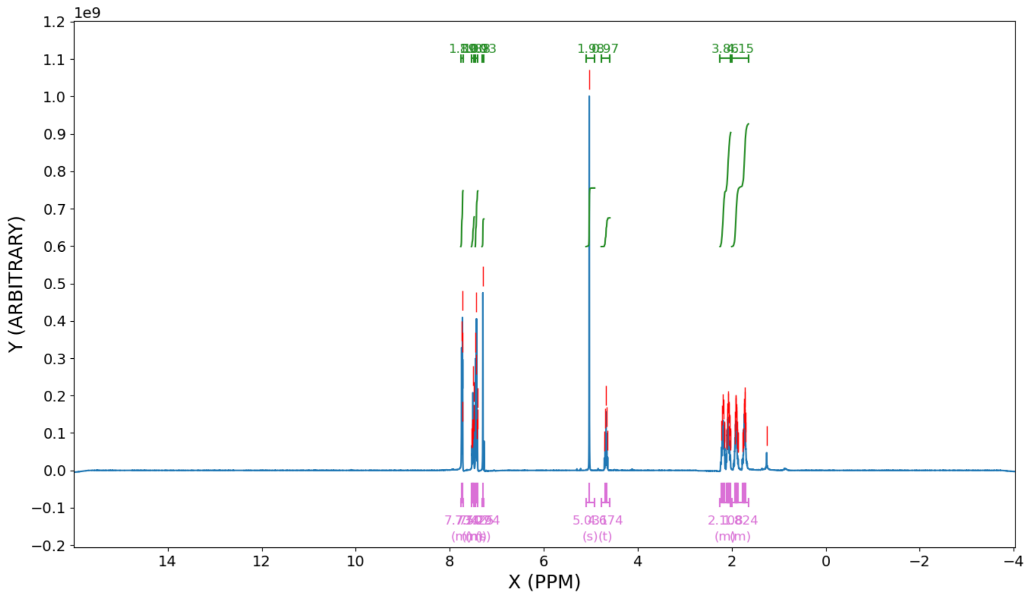

CHMO:0000595 | 13C nuclear magnetic resonance spectroscopy (13C NMR)

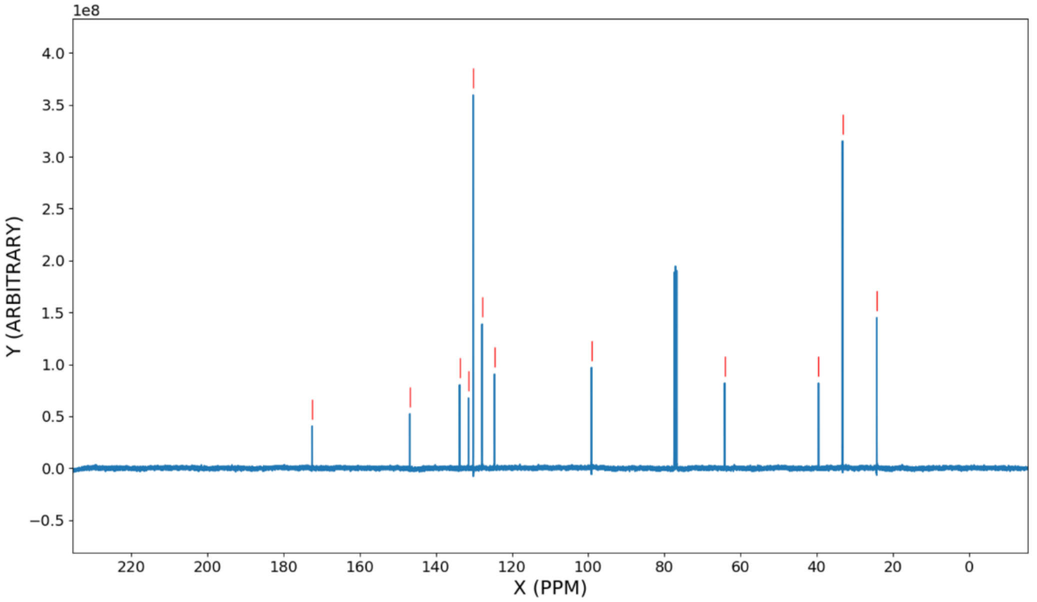

CHMO:0000470 | mass spectrometry (MS)

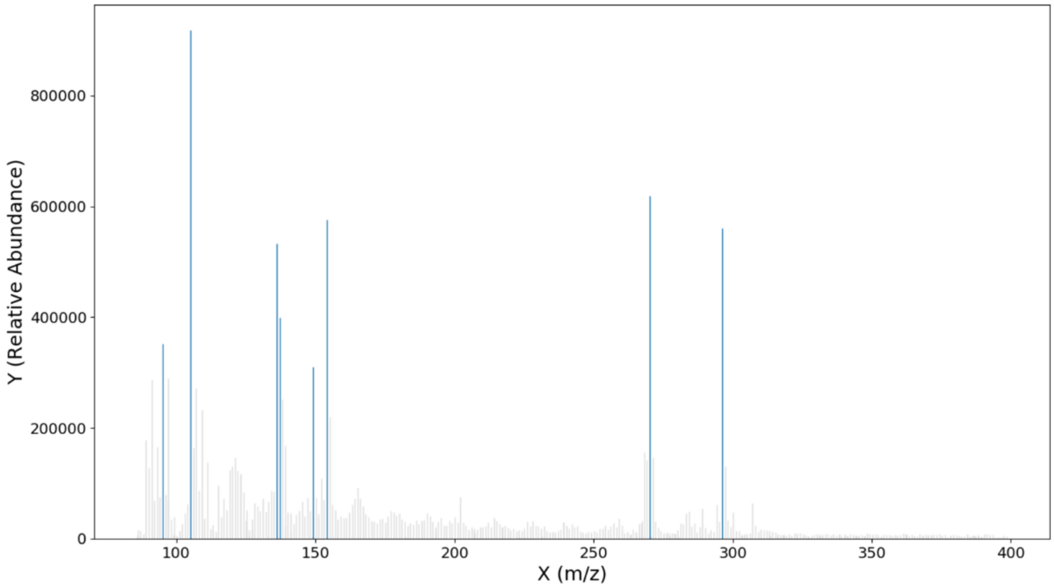

CHMO:0000630 | infrared absorption spectroscopy (IR)

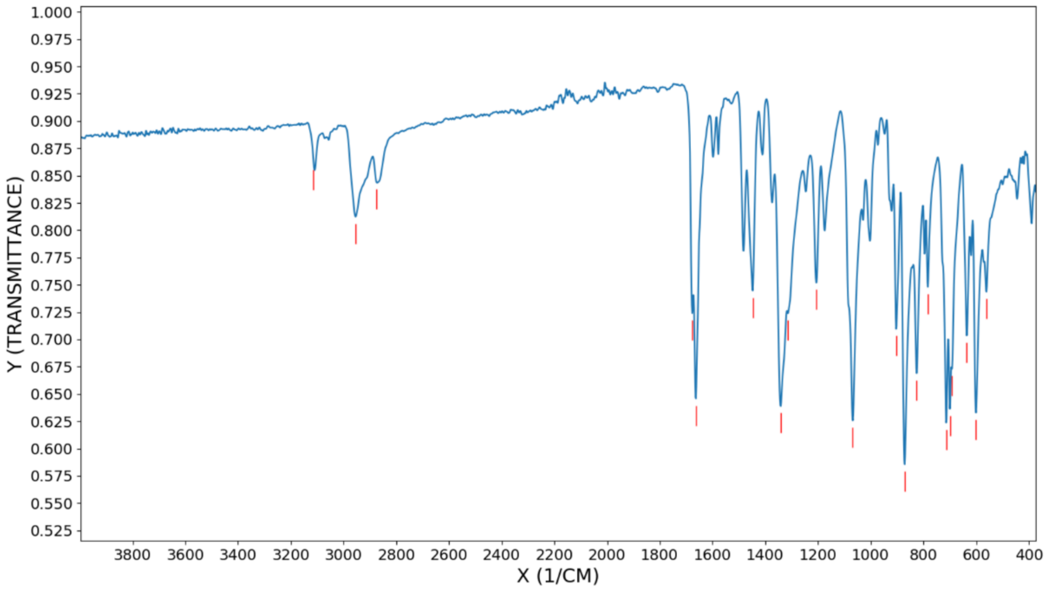

1-(6-Isobutyl-4,6-dihydro-3*H*-pyrazolo[3,4-*d*][1,2,3]triazin-3-yl)ethan-1-one (5h)

CHMO:0000593 | 1H nuclear magnetic resonance spectroscopy (1H NMR)

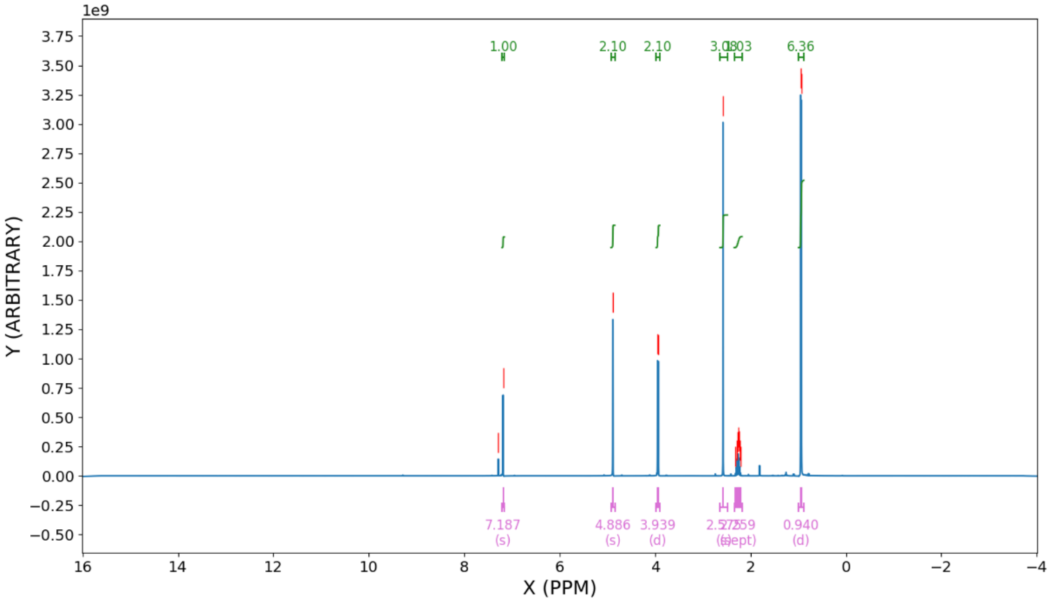

CHMO:0000595 | 13C nuclear magnetic resonance spectroscopy (13C NMR)

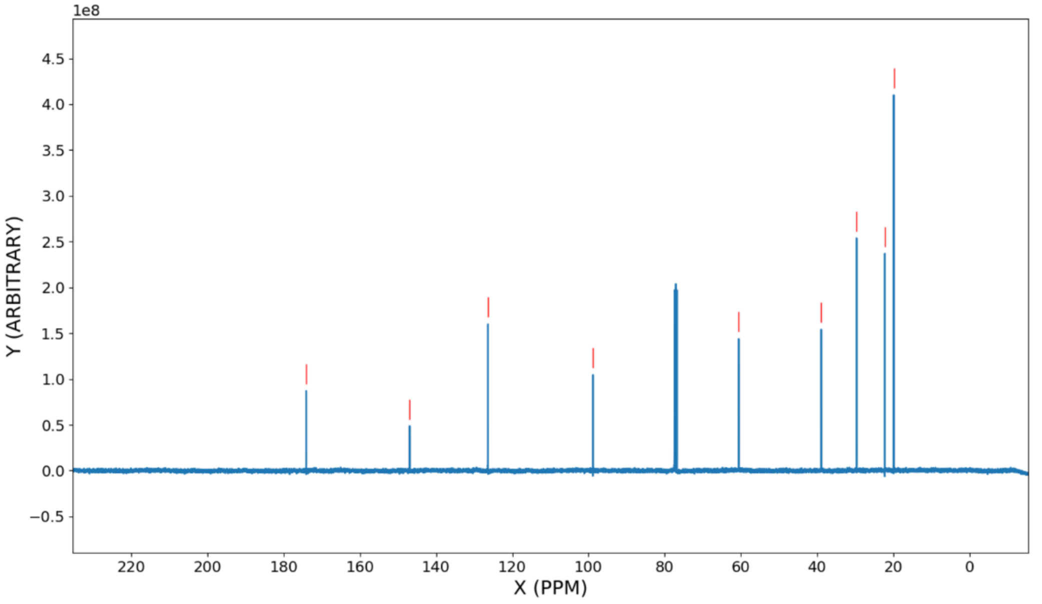

CHMO:0000563 | fast-atom bombardment mass spectrometry (FABMS)

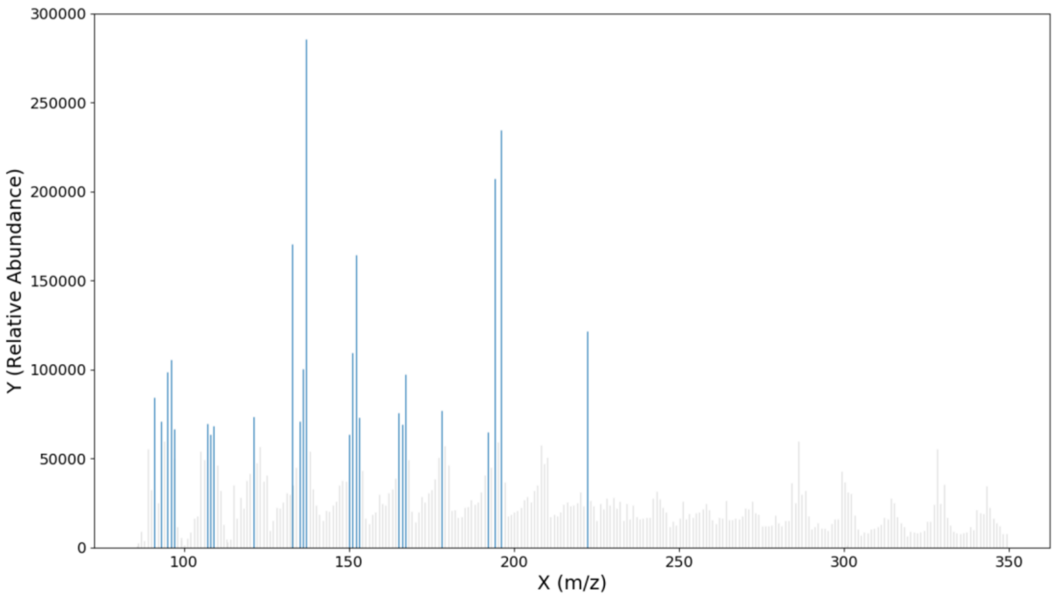

2-(3-Acetyl-3,4-dihydro-6*H*-pyrazolo[3,4-*d*][1,2,3]triazin-6-yl)ethyl acetate (5i)

CHMO:0000593 | 1H nuclear magnetic resonance spectroscopy (1H NMR)

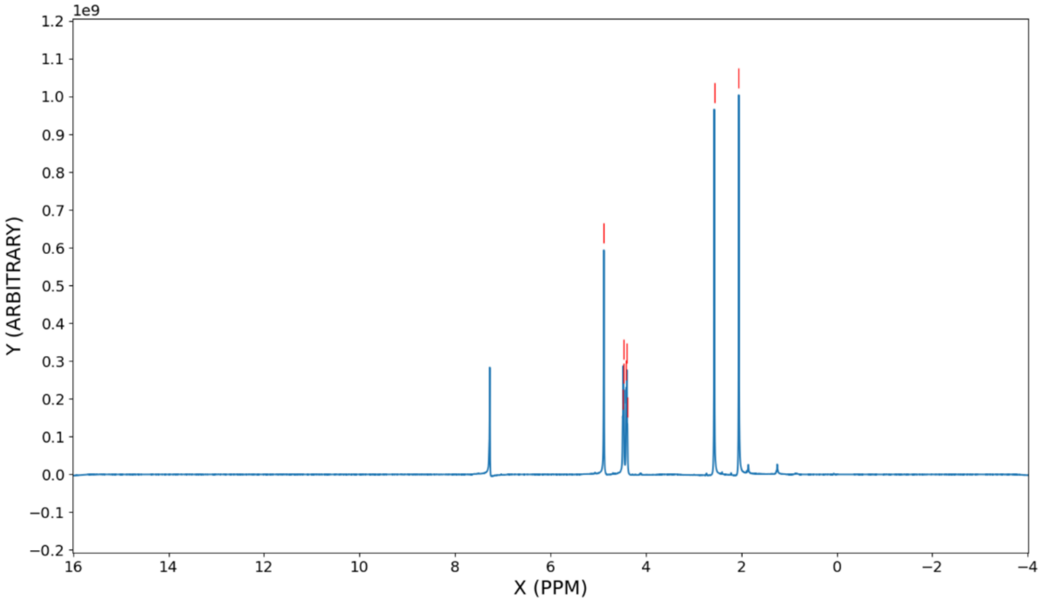

CHMO:0000595 | 13C nuclear magnetic resonance spectroscopy (13C NMR)

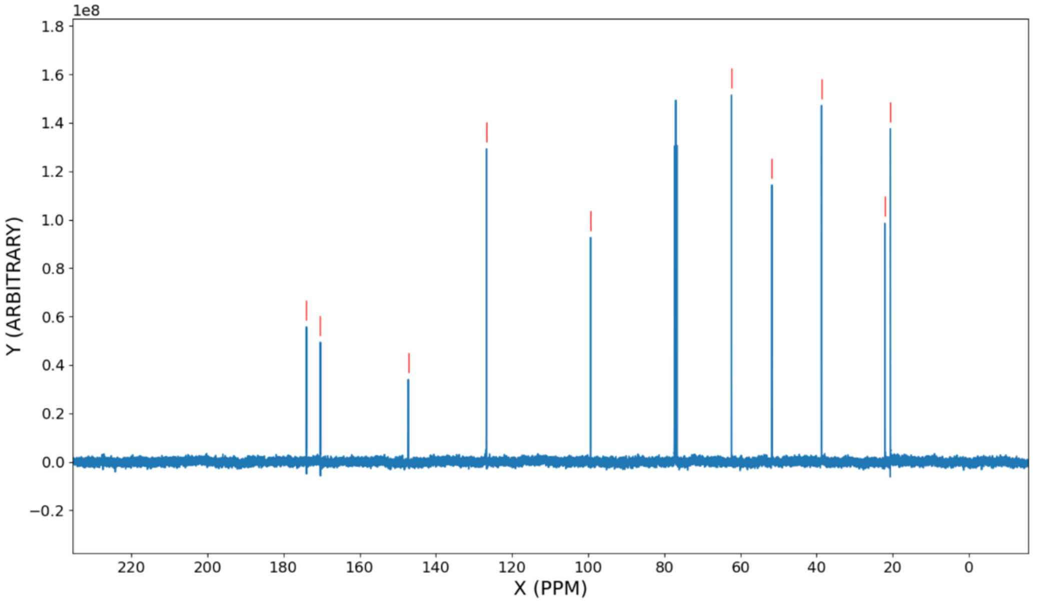

CHMO:0000563 | fast-atom bombardment mass spectrometry (FABMS)

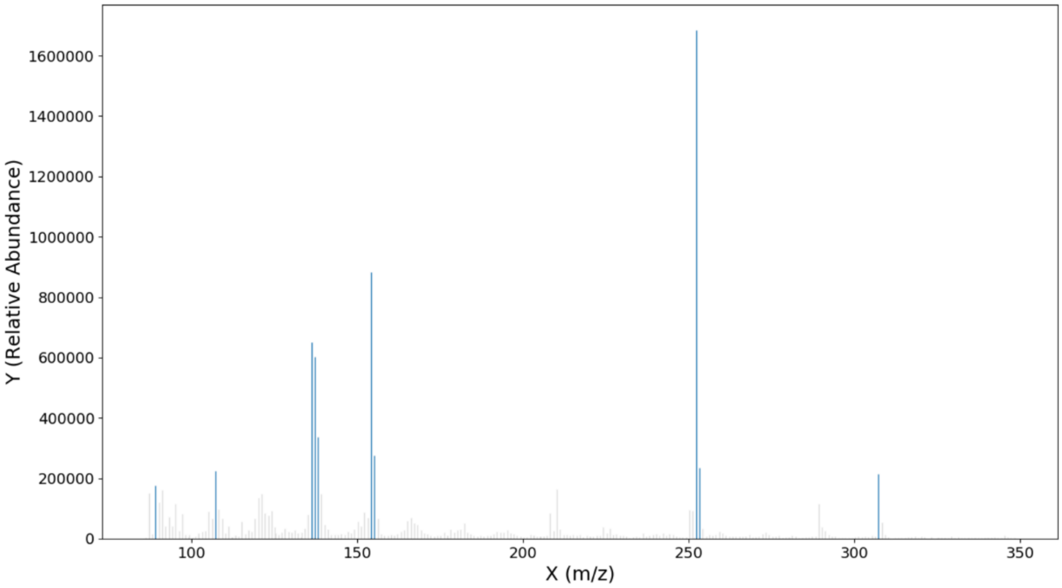

CHMO:0000630 | infrared absorption spectroscopy (IR)

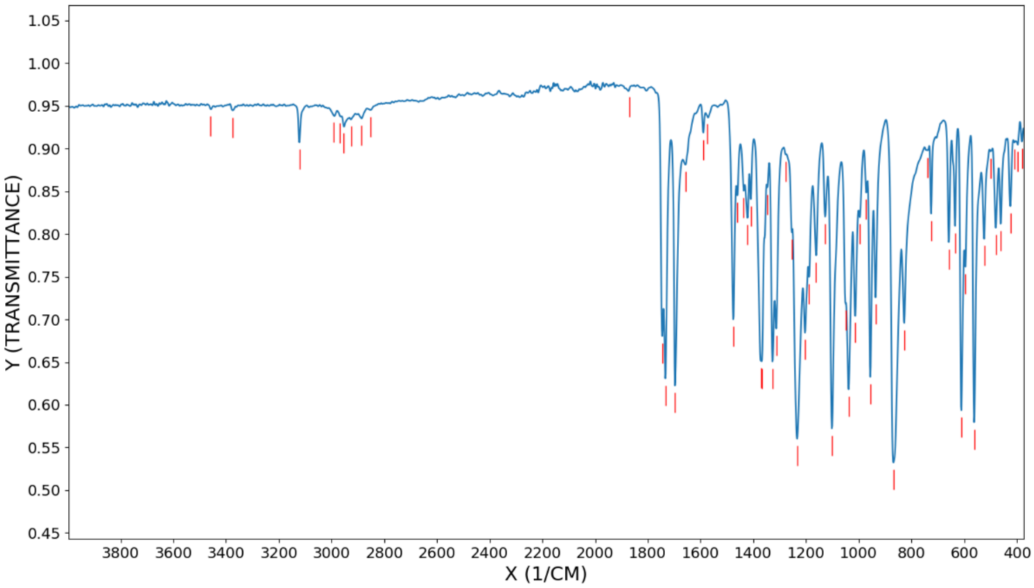

Supplement: File 2 — Copies of spectra. [file Beilstein_J_Org_Chem-17-2773-s002.pdf]
